# Supplementary material for: RHEB neddylation by the UBE2F-SAG axis enhances mTORC1 activity and aggravates liver tumorigenesis
Source: EMBO J. 2025 Jan 6;44(4):1185–219. doi: 10.1038/s44318-024-00353-5 (PMC11832924; doi:10.1038/s44318-024-00353-5)
Supplement: Supplementary file 1 — Appendix [file 44318_2024_353_MOESM1_ESM.pdf]

Appendix Figure for

RHEB neddylation by the UBE2F-SAG axis enhances mTORC1 activity and aggravates liver tumorigenesis

Fengwu Zhang, Xiufang Xiong, Zhijian Li, Haibo Wang, Weilin Wang, Yongchao Zhao*, and Yi Sun*

Table of Content:

Appendix Figure S1	Page6
Appendix Figure S2	Page11
Appendix Figure S3	Page13

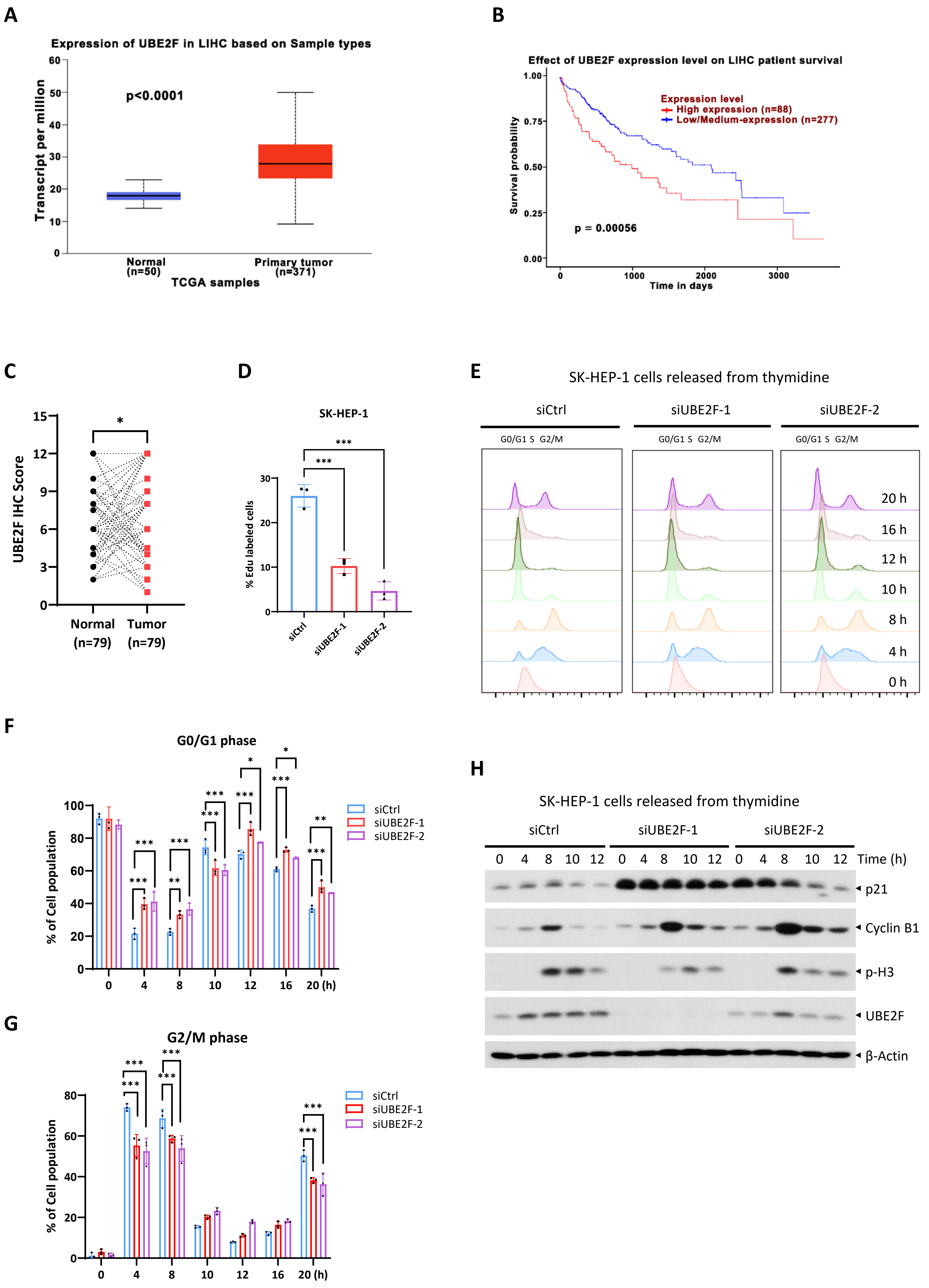
A
Expression of UBE2F in LIHC based on Sample types
Transcript per million
p<0.0001
Normal (n=50)
Primary tumor (n=371)
TCGA samples
B
Effect of UBE2F expression level on LIHC patient survival
Survival probability
Expression level
High expression (n=88)
Low/Medium-expression (n=277)
p = 0.00056
Time in days
C
UBE2F IHC Score
Normal (n=79)
Tumor (n=79)
D
SK-HEP-1
% Edu labeled cells
siCtrl
siUBE2F-1
siUBE2F-2
E
SK-HEP-1 cells released from thymidine
siCtrl
siUBE2F-1
siUBE2F-2
G0/G1 S G2/M
20 h
16 h
12 h
10 h
8 h
4 h
0 h
F
G0/G1 phase
% of Cell population
siCtrl
siUBE2F-1
siUBE2F-2
0 4 8 10 12 16 20 (h)
G
G2/M phase
% of Cell population
siCtrl
siUBE2F-1
siUBE2F-2
0 4 8 10 12 16 20 (h)
H
SK-HEP-1 cells released from thymidine
siCtrl
siUBE2F-1
siUBE2F-2
Time (h)
p21
Cyclin B1
p-H3
UBE2F
β-Actin

Appendix Figure S1: UBE2F is overexpressed in liver cancer tissues, and knockdown of UBE2F induces growth inhibition

(A) The expression of *UBE2F* mRNA level between hepatocellular carcinoma tumor and normal liver tissue, data from TCGA database. (B) The association between *UBE2F* mRNA levels and the survival probability in HCC patients based on TCGA database. (C) This figure presents the same data of Figure 1B, but in a different chart format. Data were analyzed by student's *t*-test. (D) SK-HEP-1 cells were transfected with the indicated siRNAs for 48 h and then labeled with 20 μM EdU for 2 h. The cells were then fixed and incubated with Azide 488 before being subjected to flow cytometry. The statistical analysis of the percentage of proliferating cells is shown. Data were presented as mean ± SEM from three independent experiments and analyzed by one-way ANOVA. *** $p < 0.001$. (E-H) Cells transfected with the indicated siRNA were synchronized in the G1/S phase using 2 mM thymidine, followed by release for the indicated time periods. The cells were then subjected to FACS analysis (E) or immunoblotting with the indicated antibodies (H), and the statistics of the percentage of cells at the G0/G1 (F) and G2/M (G) phases are shown. Data were presented as mean ± SEM from three independent experiments and analyzed by two-way ANOVA. * $p < 0.05$; ** $p < 0.01$; *** $p < 0.001$.

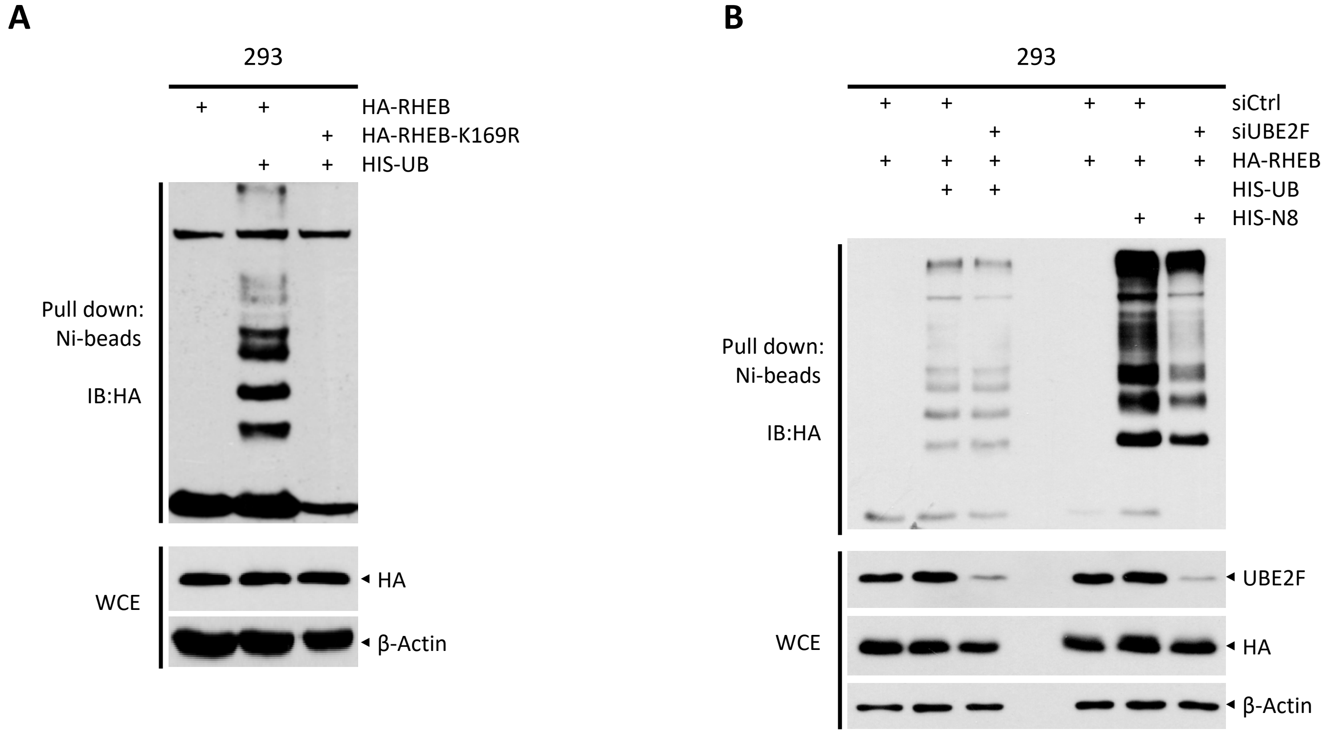
A
293
HA-RHEB
HA-RHEB-K169R
HIS-UB
Pull down:
Ni-beads
IB:HA
WCE
HA
β-Actin
B
293
siCtrl
siUBE2F
HA-RHEB
HIS-UB
HIS-N8
Pull down:
Ni-beads
IB:HA
WCE
UBE2F
HA
β-Actin

Appendix Figure S2. UBE2F differentially regulates RHEB neddylation, not affecting its ubiquitylation.

(A) HEK293 cells were transfected with the indicated plasmids for 48 h, and then lysed under denatured condition. His-tagged neddylated proteins were pulled down by Ni-NTA beads, and then subjected to IB analysis with indicated antibodies. (B) HEK293 cells were transfected with the indicated siRNAs for 24 h, followed by co-transfection with 6 μg HA-RHEB and 9 μg HIS-N8 or HIS-Ub plasmids. For detection of RHEB ubiquitylation, cells were treated with 20 μM MG132 for 6 h before being harvested. Cells were then lysed in denatured buffer, and neddylated or ubiquitinated proteins were pulled down using Ni-NTA beads, followed by IB analysis using an anti-HA antibody.

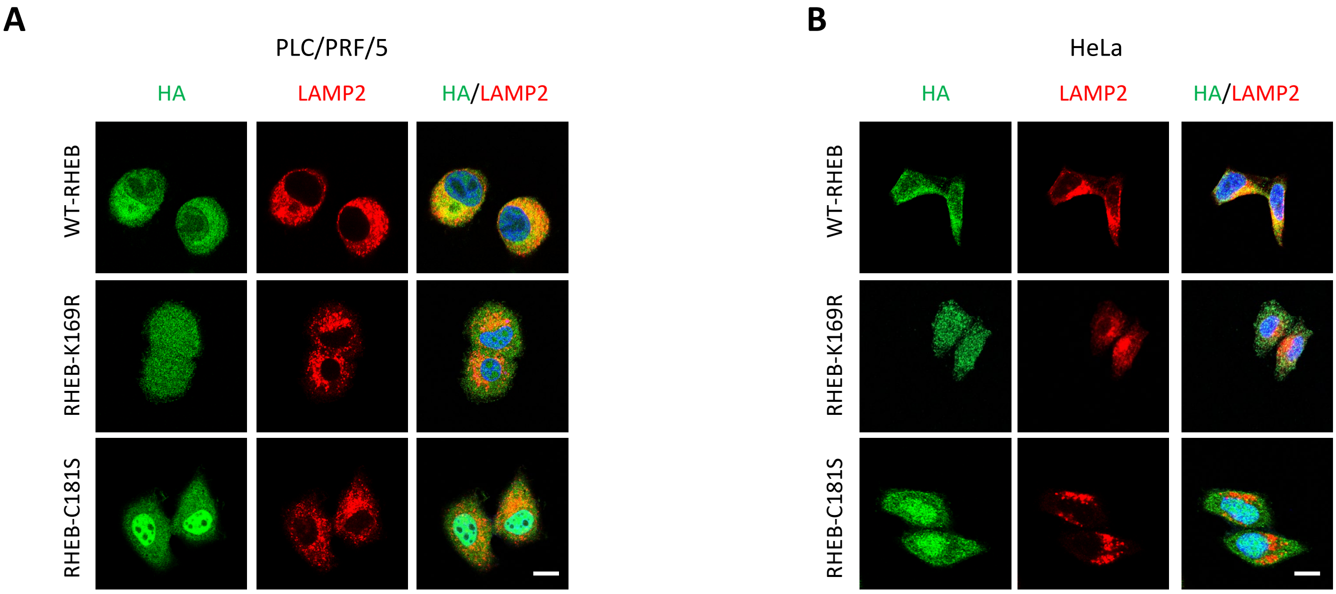
A
PLC/PRF/5
HA
LAMP2
HA/LAMP2
WT-RHEB
RHEB-K169R
RHEB-C181S
B
HeLa
HA
LAMP2
HA/LAMP2
WT-RHEB
RHEB-K169R
RHEB-C181S

Appendix Figure S3: RHEB neddylation-dead mutant weakens its lysosome localization and activity

(A and B) Cells were transfected with indicated plasmids for 48 h, and then immunostained with HA (green) and LAMP2 (red), followed by photography under a confocal fluorescent microscope. Scale bar, 10 μm.
