# Supplementary figures and images for: RHEB neddylation by the UBE2F-SAG axis enhances mTORC1 activity and aggravates liver tumorigenesis (part 1 of 3)
Source: EMBO J. 2025 Jan 6;44(4):1185–219. doi: 10.1038/s44318-024-00353-5 (PMC11832924; doi:10.1038/s44318-024-00353-5)

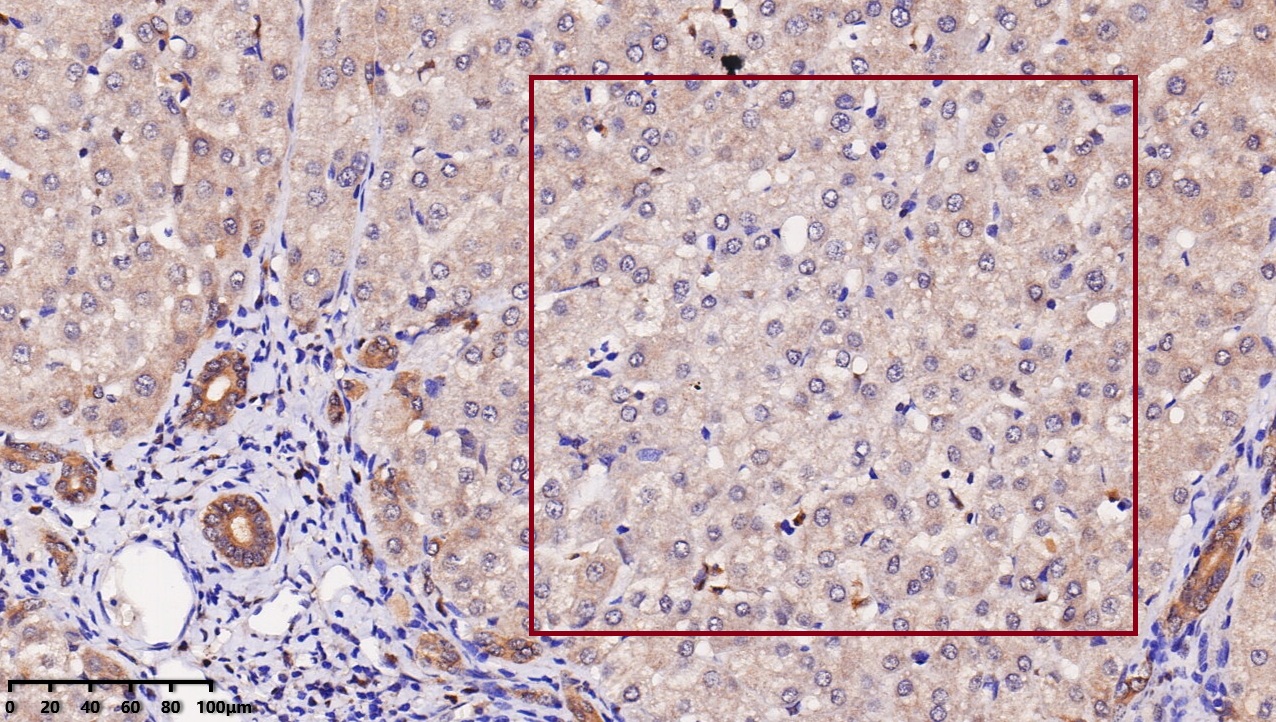

Supplement: Supplementary file 3 — Source data Fig. 1 [file 44318_2024_353_MOESM3_ESM.zip › Figure 1/Figure 1/1A/UBE2F High Normal tissue insert.jpg]

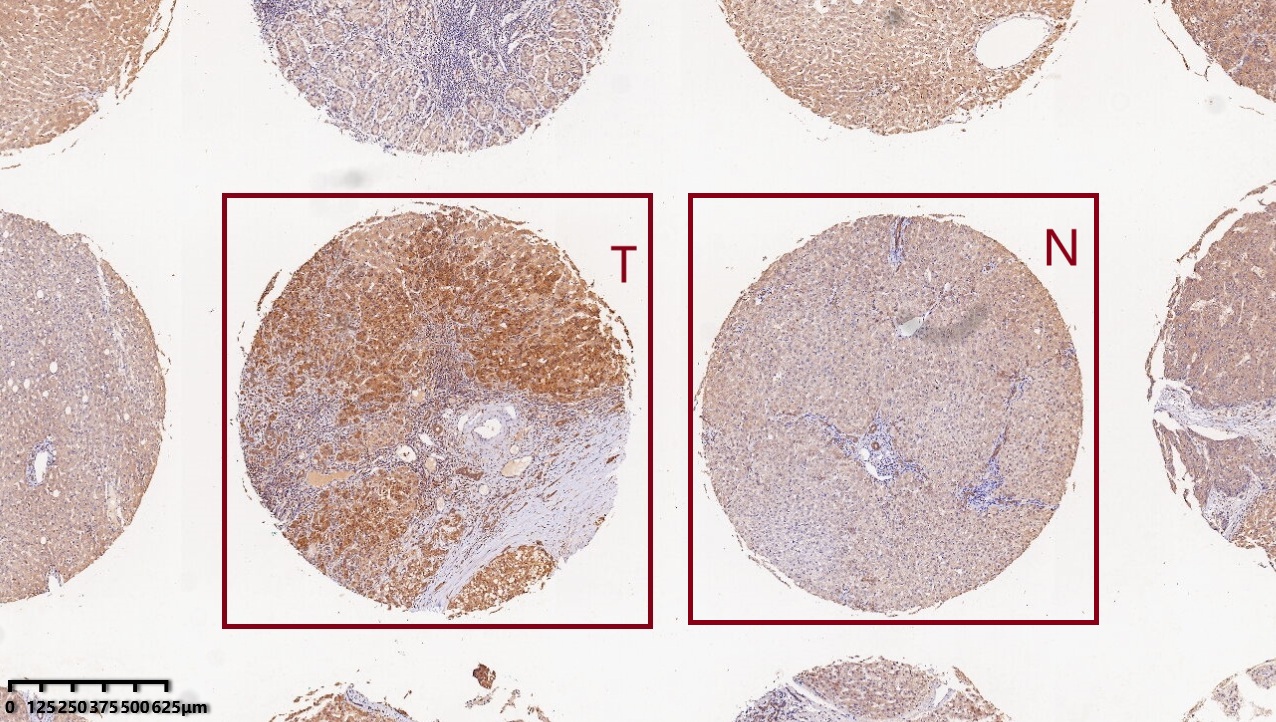

Supplement: Supplementary file 3 — Source data Fig. 1 [file 44318_2024_353_MOESM3_ESM.zip › Figure 1/Figure 1/1A/UBE2F High Normal Tumor tissue.jpg]

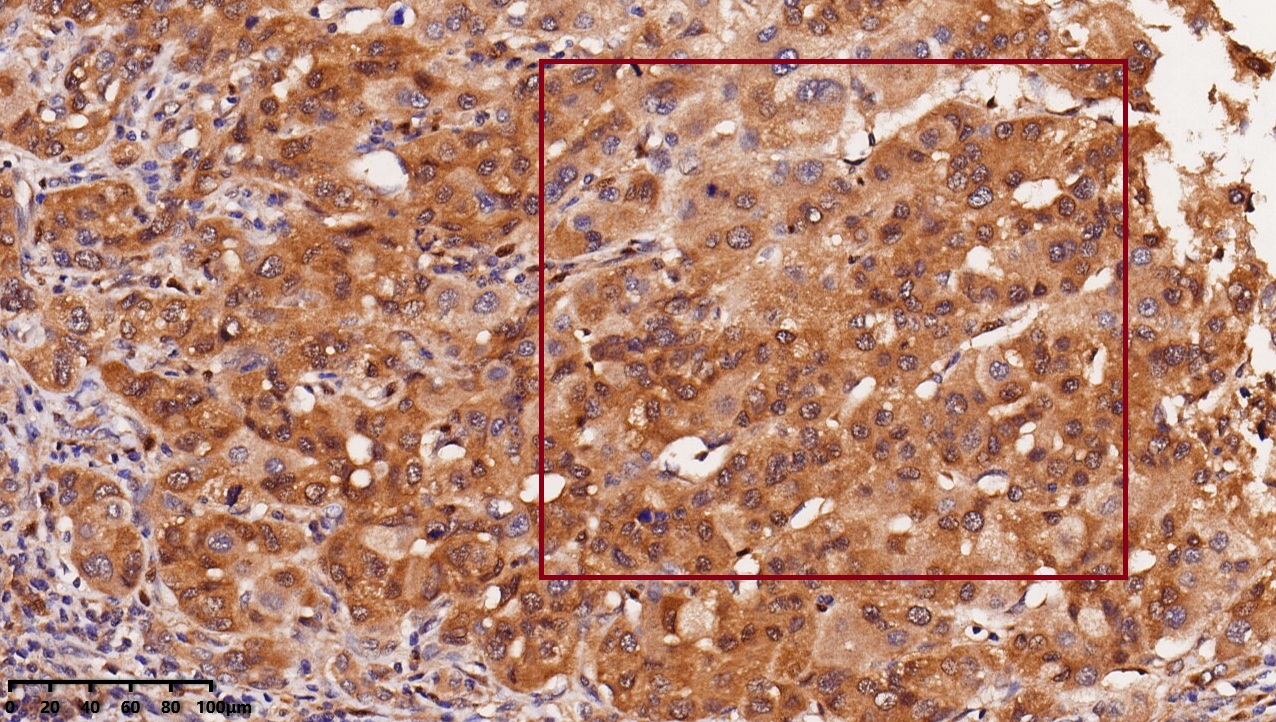

Supplement: Supplementary file 3 — Source data Fig. 1 [file 44318_2024_353_MOESM3_ESM.zip › Figure 1/Figure 1/1A/UBE2F High Tumor tissue insert.jpg]

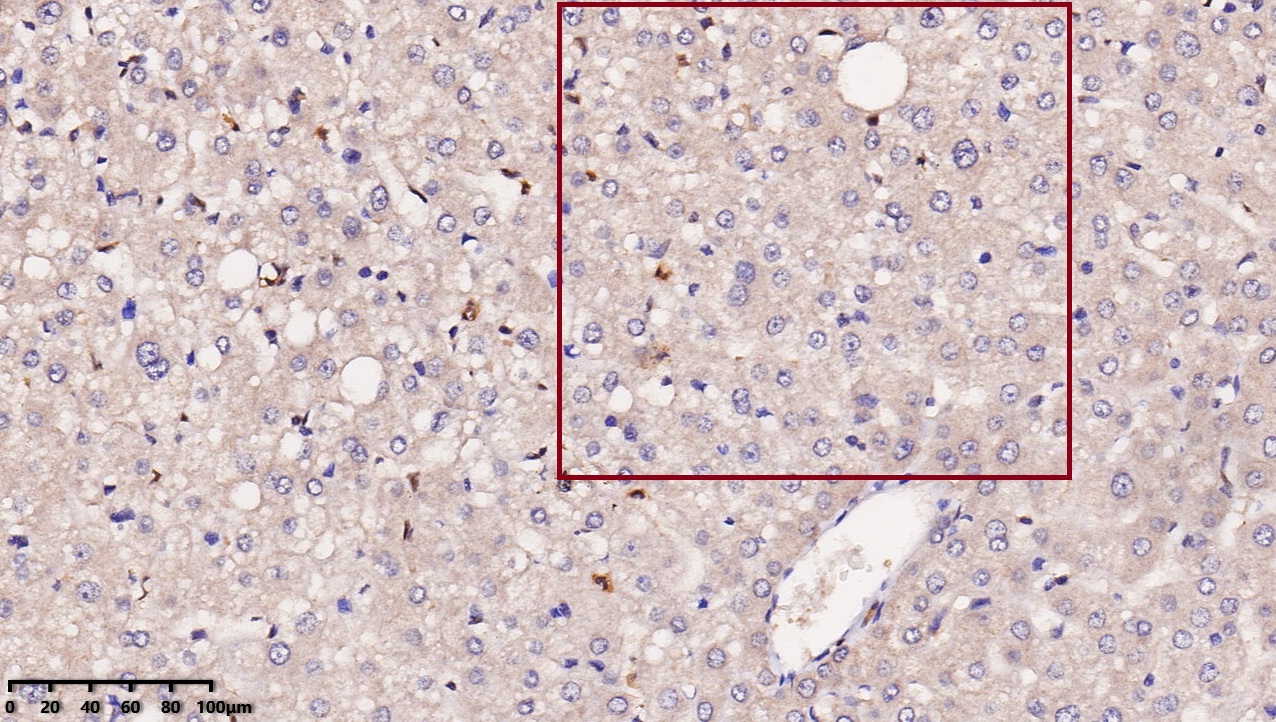

Supplement: Supplementary file 3 — Source data Fig. 1 [file 44318_2024_353_MOESM3_ESM.zip › Figure 1/Figure 1/1A/UBE2F Low Normal Tisusue insert.jpg]

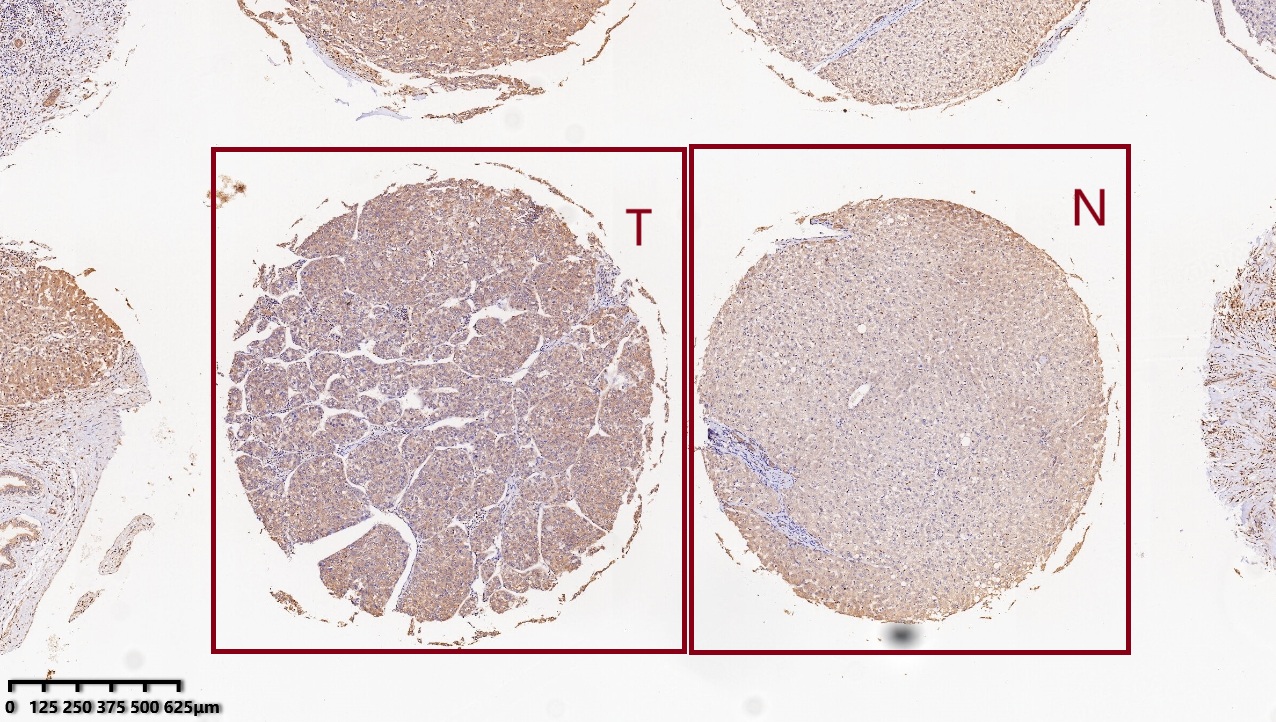

Supplement: Supplementary file 3 — Source data Fig. 1 [file 44318_2024_353_MOESM3_ESM.zip › Figure 1/Figure 1/1A/UBE2F Low Normal Tumor tissue.jpg]

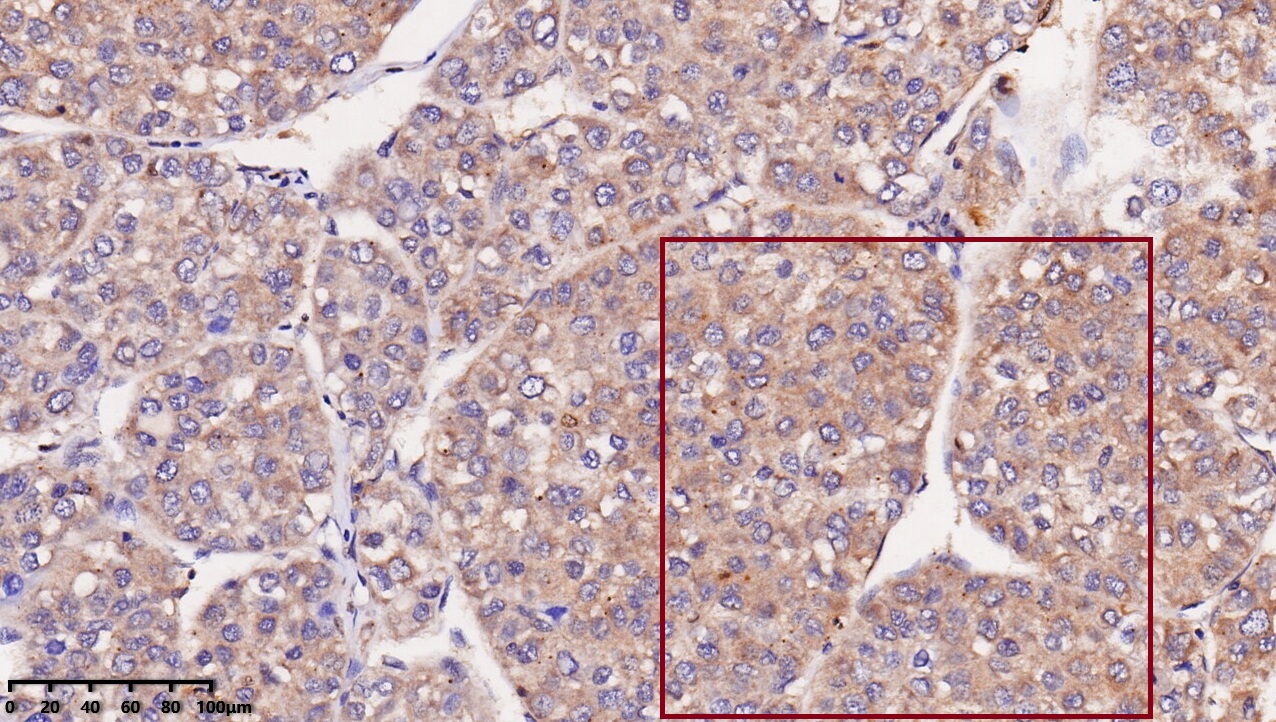

Supplement: Supplementary file 3 — Source data Fig. 1 [file 44318_2024_353_MOESM3_ESM.zip › Figure 1/Figure 1/1A/UBE2F Low Tumor tissue insert.jpg]

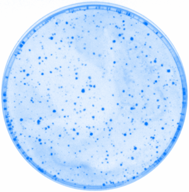

Supplement: Supplementary file 3 — Source data Fig. 1 [file 44318_2024_353_MOESM3_ESM.zip › Figure 1/Figure 1/1E/shGFP.tif]

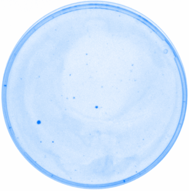

Supplement: Supplementary file 3 — Source data Fig. 1 [file 44318_2024_353_MOESM3_ESM.zip › Figure 1/Figure 1/1E/shUBE2F-1.png]

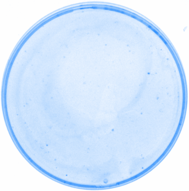

Supplement: Supplementary file 3 — Source data Fig. 1 [file 44318_2024_353_MOESM3_ESM.zip › Figure 1/Figure 1/1E/shUBE2F-2.png]

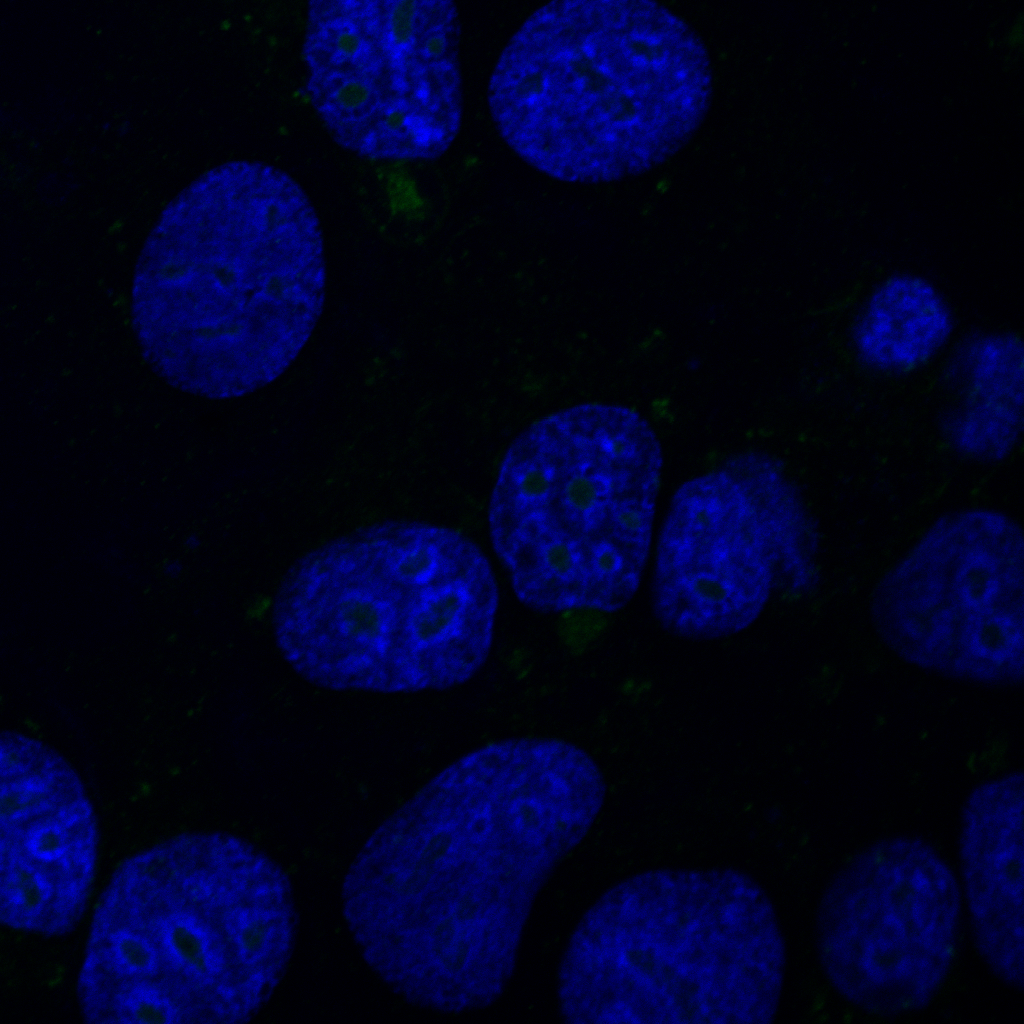

Supplement: Supplementary file 3 — Source data Fig. 1 [file 44318_2024_353_MOESM3_ESM.zip › Figure 1/Figure 1/1I/PLCPRF5 siCtrl/HP_PLC SINC 60X2.5-3_RGB.tif]

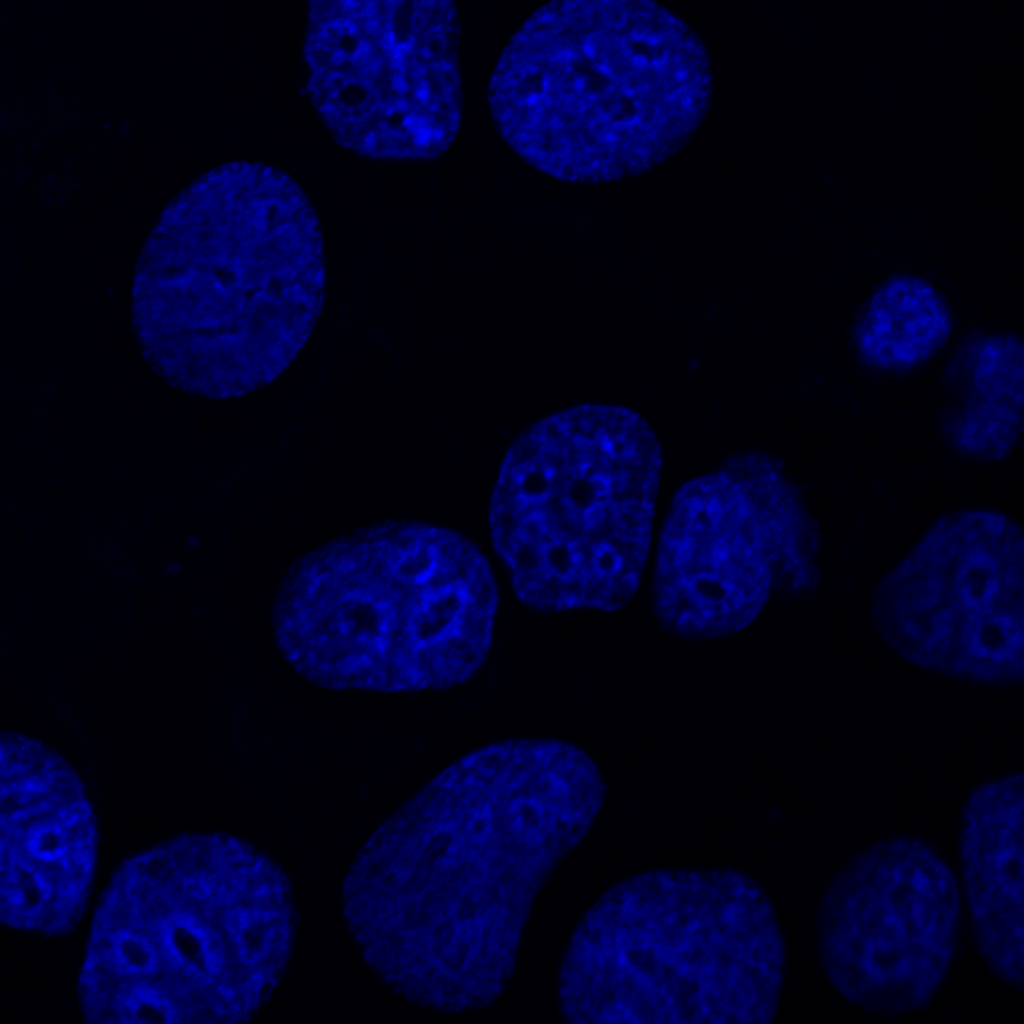

Supplement: Supplementary file 3 — Source data Fig. 1 [file 44318_2024_353_MOESM3_ESM.zip › Figure 1/Figure 1/1I/PLCPRF5 siCtrl/HP_PLC SINC 60X2.5-3_RGB_C1.tif]

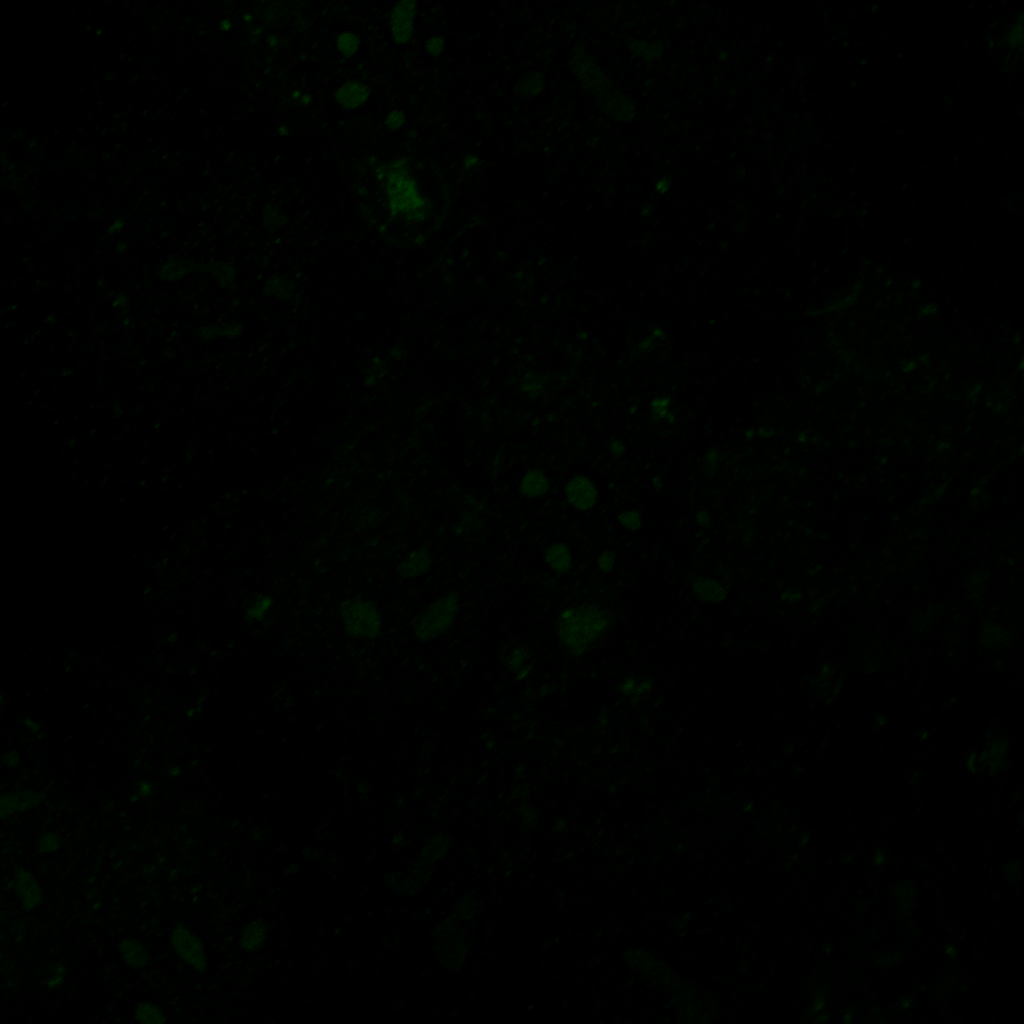

Supplement: Supplementary file 3 — Source data Fig. 1 [file 44318_2024_353_MOESM3_ESM.zip › Figure 1/Figure 1/1I/PLCPRF5 siCtrl/HP_PLC SINC 60X2.5-3_RGB_C2.tif]

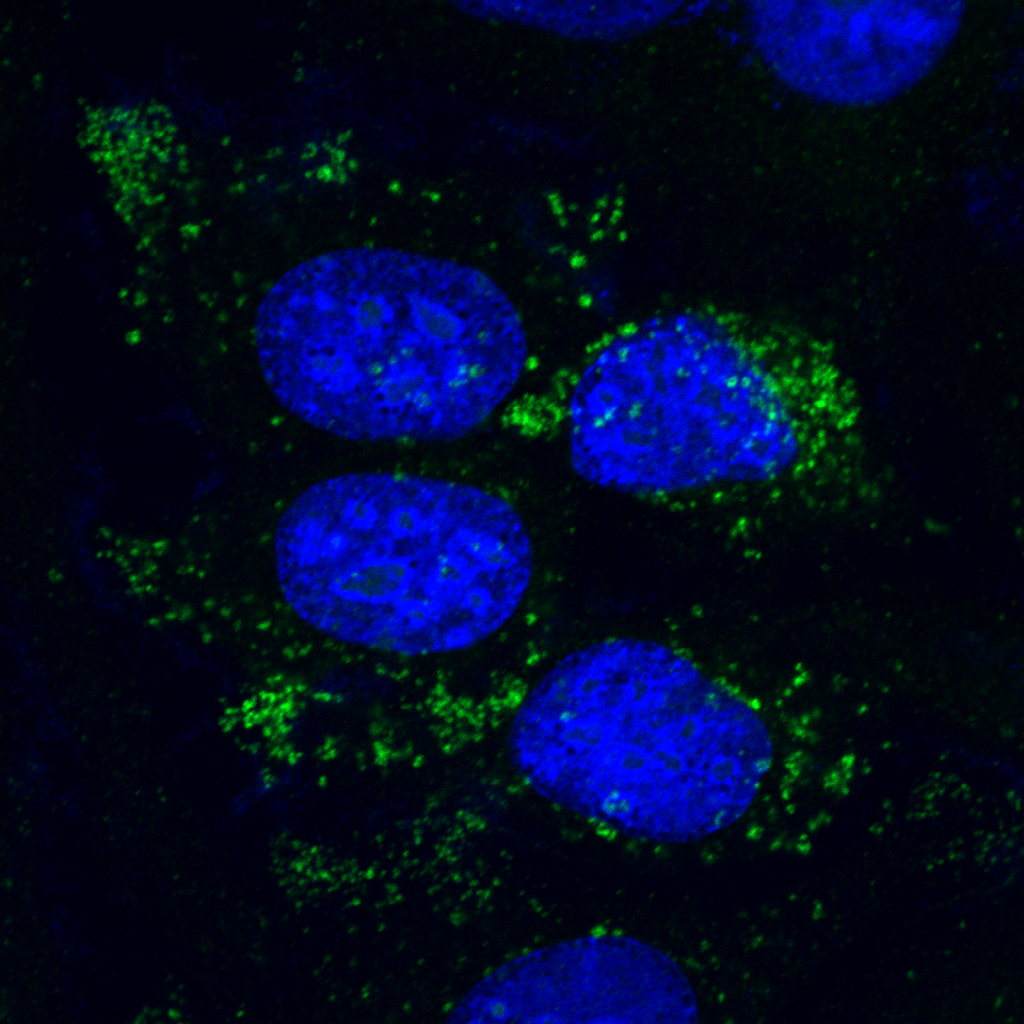

Supplement: Supplementary file 3 — Source data Fig. 1 [file 44318_2024_353_MOESM3_ESM.zip › Figure 1/Figure 1/1I/PLCPRF5 siUBE2F-1/HP_PLC SI2F-3 60X2.5-2_RGB.tif]

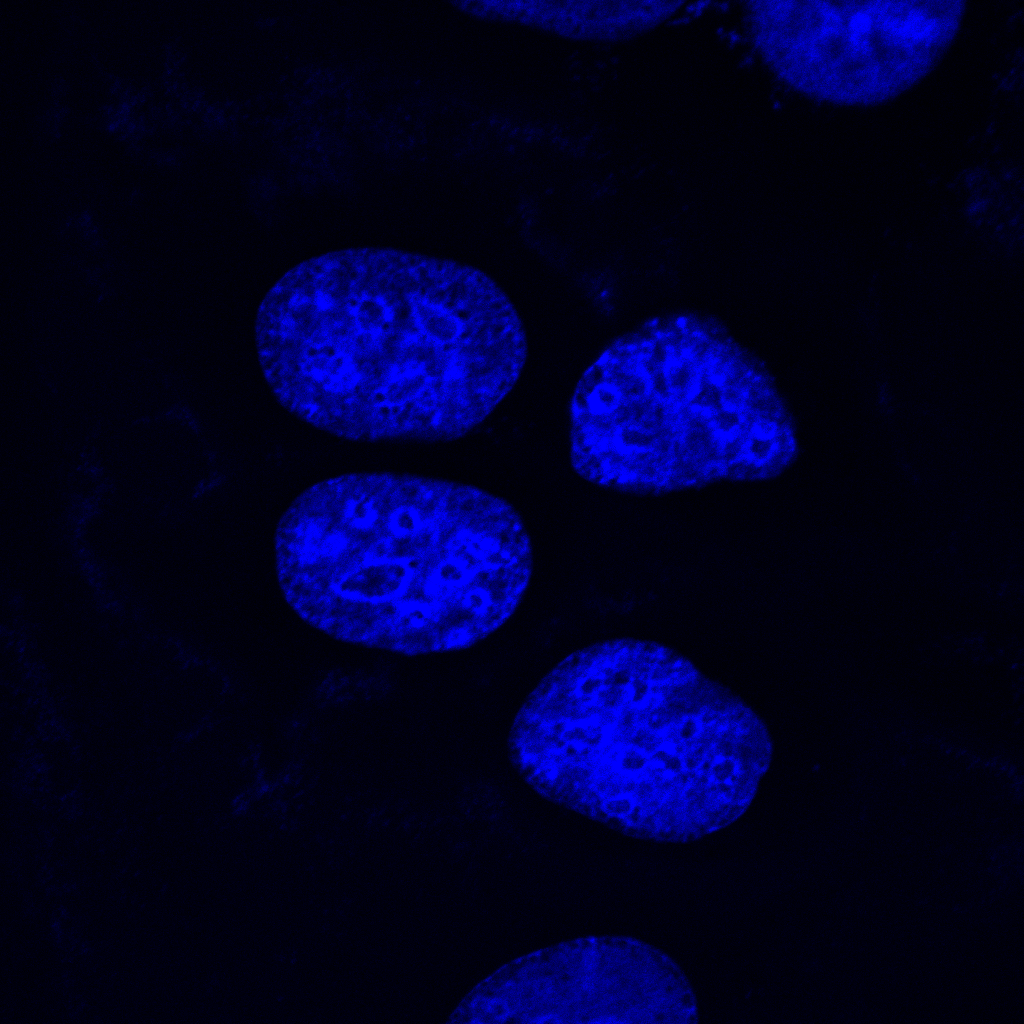

Supplement: Supplementary file 3 — Source data Fig. 1 [file 44318_2024_353_MOESM3_ESM.zip › Figure 1/Figure 1/1I/PLCPRF5 siUBE2F-1/HP_PLC SI2F-3 60X2.5-2_RGB_C1.tif]

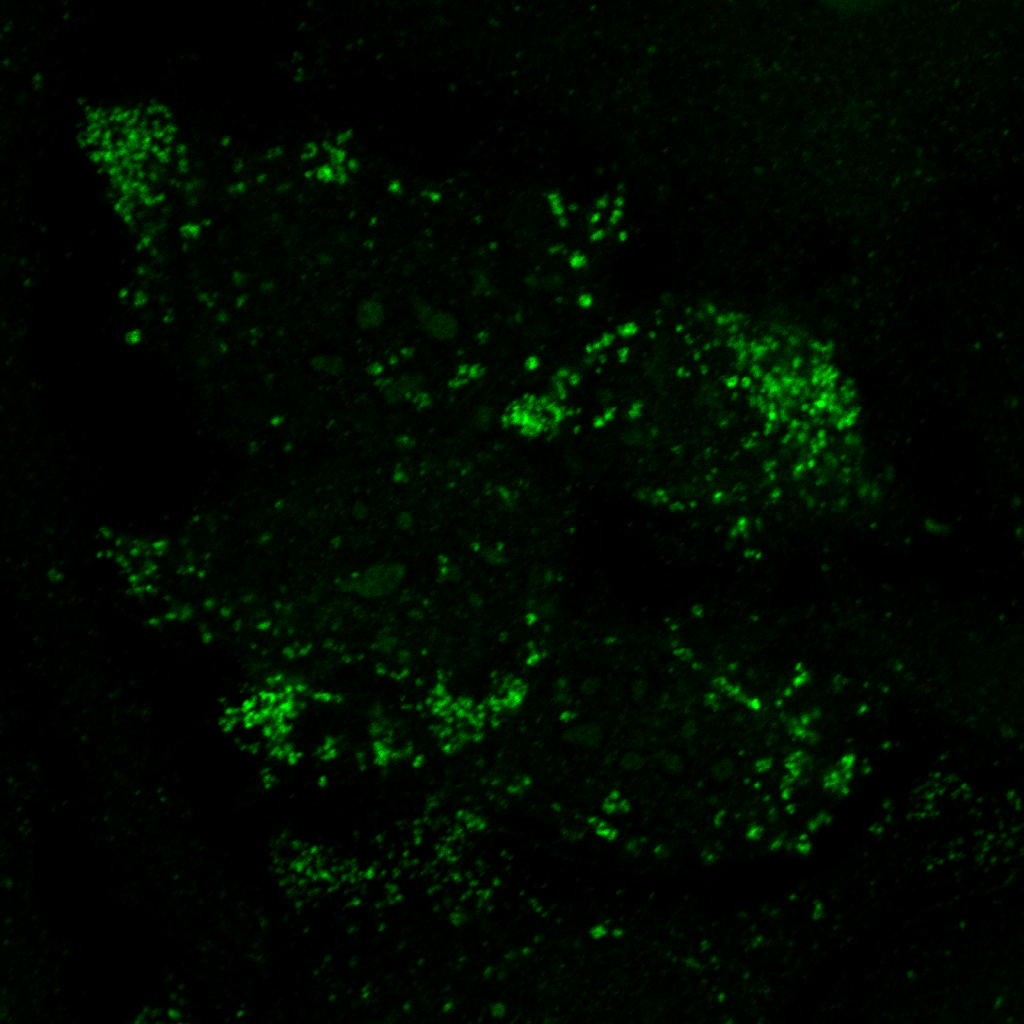

Supplement: Supplementary file 3 — Source data Fig. 1 [file 44318_2024_353_MOESM3_ESM.zip › Figure 1/Figure 1/1I/PLCPRF5 siUBE2F-1/HP_PLC SI2F-3 60X2.5-2_RGB_C2.tif]

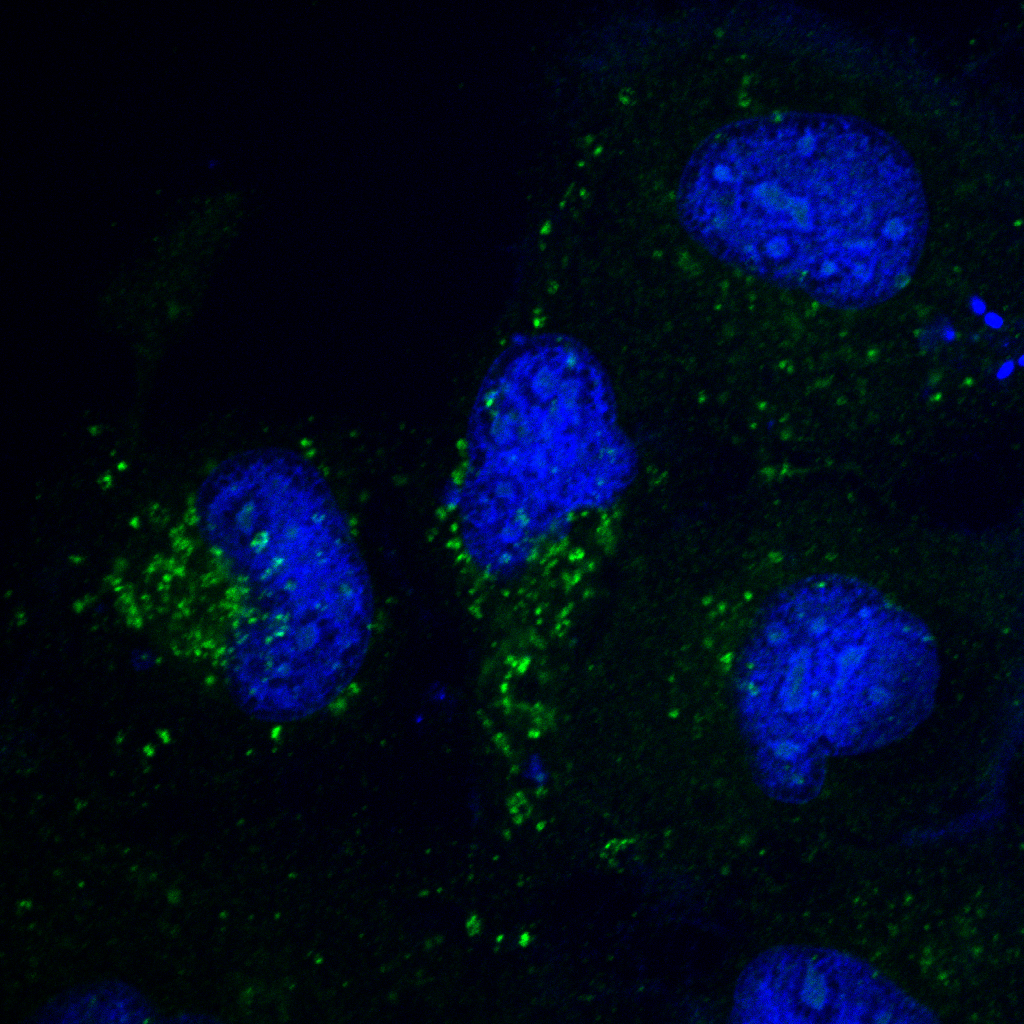

Supplement: Supplementary file 3 — Source data Fig. 1 [file 44318_2024_353_MOESM3_ESM.zip › Figure 1/Figure 1/1I/PLCPRF5 siUBE2F-2/HP_PLC SI2F-5 60X2.5-1_RGB.tif]

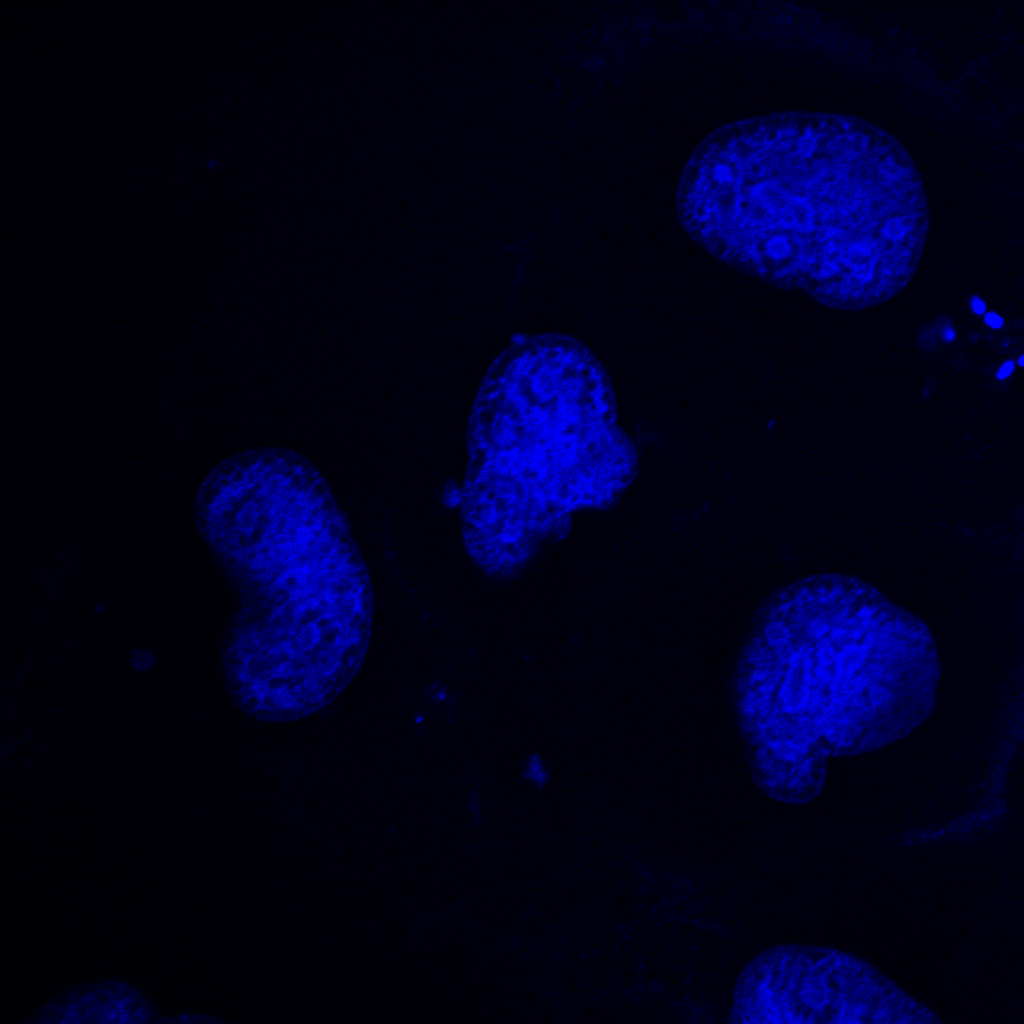

Supplement: Supplementary file 3 — Source data Fig. 1 [file 44318_2024_353_MOESM3_ESM.zip › Figure 1/Figure 1/1I/PLCPRF5 siUBE2F-2/HP_PLC SI2F-5 60X2.5-1_RGB_C1.tif]

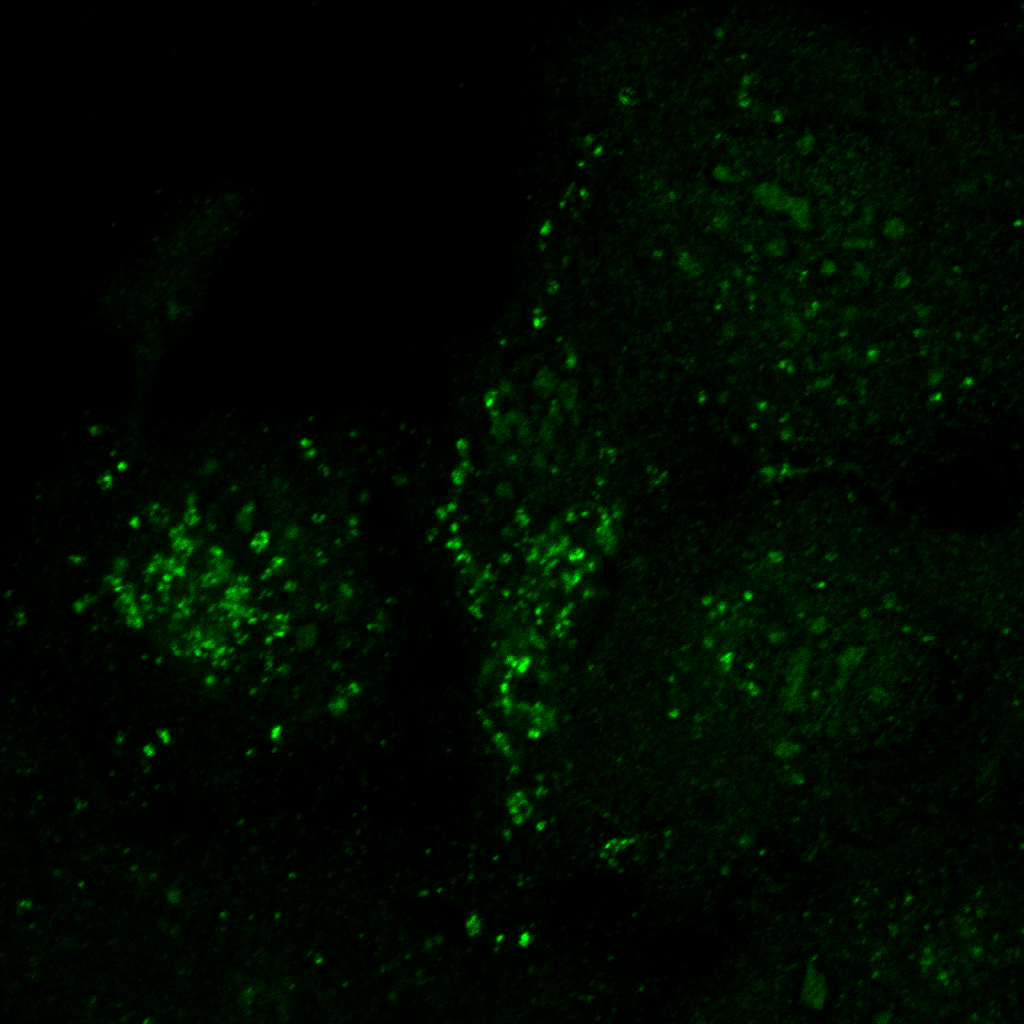

Supplement: Supplementary file 3 — Source data Fig. 1 [file 44318_2024_353_MOESM3_ESM.zip › Figure 1/Figure 1/1I/PLCPRF5 siUBE2F-2/HP_PLC SI2F-5 60X2.5-1_RGB_C2.tif]

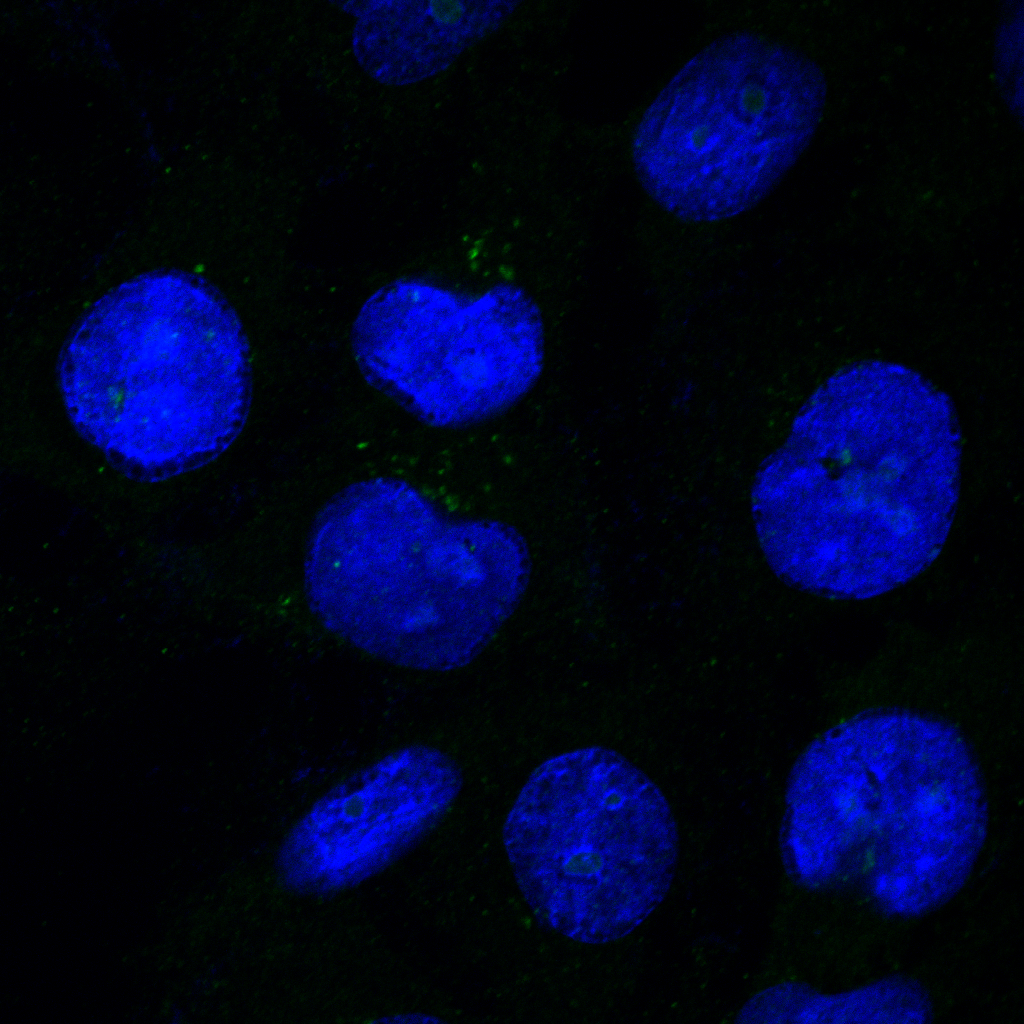

Supplement: Supplementary file 3 — Source data Fig. 1 [file 44318_2024_353_MOESM3_ESM.zip › Figure 1/Figure 1/1I/SKHEP1 siCtrl/HP_SK SINC 60X2.5-2_RGB.tif]

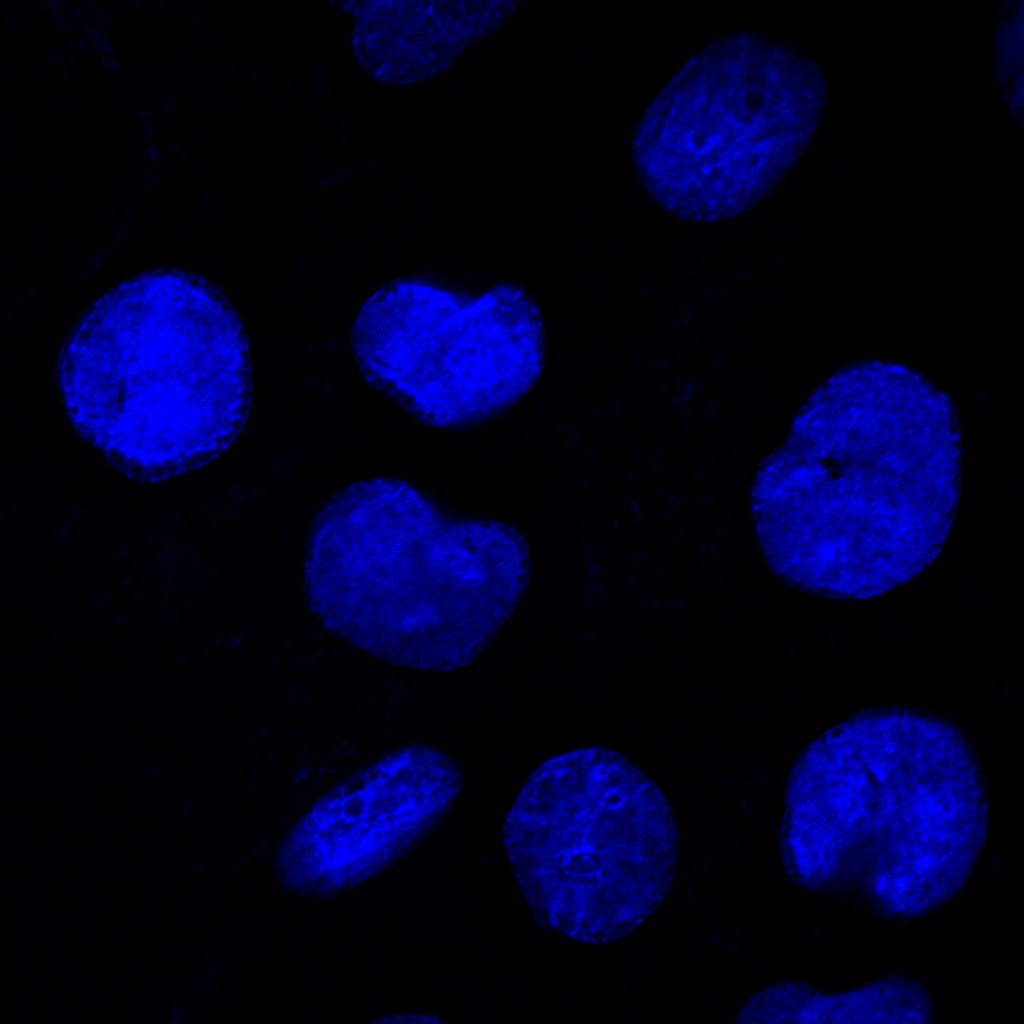

Supplement: Supplementary file 3 — Source data Fig. 1 [file 44318_2024_353_MOESM3_ESM.zip › Figure 1/Figure 1/1I/SKHEP1 siCtrl/HP_SK SINC 60X2.5-2_RGB_C1.tif]

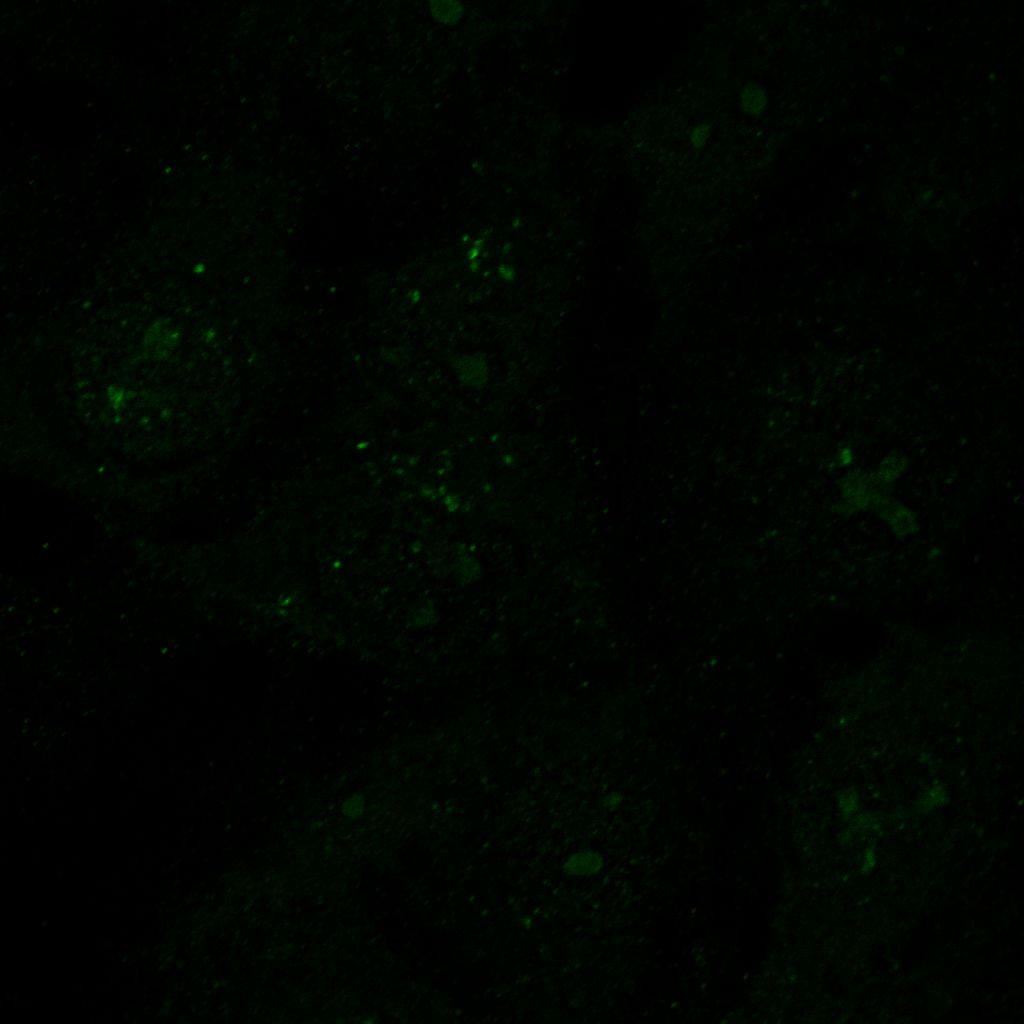

Supplement: Supplementary file 3 — Source data Fig. 1 [file 44318_2024_353_MOESM3_ESM.zip › Figure 1/Figure 1/1I/SKHEP1 siCtrl/HP_SK SINC 60X2.5-2_RGB_C2.tif]

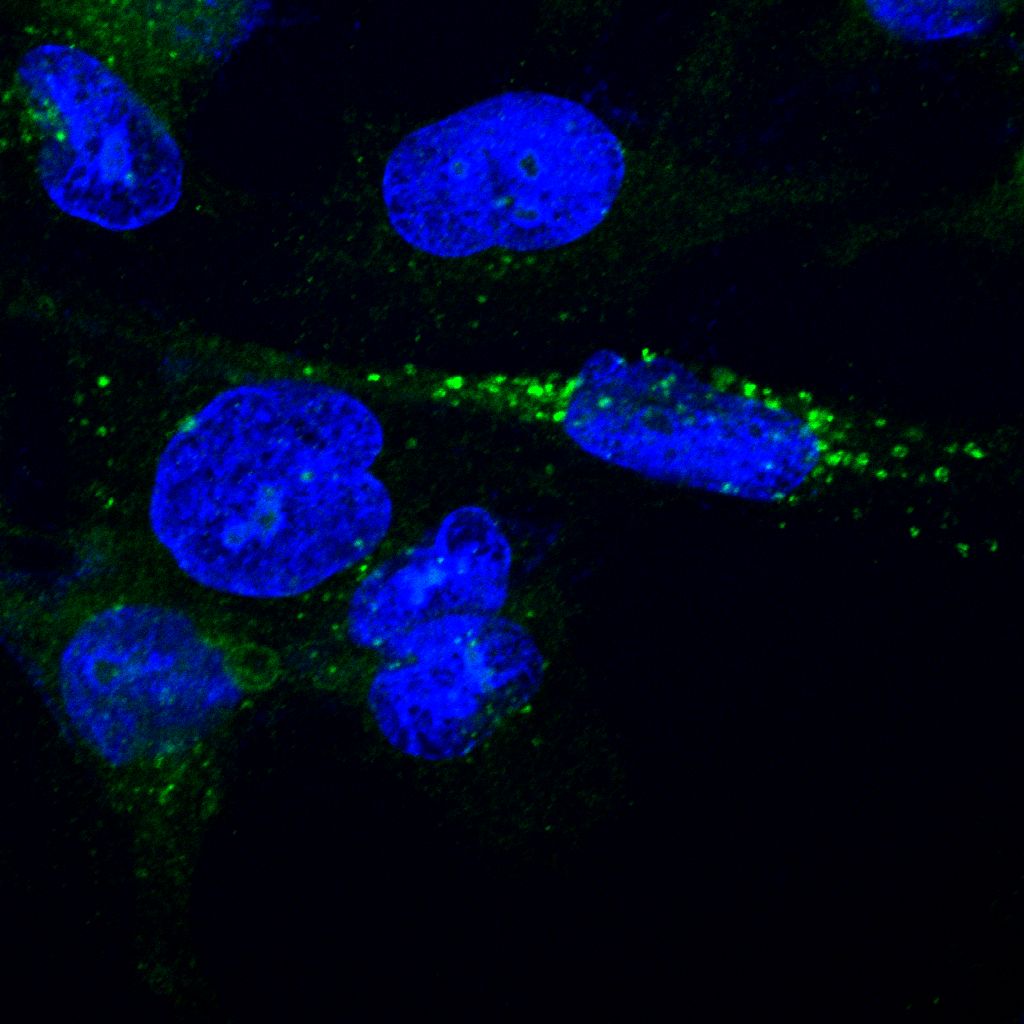

Supplement: Supplementary file 3 — Source data Fig. 1 [file 44318_2024_353_MOESM3_ESM.zip › Figure 1/Figure 1/1I/SKHEP1 siUBE2F-1/HP_RGB.tif]

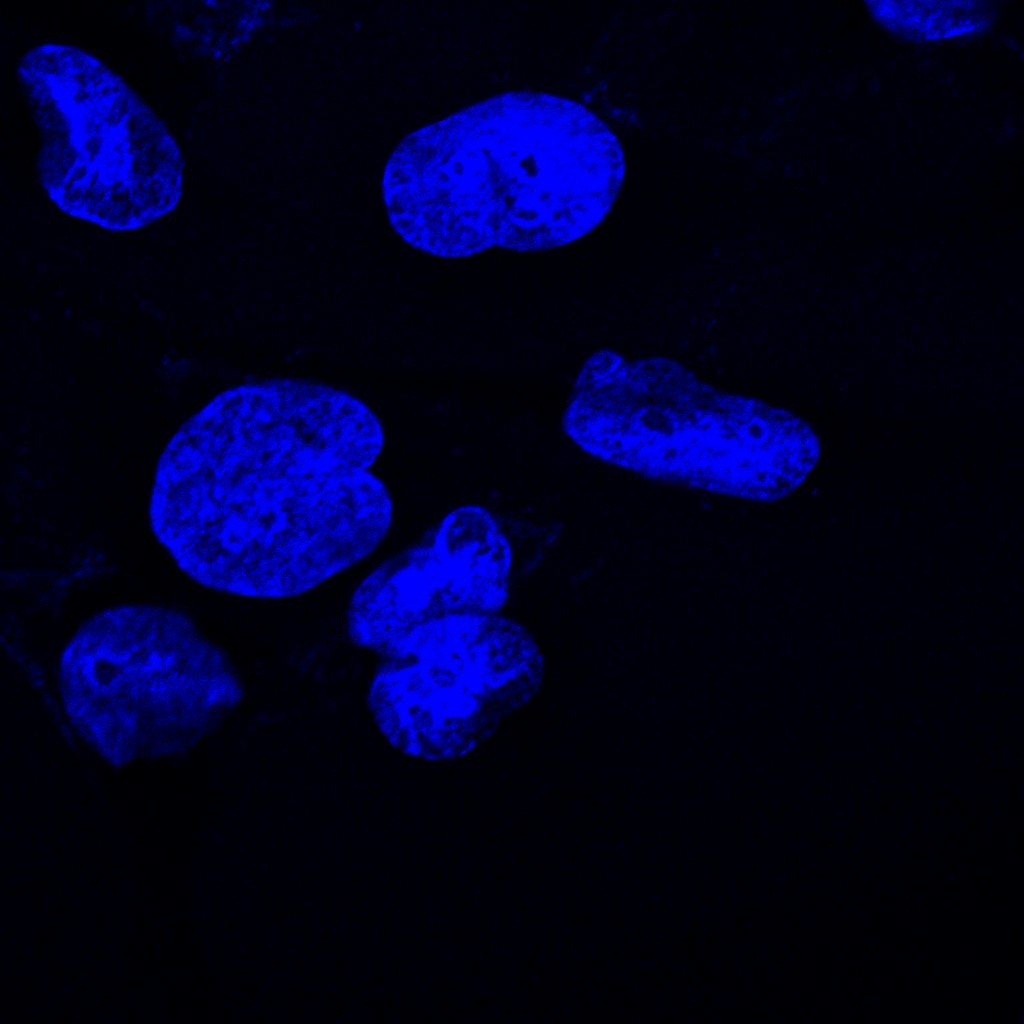

Supplement: Supplementary file 3 — Source data Fig. 1 [file 44318_2024_353_MOESM3_ESM.zip › Figure 1/Figure 1/1I/SKHEP1 siUBE2F-1/HP_RGB_C1.tif]

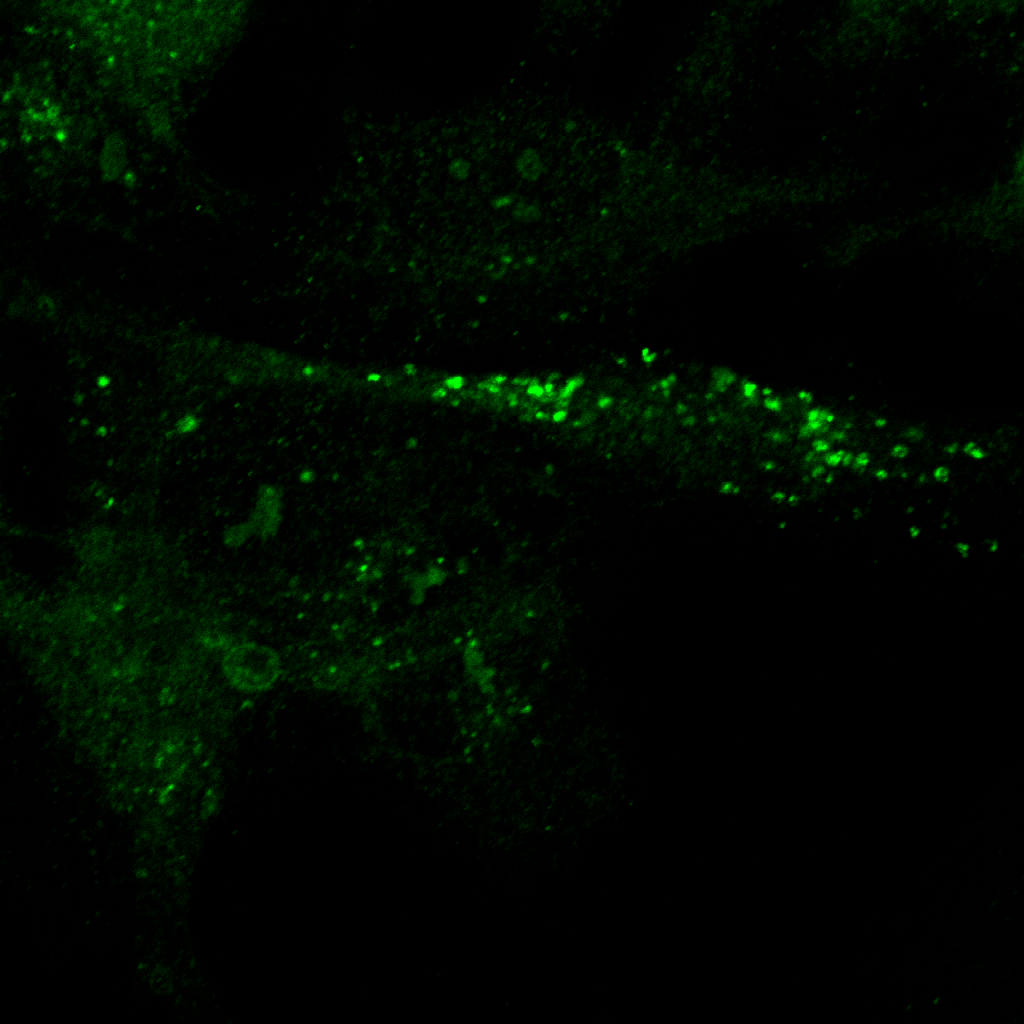

Supplement: Supplementary file 3 — Source data Fig. 1 [file 44318_2024_353_MOESM3_ESM.zip › Figure 1/Figure 1/1I/SKHEP1 siUBE2F-1/HP_RGB_C2.tif]

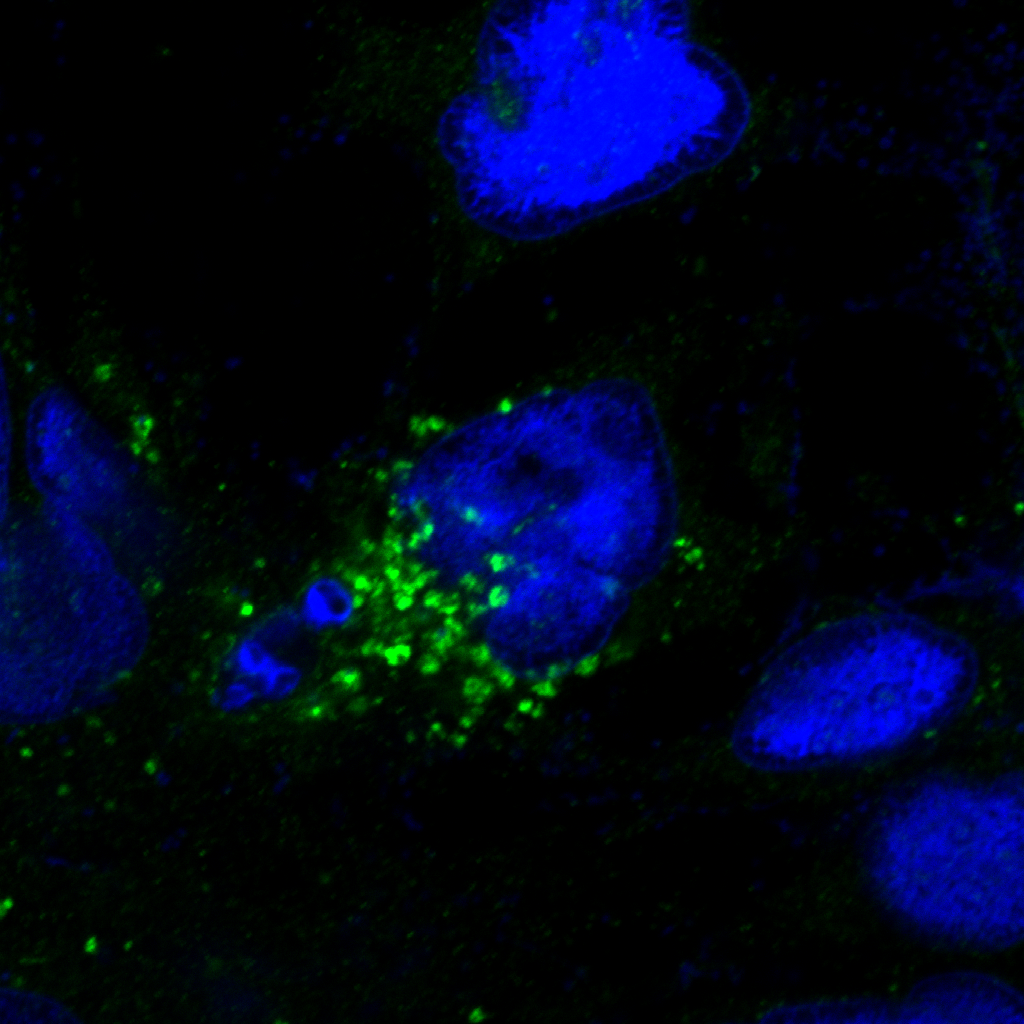

Supplement: Supplementary file 3 — Source data Fig. 1 [file 44318_2024_353_MOESM3_ESM.zip › Figure 1/Figure 1/1I/SKHEP1 siUBE2F-2 60x2.5-2_RGB/HP_HP_SK siUBE2F-5 60x2.5-2_RGB_RGB.tif]

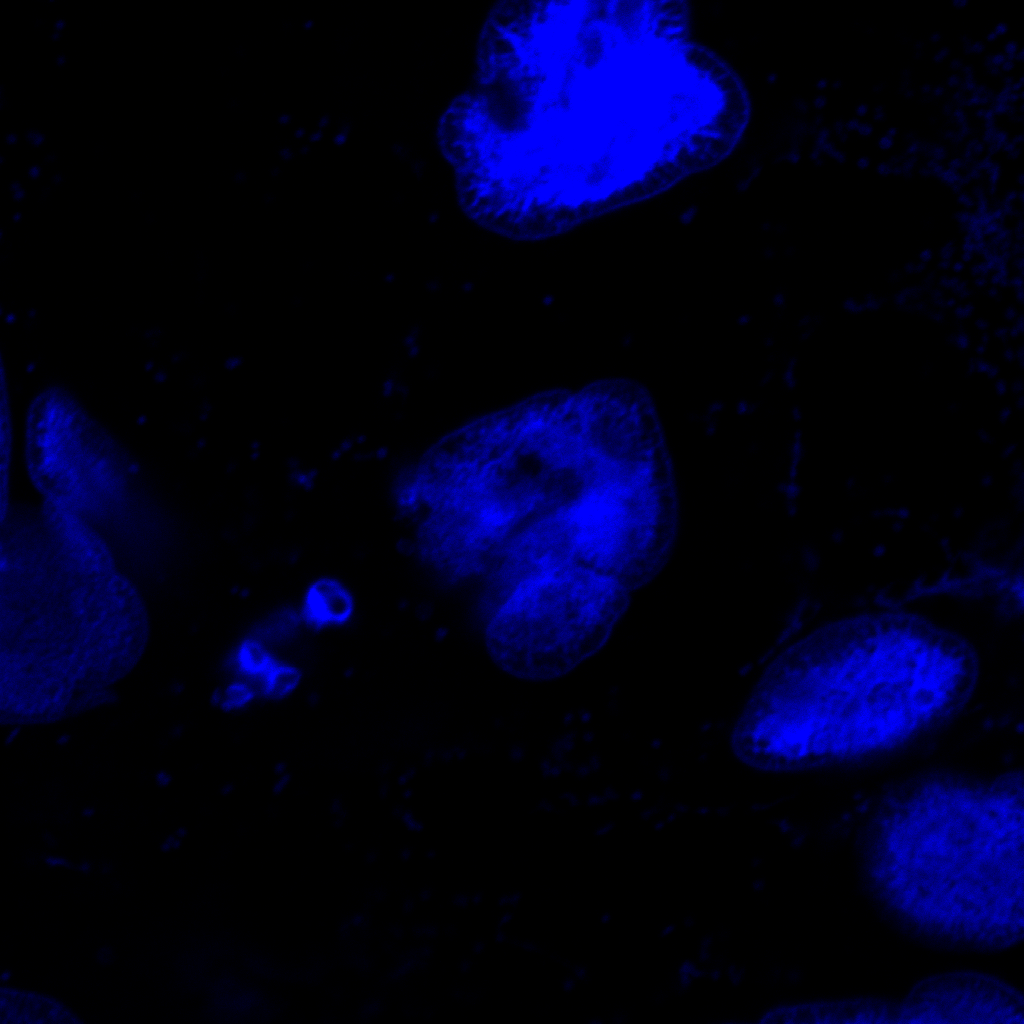

Supplement: Supplementary file 3 — Source data Fig. 1 [file 44318_2024_353_MOESM3_ESM.zip › Figure 1/Figure 1/1I/SKHEP1 siUBE2F-2 60x2.5-2_RGB/HP_HP_SK siUBE2F-5 60x2.5-2_RGB_RGB_C1.tif]

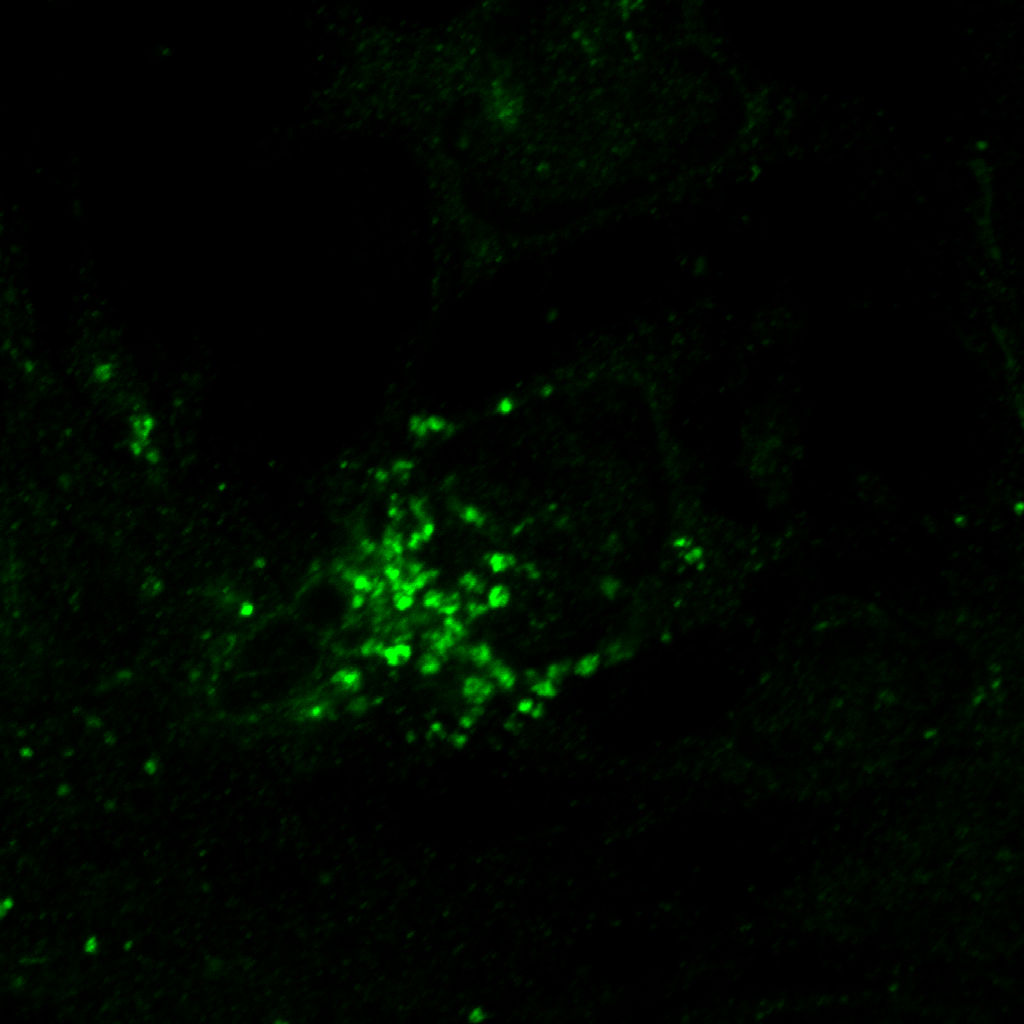

Supplement: Supplementary file 3 — Source data Fig. 1 [file 44318_2024_353_MOESM3_ESM.zip › Figure 1/Figure 1/1I/SKHEP1 siUBE2F-2 60x2.5-2_RGB/HP_HP_SK siUBE2F-5 60x2.5-2_RGB_RGB_C2.tif]

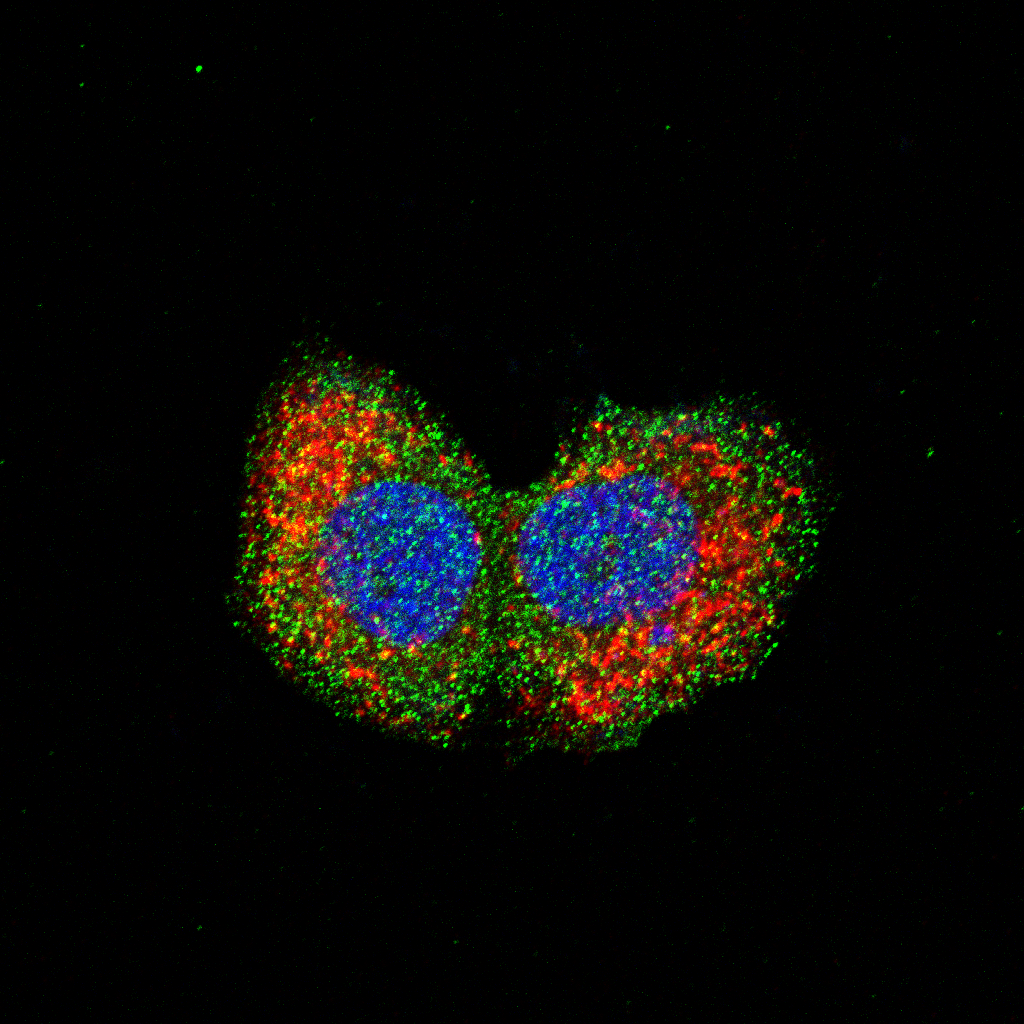

Supplement: Supplementary file 7 — Source data Fig. 5 [file 44318_2024_353_MOESM7_ESM.zip › Figure 5/5C/PLCPRF5 RHEB-K169R HA-R488+M-LAMP2/HP_2023_07_30_RGB.tif]

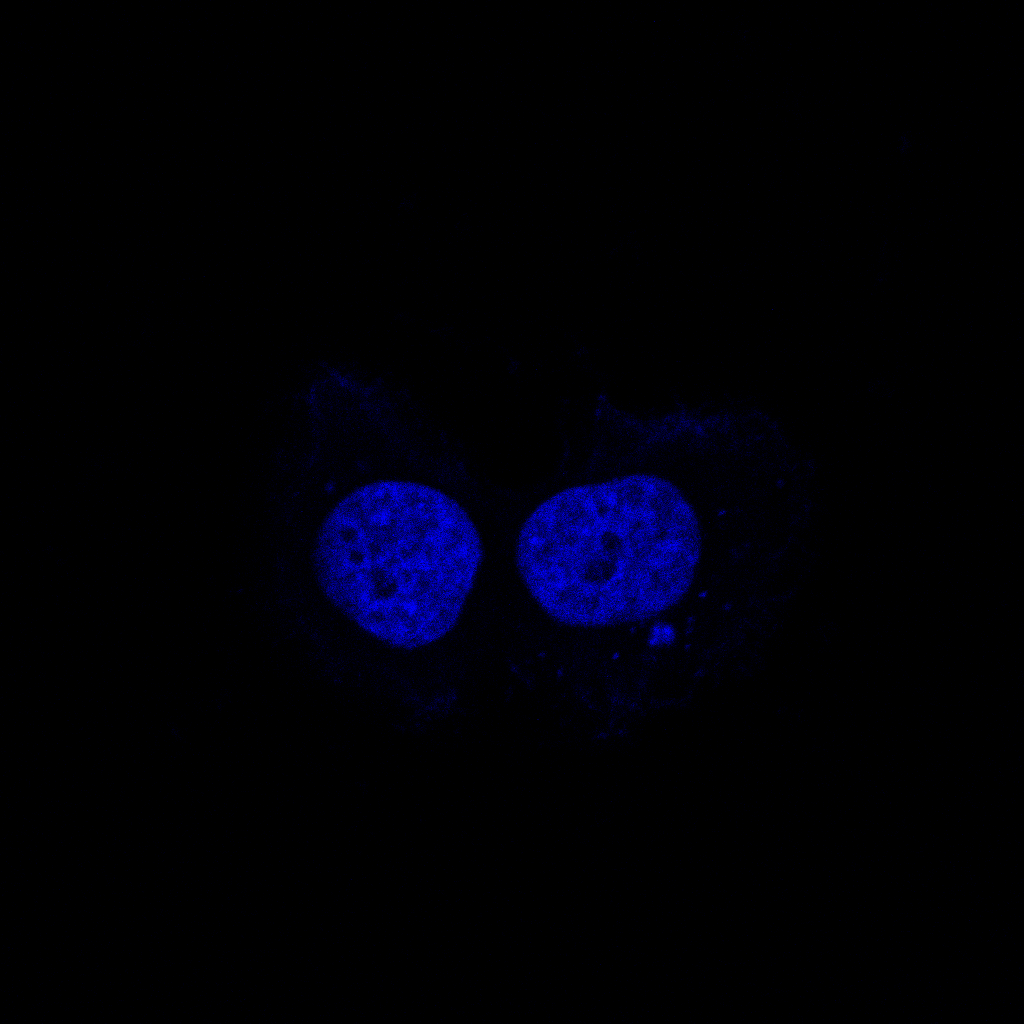

Supplement: Supplementary file 7 — Source data Fig. 5 [file 44318_2024_353_MOESM7_ESM.zip › Figure 5/5C/PLCPRF5 RHEB-K169R HA-R488+M-LAMP2/HP_2023_07_30_RGB_DAPI.tif]

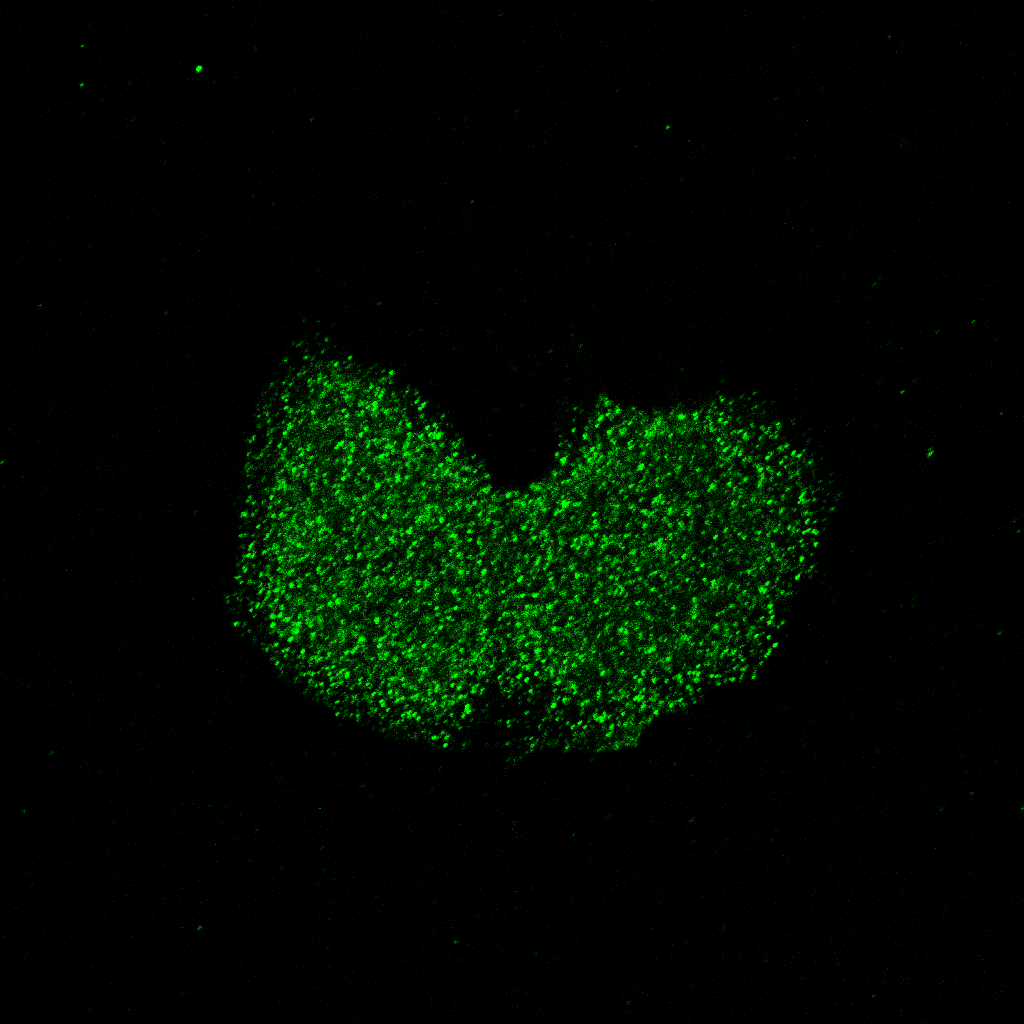

Supplement: Supplementary file 7 — Source data Fig. 5 [file 44318_2024_353_MOESM7_ESM.zip › Figure 5/5C/PLCPRF5 RHEB-K169R HA-R488+M-LAMP2/HP_2023_07_30_RGB_FITC.tif]

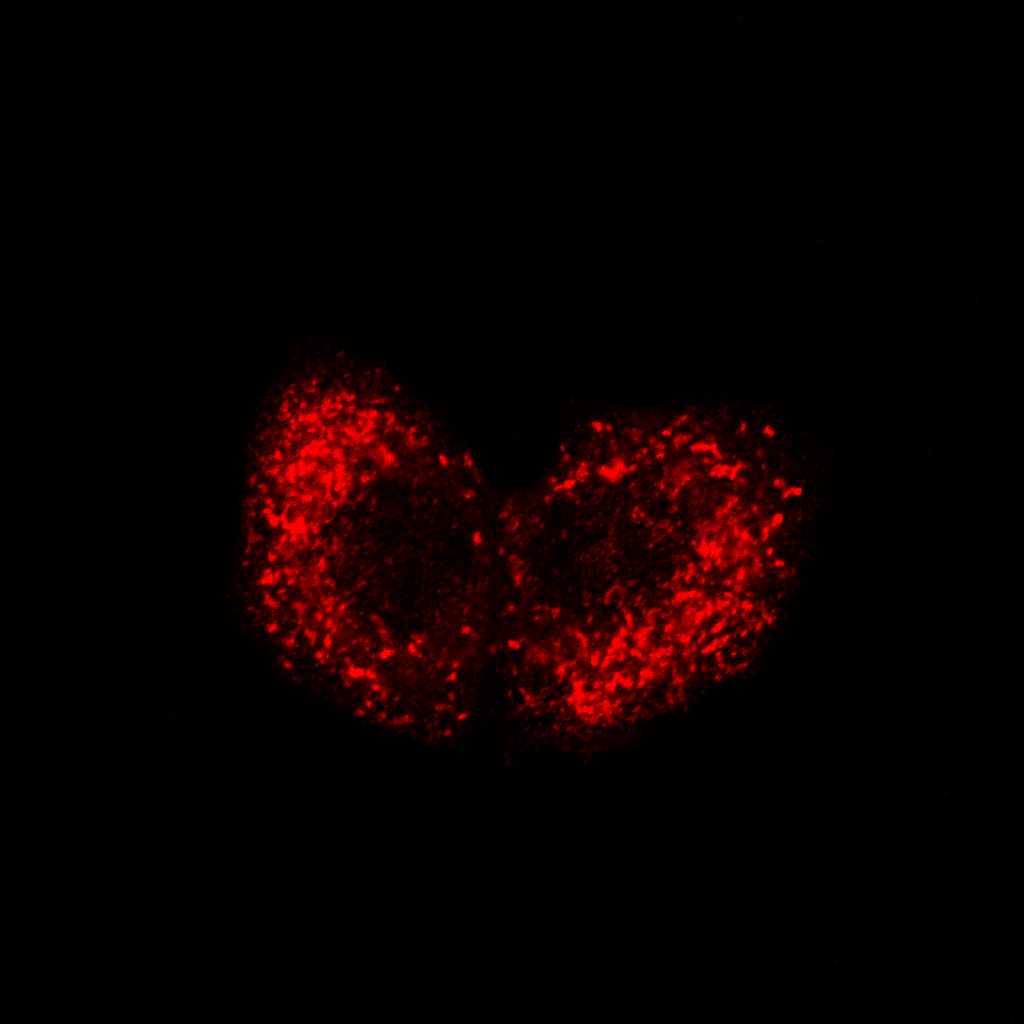

Supplement: Supplementary file 7 — Source data Fig. 5 [file 44318_2024_353_MOESM7_ESM.zip › Figure 5/5C/PLCPRF5 RHEB-K169R HA-R488+M-LAMP2/HP_2023_07_30_RGB_TRITC.tif]

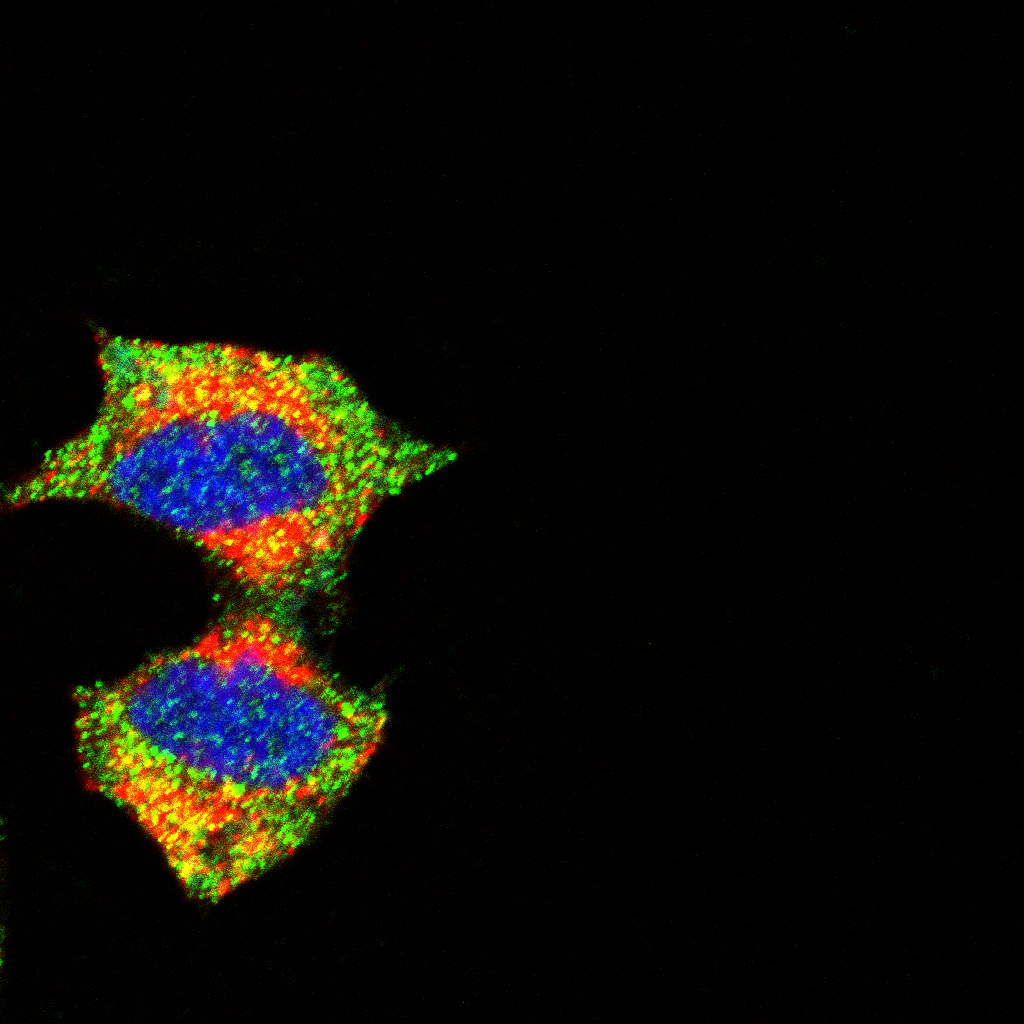

Supplement: Supplementary file 7 — Source data Fig. 5 [file 44318_2024_353_MOESM7_ESM.zip › Figure 5/5C/PLCPRF5 WTRHEB HA-R488+M-LAMP2/HP_2023_07_29_RGB.tif]

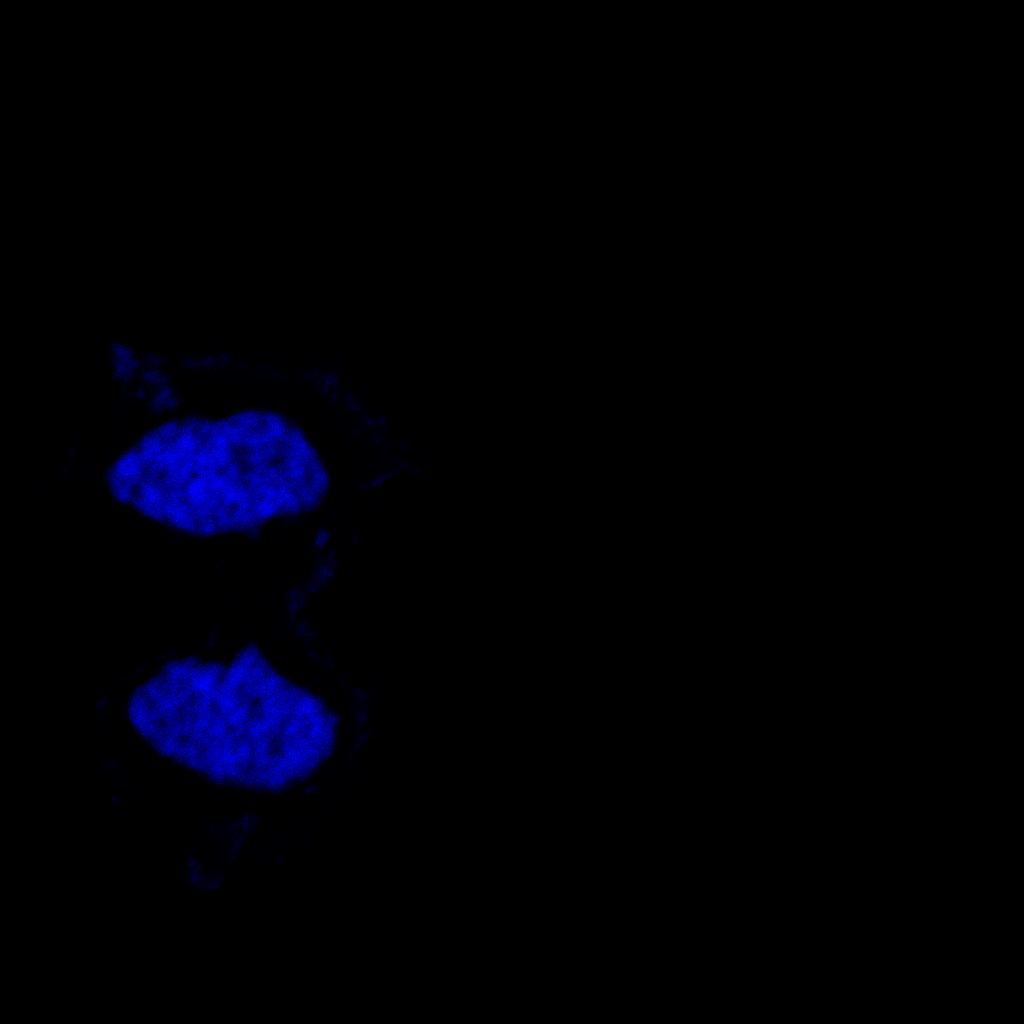

Supplement: Supplementary file 7 — Source data Fig. 5 [file 44318_2024_353_MOESM7_ESM.zip › Figure 5/5C/PLCPRF5 WTRHEB HA-R488+M-LAMP2/HP_2023_07_29_RGB_DAPI.tif]

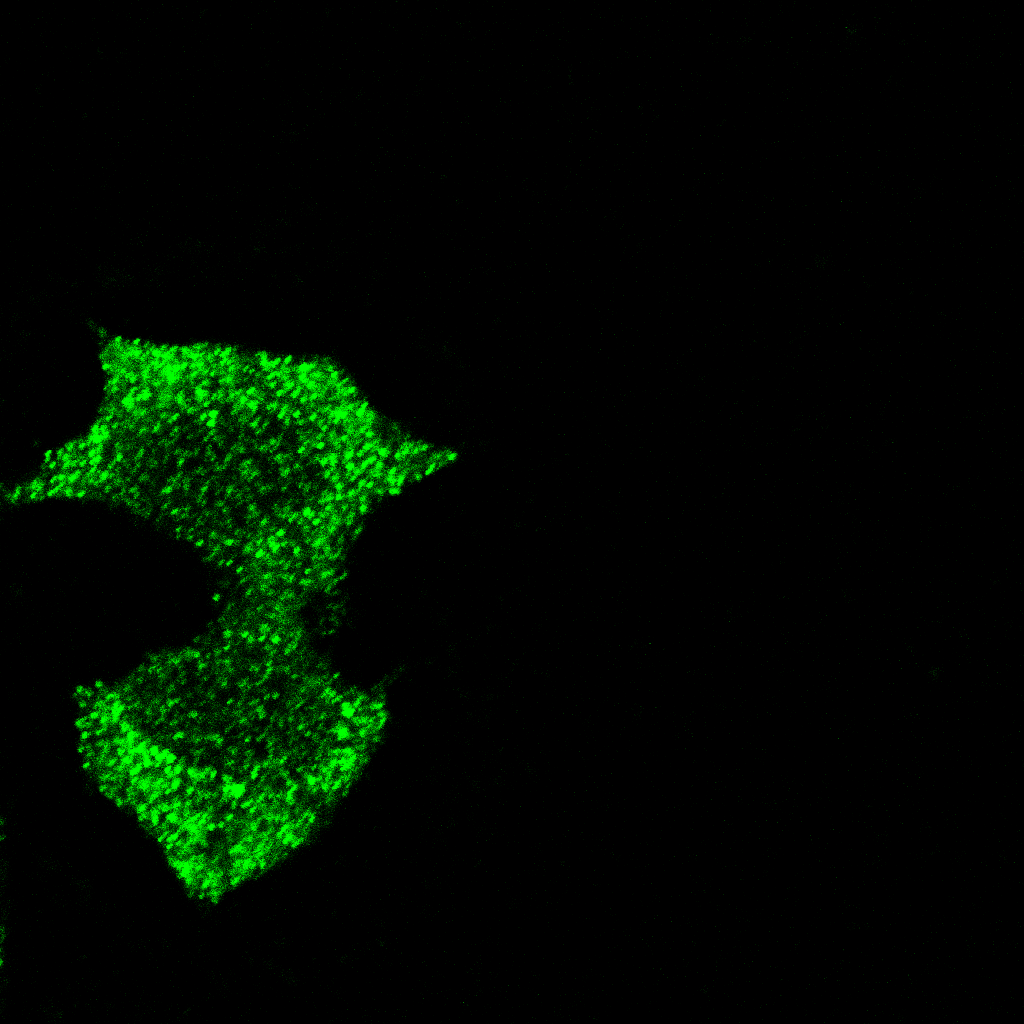

Supplement: Supplementary file 7 — Source data Fig. 5 [file 44318_2024_353_MOESM7_ESM.zip › Figure 5/5C/PLCPRF5 WTRHEB HA-R488+M-LAMP2/HP_2023_07_29_RGB_FITC.tif]

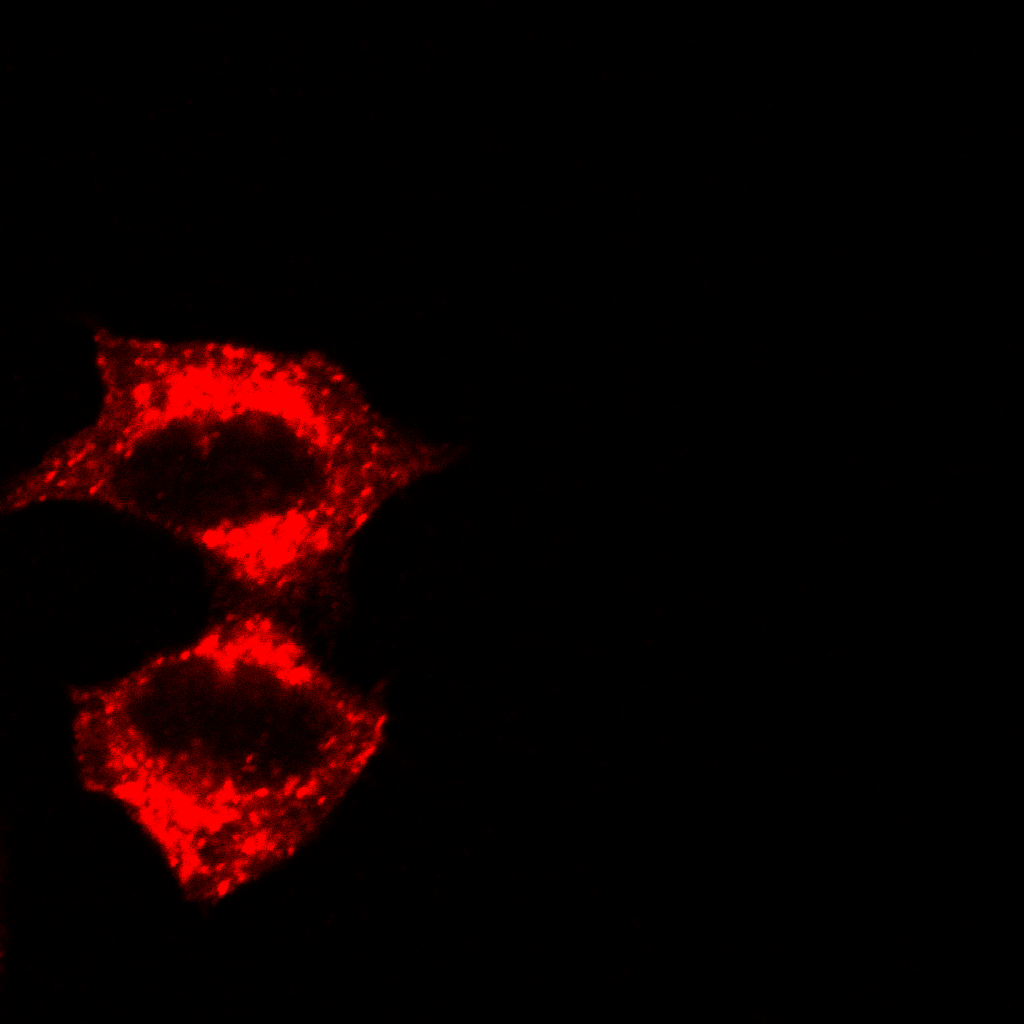

Supplement: Supplementary file 7 — Source data Fig. 5 [file 44318_2024_353_MOESM7_ESM.zip › Figure 5/5C/PLCPRF5 WTRHEB HA-R488+M-LAMP2/HP_2023_07_29_RGB_TRITC.tif]

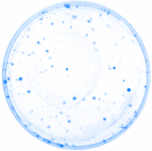

Supplement: Supplementary file 8 — Source data Fig. 6 [file 44318_2024_353_MOESM8_ESM.zip › Figure 6/Figure 6/6B/shGFP+RHEB-K169R.png]

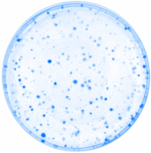

Supplement: Supplementary file 8 — Source data Fig. 6 [file 44318_2024_353_MOESM8_ESM.zip › Figure 6/Figure 6/6B/shGFP+WT-RHEB.png]

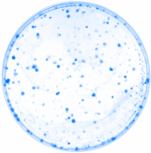

Supplement: Supplementary file 8 — Source data Fig. 6 [file 44318_2024_353_MOESM8_ESM.zip › Figure 6/Figure 6/6B/shGFP+pLVX.png]

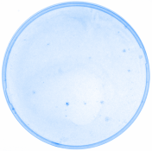

Supplement: Supplementary file 8 — Source data Fig. 6 [file 44318_2024_353_MOESM8_ESM.zip › Figure 6/Figure 6/6B/shSAG+RHEB-K169R.png]

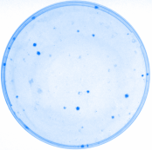

Supplement: Supplementary file 8 — Source data Fig. 6 [file 44318_2024_353_MOESM8_ESM.zip › Figure 6/Figure 6/6B/shSAG+WT-RHEB.png]

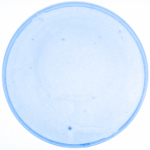

Supplement: Supplementary file 8 — Source data Fig. 6 [file 44318_2024_353_MOESM8_ESM.zip › Figure 6/Figure 6/6B/shSAG+pLVX.png]

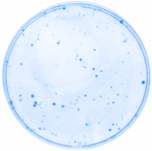

Supplement: Supplementary file 8 — Source data Fig. 6 [file 44318_2024_353_MOESM8_ESM.zip › Figure 6/Figure 6/6B/shUBE2F+RHEB-K169R.png]

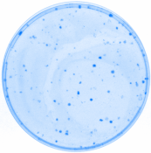

Supplement: Supplementary file 8 — Source data Fig. 6 [file 44318_2024_353_MOESM8_ESM.zip › Figure 6/Figure 6/6B/shUBE2F+WT-RHEB.png]

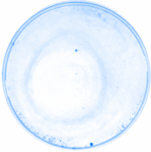

Supplement: Supplementary file 8 — Source data Fig. 6 [file 44318_2024_353_MOESM8_ESM.zip › Figure 6/Figure 6/6B/shUBE2F+pLVX.png]

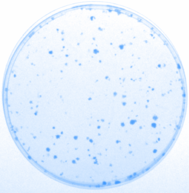

Supplement: Supplementary file 8 — Source data Fig. 6 [file 44318_2024_353_MOESM8_ESM.zip › Figure 6/Figure 6/6E/FLAG-SAG+DMSO.png]

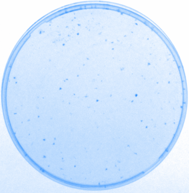

Supplement: Supplementary file 8 — Source data Fig. 6 [file 44318_2024_353_MOESM8_ESM.zip › Figure 6/Figure 6/6E/FLAG-SAG+Rapamycin.png]

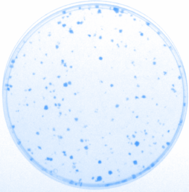

Supplement: Supplementary file 8 — Source data Fig. 6 [file 44318_2024_353_MOESM8_ESM.zip › Figure 6/Figure 6/6E/FLAG-UBE2F+DMSO.png]

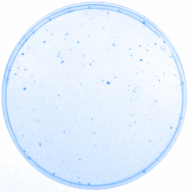

Supplement: Supplementary file 8 — Source data Fig. 6 [file 44318_2024_353_MOESM8_ESM.zip › Figure 6/Figure 6/6E/FLAG-UBE2F+Rapamycin.png]

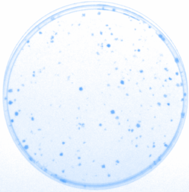

Supplement: Supplementary file 8 — Source data Fig. 6 [file 44318_2024_353_MOESM8_ESM.zip › Figure 6/Figure 6/6E/pLVX+DMSO.png]

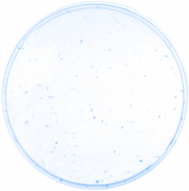

Supplement: Supplementary file 8 — Source data Fig. 6 [file 44318_2024_353_MOESM8_ESM.zip › Figure 6/Figure 6/6E/pLVX+Rapamycin.png]

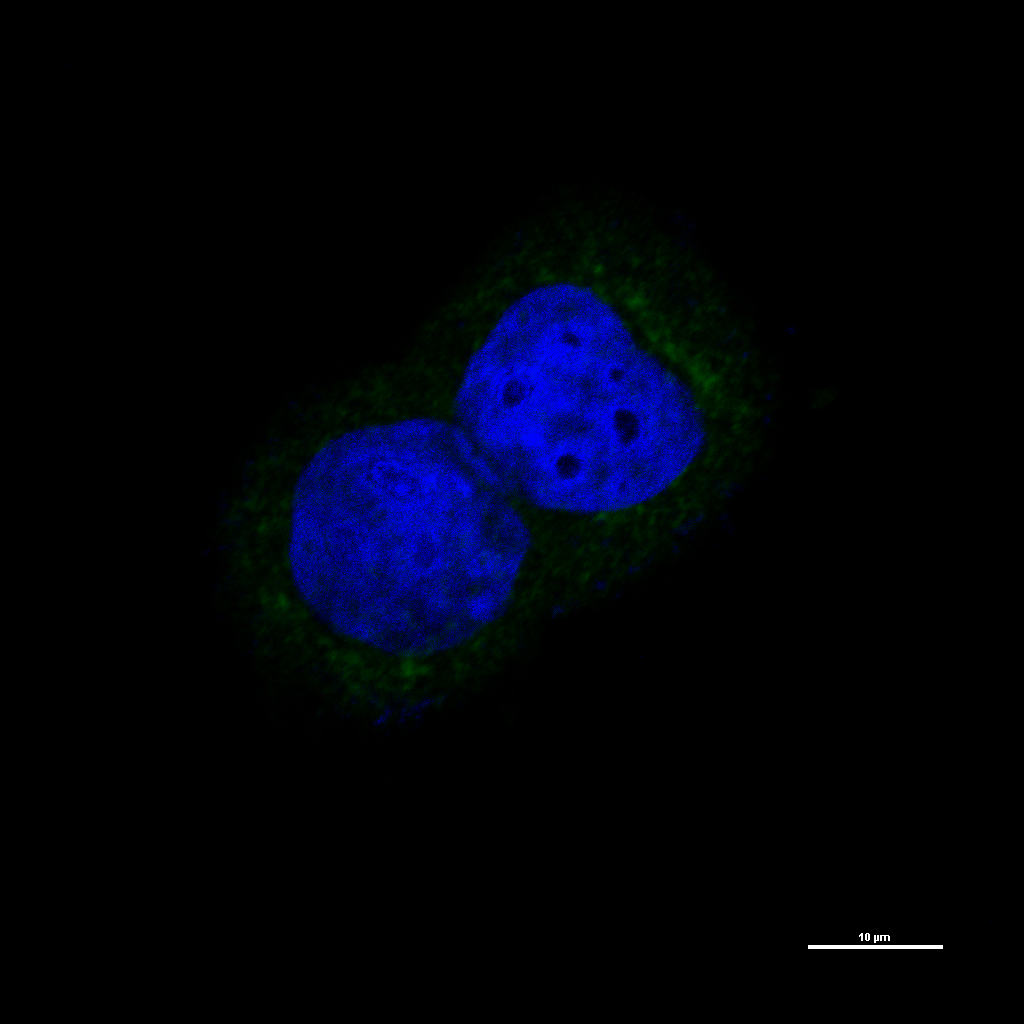

Supplement: Supplementary file 8 — Source data Fig. 6 [file 44318_2024_353_MOESM8_ESM.zip › Figure 6/Figure 6/6K/PLCPRF5 siCtrl+RHEB-DM/HP_RGB.tif]

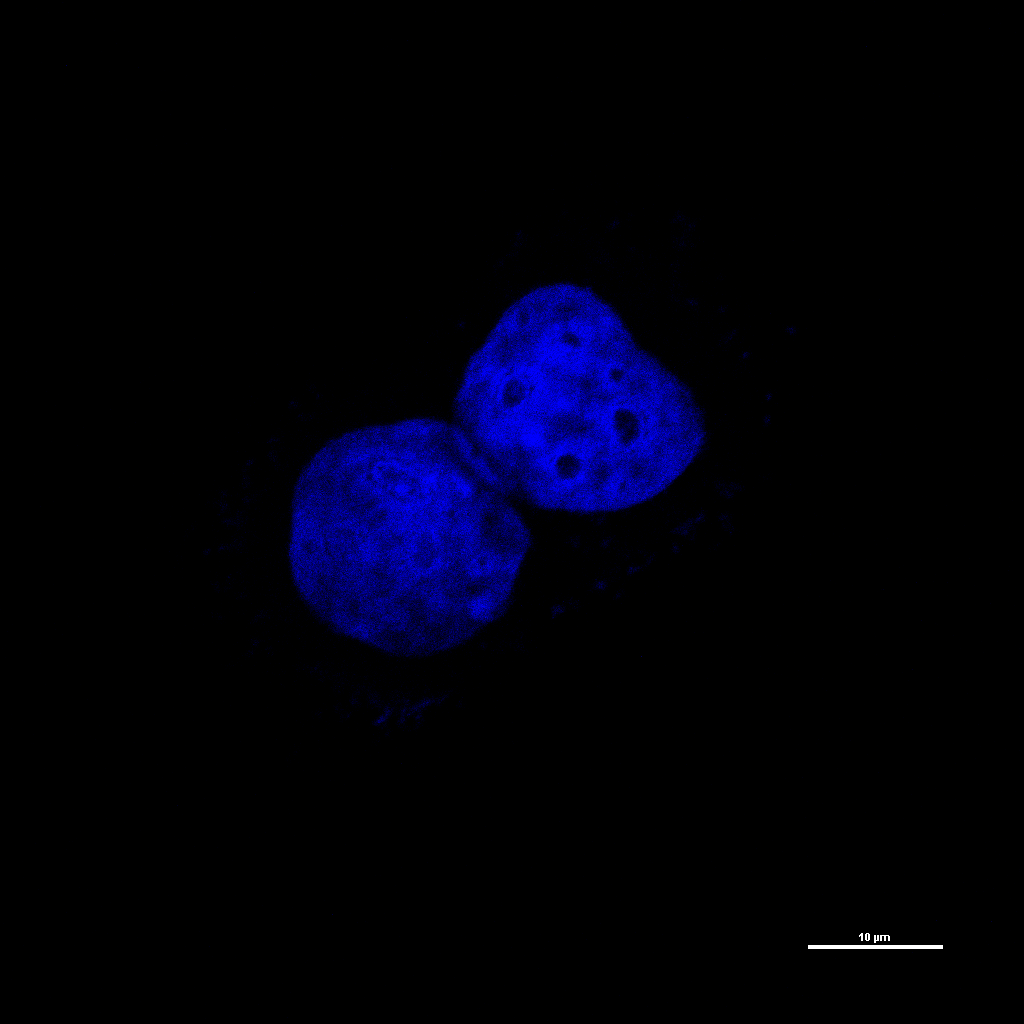

Supplement: Supplementary file 8 — Source data Fig. 6 [file 44318_2024_353_MOESM8_ESM.zip › Figure 6/Figure 6/6K/PLCPRF5 siCtrl+RHEB-DM/HP_RGB_DAPI.tif]

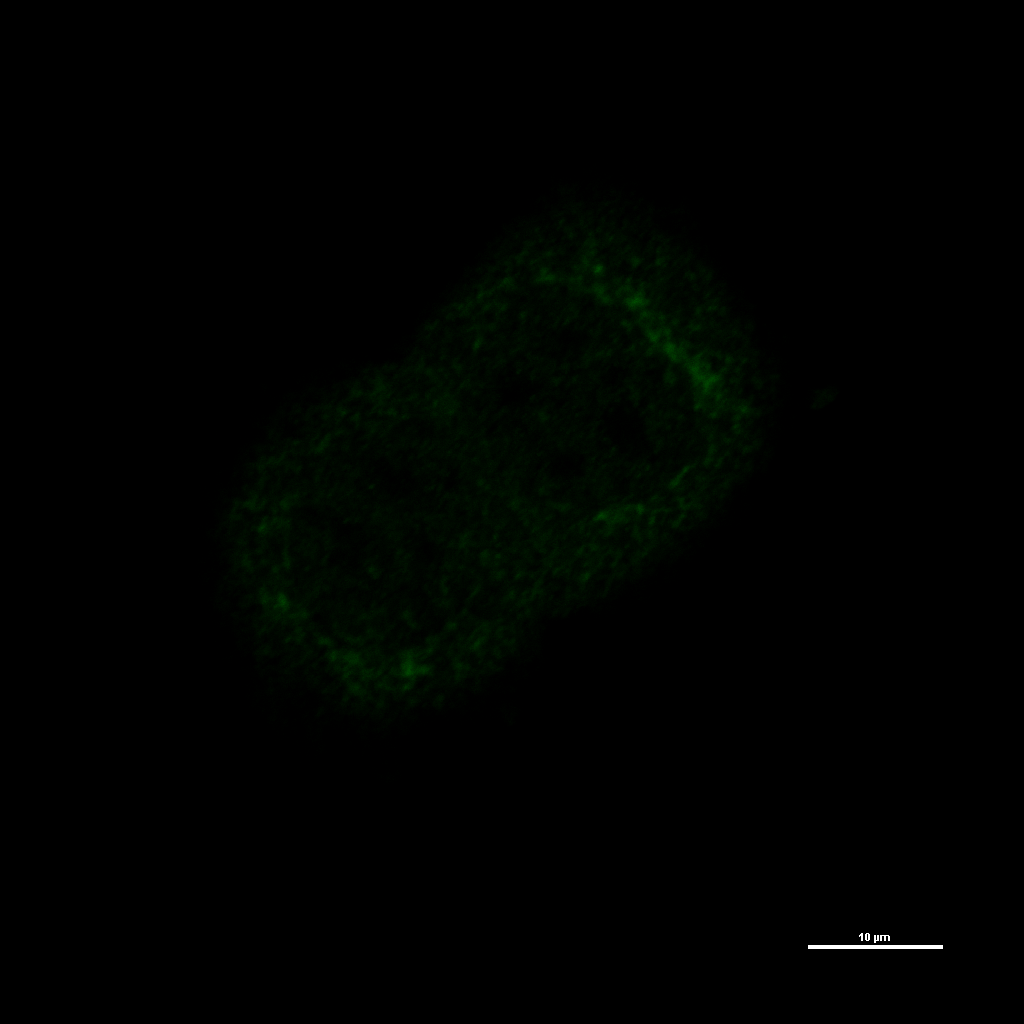

Supplement: Supplementary file 8 — Source data Fig. 6 [file 44318_2024_353_MOESM8_ESM.zip › Figure 6/Figure 6/6K/PLCPRF5 siCtrl+RHEB-DM/HP_RGB_FITC.tif]

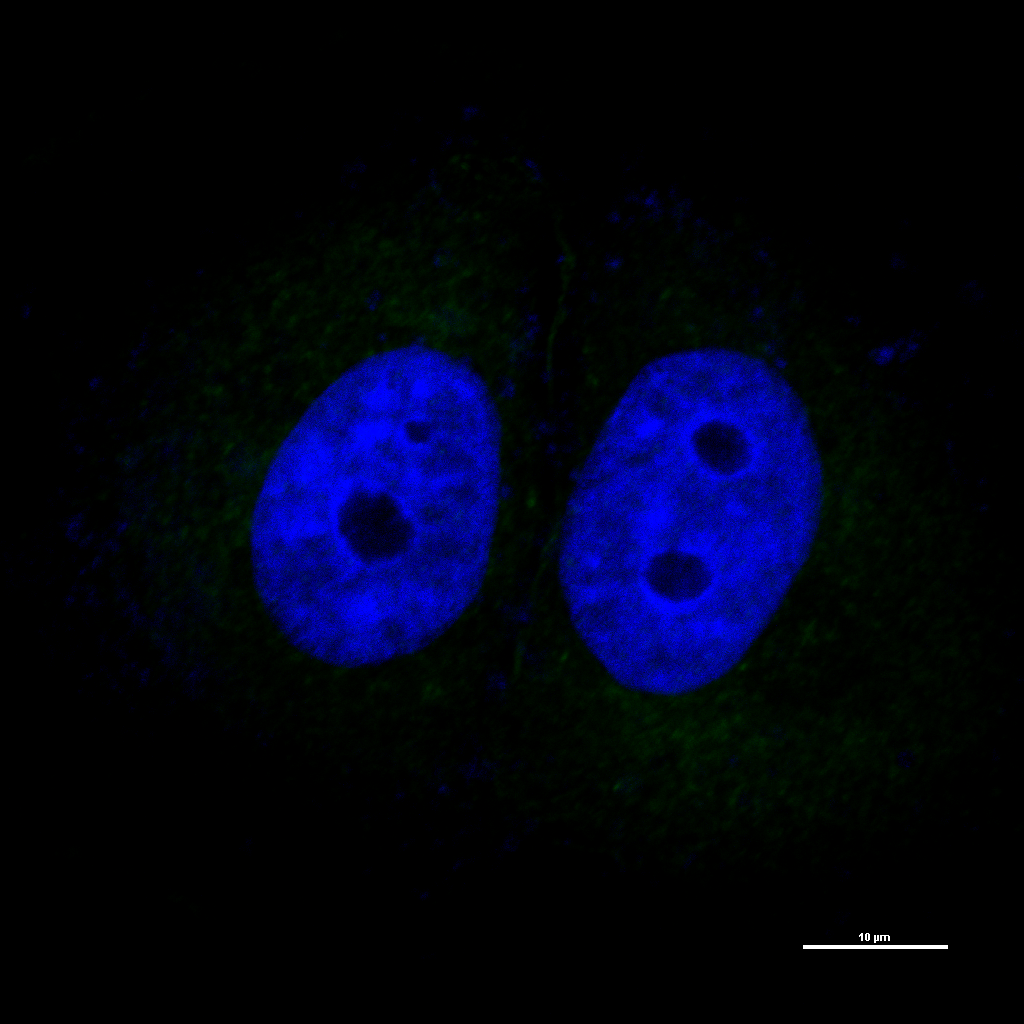

Supplement: Supplementary file 8 — Source data Fig. 6 [file 44318_2024_353_MOESM8_ESM.zip › Figure 6/Figure 6/6K/PLCPRF5 siCtrl+RHEB-Q64L/HP_RGB.tif]

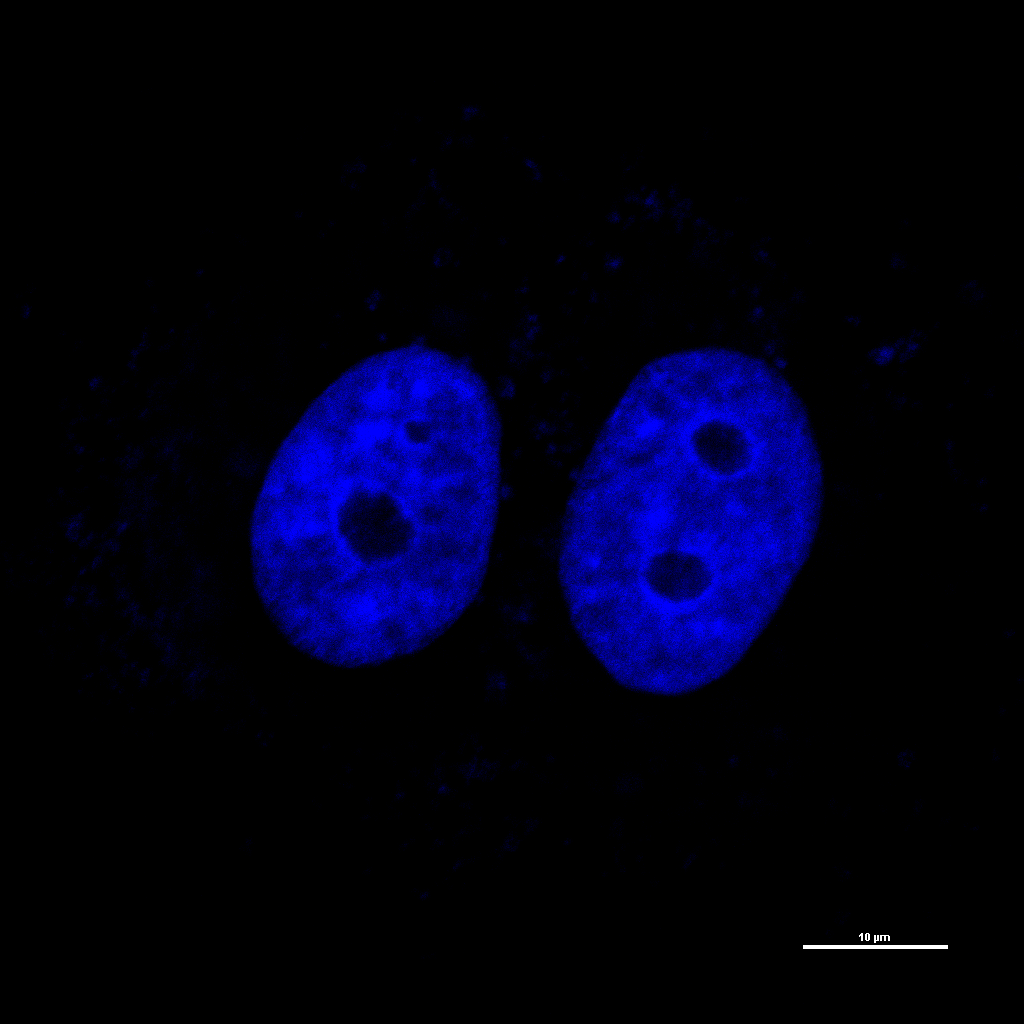

Supplement: Supplementary file 8 — Source data Fig. 6 [file 44318_2024_353_MOESM8_ESM.zip › Figure 6/Figure 6/6K/PLCPRF5 siCtrl+RHEB-Q64L/HP_RGB_DAPI.tif]

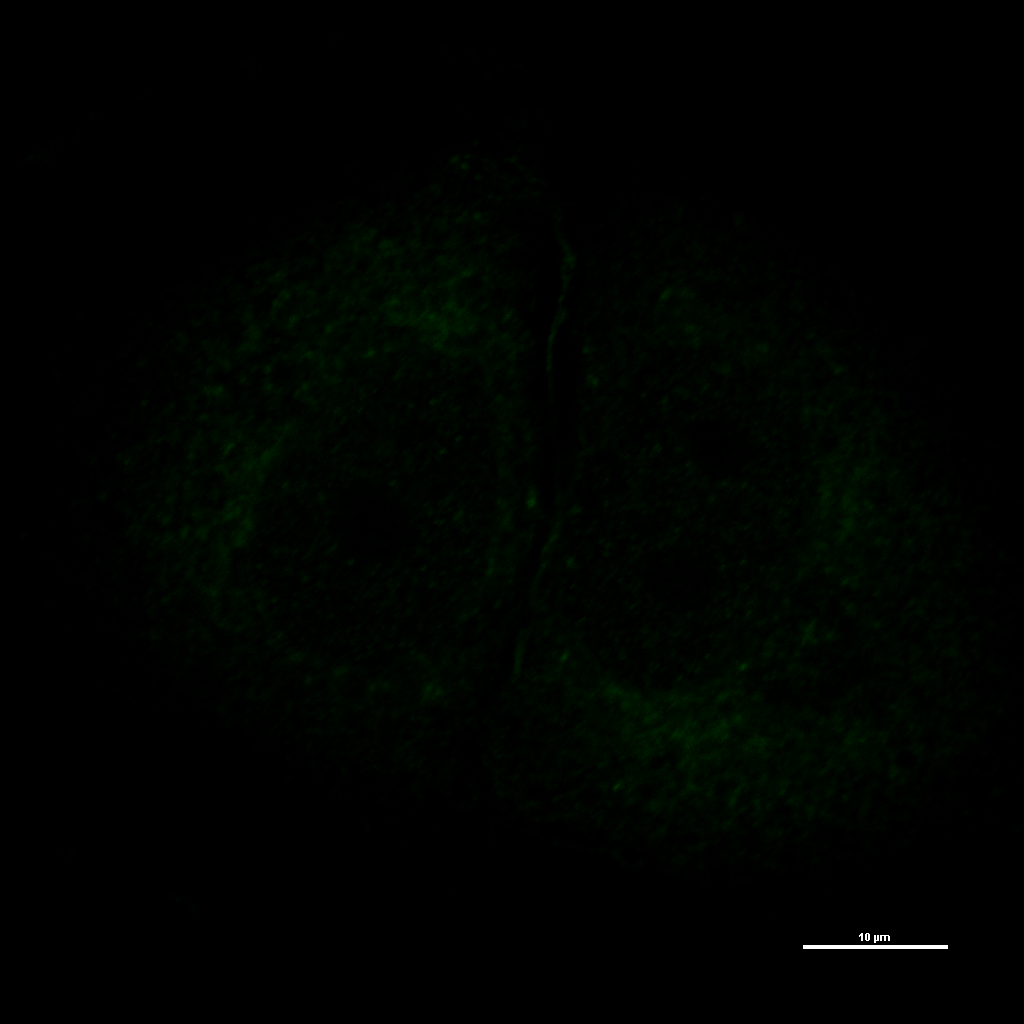

Supplement: Supplementary file 8 — Source data Fig. 6 [file 44318_2024_353_MOESM8_ESM.zip › Figure 6/Figure 6/6K/PLCPRF5 siCtrl+RHEB-Q64L/HP_RGB_FITC.tif]

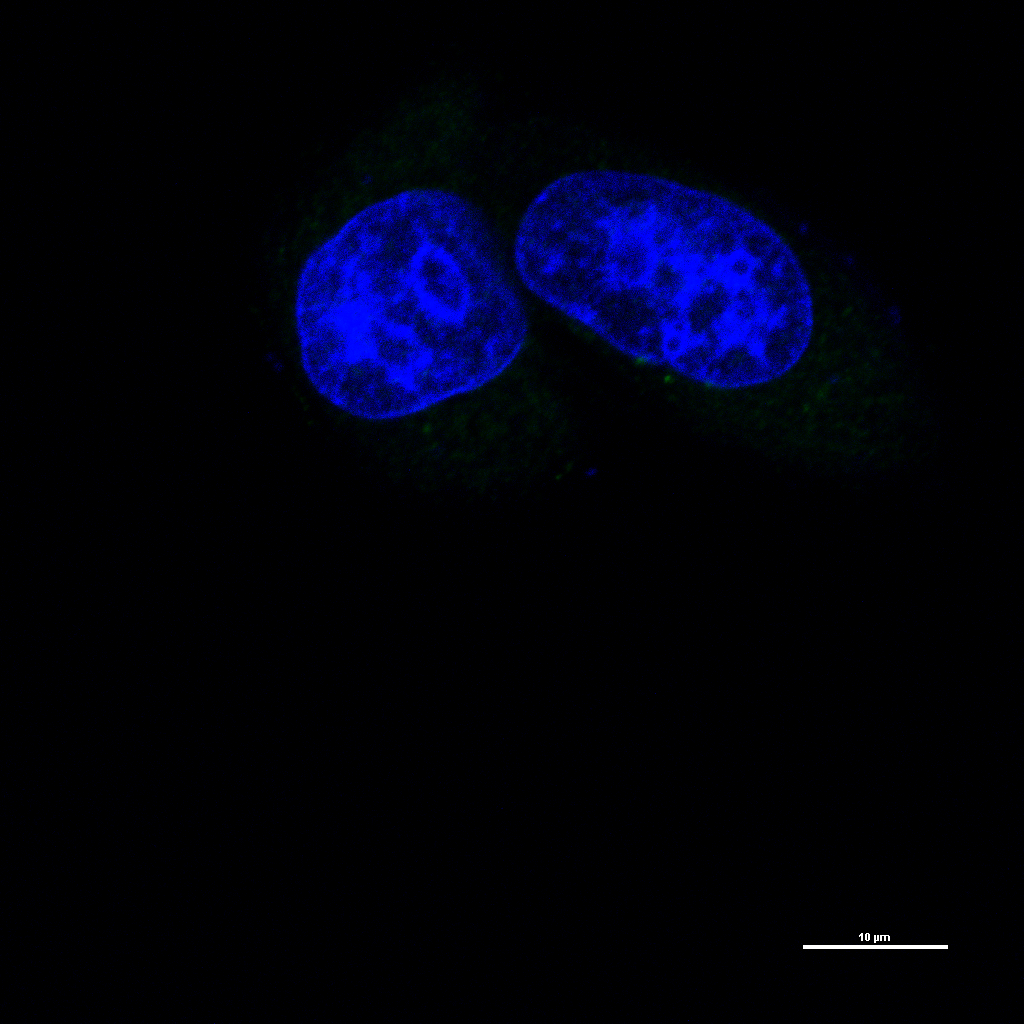

Supplement: Supplementary file 8 — Source data Fig. 6 [file 44318_2024_353_MOESM8_ESM.zip › Figure 6/Figure 6/6K/PLCPRF5 siCtrl+Vector/HP_RGB.tif]

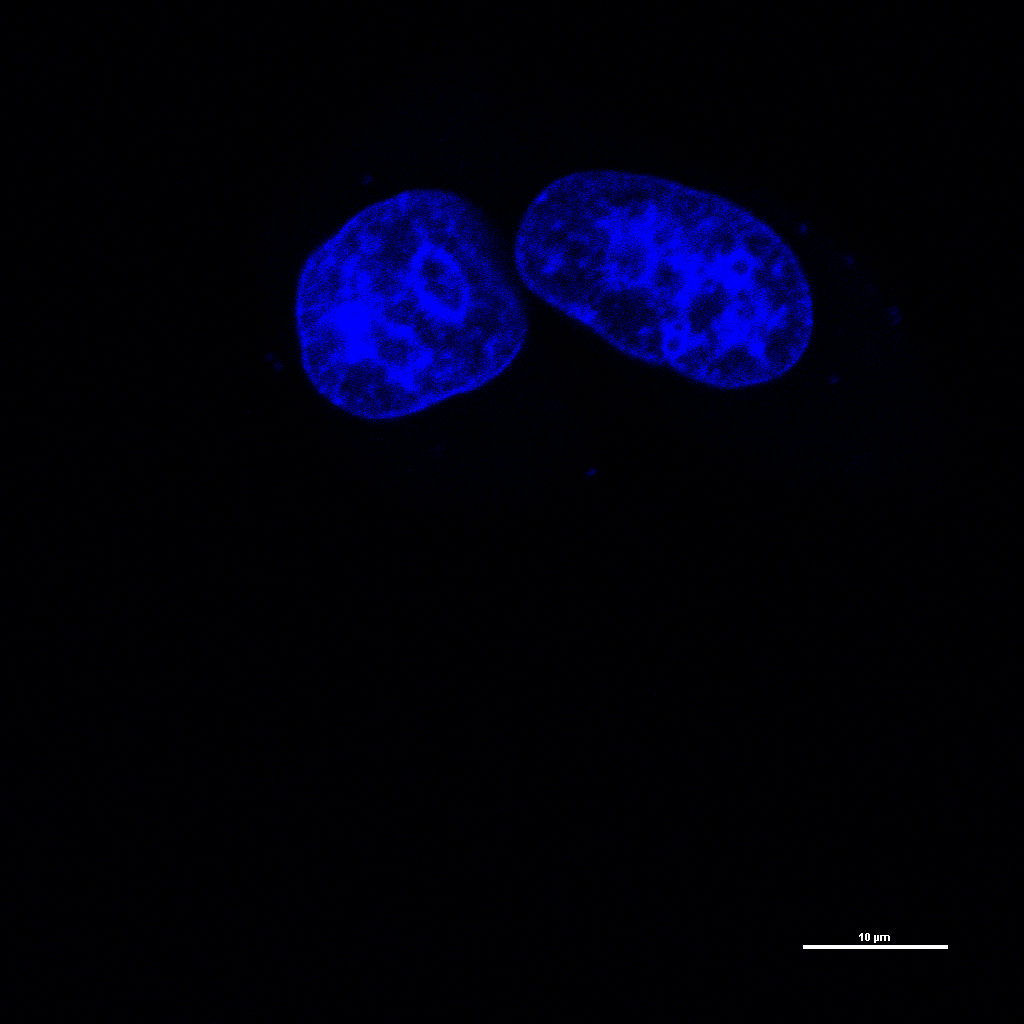

Supplement: Supplementary file 8 — Source data Fig. 6 [file 44318_2024_353_MOESM8_ESM.zip › Figure 6/Figure 6/6K/PLCPRF5 siCtrl+Vector/HP_RGB_DAPI.tif]

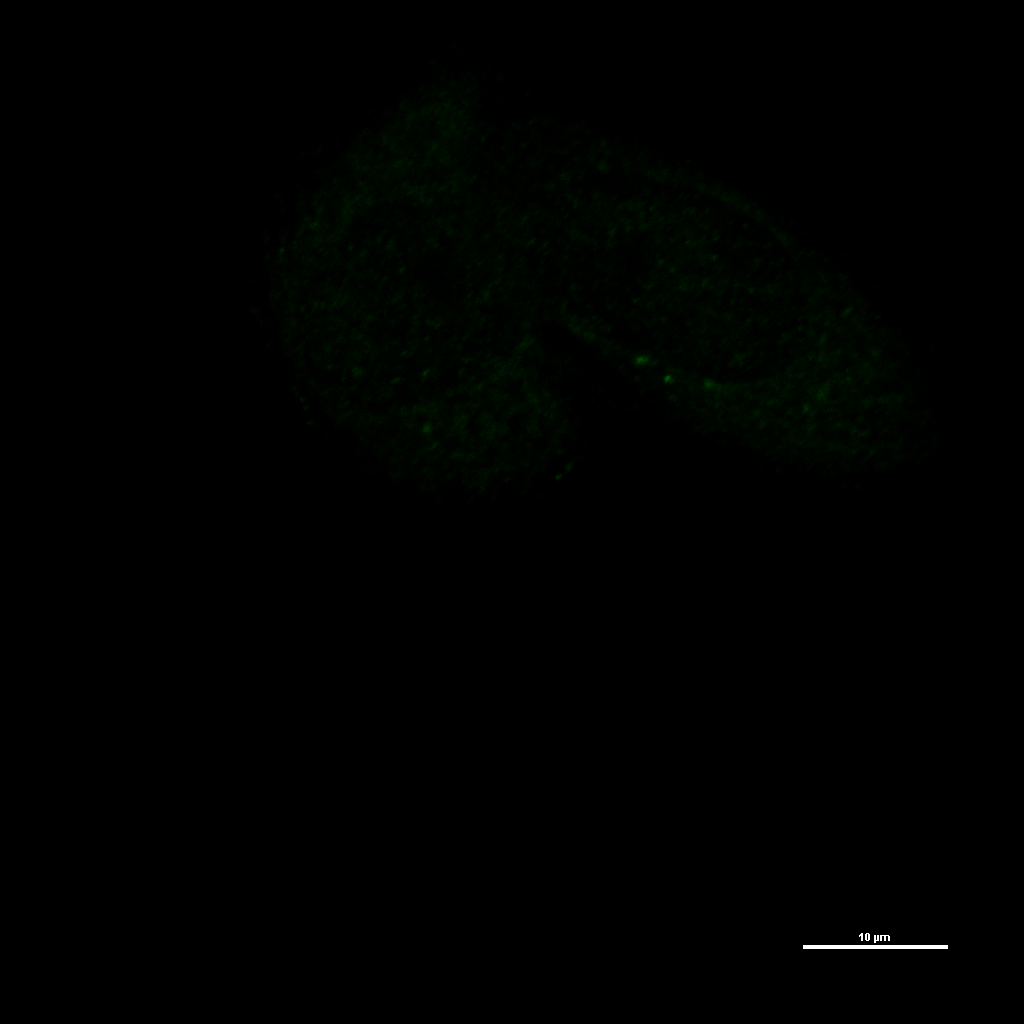

Supplement: Supplementary file 8 — Source data Fig. 6 [file 44318_2024_353_MOESM8_ESM.zip › Figure 6/Figure 6/6K/PLCPRF5 siCtrl+Vector/HP_RGB_FITC.tif]

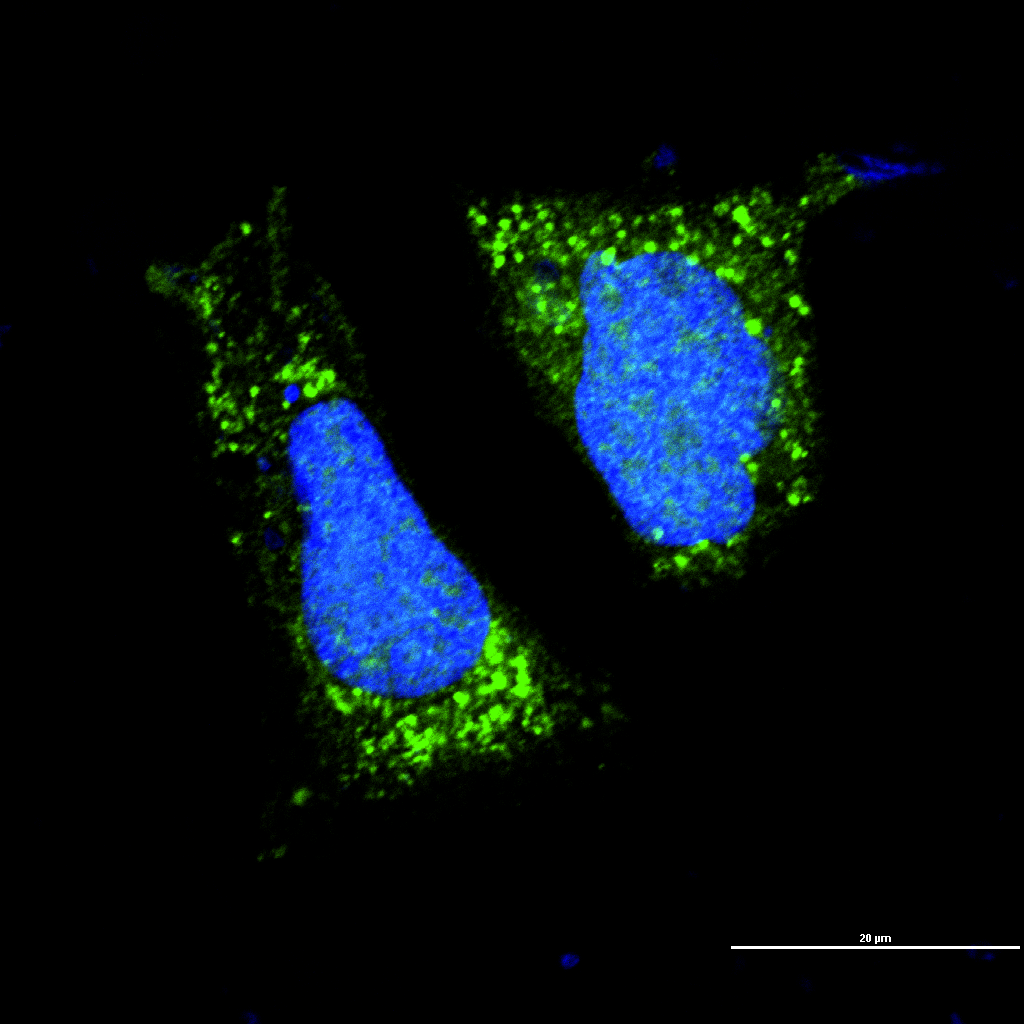

Supplement: Supplementary file 8 — Source data Fig. 6 [file 44318_2024_353_MOESM8_ESM.zip › Figure 6/Figure 6/6K/PLCPRF5 siSAG+RHEB-DM/HP_2024_02_25_RGB.tif]

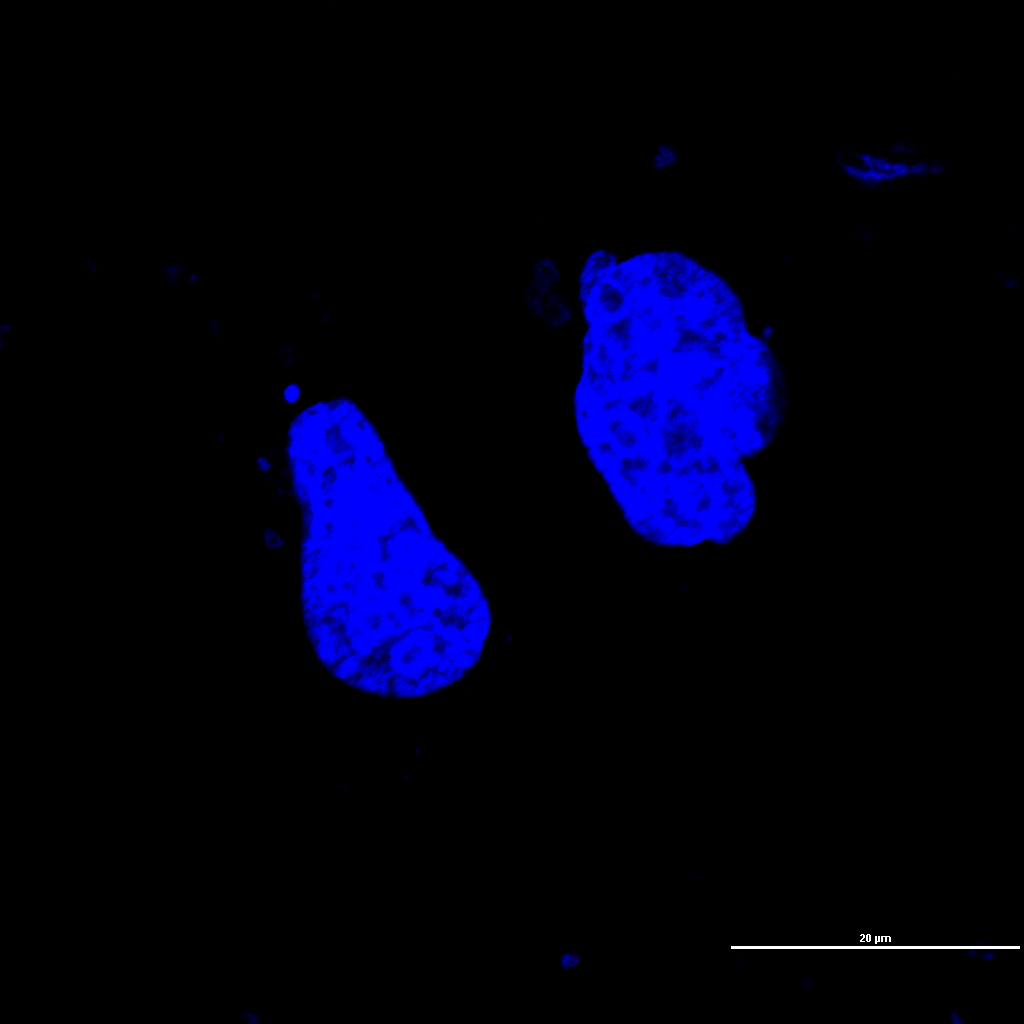

Supplement: Supplementary file 8 — Source data Fig. 6 [file 44318_2024_353_MOESM8_ESM.zip › Figure 6/Figure 6/6K/PLCPRF5 siSAG+RHEB-DM/HP_2024_02_25_RGB_DAPI.tif]

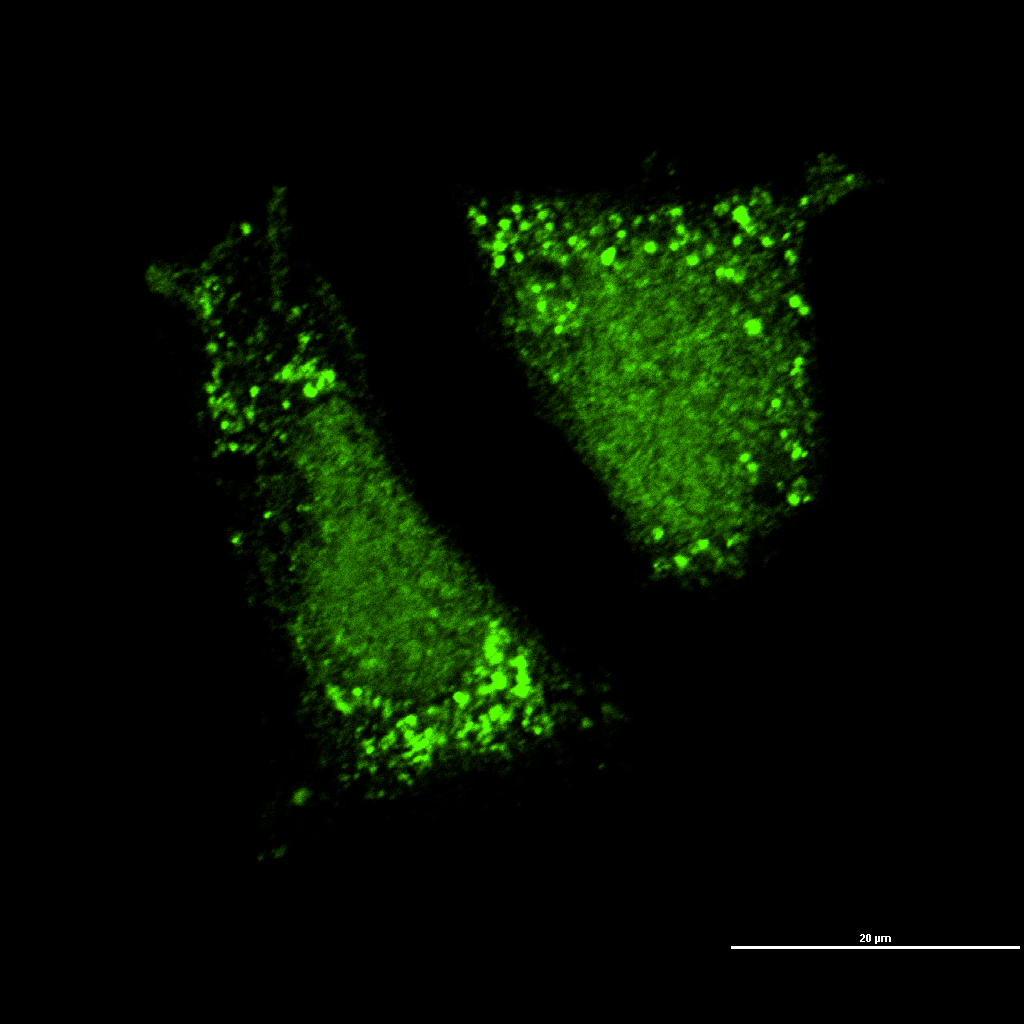

Supplement: Supplementary file 8 — Source data Fig. 6 [file 44318_2024_353_MOESM8_ESM.zip › Figure 6/Figure 6/6K/PLCPRF5 siSAG+RHEB-DM/HP_2024_02_25_RGB_FITC.tif]

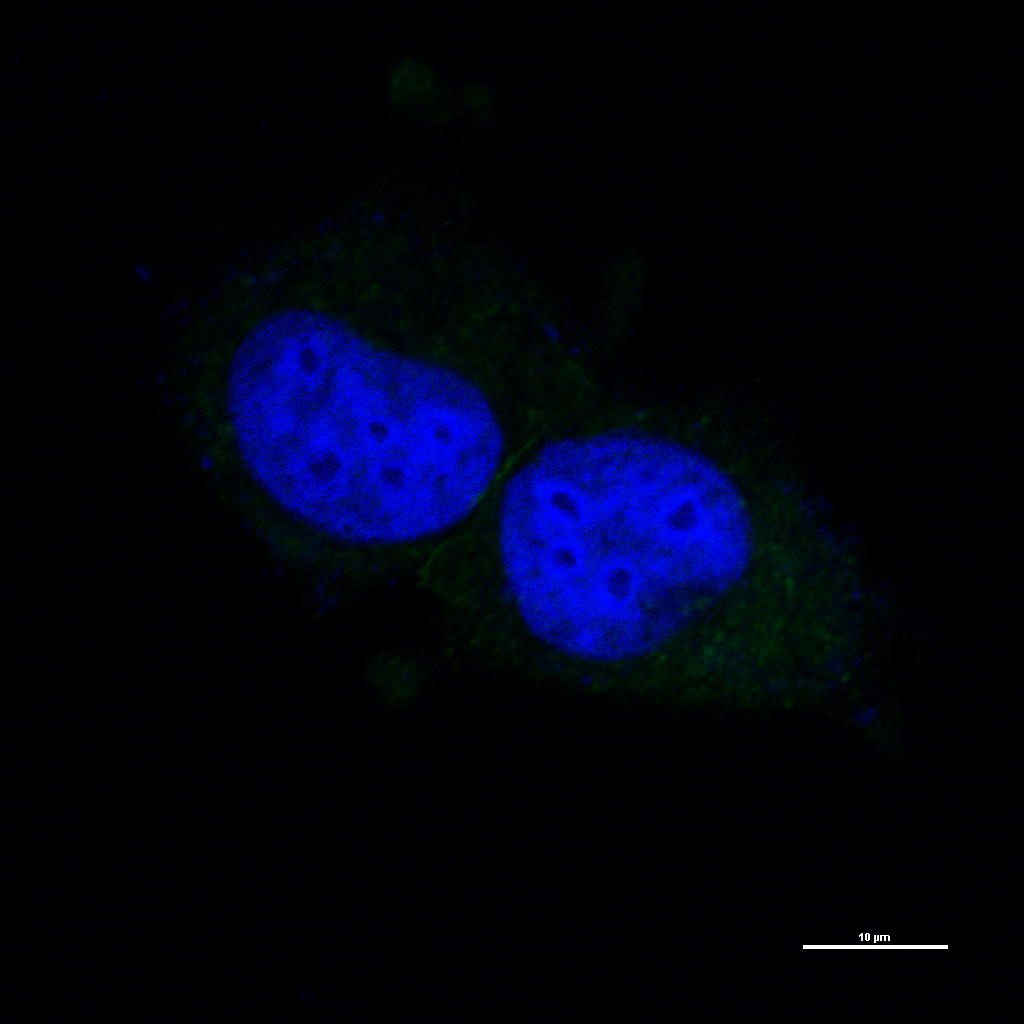

Supplement: Supplementary file 8 — Source data Fig. 6 [file 44318_2024_353_MOESM8_ESM.zip › Figure 6/Figure 6/6K/PLCPRF5 siSAG+RHEB-Q64L/HP_RGB.tif]

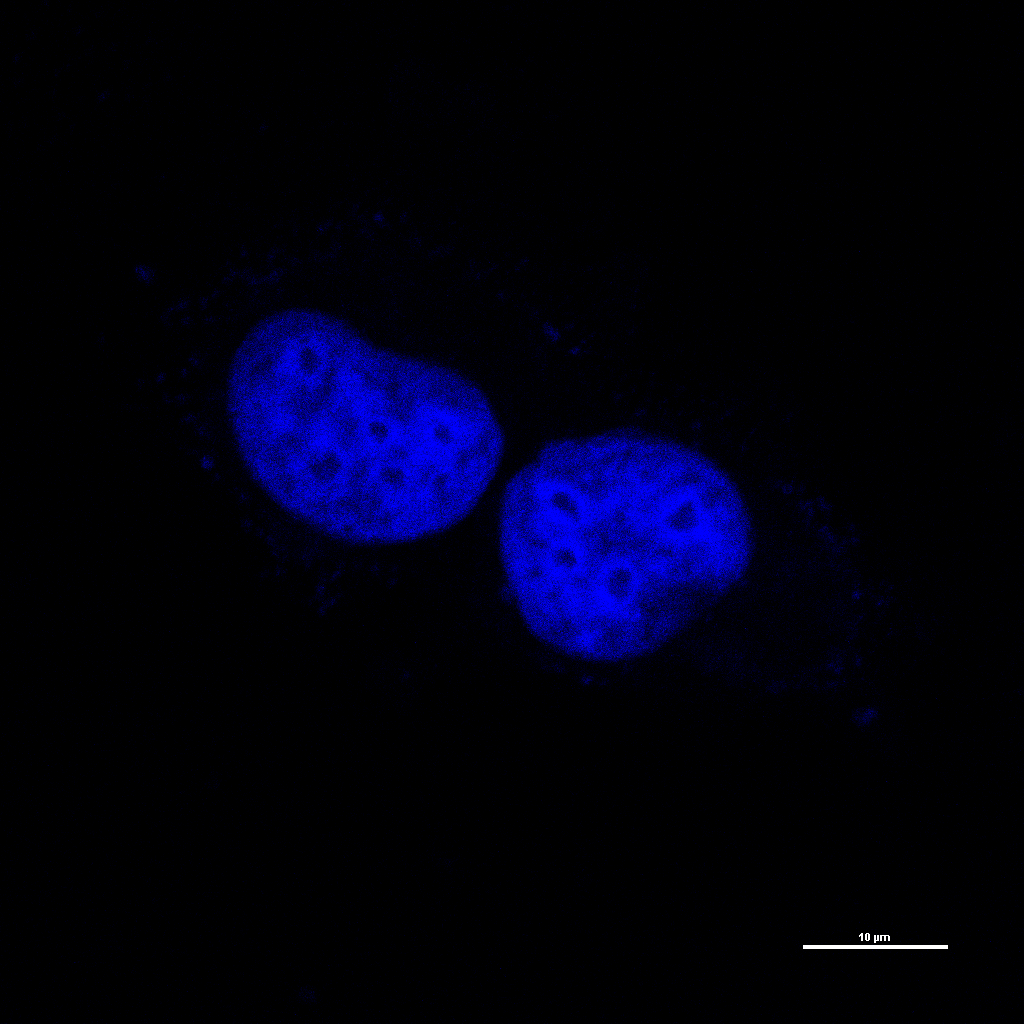

Supplement: Supplementary file 8 — Source data Fig. 6 [file 44318_2024_353_MOESM8_ESM.zip › Figure 6/Figure 6/6K/PLCPRF5 siSAG+RHEB-Q64L/HP_RGB_DAPI.tif]

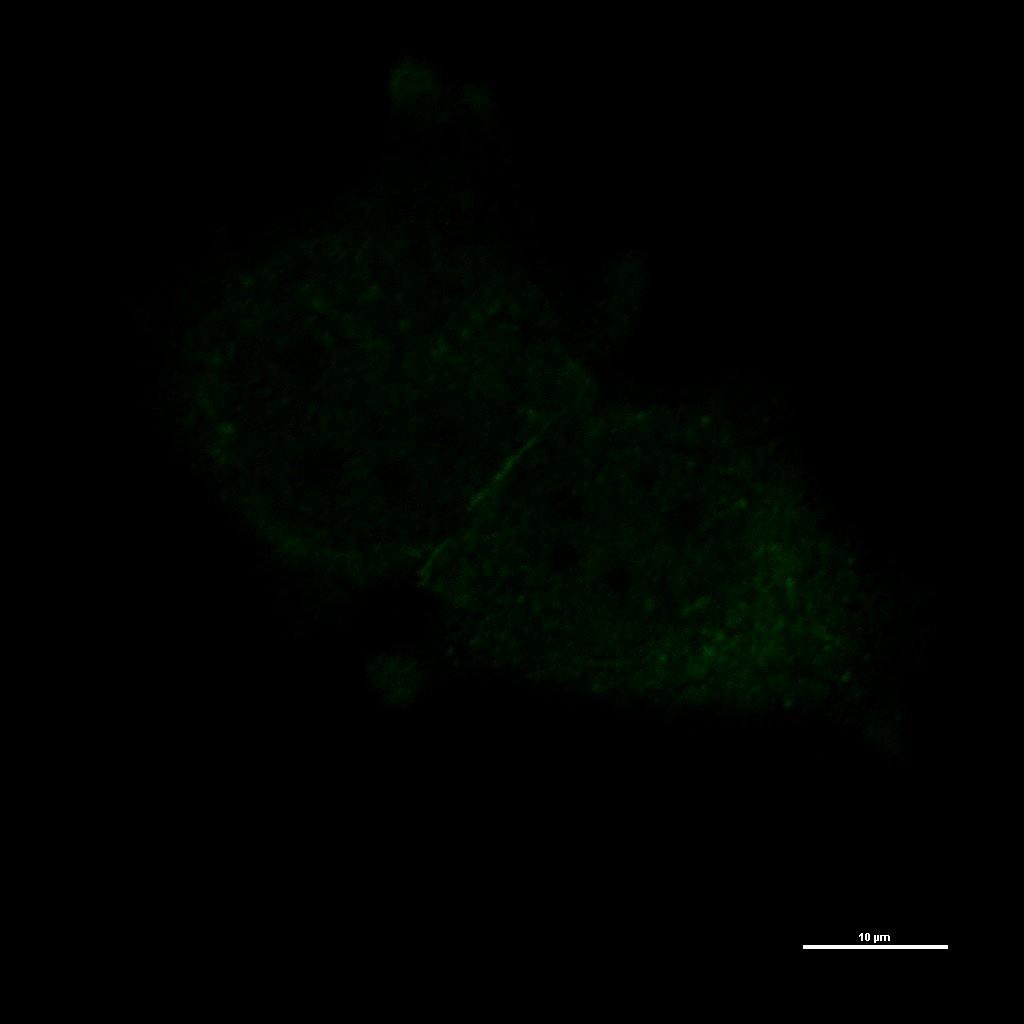

Supplement: Supplementary file 8 — Source data Fig. 6 [file 44318_2024_353_MOESM8_ESM.zip › Figure 6/Figure 6/6K/PLCPRF5 siSAG+RHEB-Q64L/HP_RGB_FITC.tif]

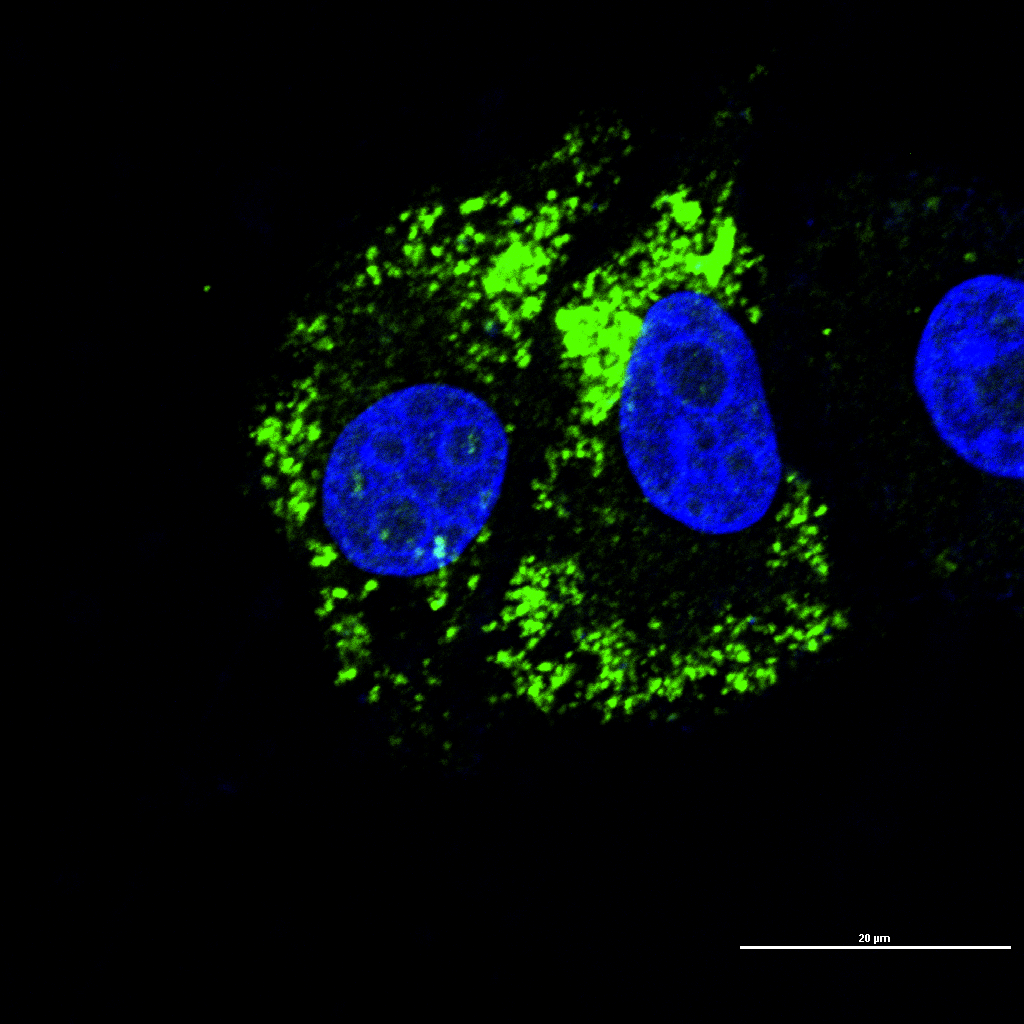

Supplement: Supplementary file 8 — Source data Fig. 6 [file 44318_2024_353_MOESM8_ESM.zip › Figure 6/Figure 6/6K/PLCPRF5 siSAG+Vector/HP_2024_02_25_RGB.tif]

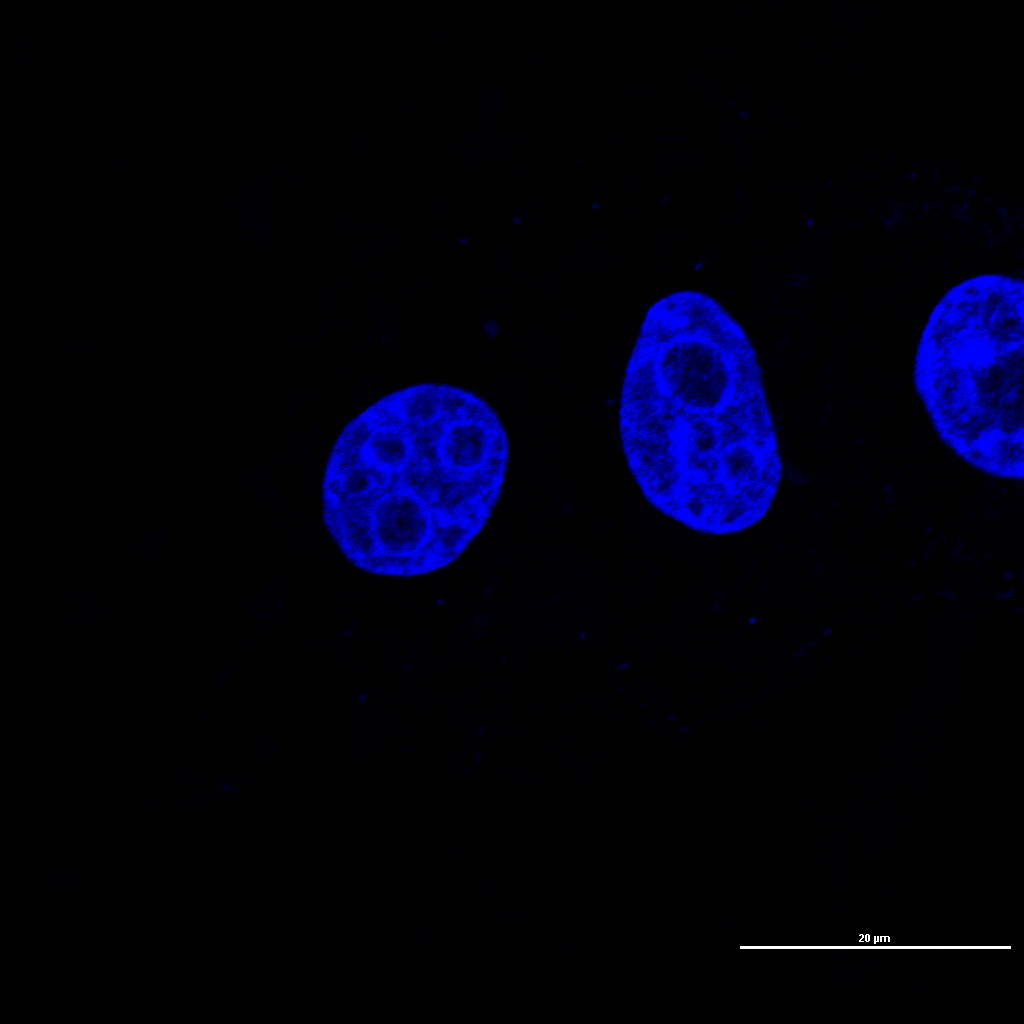

Supplement: Supplementary file 8 — Source data Fig. 6 [file 44318_2024_353_MOESM8_ESM.zip › Figure 6/Figure 6/6K/PLCPRF5 siSAG+Vector/HP_2024_02_25_RGB_DAPI.tif]

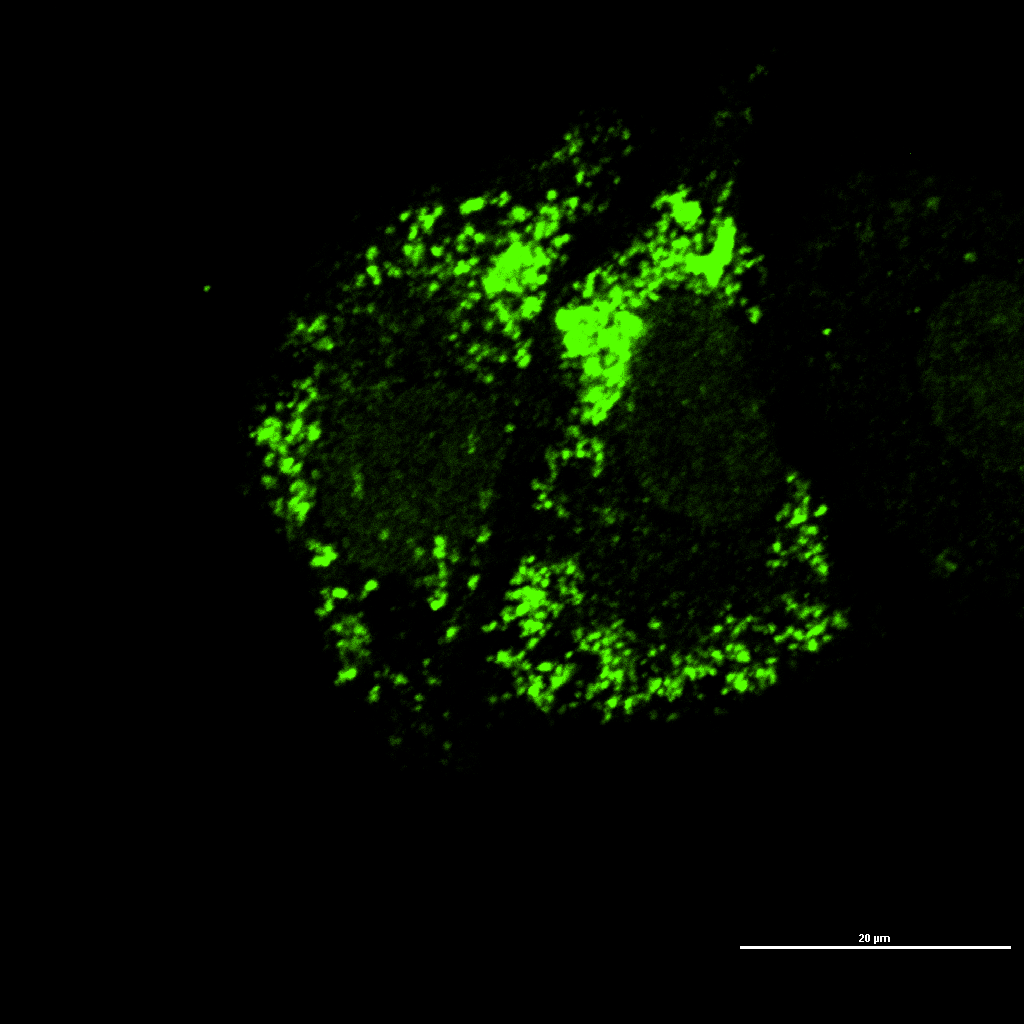

Supplement: Supplementary file 8 — Source data Fig. 6 [file 44318_2024_353_MOESM8_ESM.zip › Figure 6/Figure 6/6K/PLCPRF5 siSAG+Vector/HP_2024_02_25_RGB_FITC.tif]

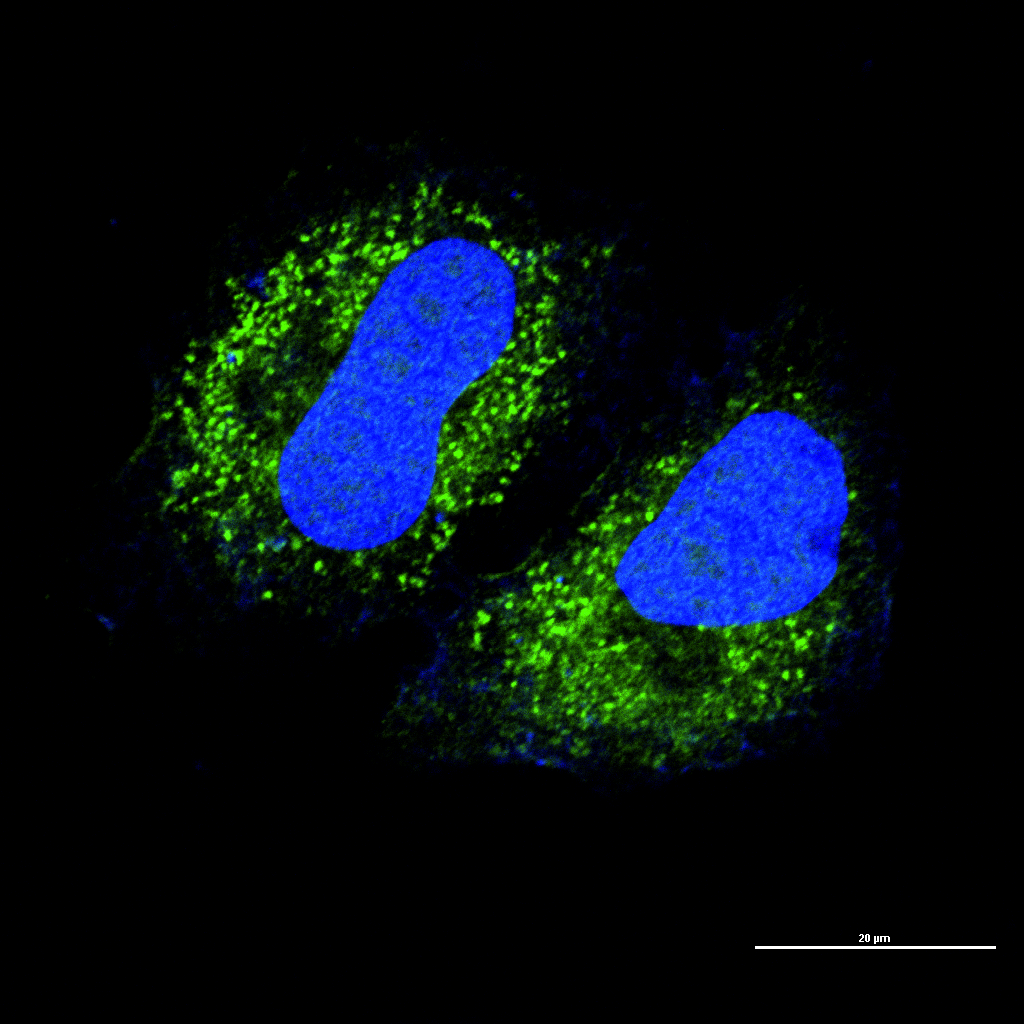

Supplement: Supplementary file 8 — Source data Fig. 6 [file 44318_2024_353_MOESM8_ESM.zip › Figure 6/Figure 6/6K/PLCPRF5 siUBE2F+RHEB-DM/HP_2024_02_25_RGB.tif]

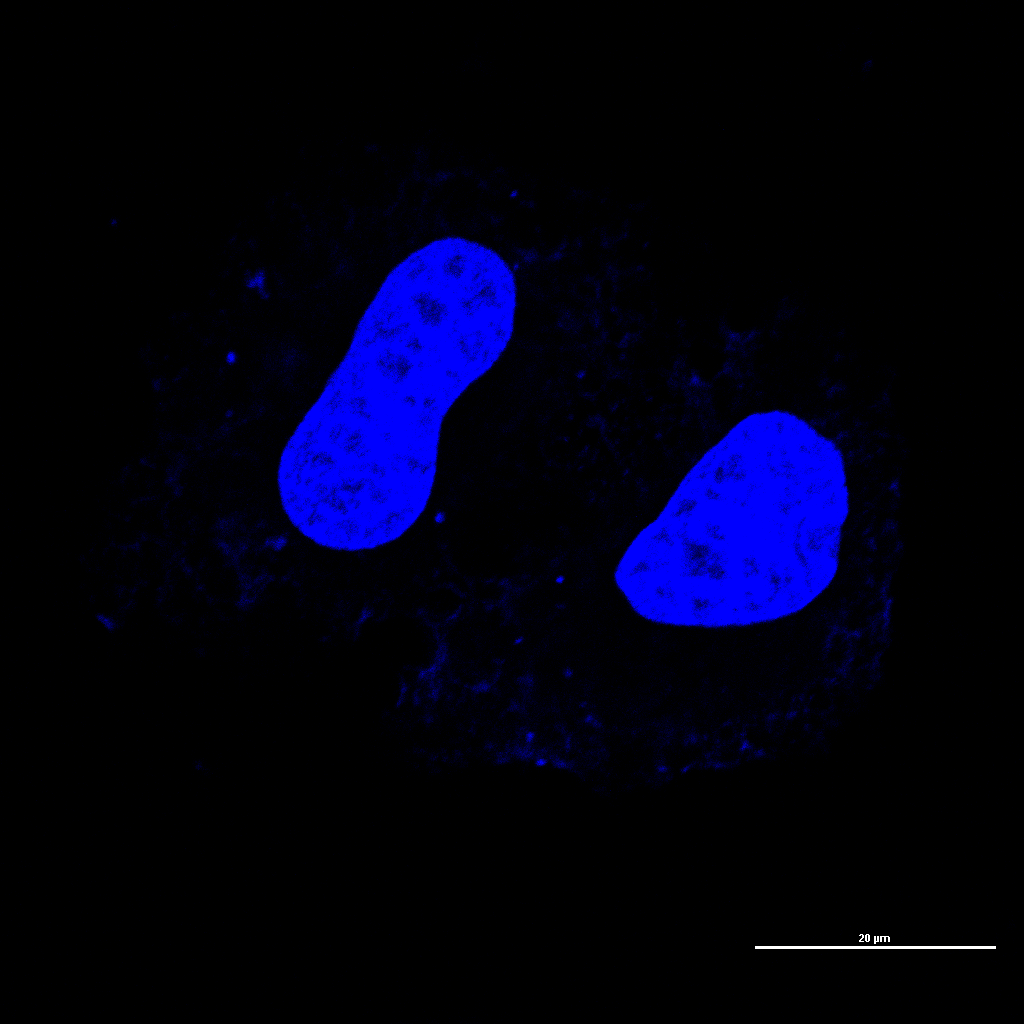

Supplement: Supplementary file 8 — Source data Fig. 6 [file 44318_2024_353_MOESM8_ESM.zip › Figure 6/Figure 6/6K/PLCPRF5 siUBE2F+RHEB-DM/HP_2024_02_25_RGB_DAPI.tif]

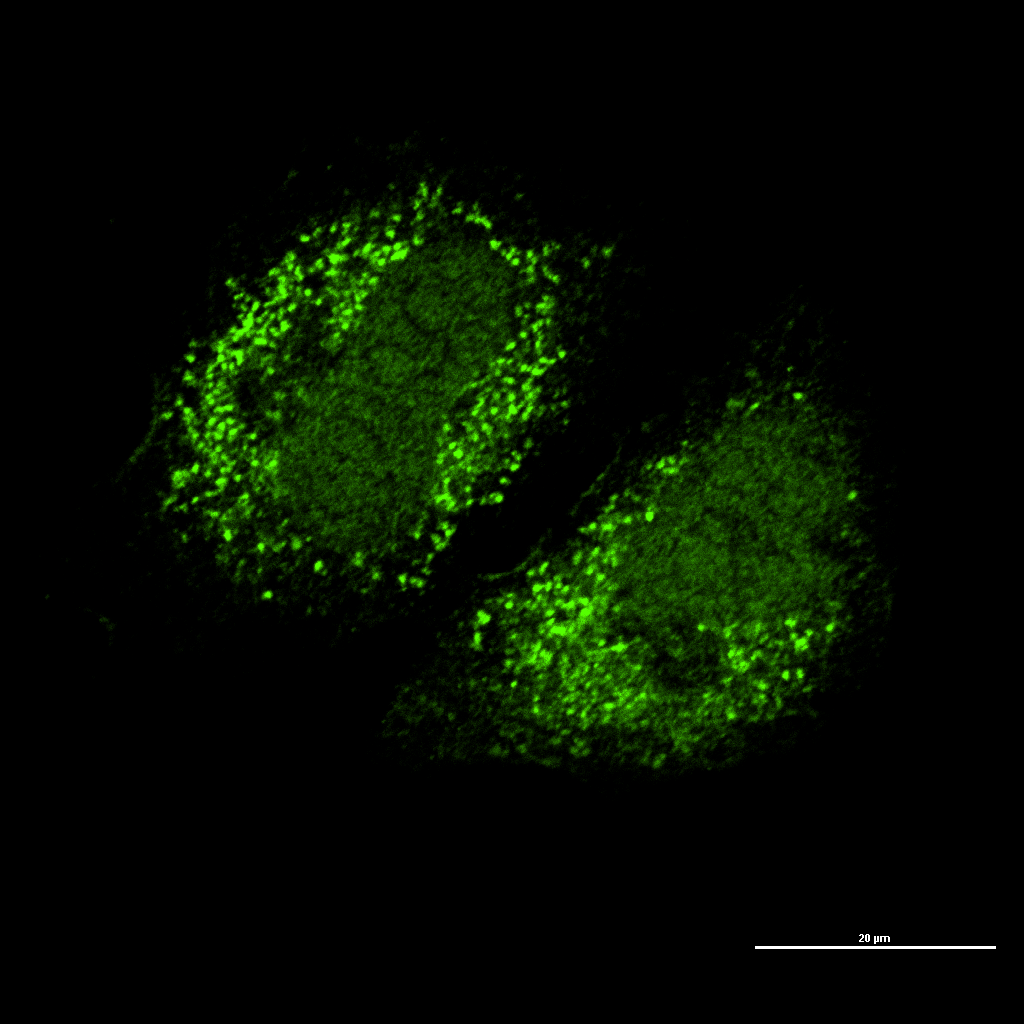

Supplement: Supplementary file 8 — Source data Fig. 6 [file 44318_2024_353_MOESM8_ESM.zip › Figure 6/Figure 6/6K/PLCPRF5 siUBE2F+RHEB-DM/HP_2024_02_25_RGB_FITC.tif]

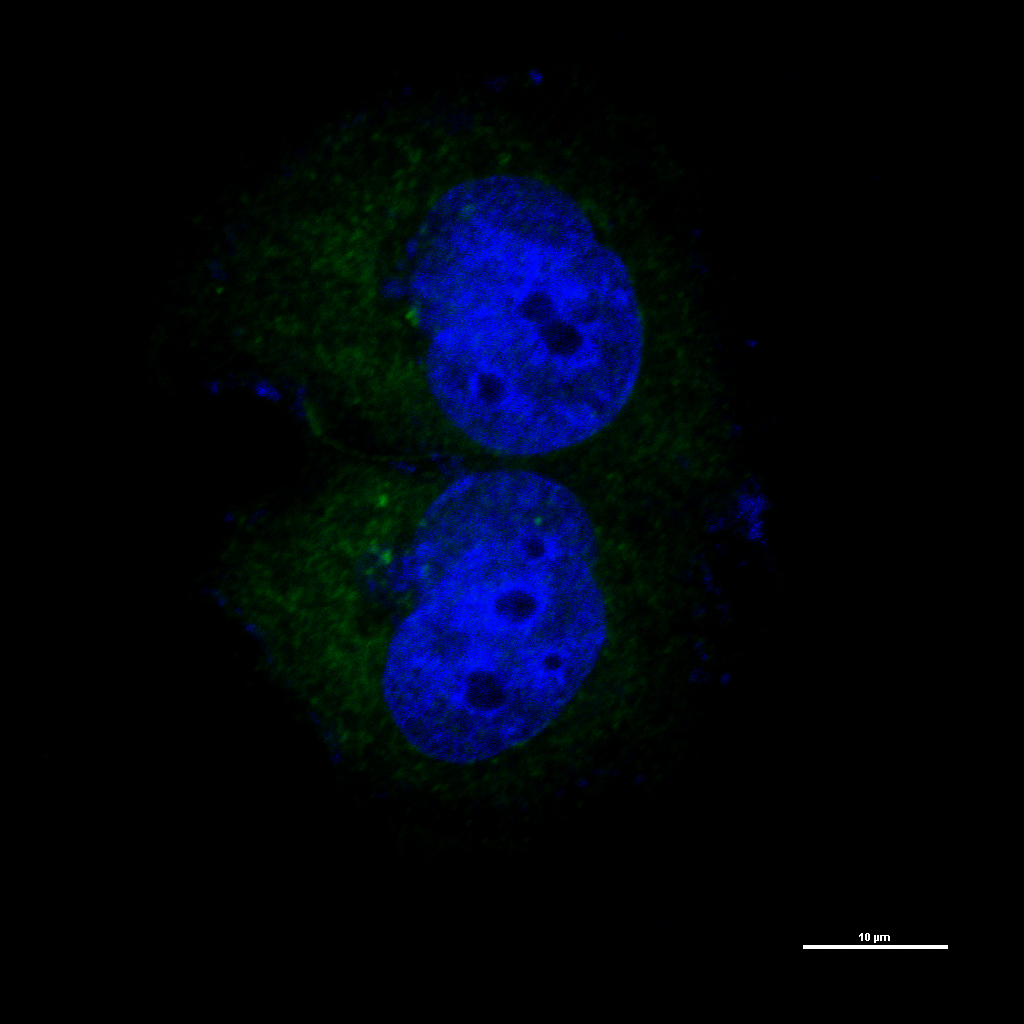

Supplement: Supplementary file 8 — Source data Fig. 6 [file 44318_2024_353_MOESM8_ESM.zip › Figure 6/Figure 6/6K/PLCPRF5 siUBE2F+RHEB-Q64L/HP_RGB.tif]

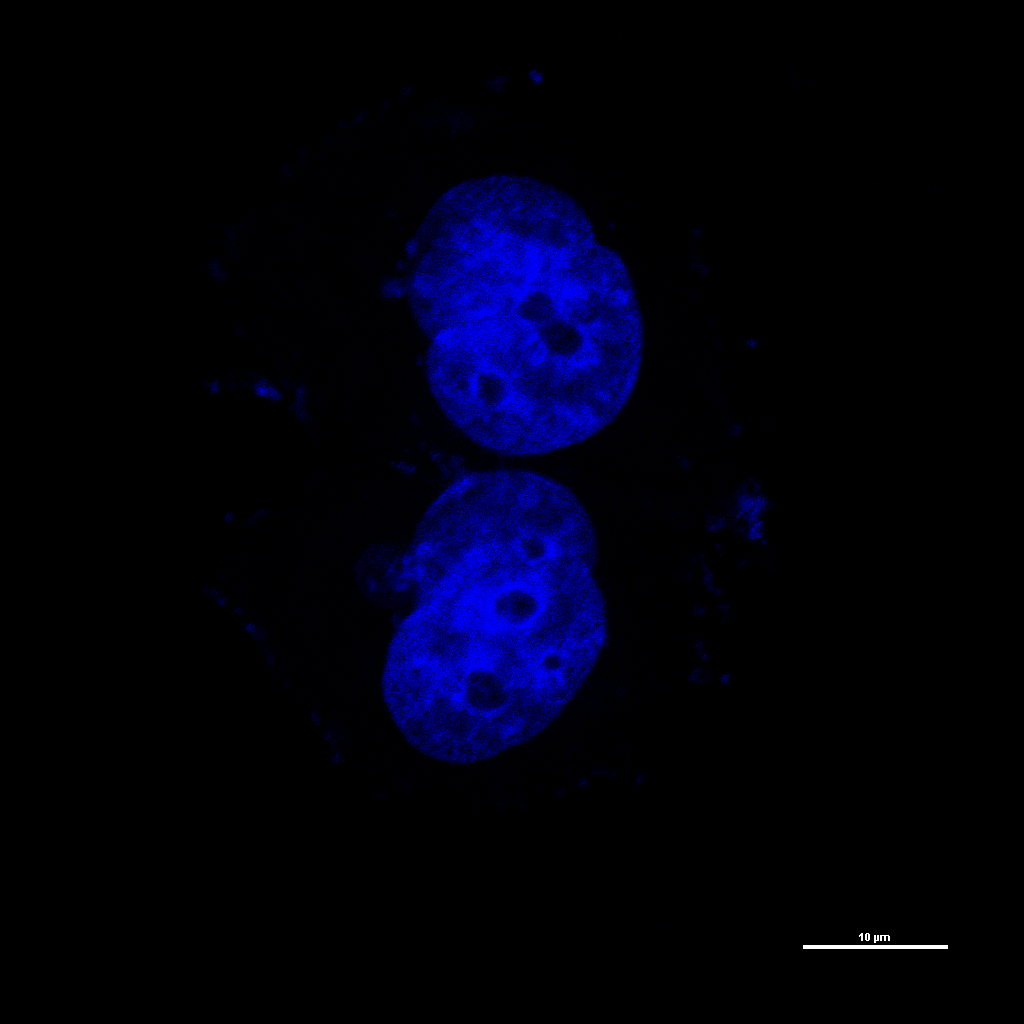

Supplement: Supplementary file 8 — Source data Fig. 6 [file 44318_2024_353_MOESM8_ESM.zip › Figure 6/Figure 6/6K/PLCPRF5 siUBE2F+RHEB-Q64L/HP_RGB_DAPI.tif]

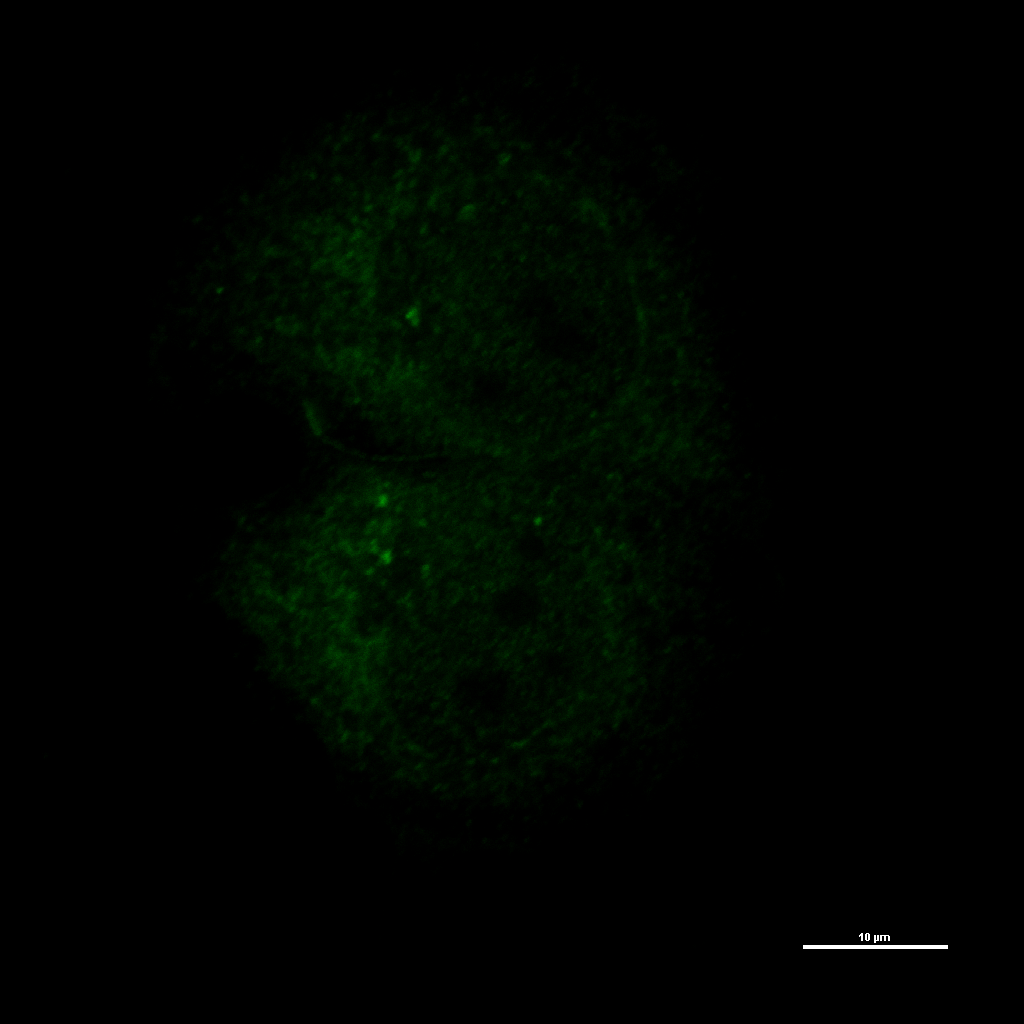

Supplement: Supplementary file 8 — Source data Fig. 6 [file 44318_2024_353_MOESM8_ESM.zip › Figure 6/Figure 6/6K/PLCPRF5 siUBE2F+RHEB-Q64L/HP_RGB_FITC.tif]

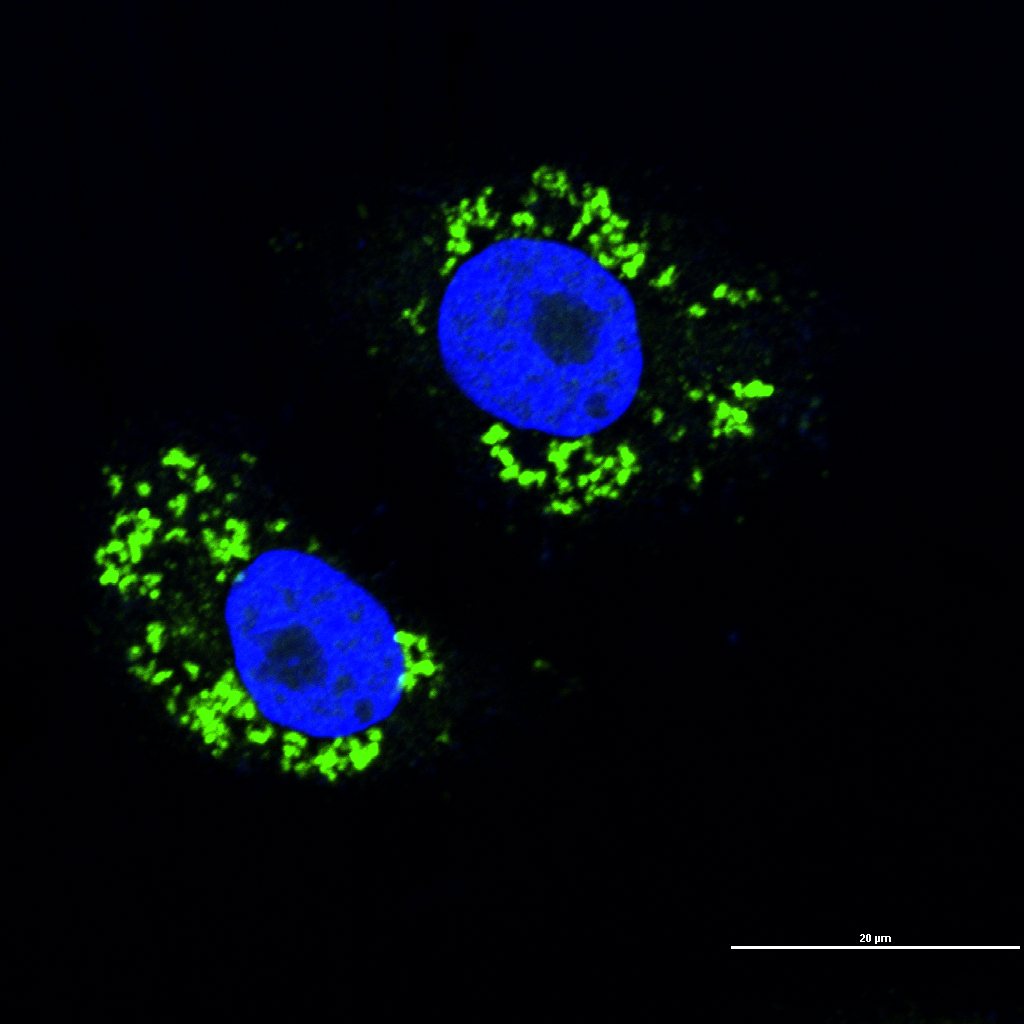

Supplement: Supplementary file 8 — Source data Fig. 6 [file 44318_2024_353_MOESM8_ESM.zip › Figure 6/Figure 6/6K/PLCPRF5 siUBE2F+Vector/HP_2024_02_25_RGB.tif]

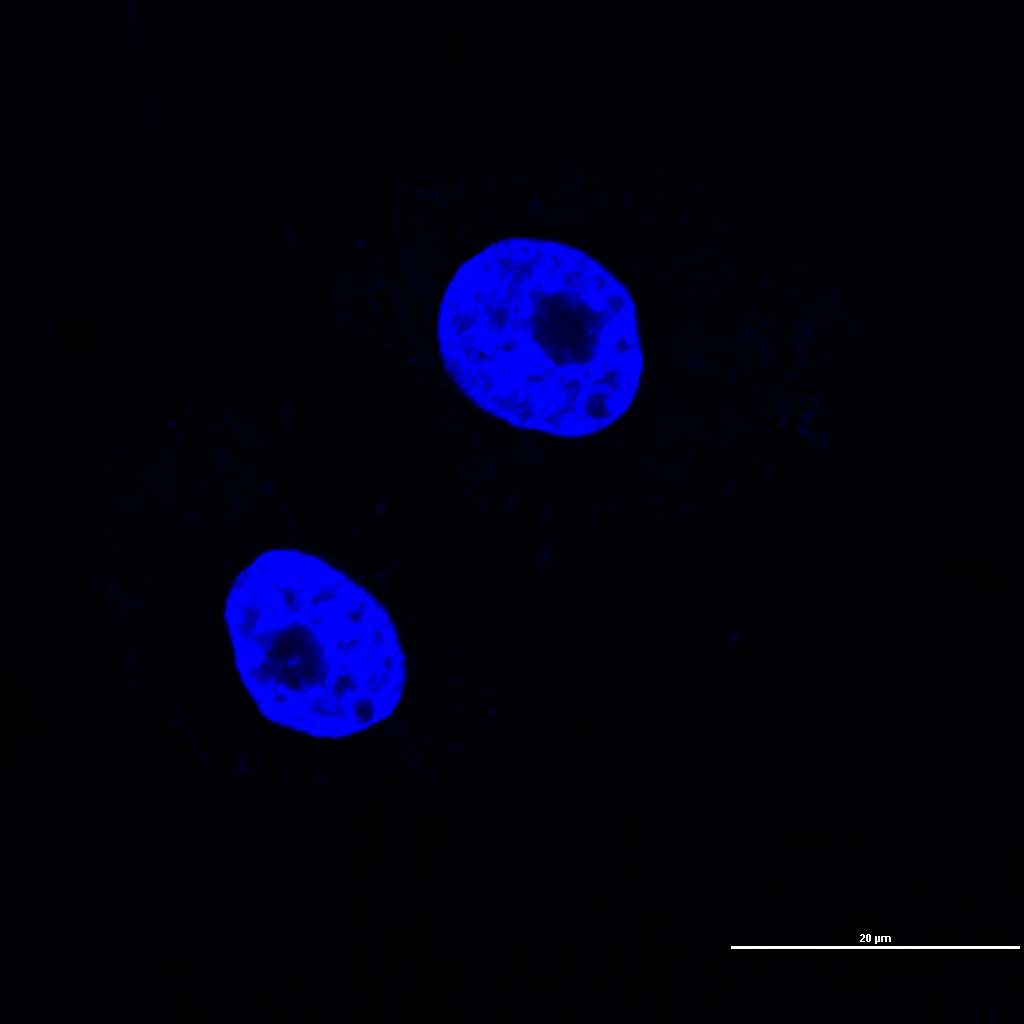

Supplement: Supplementary file 8 — Source data Fig. 6 [file 44318_2024_353_MOESM8_ESM.zip › Figure 6/Figure 6/6K/PLCPRF5 siUBE2F+Vector/HP_2024_02_25_RGB_DAPI.tif]

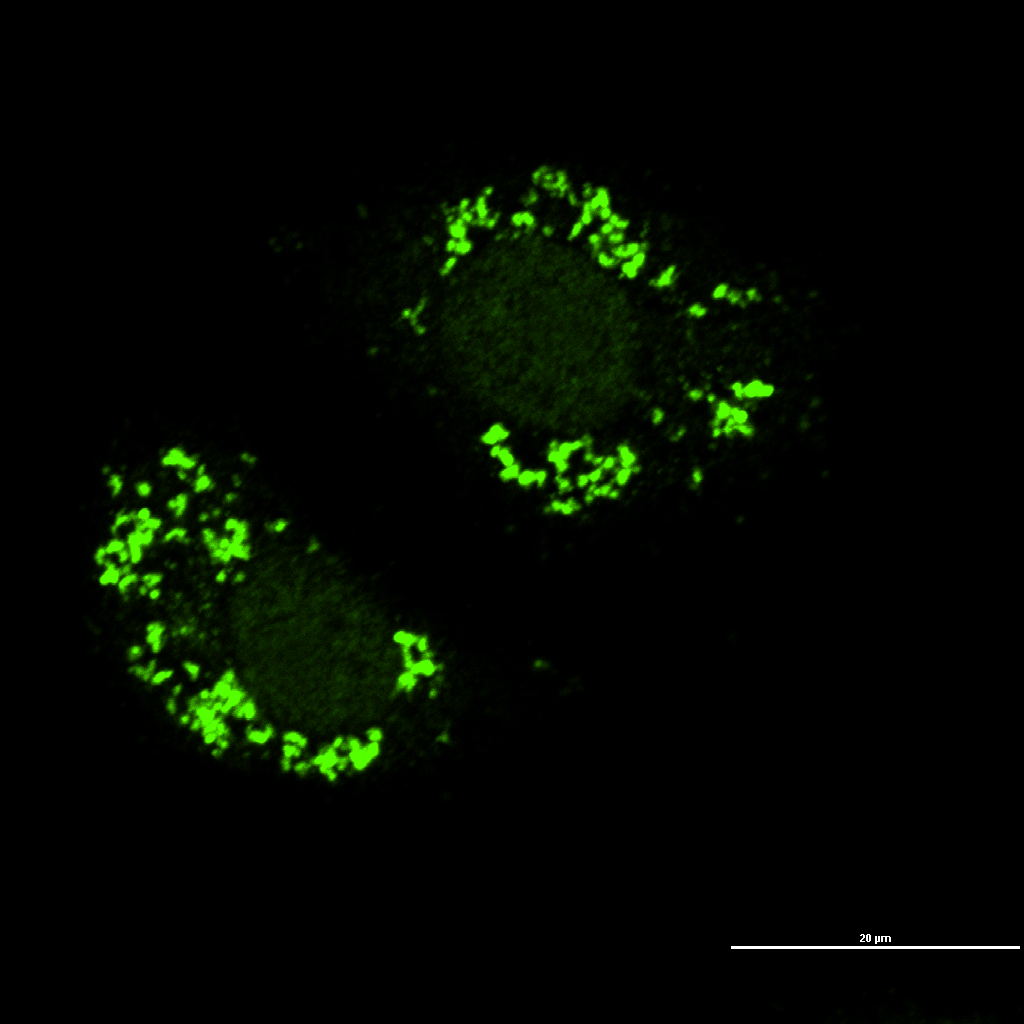

Supplement: Supplementary file 8 — Source data Fig. 6 [file 44318_2024_353_MOESM8_ESM.zip › Figure 6/Figure 6/6K/PLCPRF5 siUBE2F+Vector/HP_2024_02_25_RGB_FITC.tif]

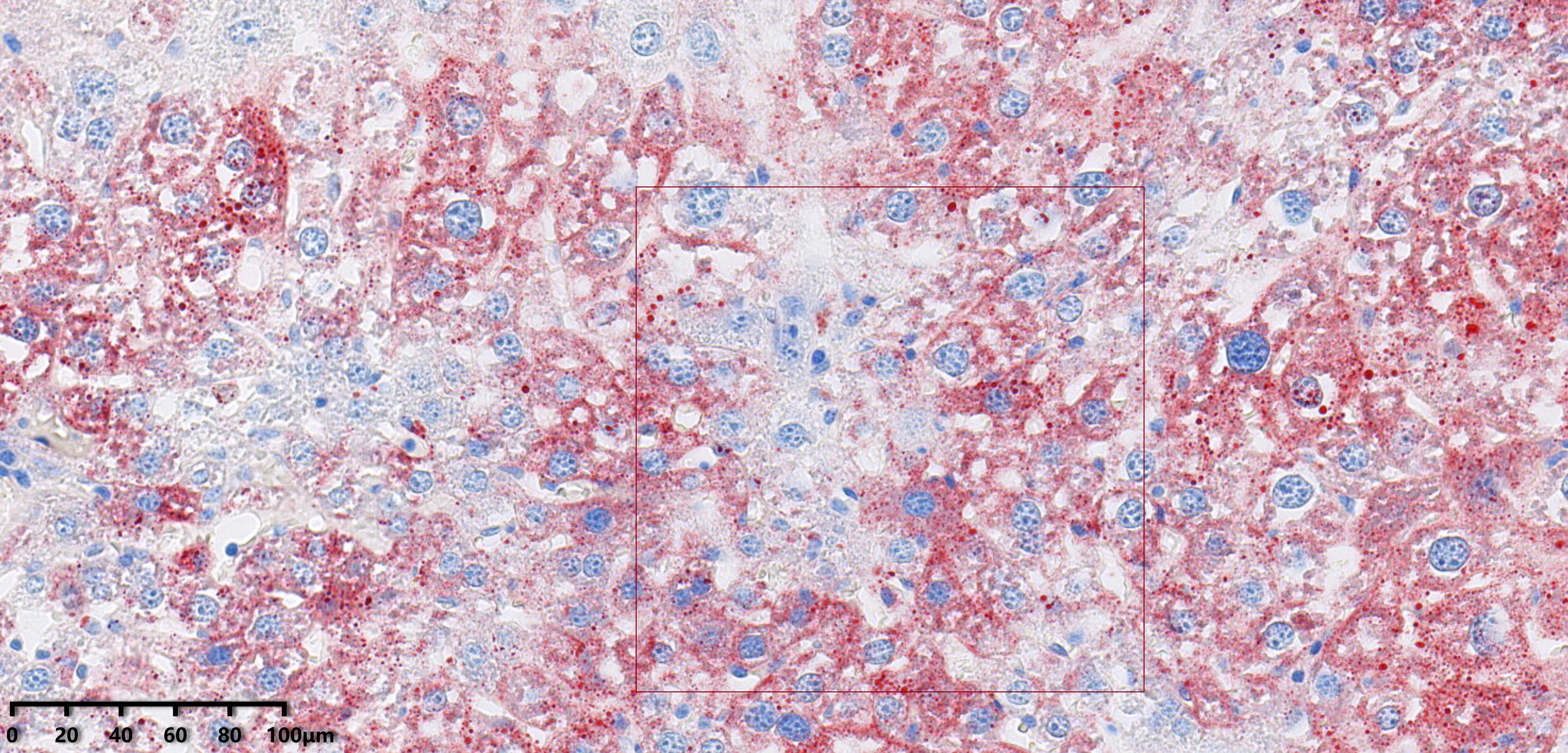

Supplement: Supplementary file 9 — Source data Fig. 7 [file 44318_2024_353_MOESM9_ESM.zip › Figure 7/7A/3 months KO insert.jpg]

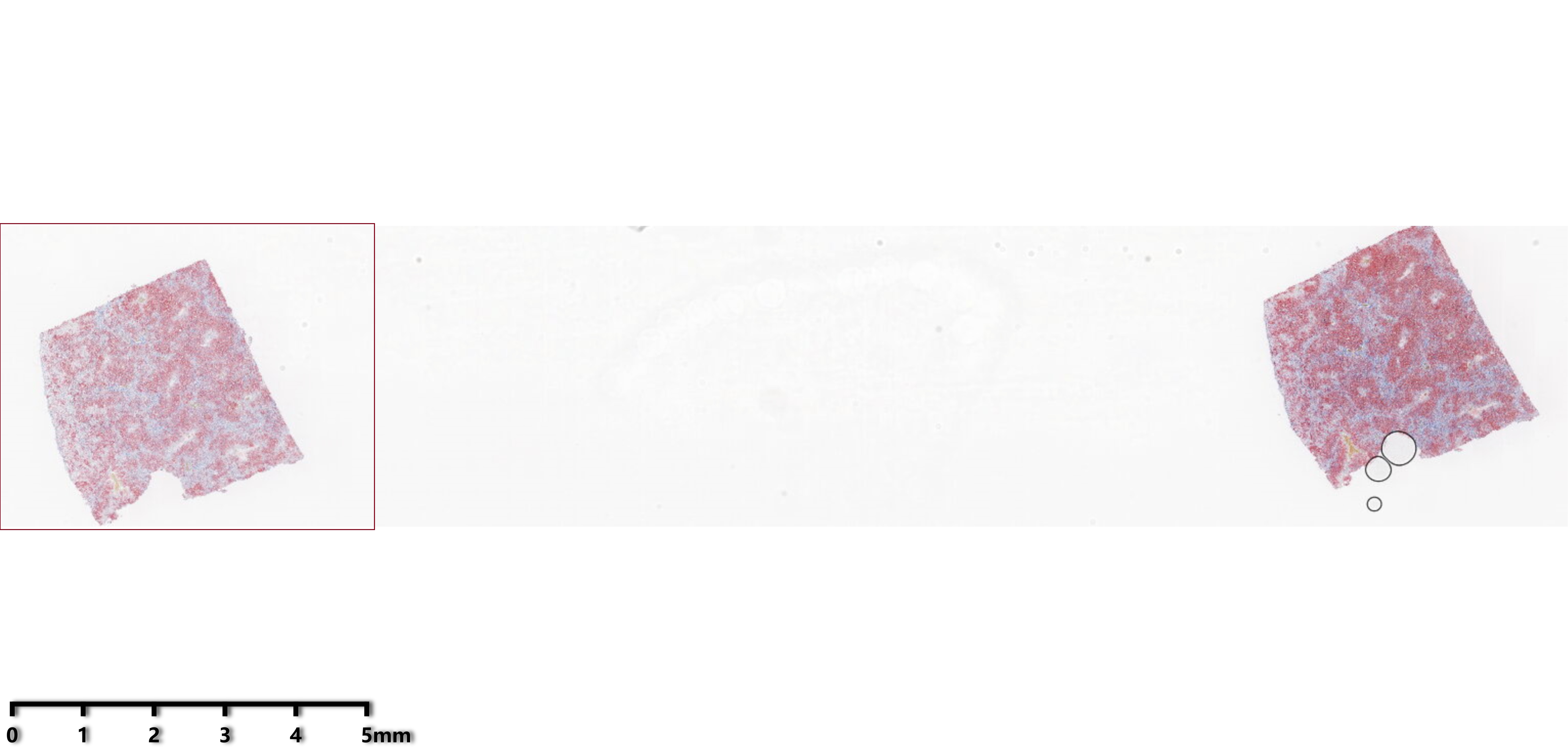

Supplement: Supplementary file 9 — Source data Fig. 7 [file 44318_2024_353_MOESM9_ESM.zip › Figure 7/7A/3 months KO.jpg]

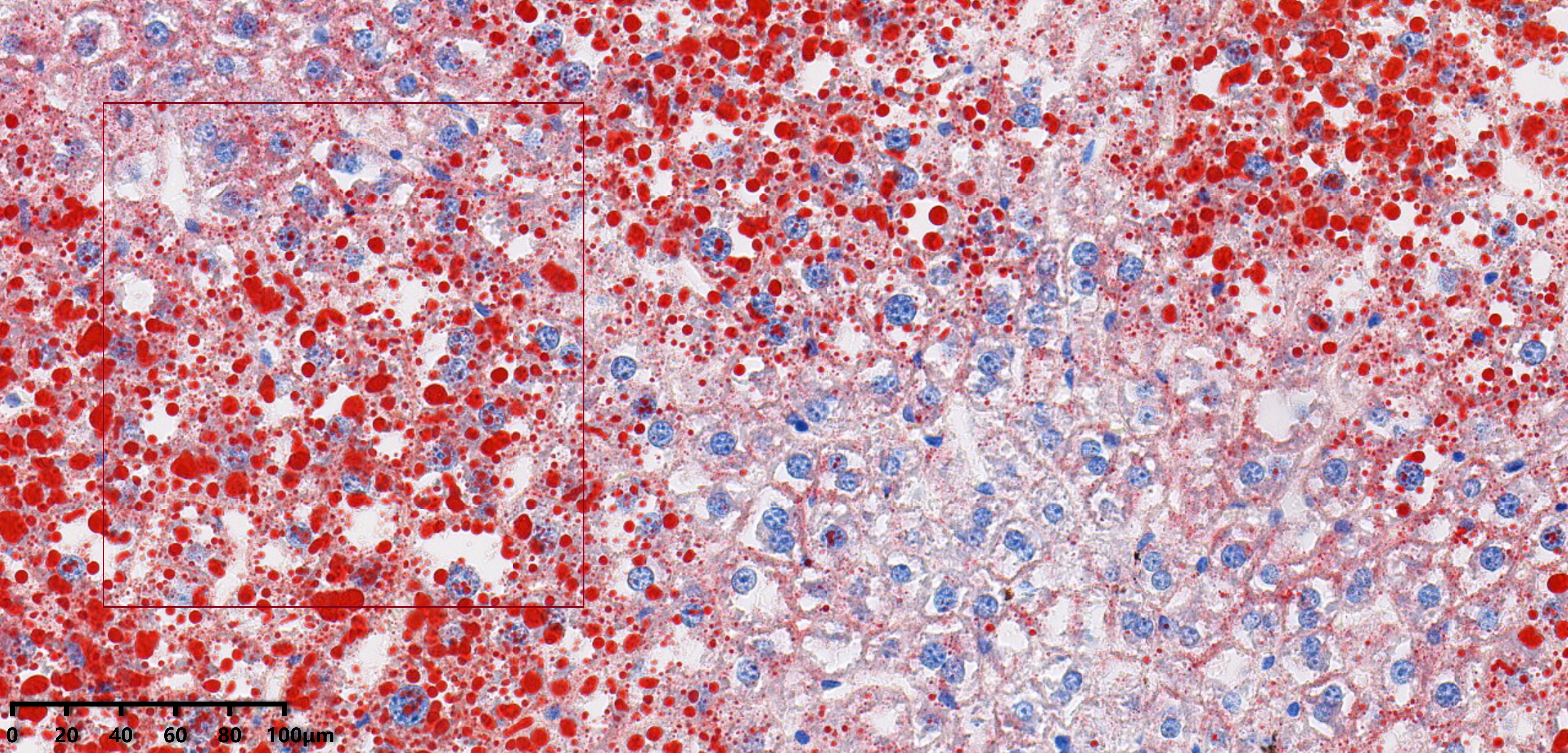

Supplement: Supplementary file 9 — Source data Fig. 7 [file 44318_2024_353_MOESM9_ESM.zip › Figure 7/7A/3 months WT insert.jpg]

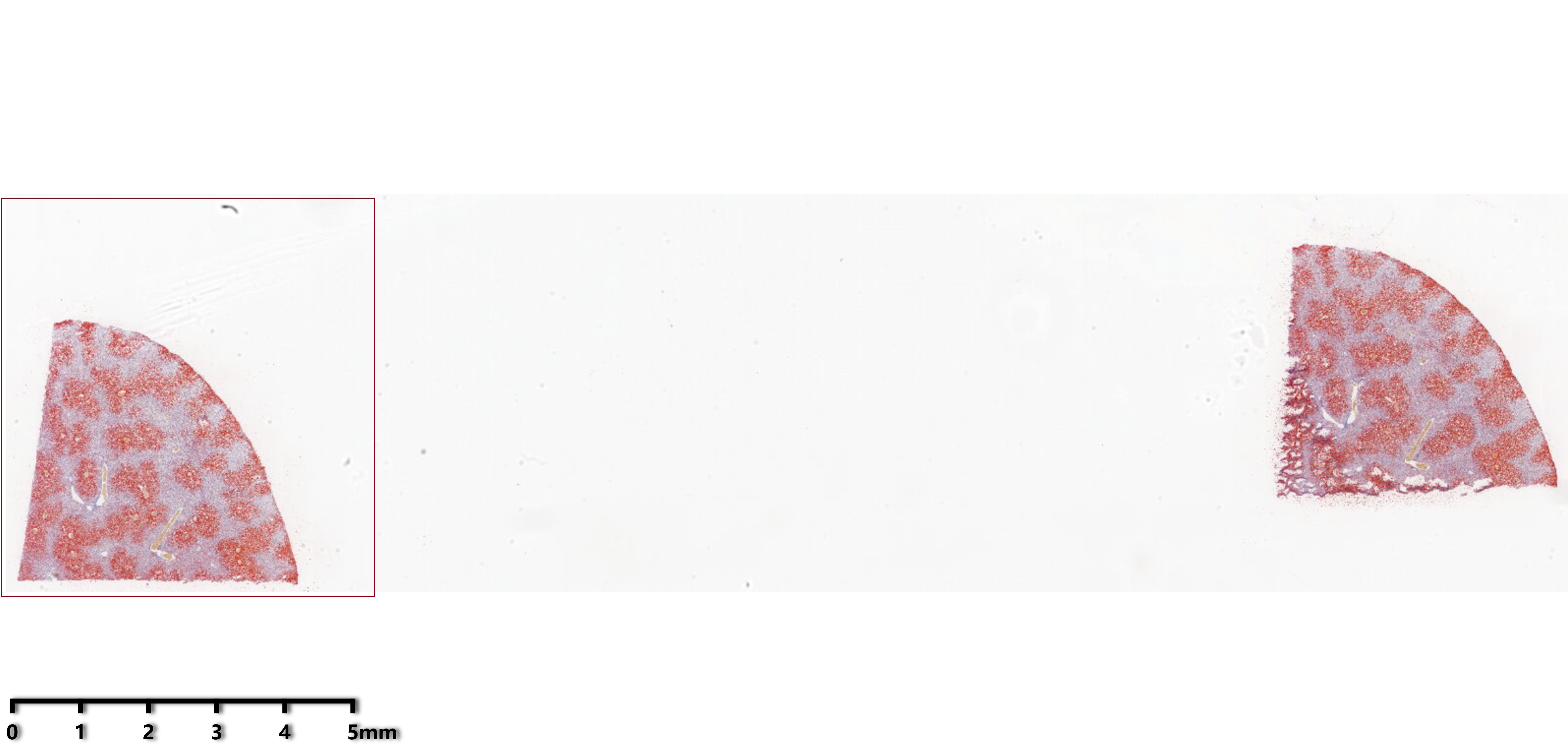

Supplement: Supplementary file 9 — Source data Fig. 7 [file 44318_2024_353_MOESM9_ESM.zip › Figure 7/7A/3 nomths WT.jpg]

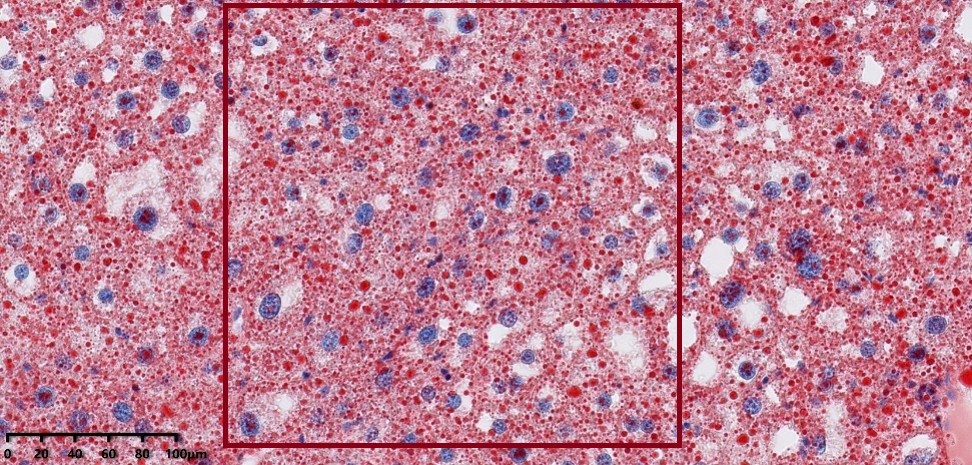

Supplement: Supplementary file 9 — Source data Fig. 7 [file 44318_2024_353_MOESM9_ESM.zip › Figure 7/7A/6 months KO insert.jpg]

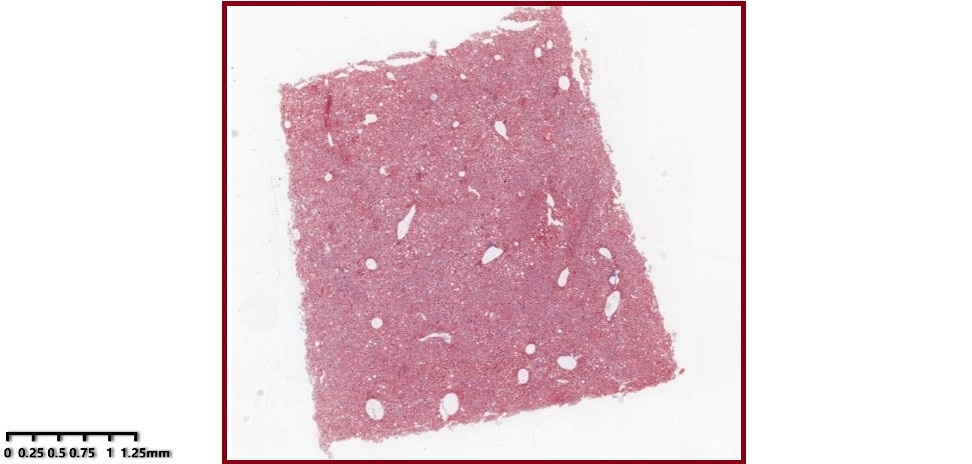

Supplement: Supplementary file 9 — Source data Fig. 7 [file 44318_2024_353_MOESM9_ESM.zip › Figure 7/7A/6 months KO.jpg]

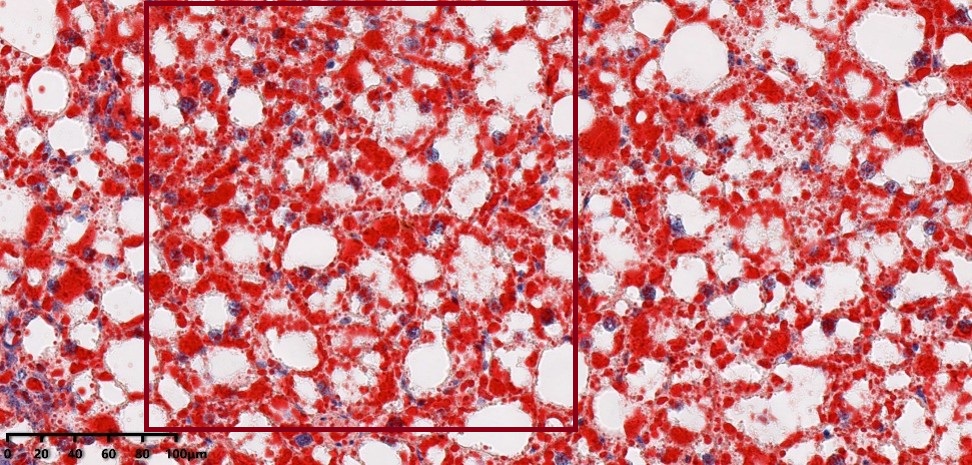

Supplement: Supplementary file 9 — Source data Fig. 7 [file 44318_2024_353_MOESM9_ESM.zip › Figure 7/7A/6 months WT insert.jpg]

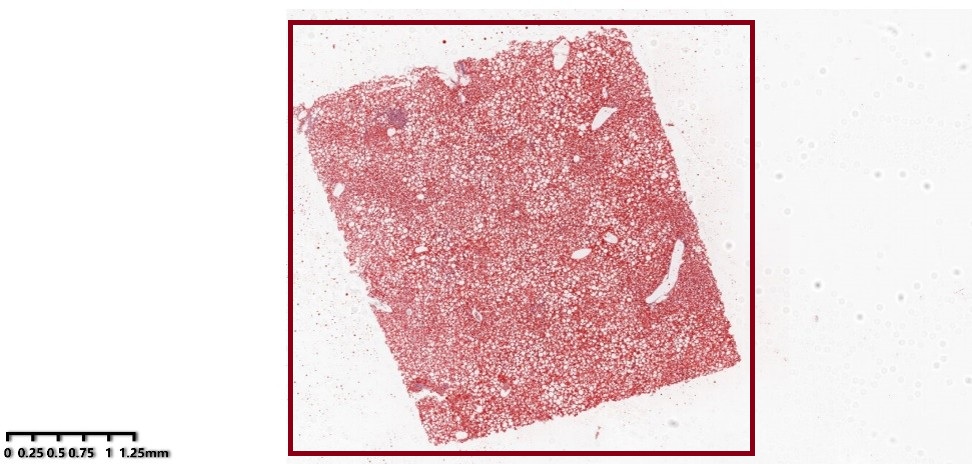

Supplement: Supplementary file 9 — Source data Fig. 7 [file 44318_2024_353_MOESM9_ESM.zip › Figure 7/7A/6 months WT.jpg]

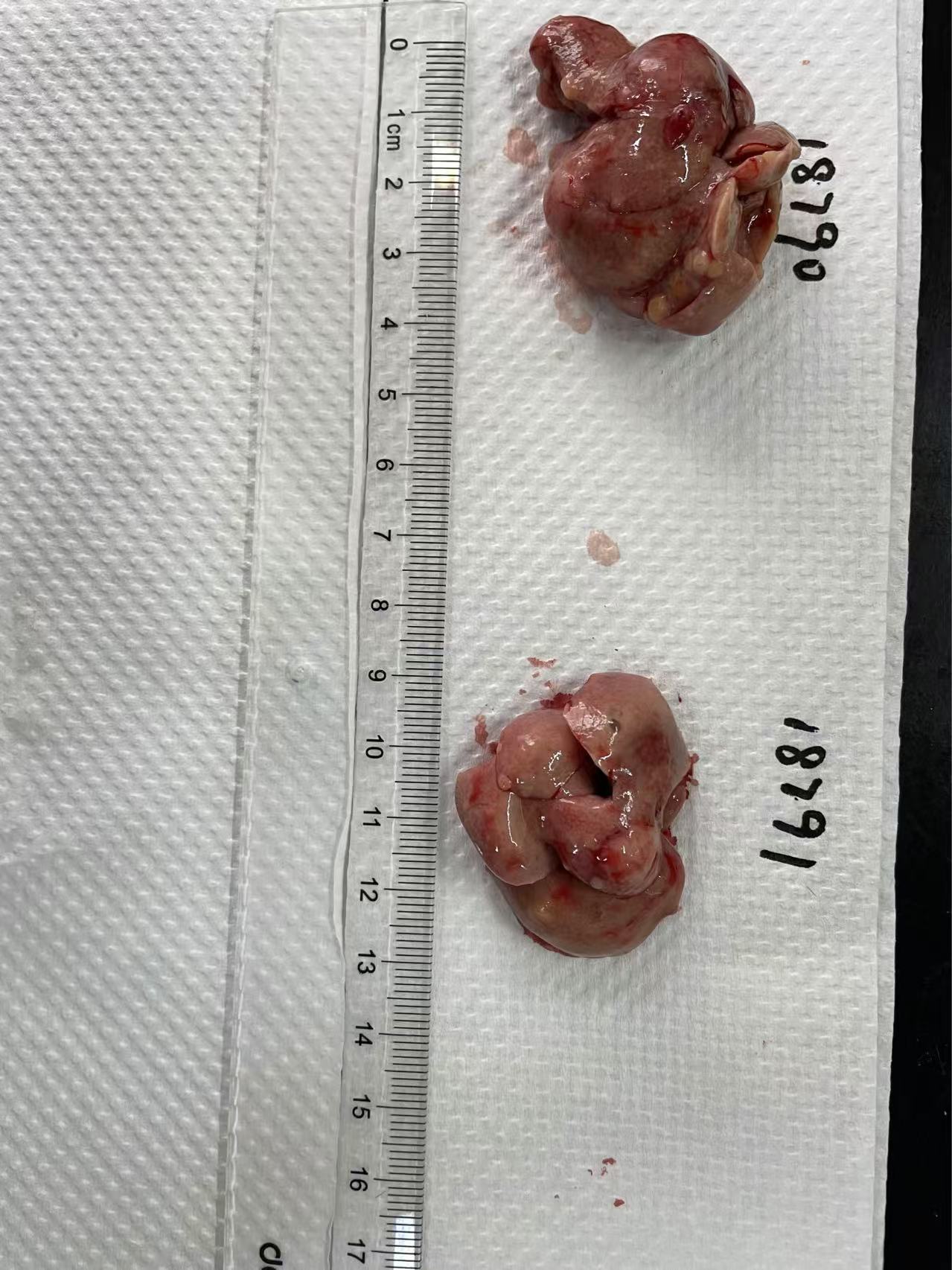

Supplement: Supplementary file 9 — Source data Fig. 7 [file 44318_2024_353_MOESM9_ESM.zip › Figure 7/7D/7D-1.jpg]

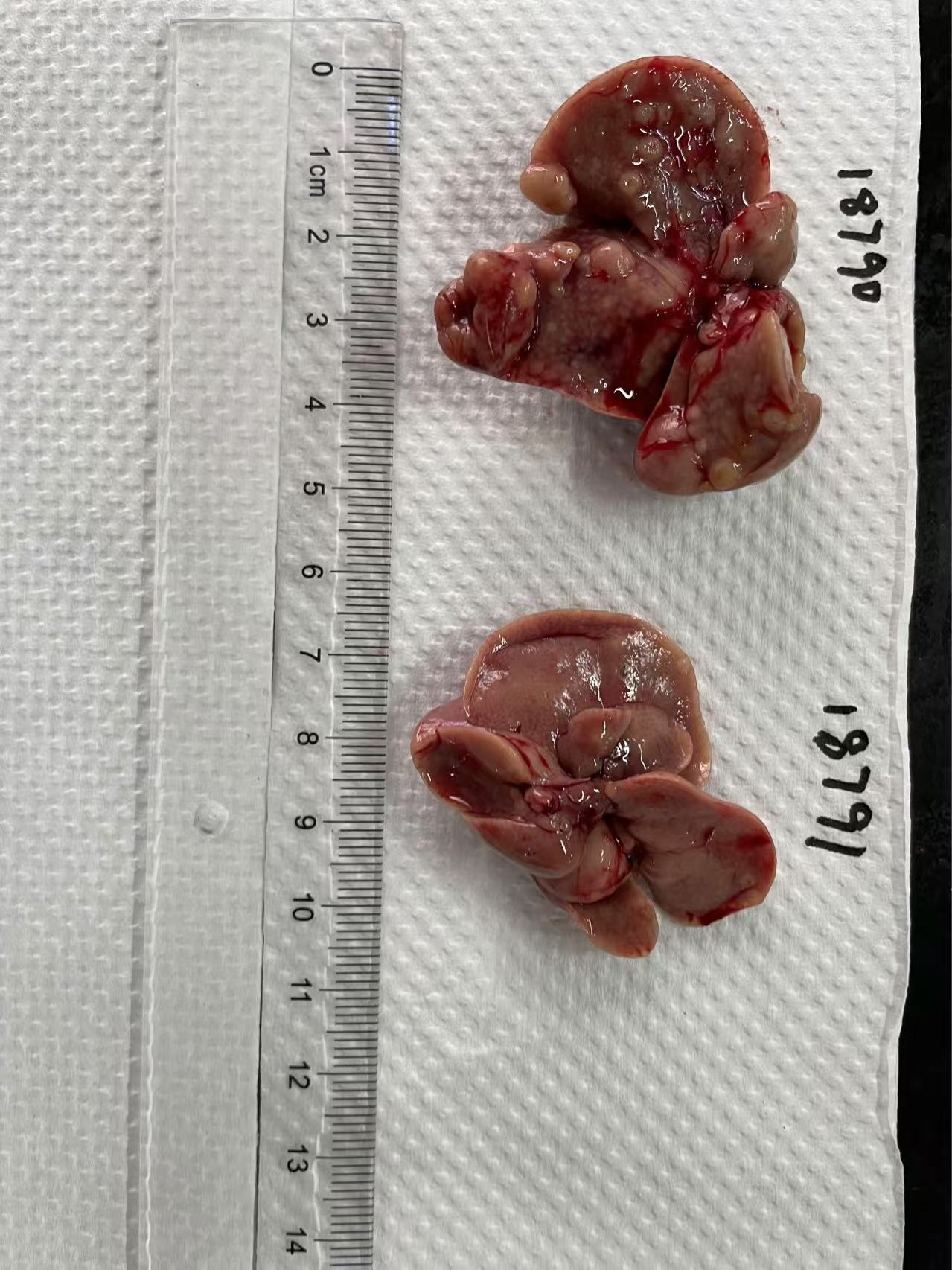

Supplement: Supplementary file 9 — Source data Fig. 7 [file 44318_2024_353_MOESM9_ESM.zip › Figure 7/7D/7D-2.jpg]

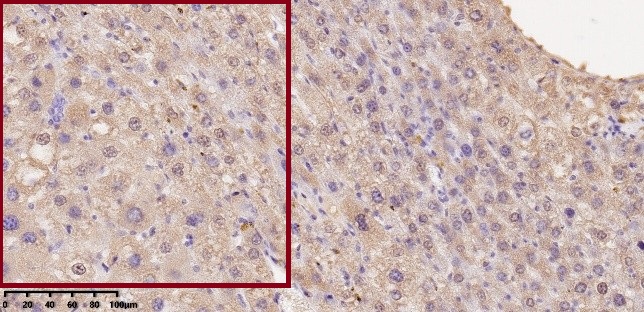

Supplement: Supplementary file 9 — Source data Fig. 7 [file 44318_2024_353_MOESM9_ESM.zip › Figure 7/7E/KO p-4Ebp1.jpg]

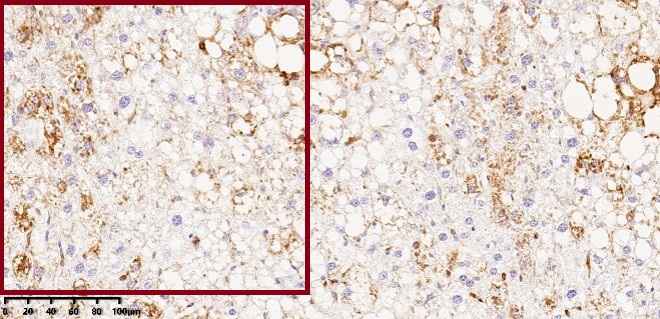

Supplement: Supplementary file 9 — Source data Fig. 7 [file 44318_2024_353_MOESM9_ESM.zip › Figure 7/7E/KO p-S6.jpg]

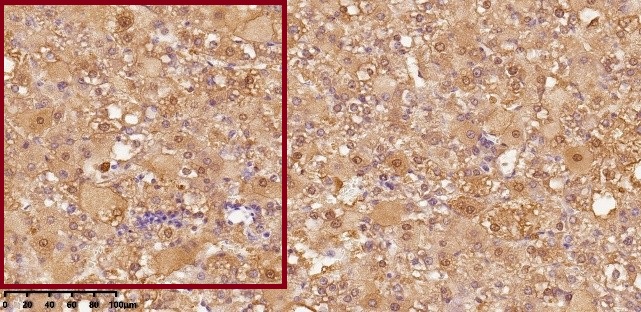

Supplement: Supplementary file 9 — Source data Fig. 7 [file 44318_2024_353_MOESM9_ESM.zip › Figure 7/7E/WT p-4Ebp1.jpg]

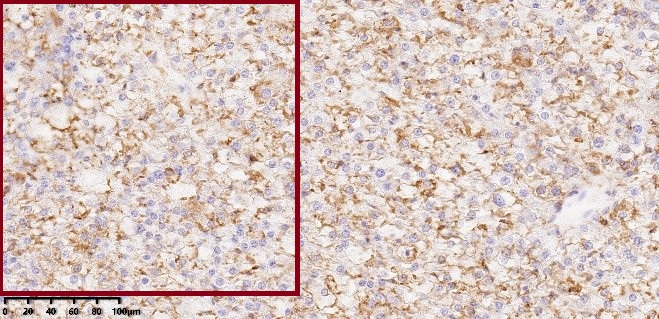

Supplement: Supplementary file 9 — Source data Fig. 7 [file 44318_2024_353_MOESM9_ESM.zip › Figure 7/7E/WT p-S6.jpg]

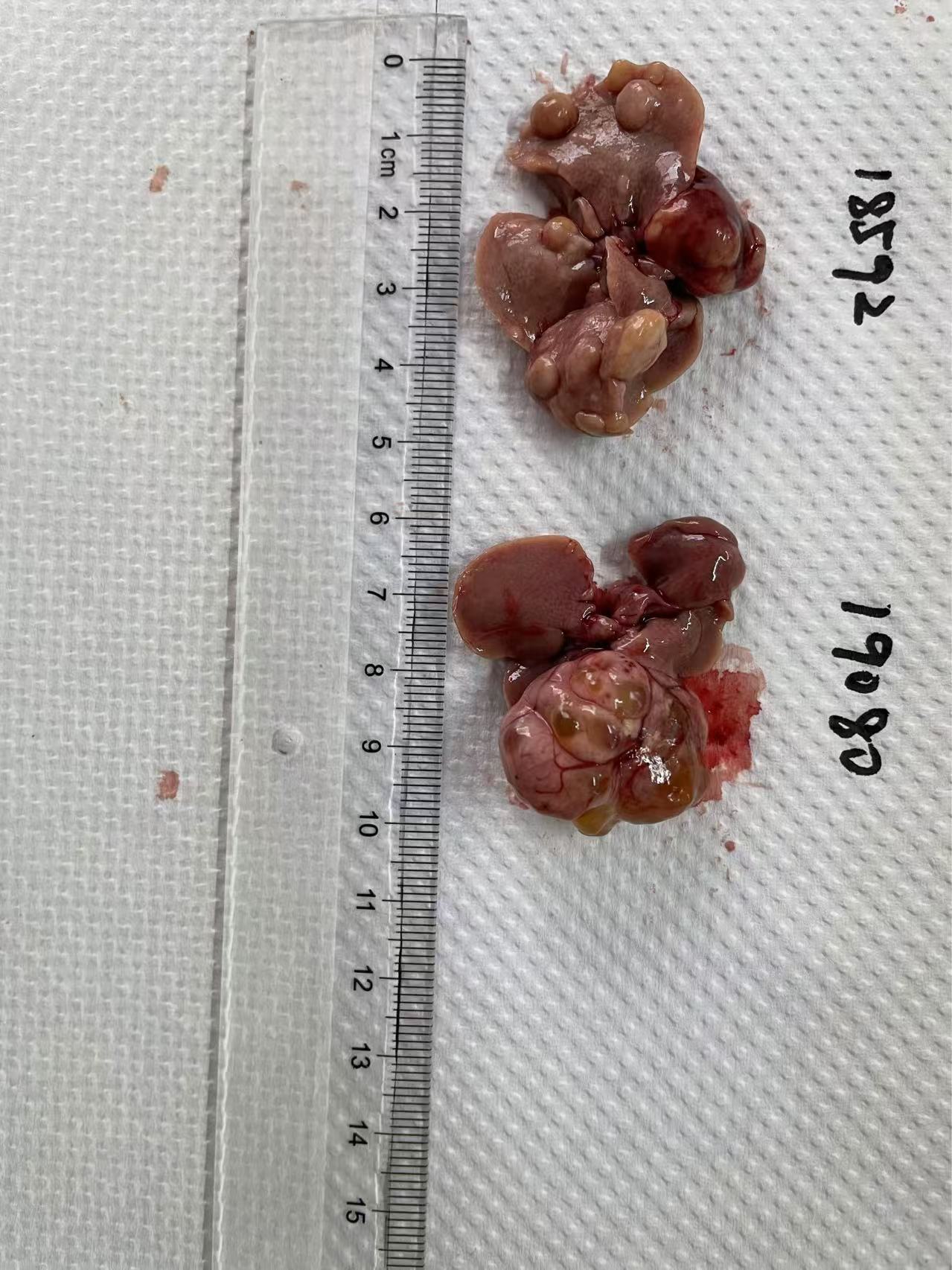

Supplement: Supplementary file 9 — Source data Fig. 7 [file 44318_2024_353_MOESM9_ESM.zip › Figure 7/7G/7G.jpg]

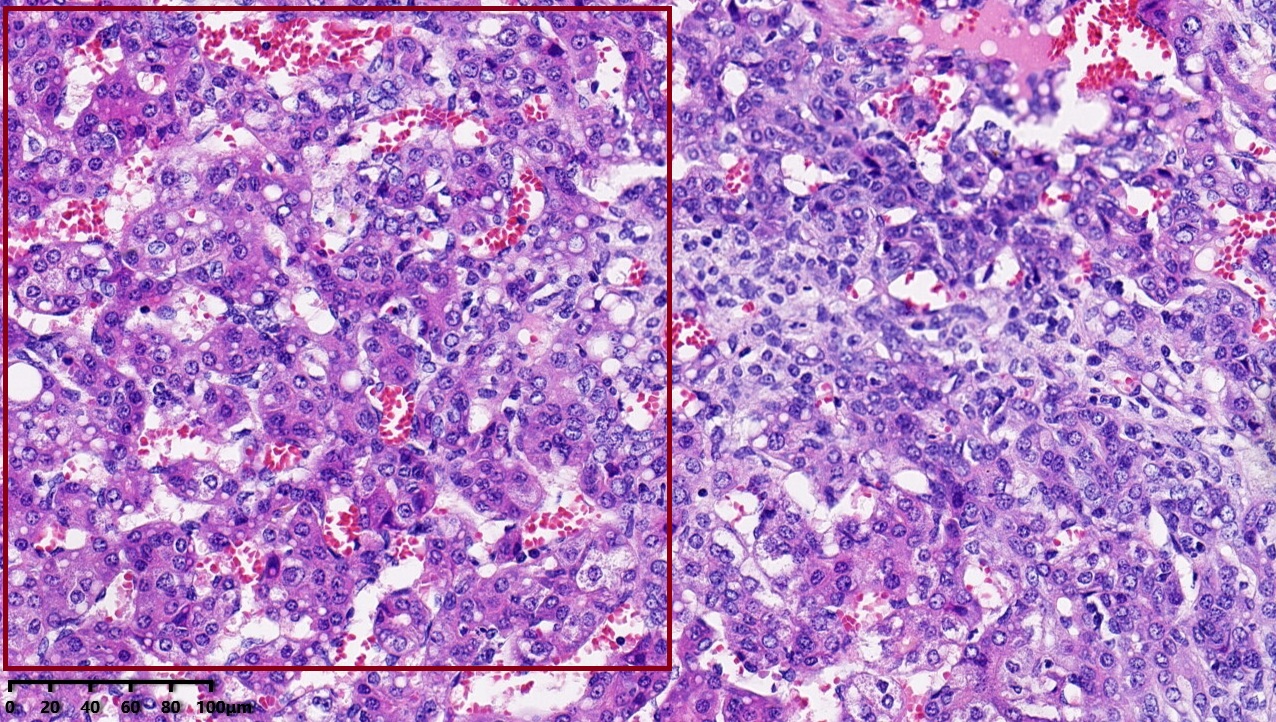

Supplement: Supplementary file 9 — Source data Fig. 7 [file 44318_2024_353_MOESM9_ESM.zip › Figure 7/7H/18590 HE 20X.jpg]

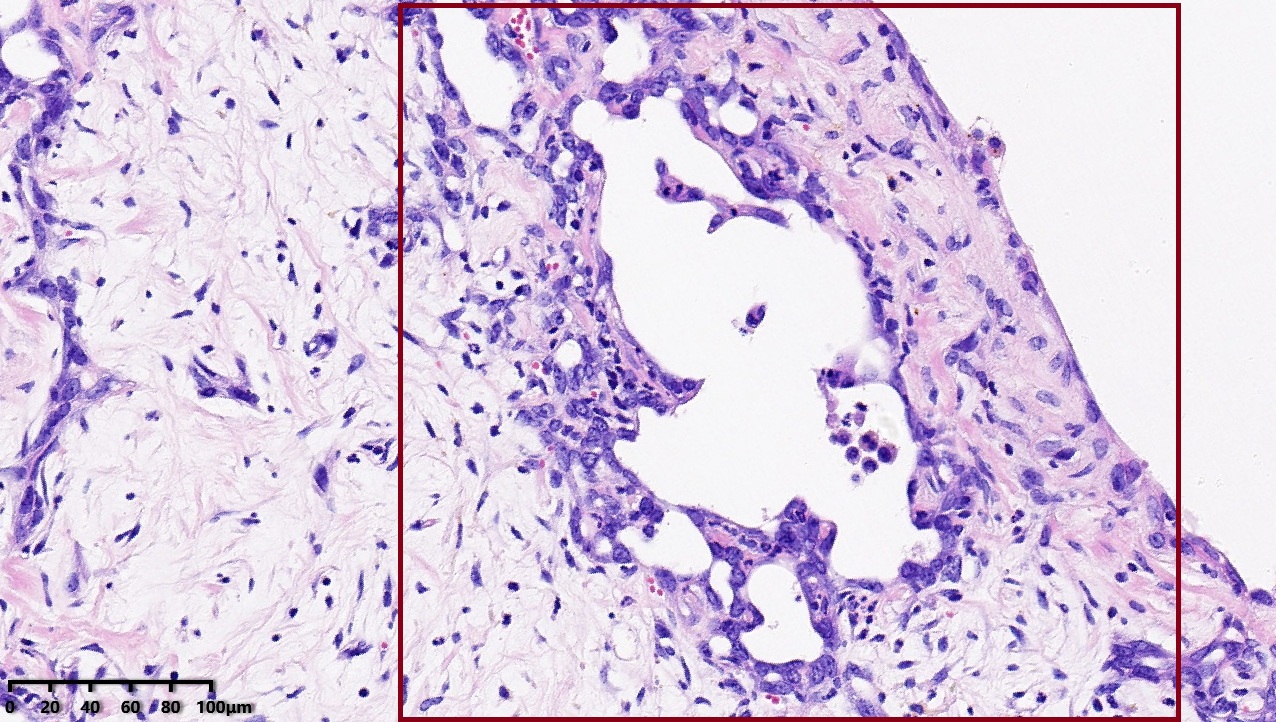

Supplement: Supplementary file 9 — Source data Fig. 7 [file 44318_2024_353_MOESM9_ESM.zip › Figure 7/7H/19080 HE 20X.jpg]

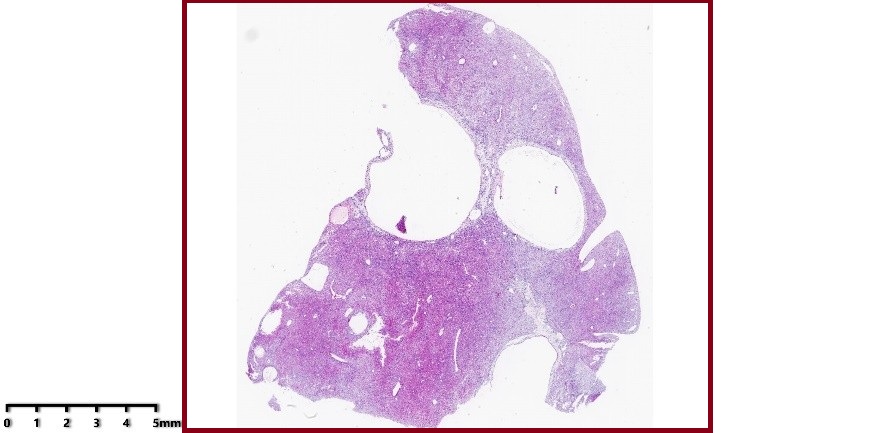

Supplement: Supplementary file 9 — Source data Fig. 7 [file 44318_2024_353_MOESM9_ESM.zip › Figure 7/7H/KO HE.jpg]

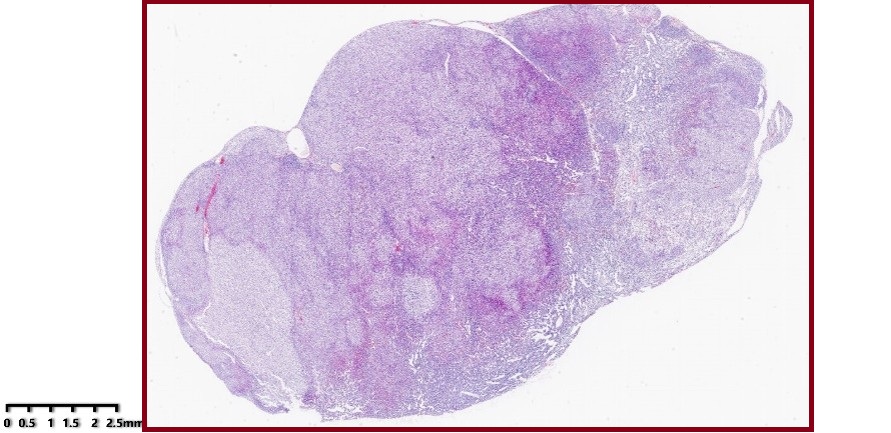

Supplement: Supplementary file 9 — Source data Fig. 7 [file 44318_2024_353_MOESM9_ESM.zip › Figure 7/7H/WT HE.jpg]

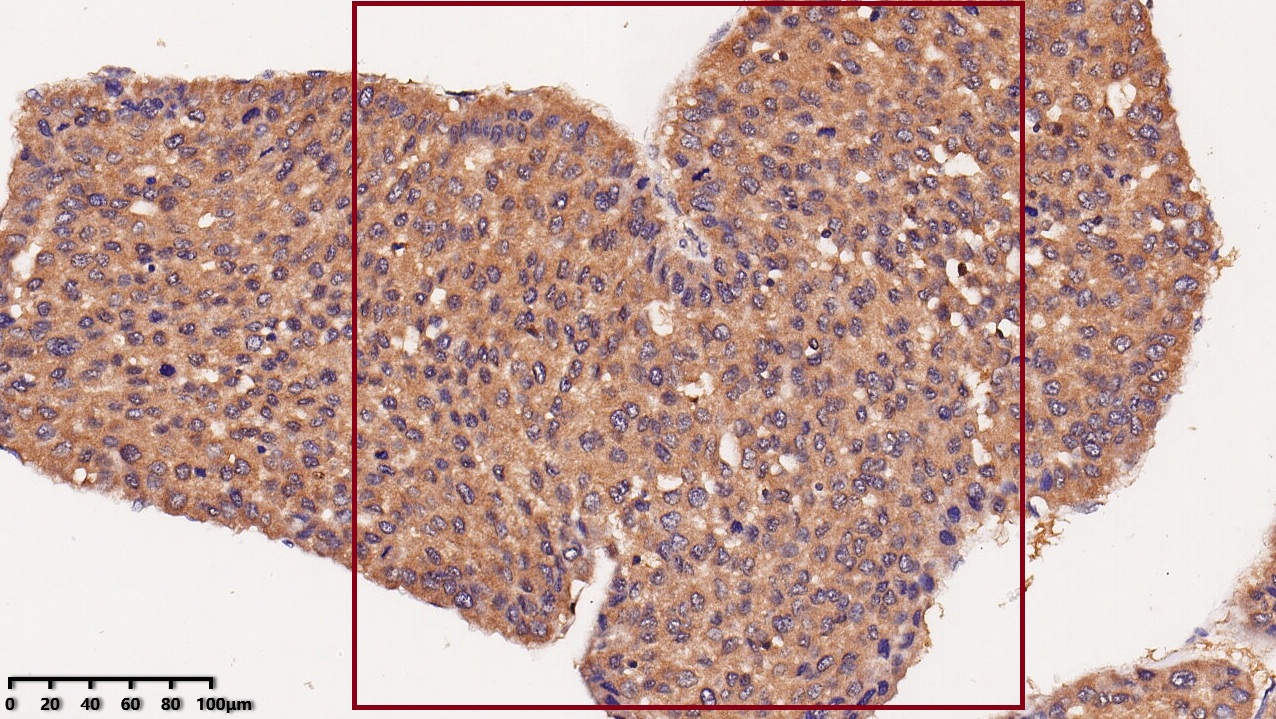

Supplement: Supplementary file 9 — Source data Fig. 7 [file 44318_2024_353_MOESM9_ESM.zip › Figure 7/7K/UBE2F High insert.jpg]

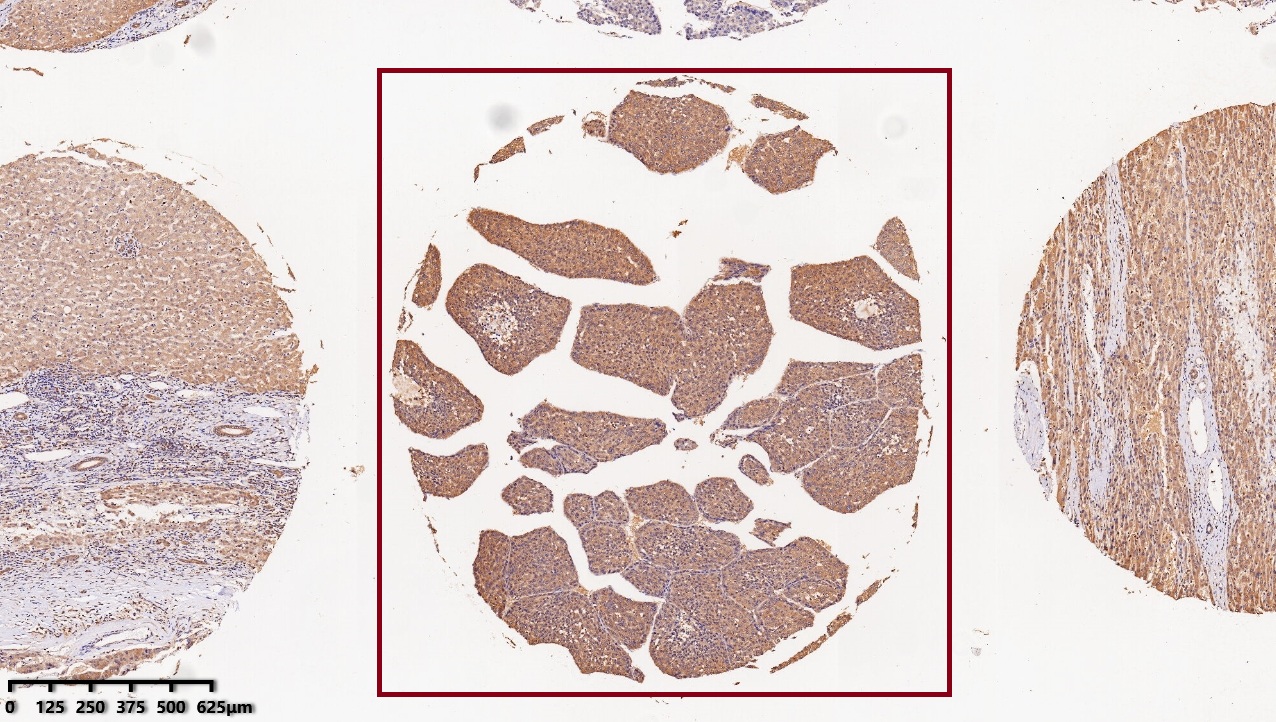

Supplement: Supplementary file 9 — Source data Fig. 7 [file 44318_2024_353_MOESM9_ESM.zip › Figure 7/7K/UBE2F High.jpg]

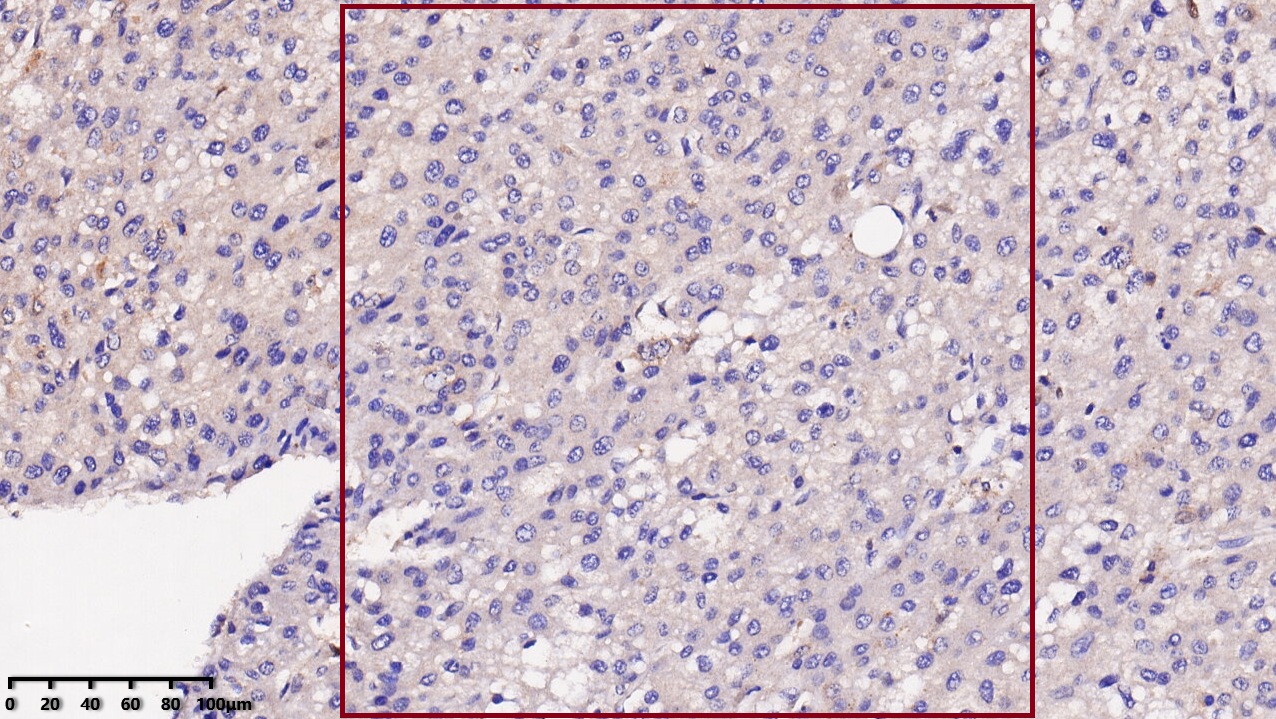

Supplement: Supplementary file 9 — Source data Fig. 7 [file 44318_2024_353_MOESM9_ESM.zip › Figure 7/7K/UBE2F low insert.jpg]

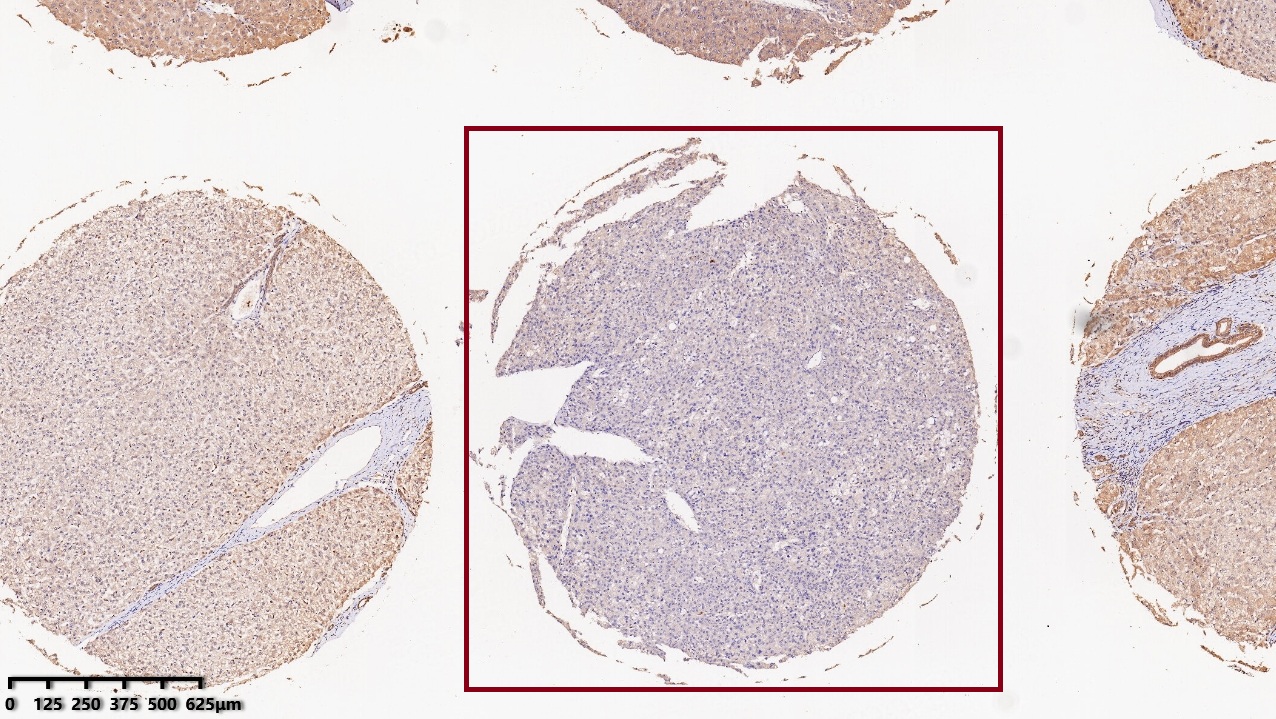

Supplement: Supplementary file 9 — Source data Fig. 7 [file 44318_2024_353_MOESM9_ESM.zip › Figure 7/7K/UBE2F low.jpg]
