# Supplementary figures and images for: RHEB neddylation by the UBE2F-SAG axis enhances mTORC1 activity and aggravates liver tumorigenesis (part 2 of 3)
Source: EMBO J. 2025 Jan 6;44(4):1185–219. doi: 10.1038/s44318-024-00353-5 (PMC11832924; doi:10.1038/s44318-024-00353-5)

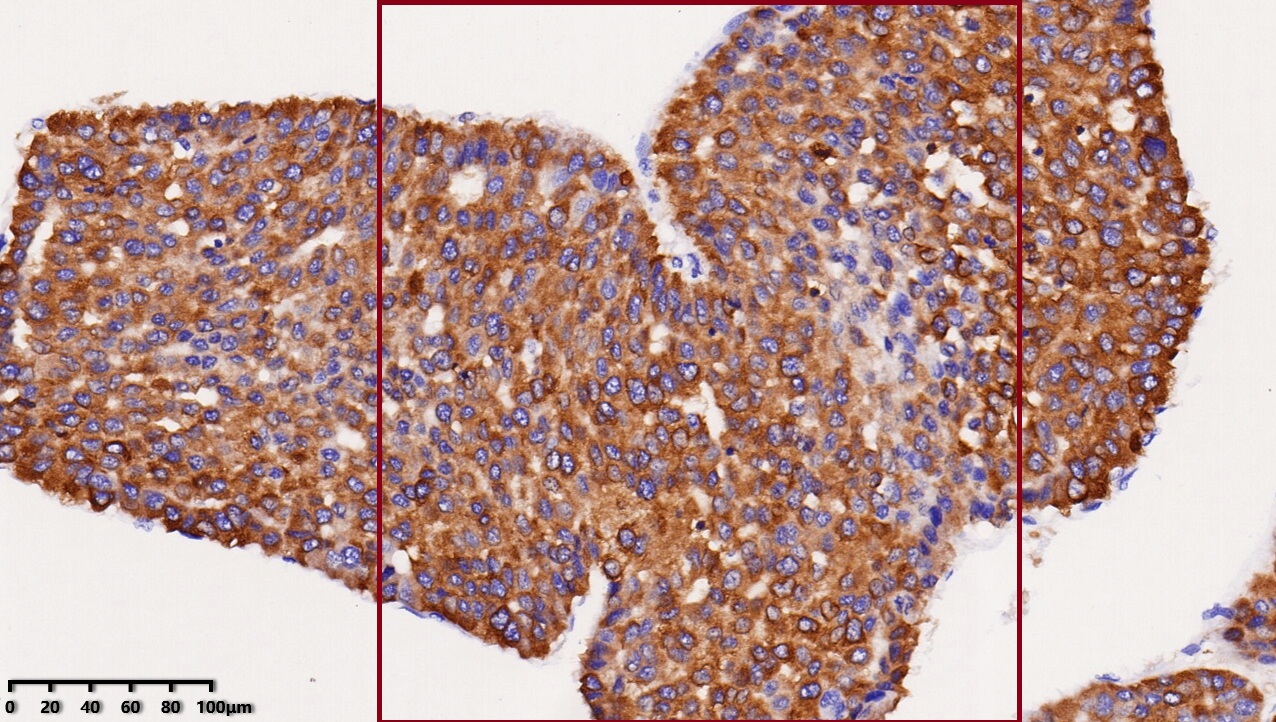

Supplement: Supplementary file 9 — Source data Fig. 7 [file 44318_2024_353_MOESM9_ESM.zip › Figure 7/7K/p-S6 High insert.jpg]

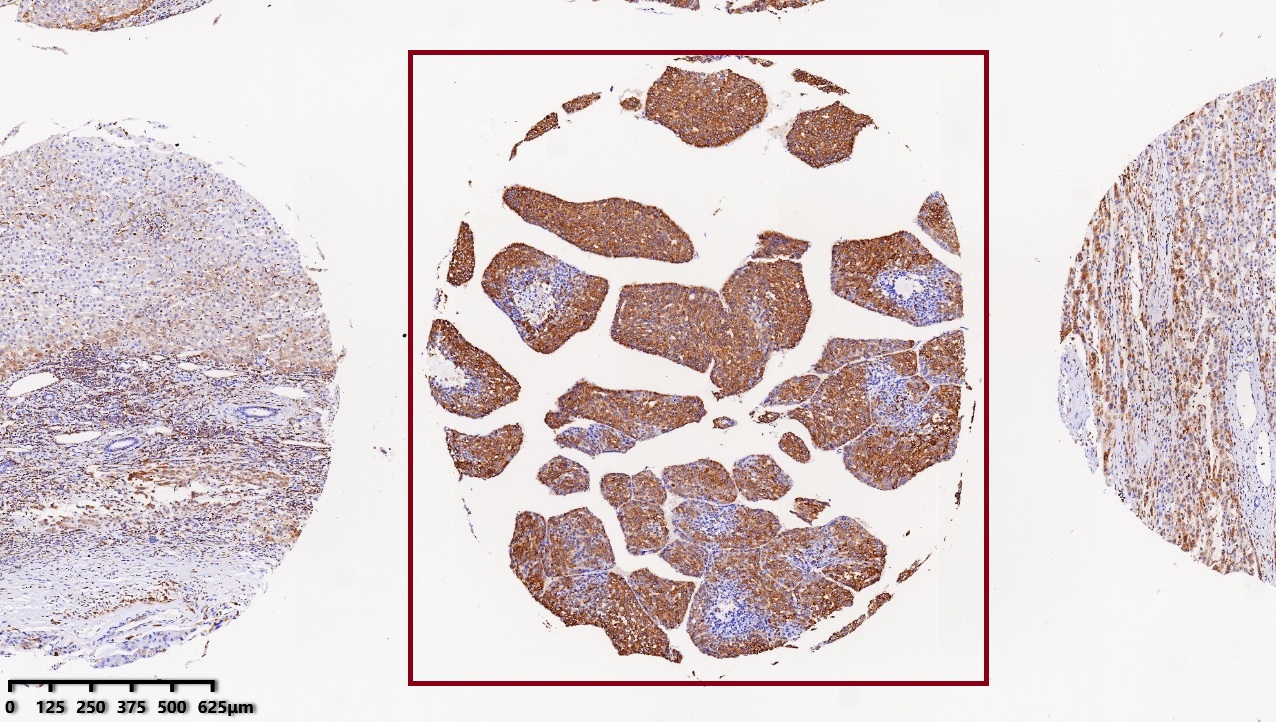

Supplement: Supplementary file 9 — Source data Fig. 7 [file 44318_2024_353_MOESM9_ESM.zip › Figure 7/7K/p-S6 High.jpg]

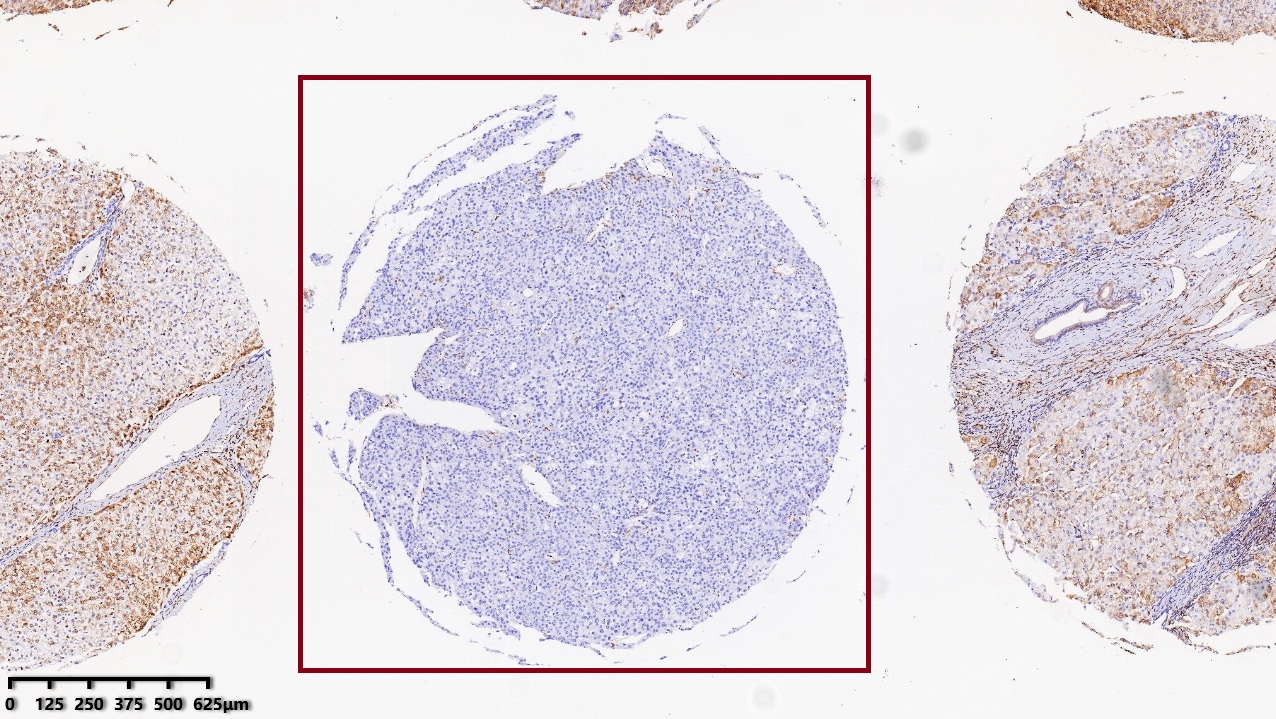

Supplement: Supplementary file 9 — Source data Fig. 7 [file 44318_2024_353_MOESM9_ESM.zip › Figure 7/7K/p-S6 Low.jpg]

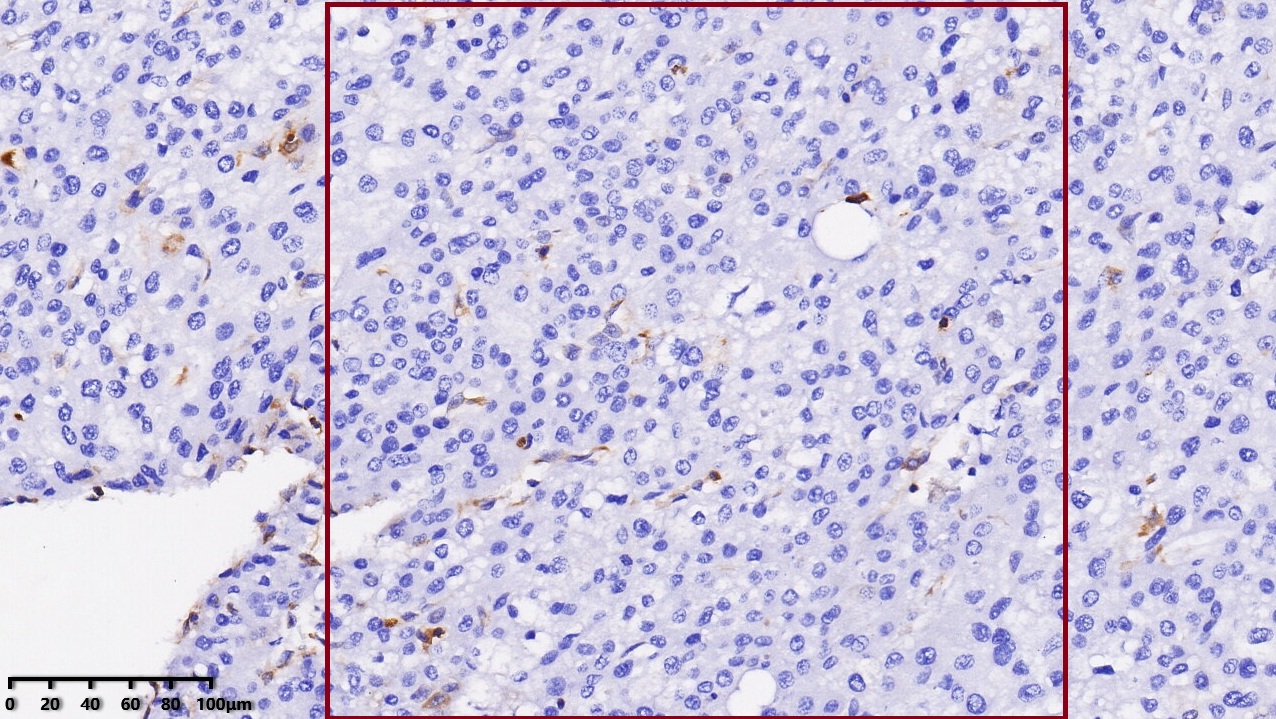

Supplement: Supplementary file 9 — Source data Fig. 7 [file 44318_2024_353_MOESM9_ESM.zip › Figure 7/7K/p-S6 low insert.jpg]

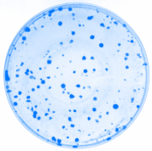

Supplement: Supplementary file 10 — Figure EV1 part 1 Source Data [file 44318_2024_353_MOESM10_ESM.zip › 1B/shGFP.png]

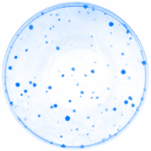

Supplement: Supplementary file 10 — Figure EV1 part 1 Source Data [file 44318_2024_353_MOESM10_ESM.zip › 1B/shUBE2F-1.png]

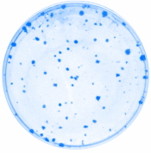

Supplement: Supplementary file 10 — Figure EV1 part 1 Source Data [file 44318_2024_353_MOESM10_ESM.zip › 1B/shUBE2F-2.png]

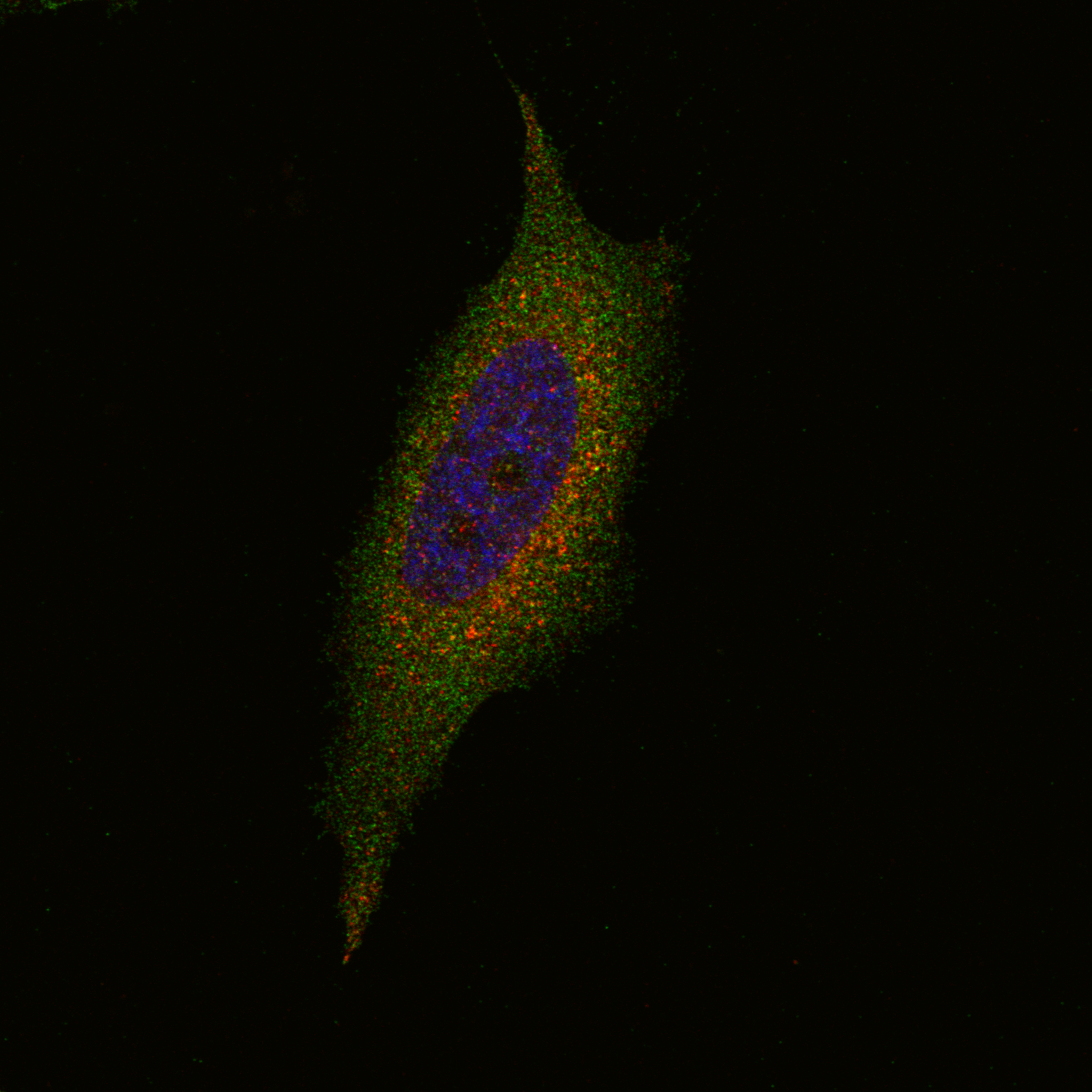

Supplement: Supplementary file 13 — Figure EV3 Source Data [file 44318_2024_353_MOESM13_ESM.zip › EVFigure 3/3I/HEP3B siCtrl/Project_sinc-5x1.6.tif]

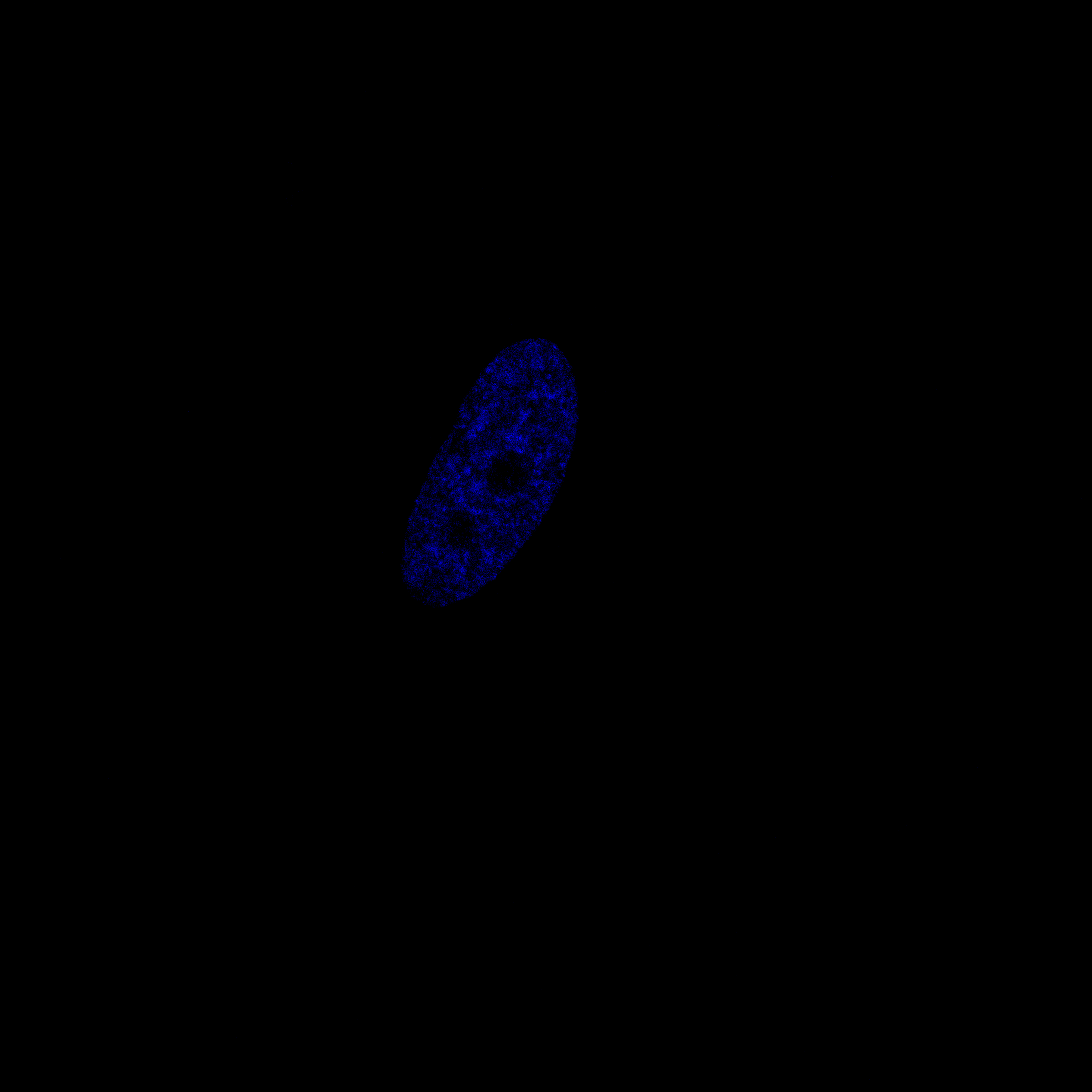

Supplement: Supplementary file 13 — Figure EV3 Source Data [file 44318_2024_353_MOESM13_ESM.zip › EVFigure 3/3I/HEP3B siCtrl/Project_sinc-5x1.6_ch00.tif]

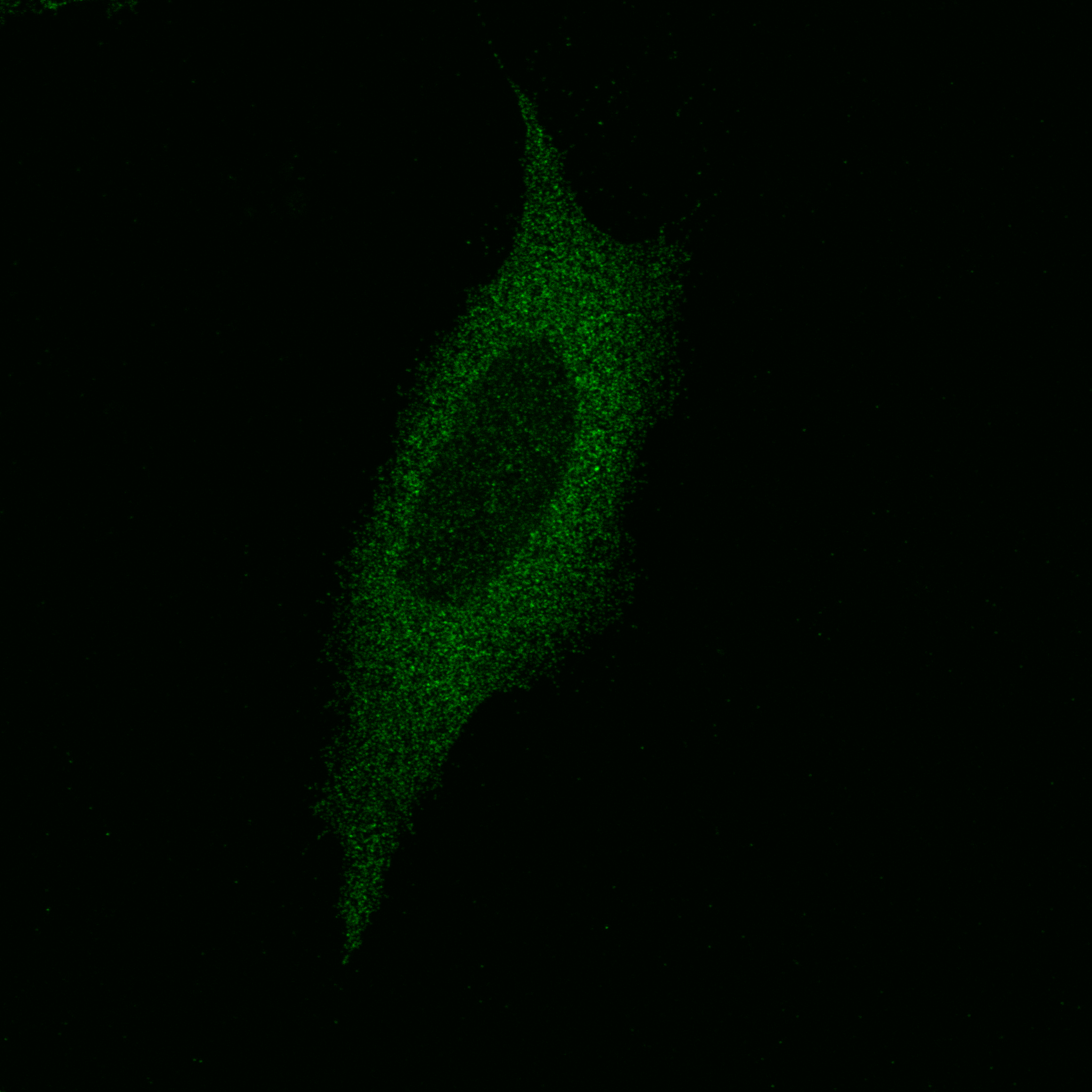

Supplement: Supplementary file 13 — Figure EV3 Source Data [file 44318_2024_353_MOESM13_ESM.zip › EVFigure 3/3I/HEP3B siCtrl/Project_sinc-5x1.6_ch01.tif]

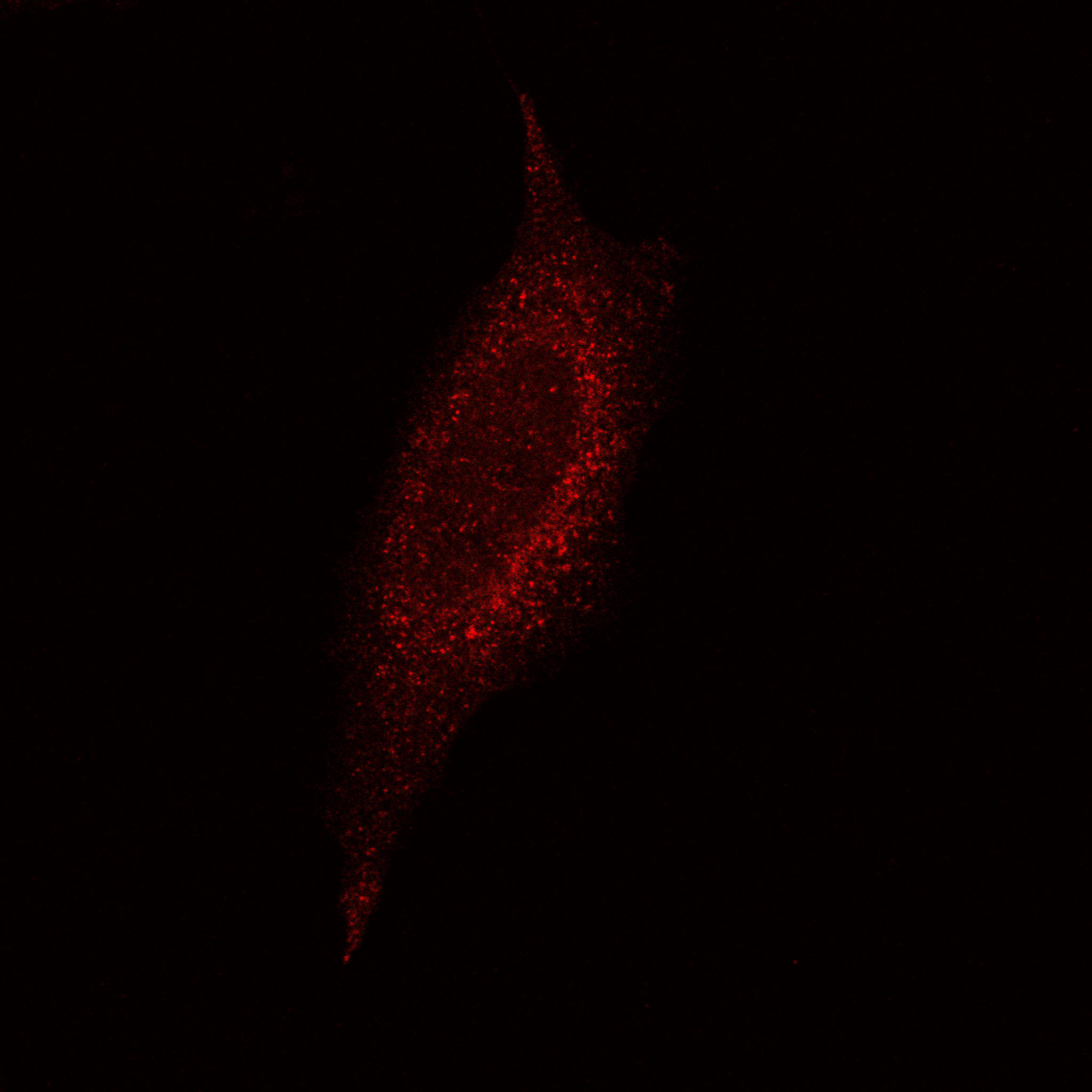

Supplement: Supplementary file 13 — Figure EV3 Source Data [file 44318_2024_353_MOESM13_ESM.zip › EVFigure 3/3I/HEP3B siCtrl/Project_sinc-5x1.6_ch02.tif]

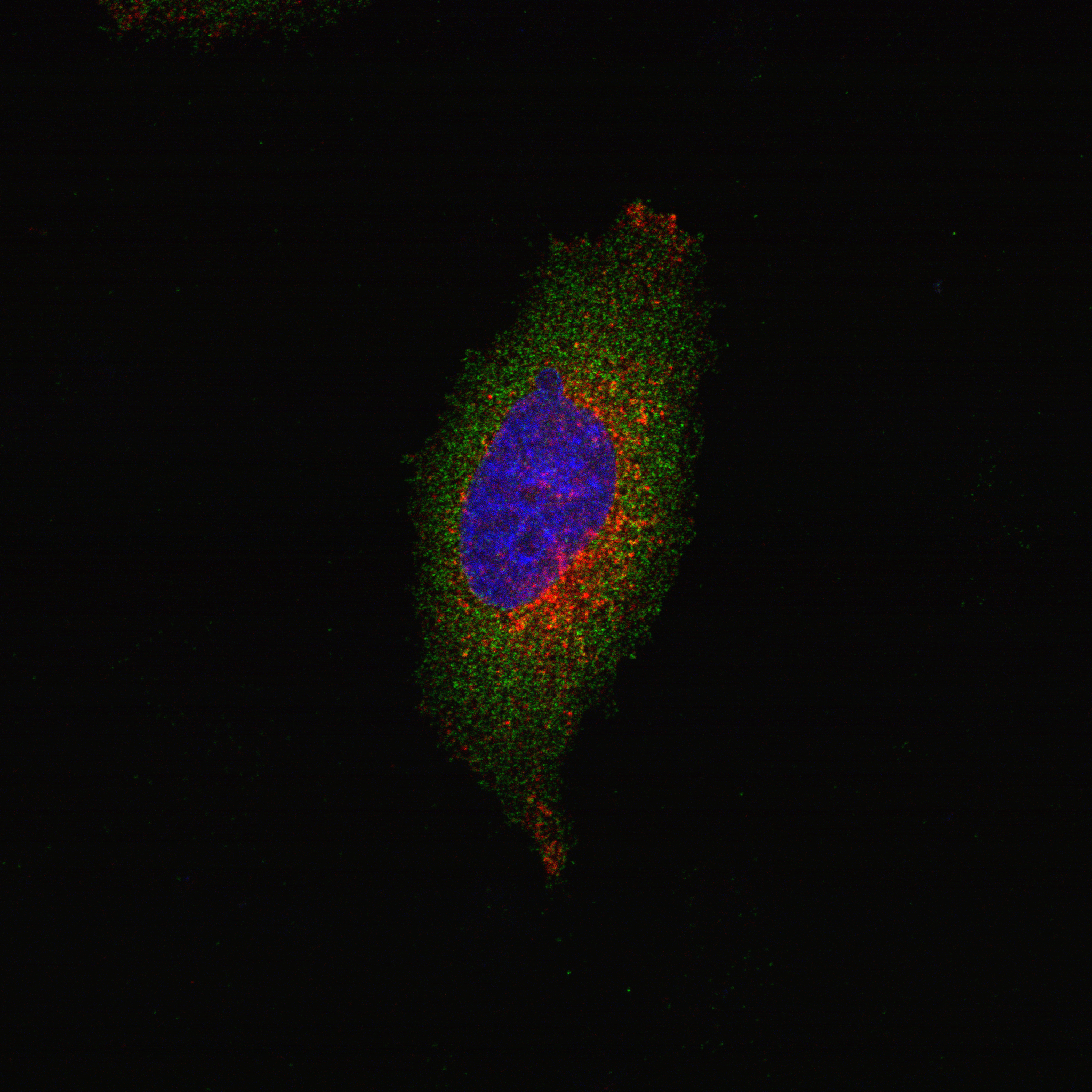

Supplement: Supplementary file 13 — Figure EV3 Source Data [file 44318_2024_353_MOESM13_ESM.zip › EVFigure 3/3I/HEP3B siSAG/Project_SISAG-1X1.5.tif]

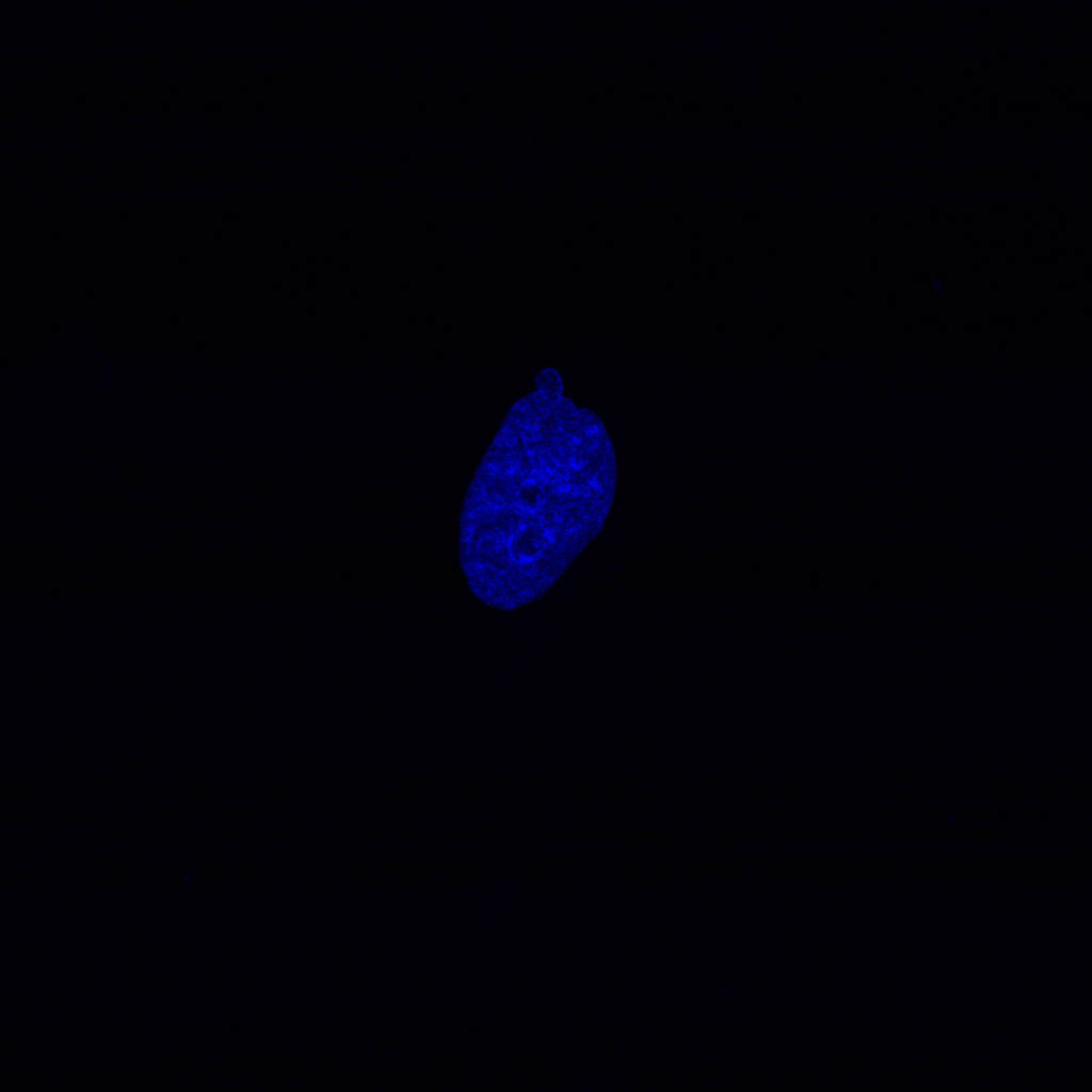

Supplement: Supplementary file 13 — Figure EV3 Source Data [file 44318_2024_353_MOESM13_ESM.zip › EVFigure 3/3I/HEP3B siSAG/Project_SISAG-1X1.5_ch00.tif]

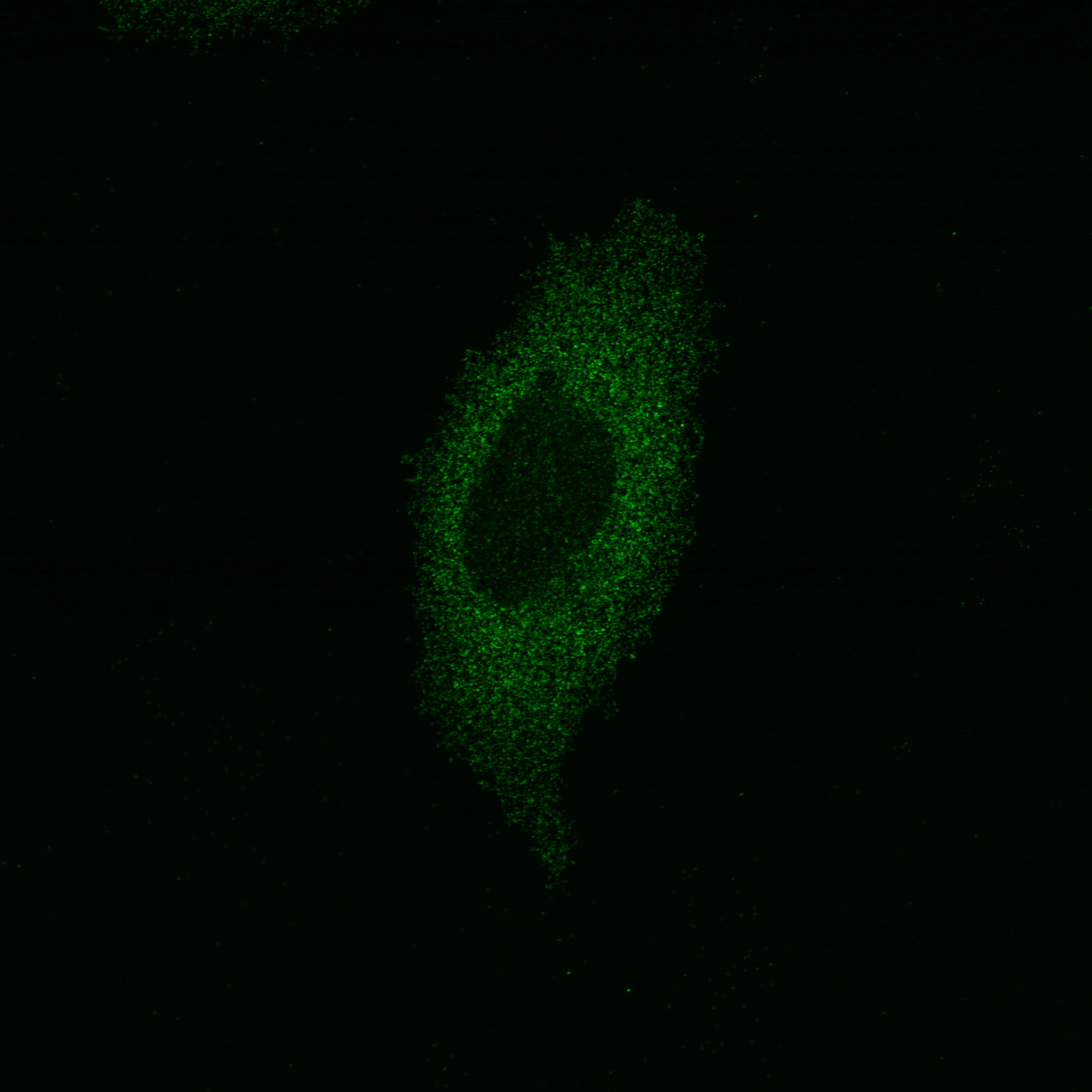

Supplement: Supplementary file 13 — Figure EV3 Source Data [file 44318_2024_353_MOESM13_ESM.zip › EVFigure 3/3I/HEP3B siSAG/Project_SISAG-1X1.5_ch01.tif]

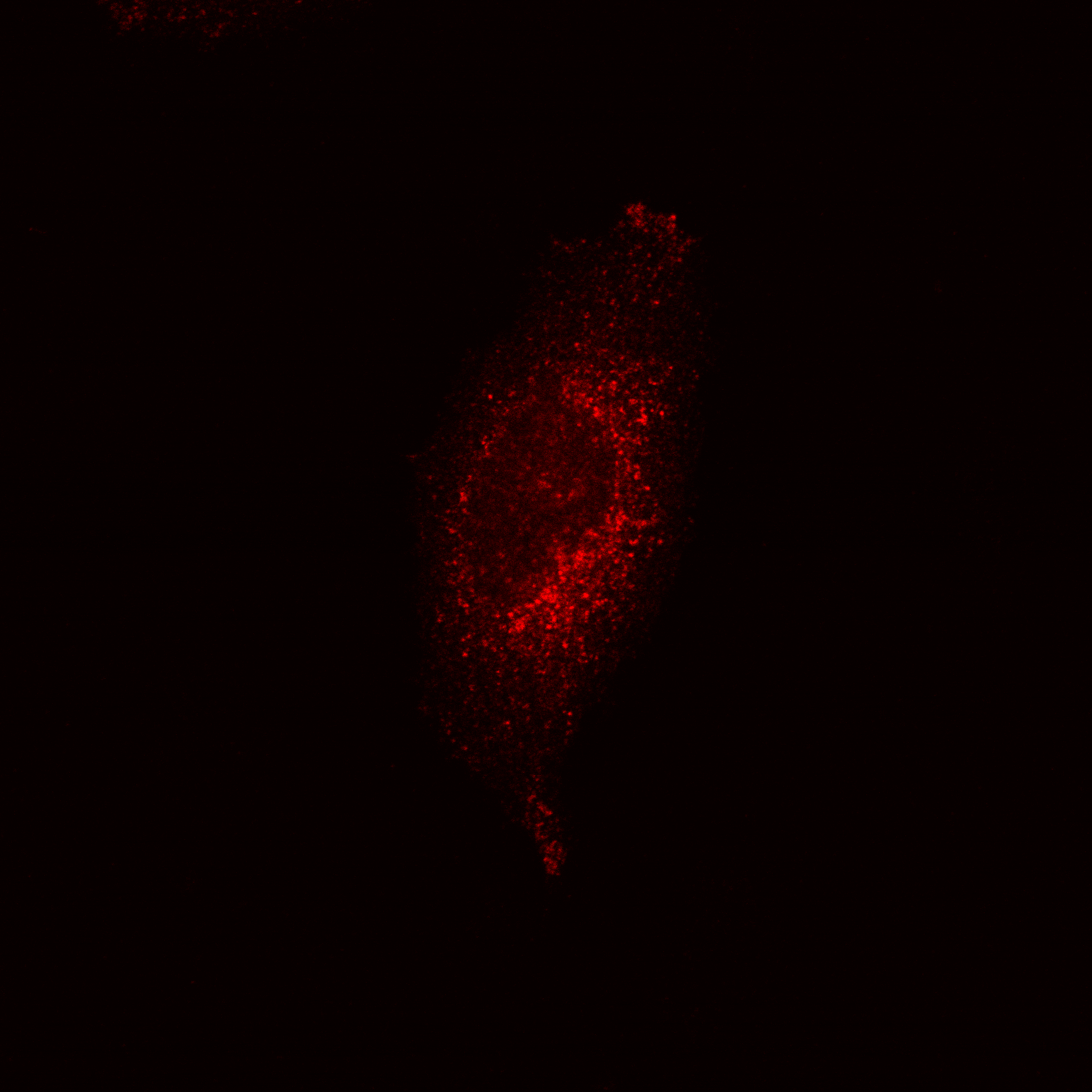

Supplement: Supplementary file 13 — Figure EV3 Source Data [file 44318_2024_353_MOESM13_ESM.zip › EVFigure 3/3I/HEP3B siSAG/Project_SISAG-1X1.5_ch02.tif]

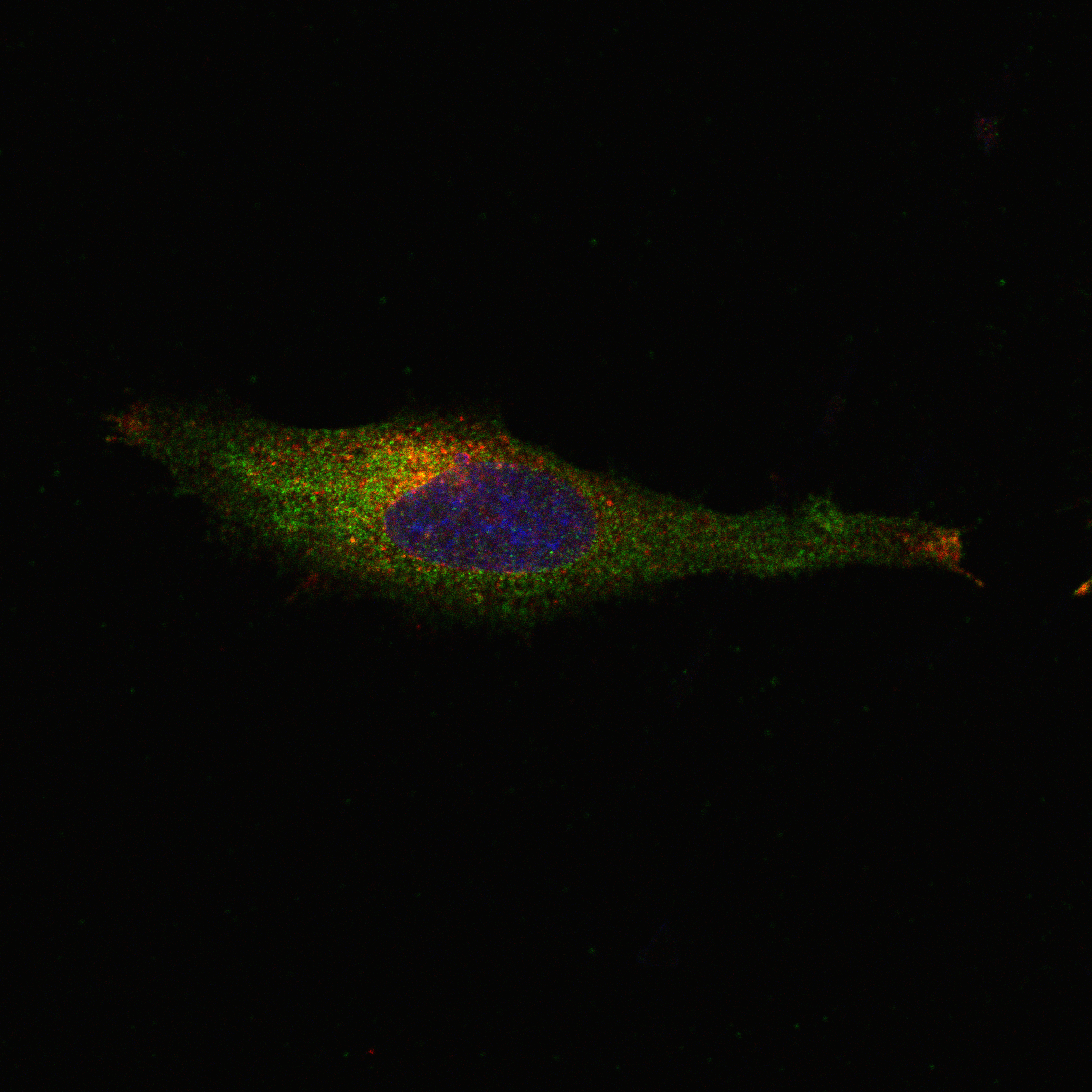

Supplement: Supplementary file 13 — Figure EV3 Source Data [file 44318_2024_353_MOESM13_ESM.zip › EVFigure 3/3I/HEP3B siUBE2F/Project_SI2F-2X1.5.tif]

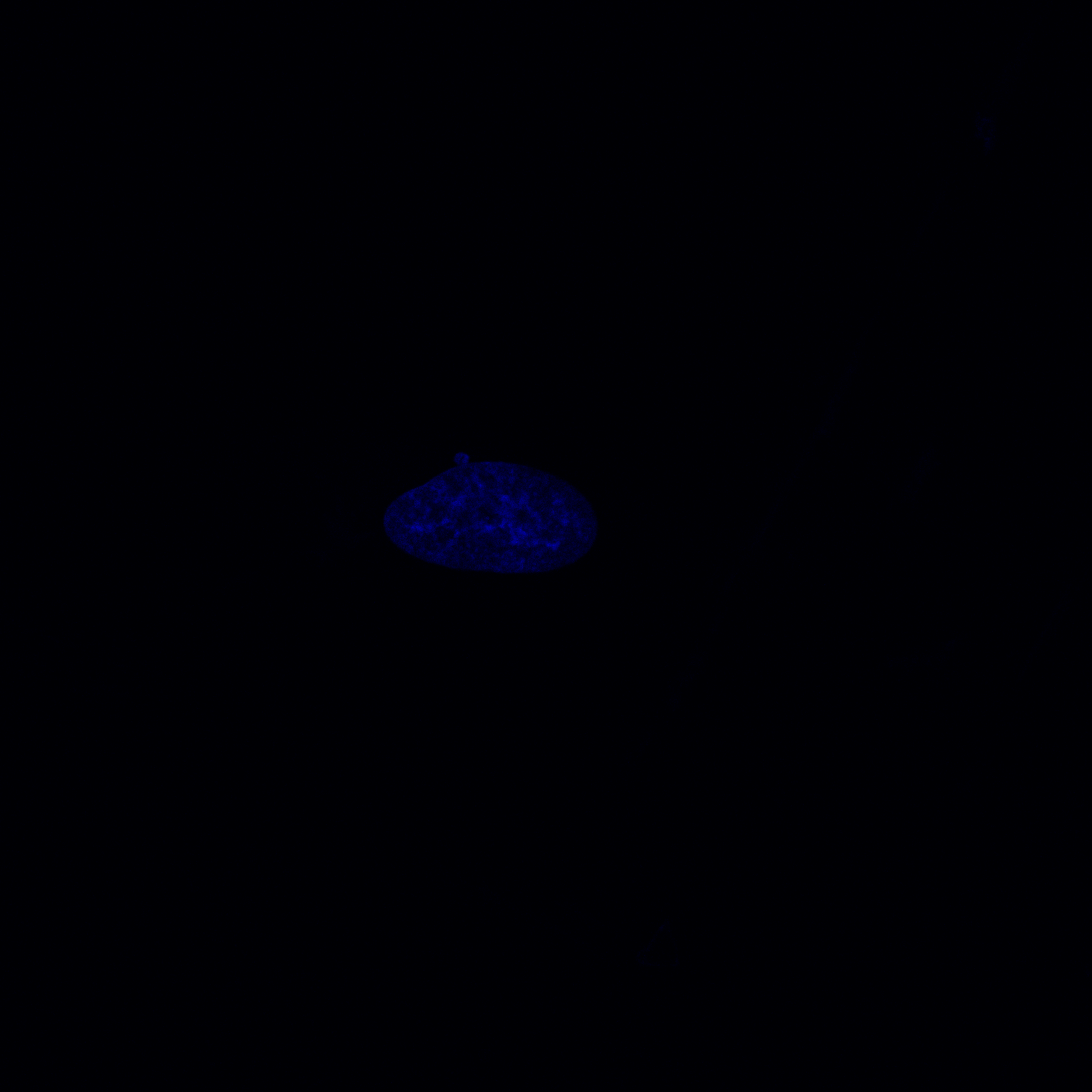

Supplement: Supplementary file 13 — Figure EV3 Source Data [file 44318_2024_353_MOESM13_ESM.zip › EVFigure 3/3I/HEP3B siUBE2F/Project_SI2F-2X1.5_ch00.tif]

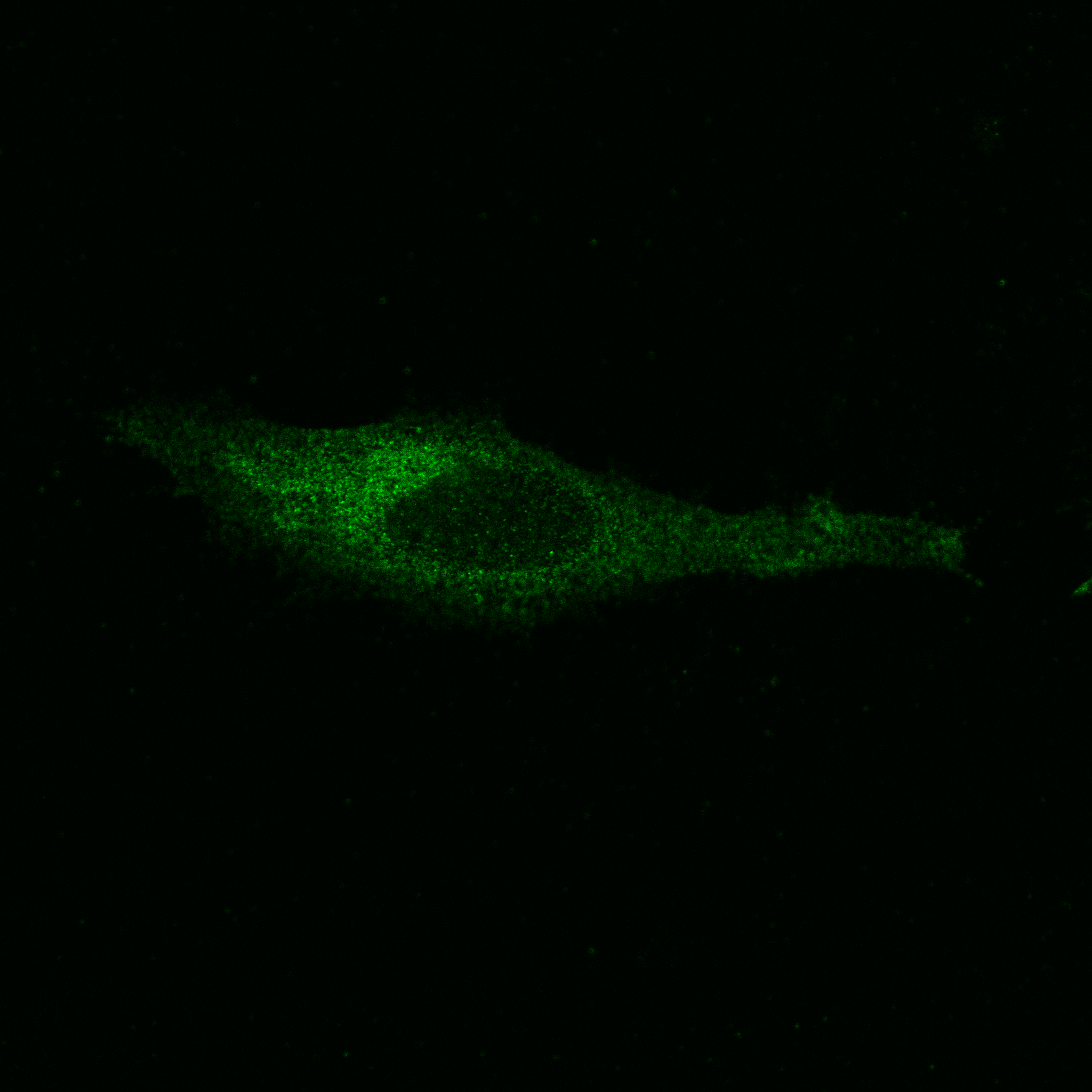

Supplement: Supplementary file 13 — Figure EV3 Source Data [file 44318_2024_353_MOESM13_ESM.zip › EVFigure 3/3I/HEP3B siUBE2F/Project_SI2F-2X1.5_ch01.tif]

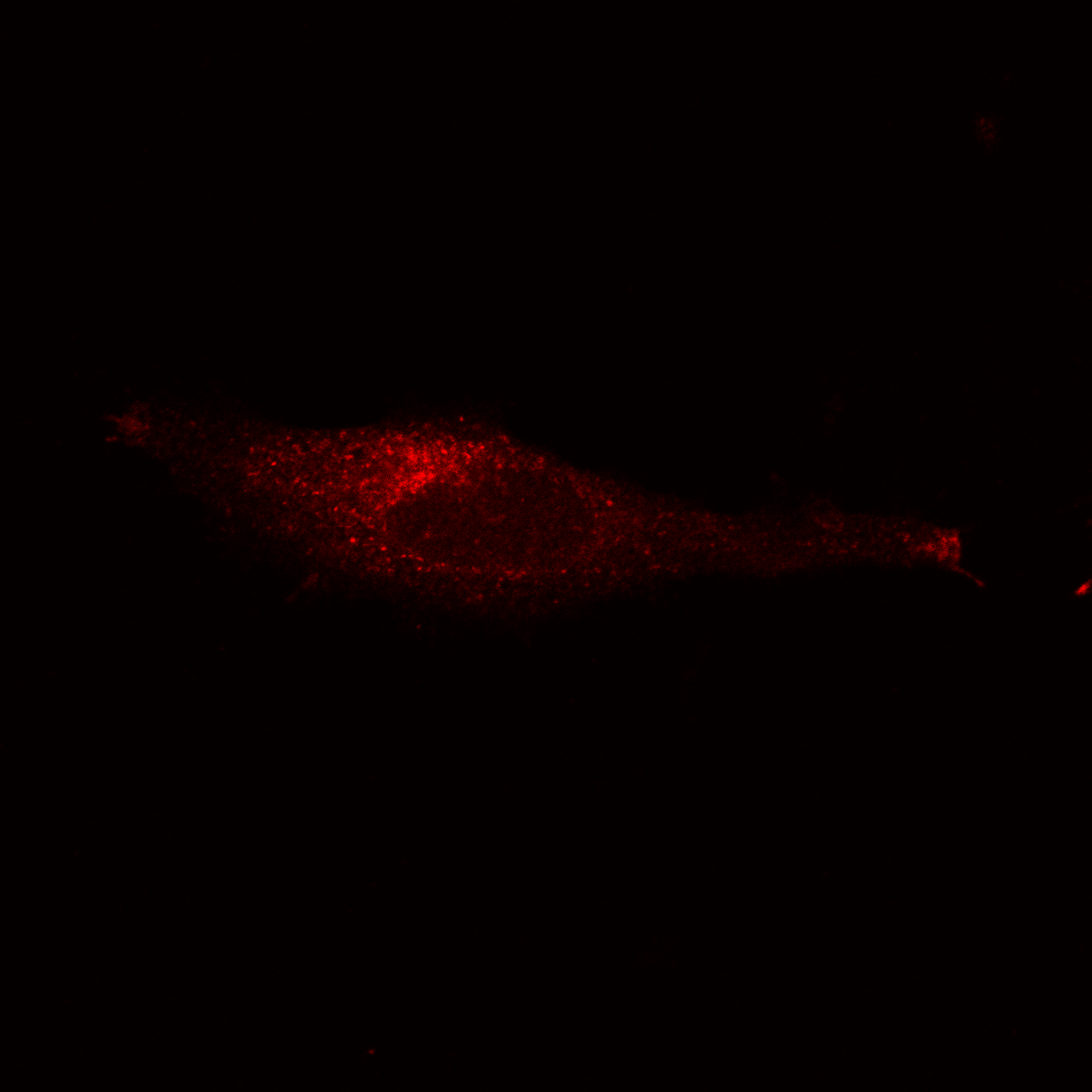

Supplement: Supplementary file 13 — Figure EV3 Source Data [file 44318_2024_353_MOESM13_ESM.zip › EVFigure 3/3I/HEP3B siUBE2F/Project_SI2F-2X1.5_ch02.tif]

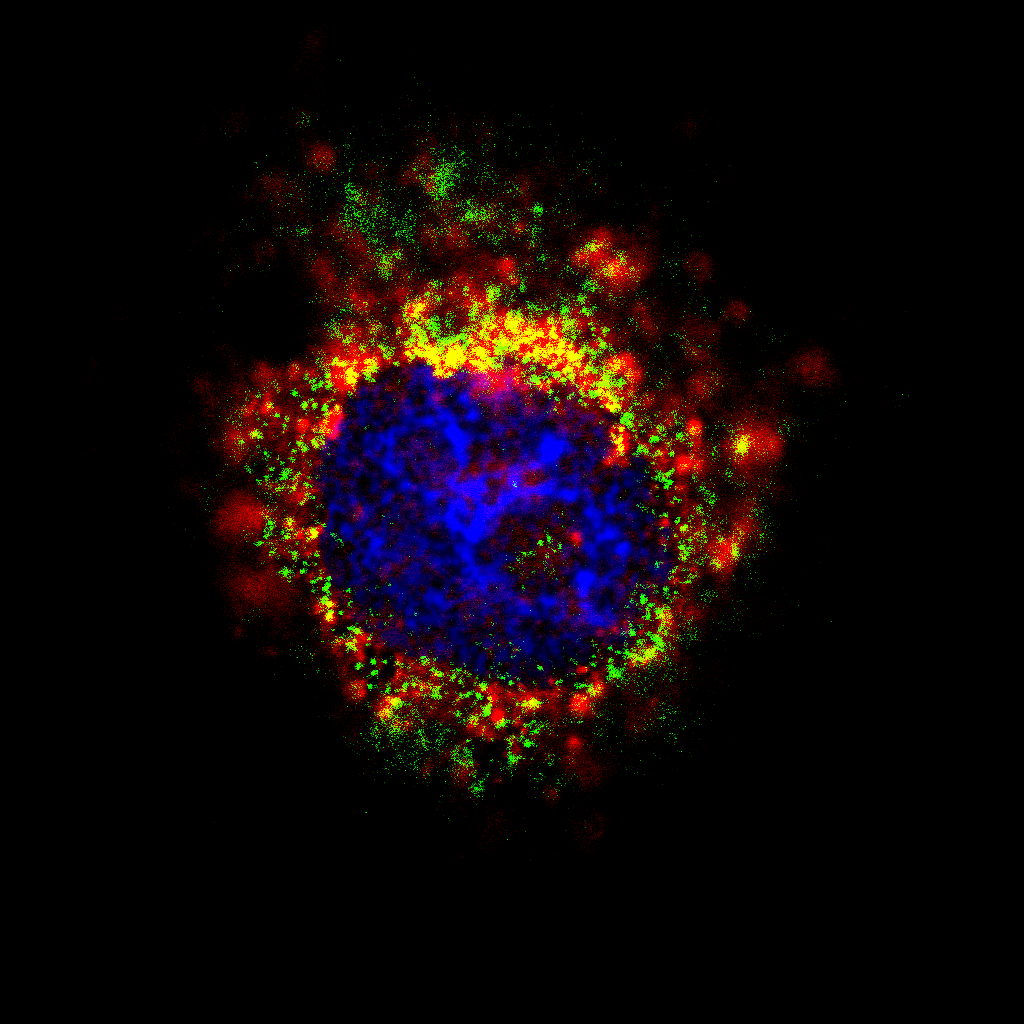

Supplement: Supplementary file 13 — Figure EV3 Source Data [file 44318_2024_353_MOESM13_ESM.zip › EVFigure 3/3J/PLCPRF5 SICTRL/Project_PP5SINCLAMP2MTOR60X3.5-4.tif]

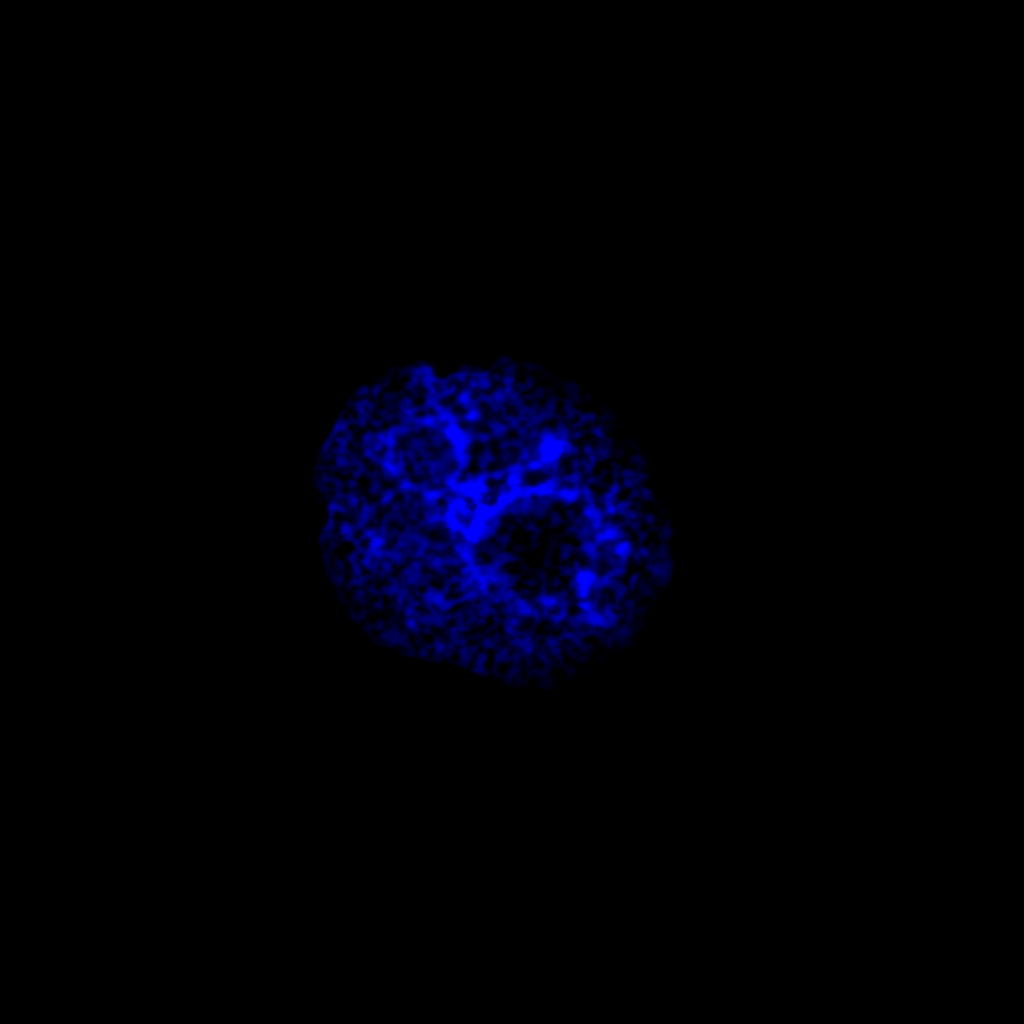

Supplement: Supplementary file 13 — Figure EV3 Source Data [file 44318_2024_353_MOESM13_ESM.zip › EVFigure 3/3J/PLCPRF5 SICTRL/Project_PP5SINCLAMP2MTOR60X3.5-4_ch00.tif]

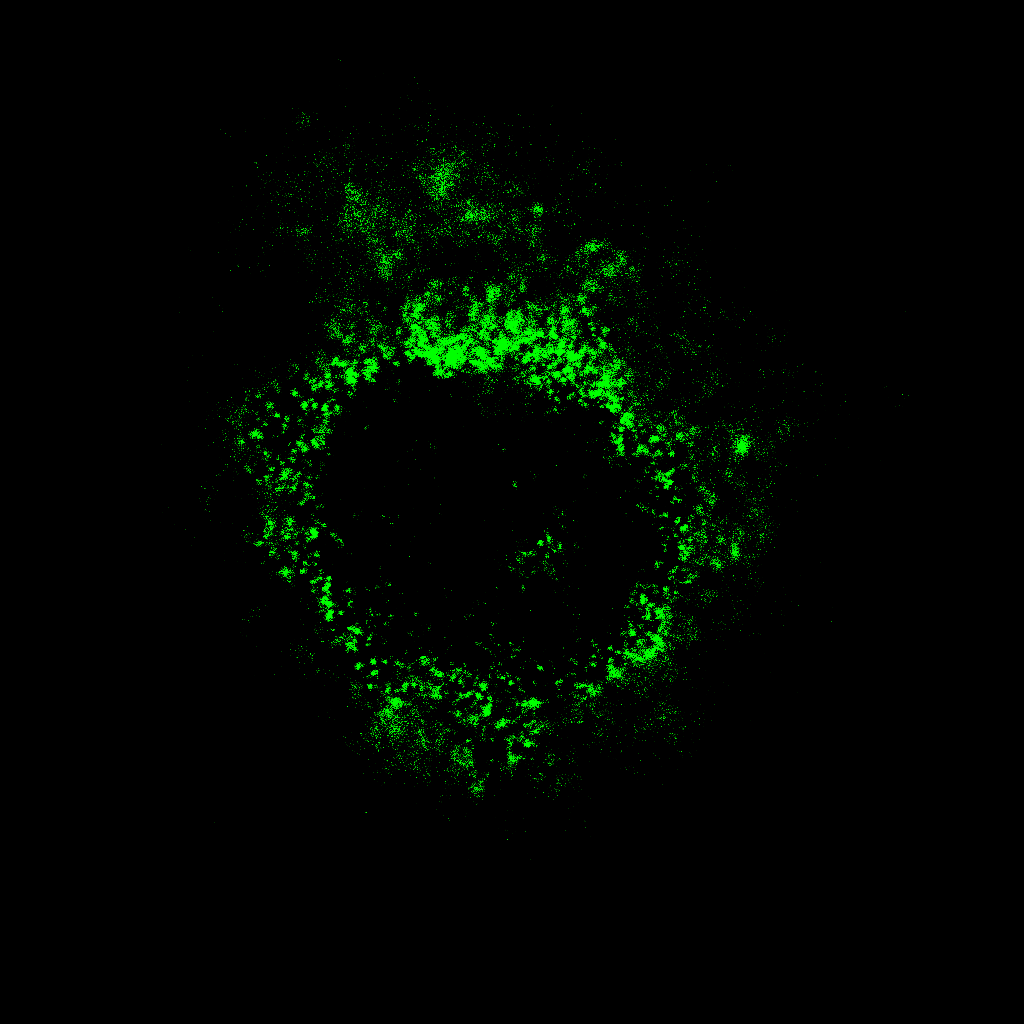

Supplement: Supplementary file 13 — Figure EV3 Source Data [file 44318_2024_353_MOESM13_ESM.zip › EVFigure 3/3J/PLCPRF5 SICTRL/Project_PP5SINCLAMP2MTOR60X3.5-4_ch01.tif]

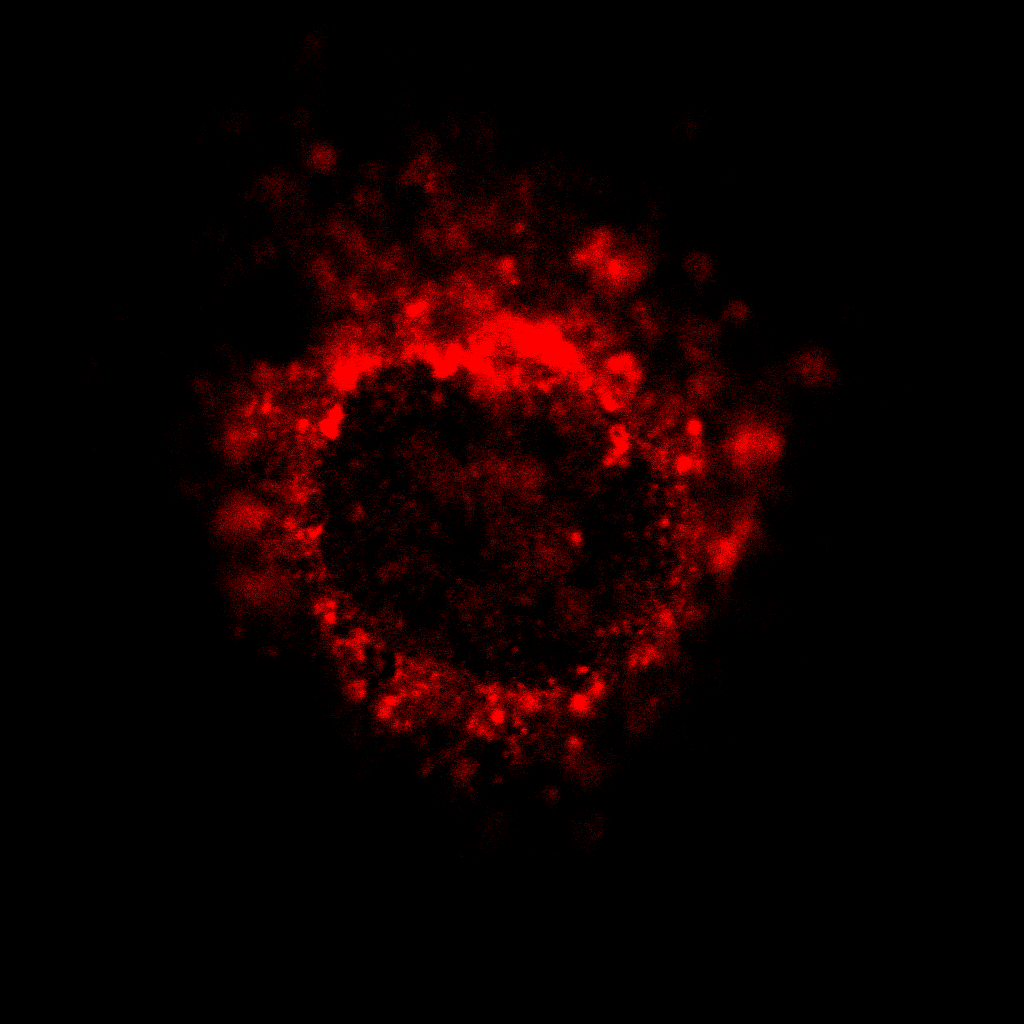

Supplement: Supplementary file 13 — Figure EV3 Source Data [file 44318_2024_353_MOESM13_ESM.zip › EVFigure 3/3J/PLCPRF5 SICTRL/Project_PP5SINCLAMP2MTOR60X3.5-4_ch02.tif]

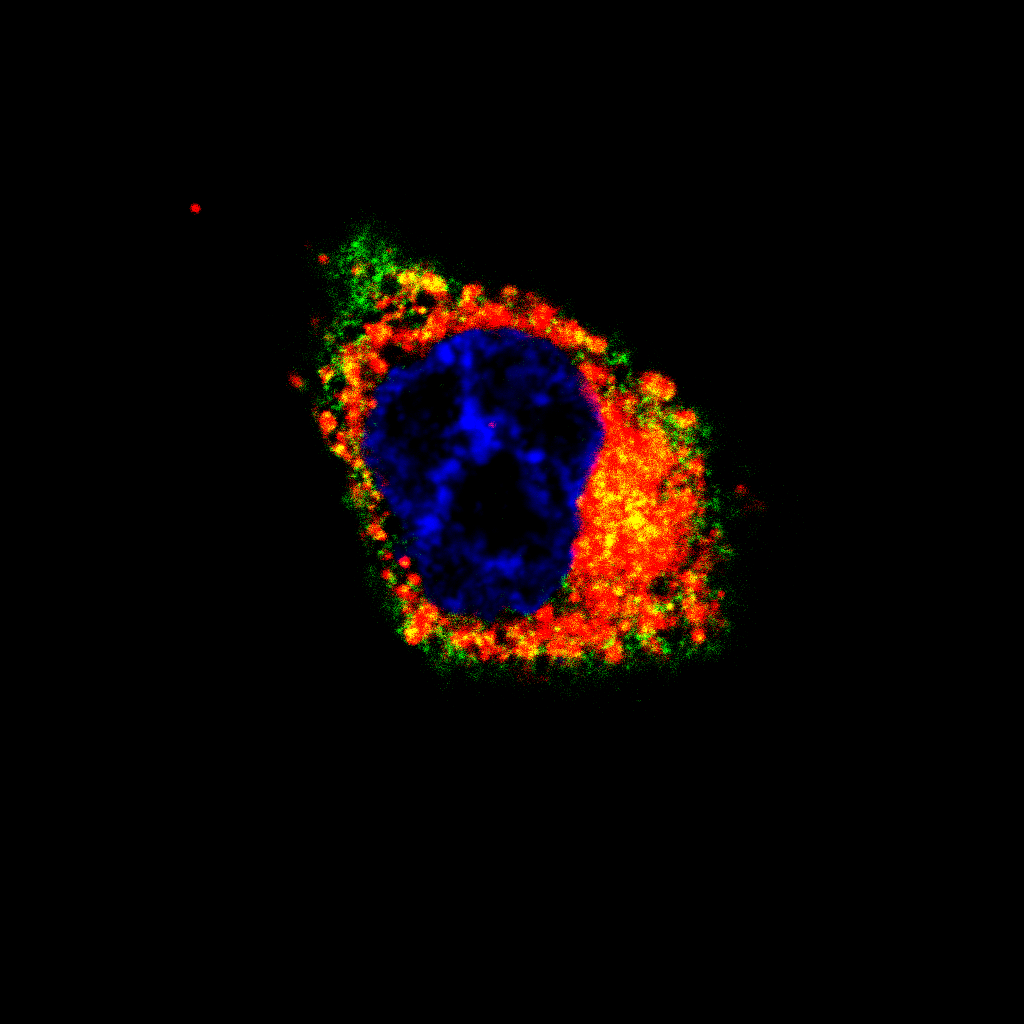

Supplement: Supplementary file 13 — Figure EV3 Source Data [file 44318_2024_353_MOESM13_ESM.zip › EVFigure 3/3J/PLCPRF5 SISAG/Project_PP5SISAGLAMP2MTOR60X3.5-2.tif]

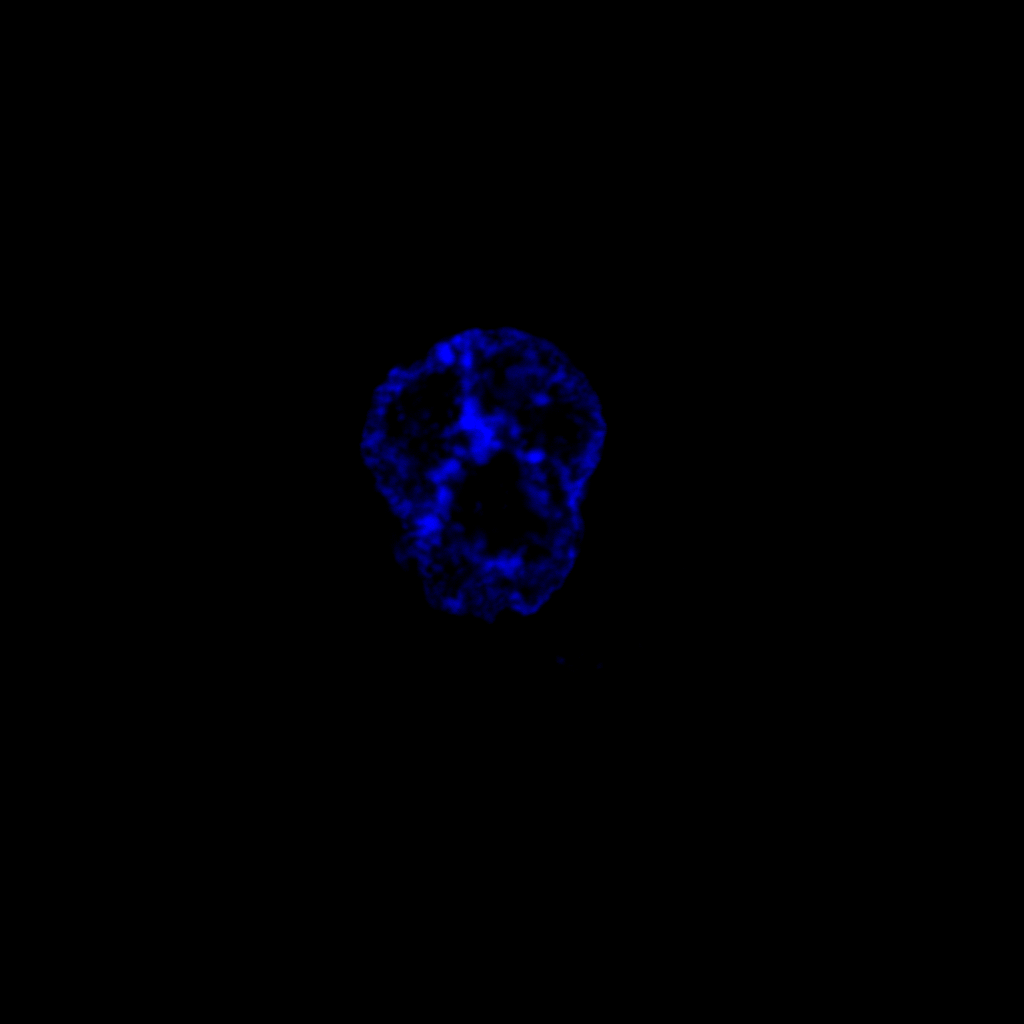

Supplement: Supplementary file 13 — Figure EV3 Source Data [file 44318_2024_353_MOESM13_ESM.zip › EVFigure 3/3J/PLCPRF5 SISAG/Project_PP5SISAGLAMP2MTOR60X3.5-2_ch00.tif]

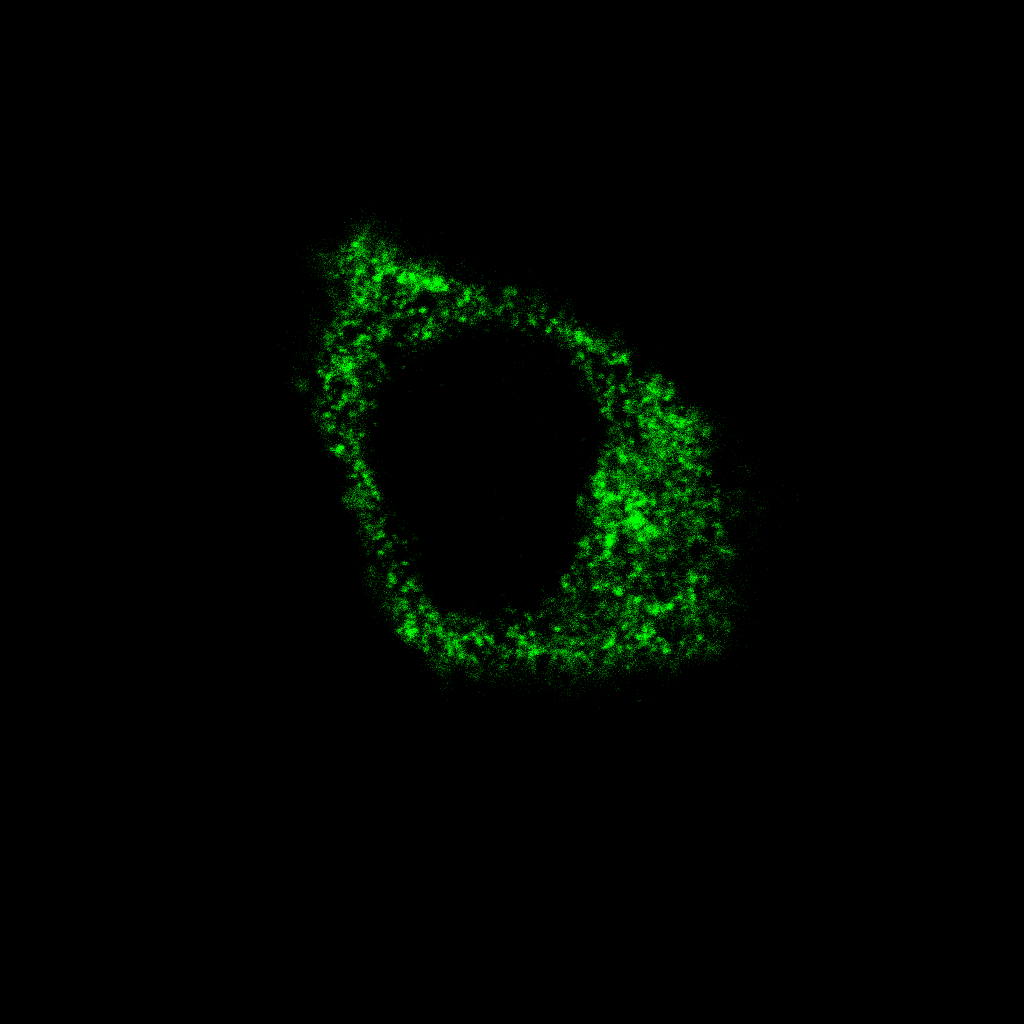

Supplement: Supplementary file 13 — Figure EV3 Source Data [file 44318_2024_353_MOESM13_ESM.zip › EVFigure 3/3J/PLCPRF5 SISAG/Project_PP5SISAGLAMP2MTOR60X3.5-2_ch01.tif]

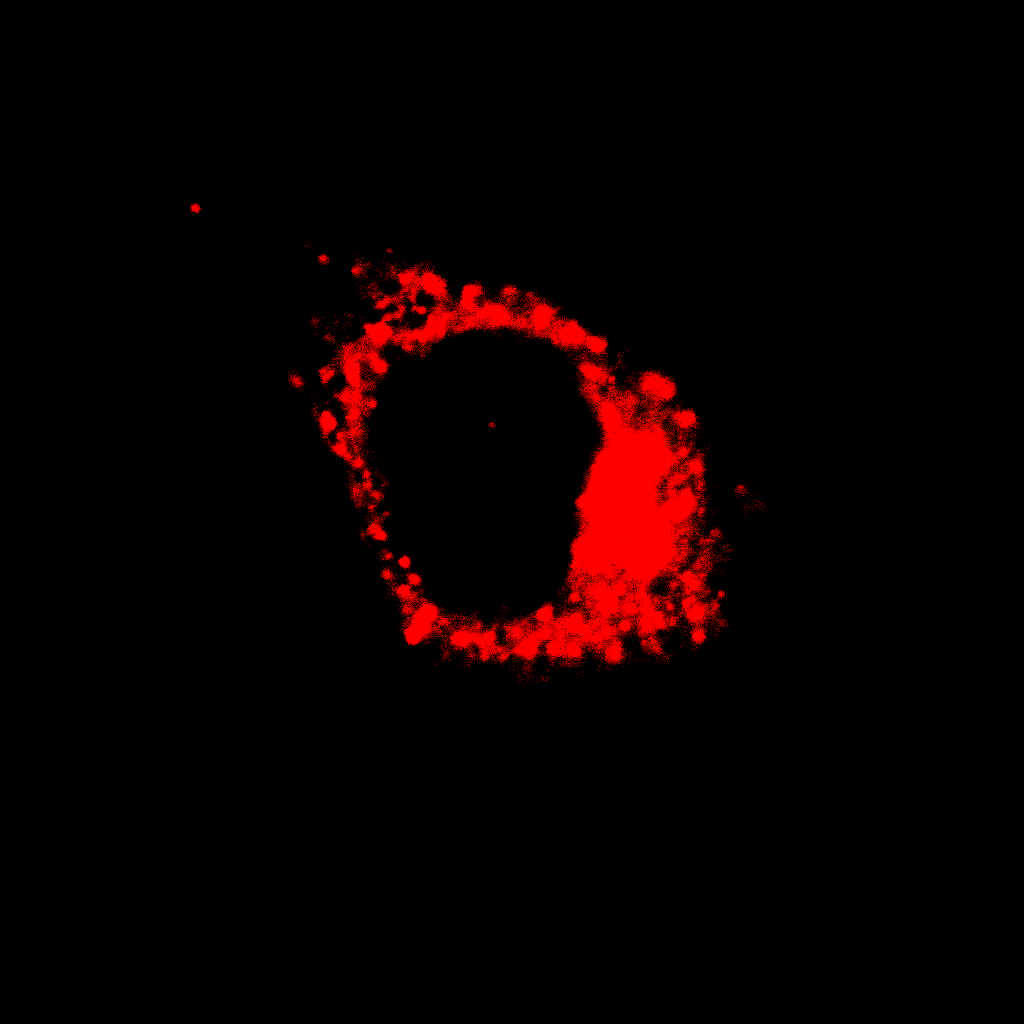

Supplement: Supplementary file 13 — Figure EV3 Source Data [file 44318_2024_353_MOESM13_ESM.zip › EVFigure 3/3J/PLCPRF5 SISAG/Project_PP5SISAGLAMP2MTOR60X3.5-2_ch02.tif]

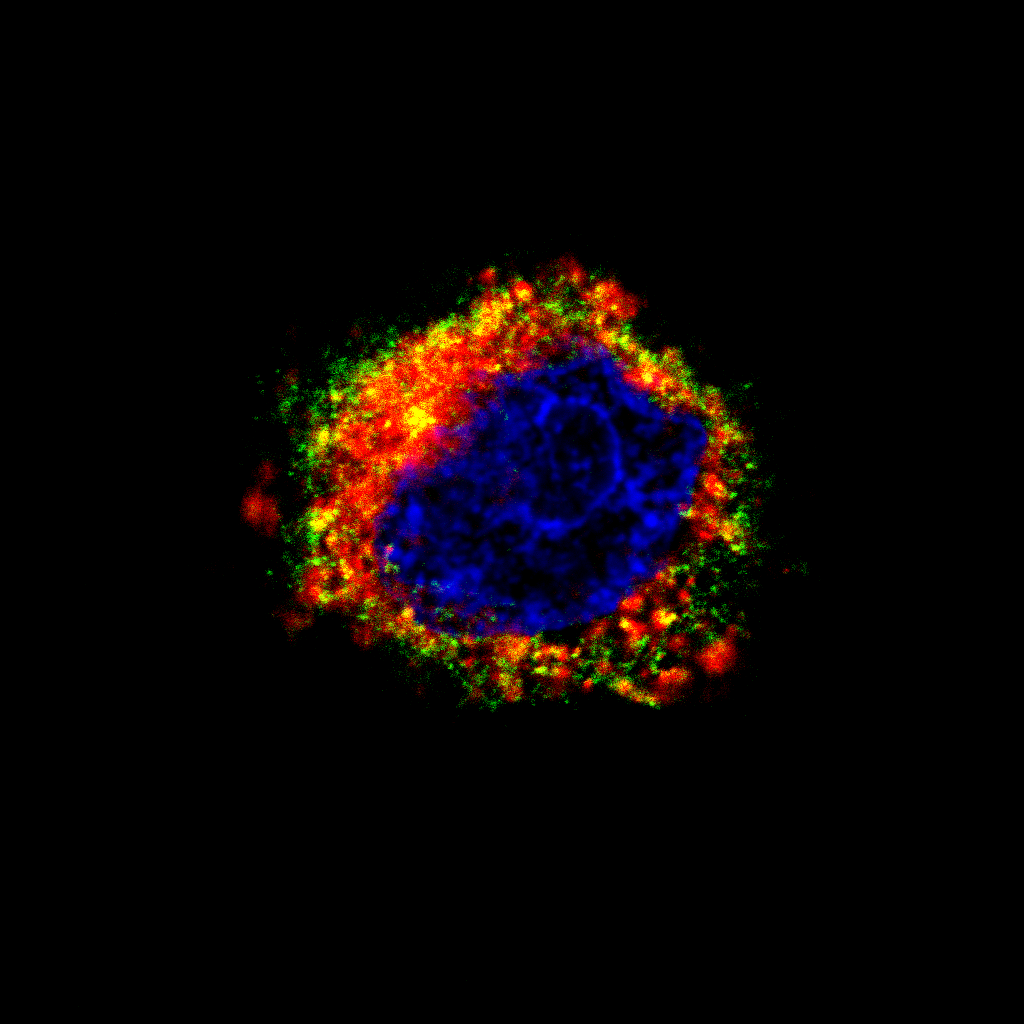

Supplement: Supplementary file 13 — Figure EV3 Source Data [file 44318_2024_353_MOESM13_ESM.zip › EVFigure 3/3J/PLCPRF5 SIUBE2F/Project_PP5SI2fLAMP2MTOR60X3.5-1.tif]

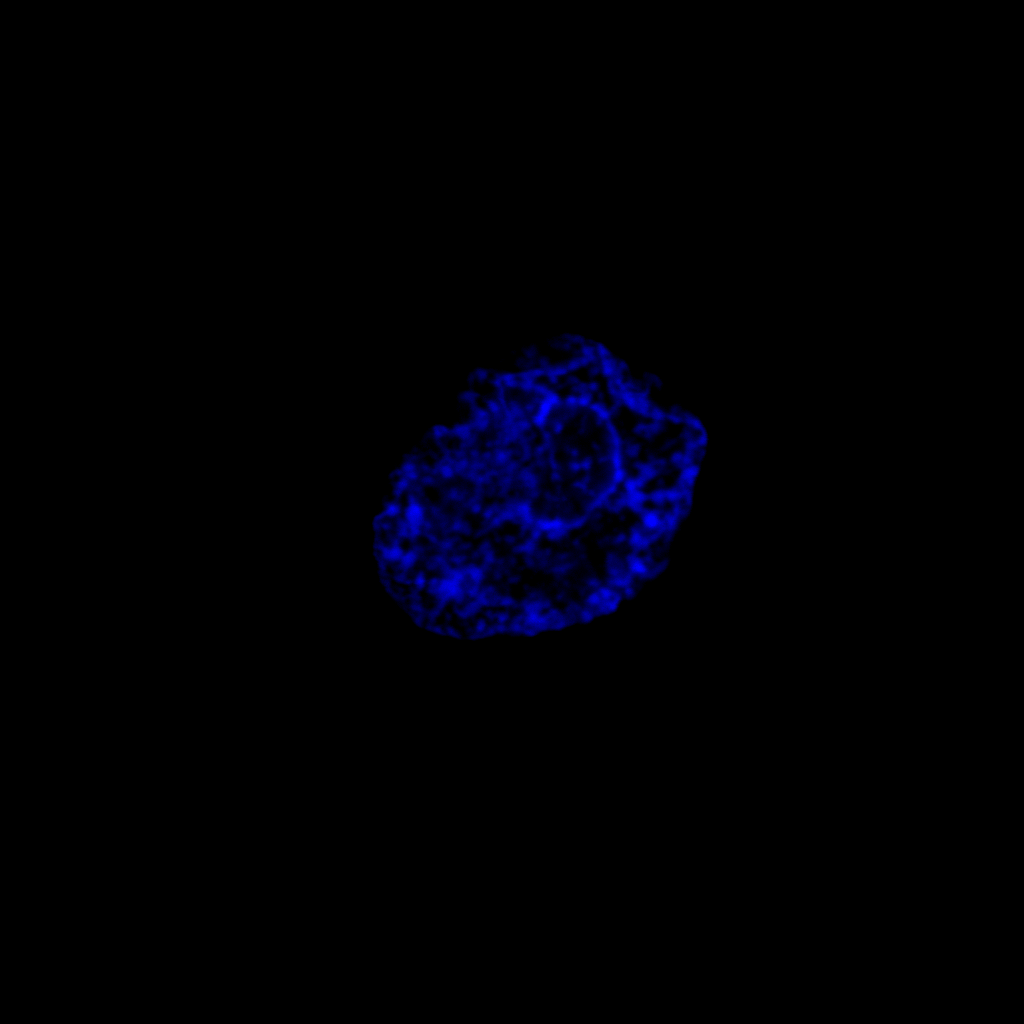

Supplement: Supplementary file 13 — Figure EV3 Source Data [file 44318_2024_353_MOESM13_ESM.zip › EVFigure 3/3J/PLCPRF5 SIUBE2F/Project_PP5SI2fLAMP2MTOR60X3.5-1_ch00.tif]

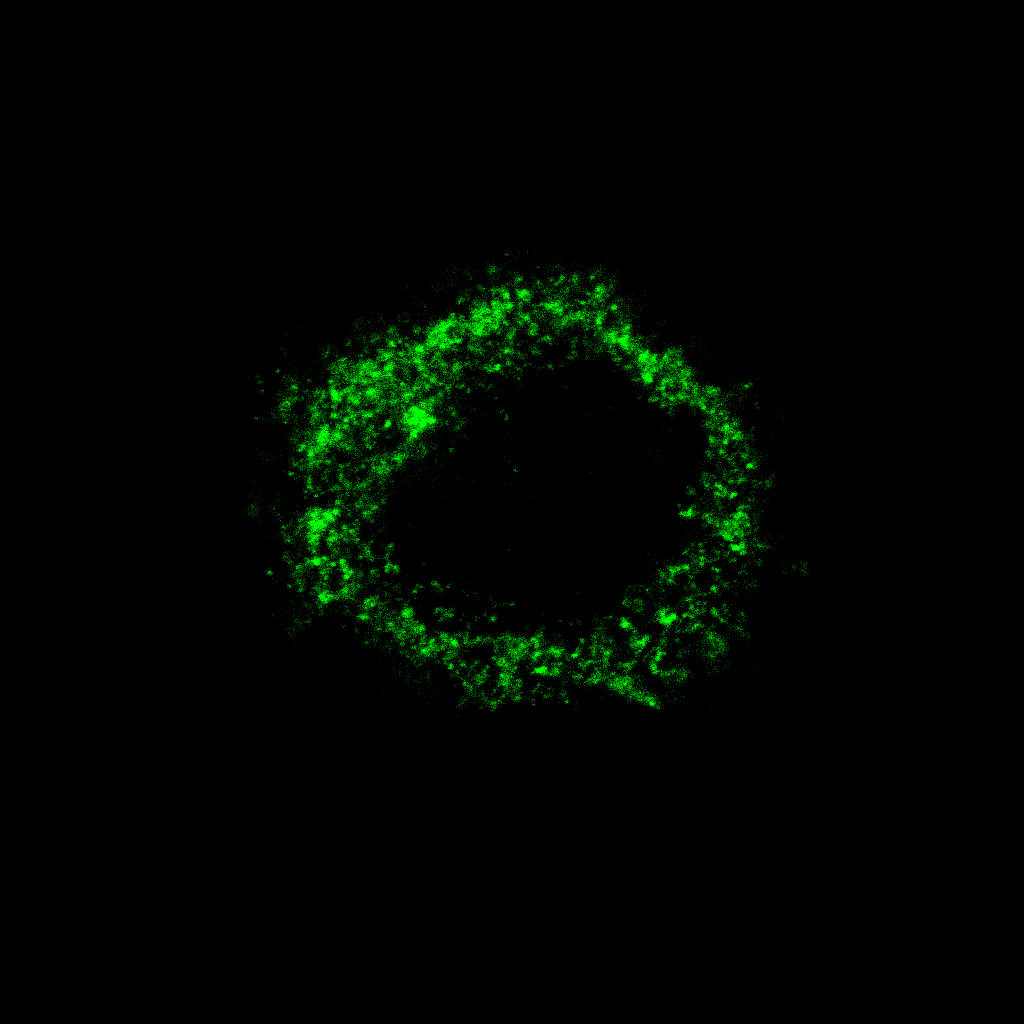

Supplement: Supplementary file 13 — Figure EV3 Source Data [file 44318_2024_353_MOESM13_ESM.zip › EVFigure 3/3J/PLCPRF5 SIUBE2F/Project_PP5SI2fLAMP2MTOR60X3.5-1_ch01.tif]

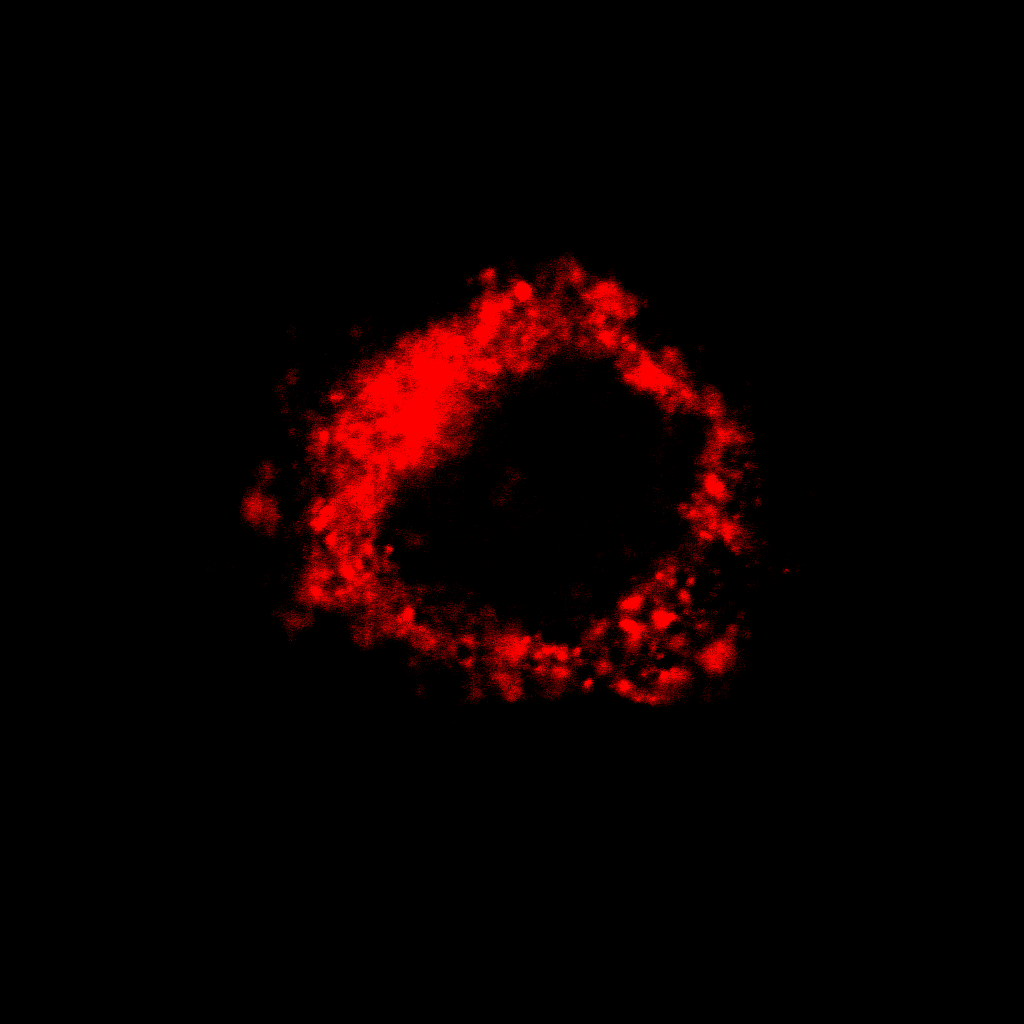

Supplement: Supplementary file 13 — Figure EV3 Source Data [file 44318_2024_353_MOESM13_ESM.zip › EVFigure 3/3J/PLCPRF5 SIUBE2F/Project_PP5SI2fLAMP2MTOR60X3.5-1_ch02.tif]

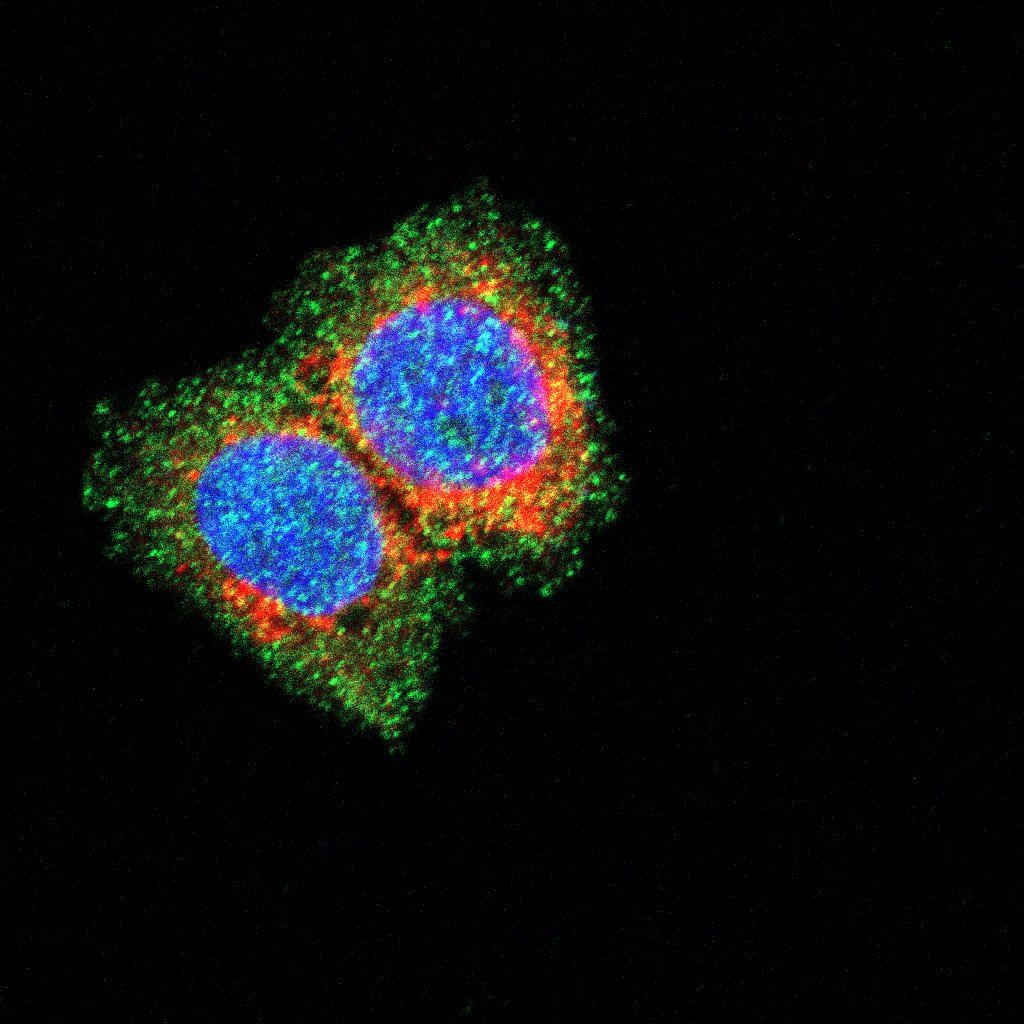

Supplement: Supplementary file 15 — Figure EV5 Source Data [file 44318_2024_353_MOESM15_ESM.zip › EVFigure 5/5F/RHEB-K169R/HP_RGB.tif]

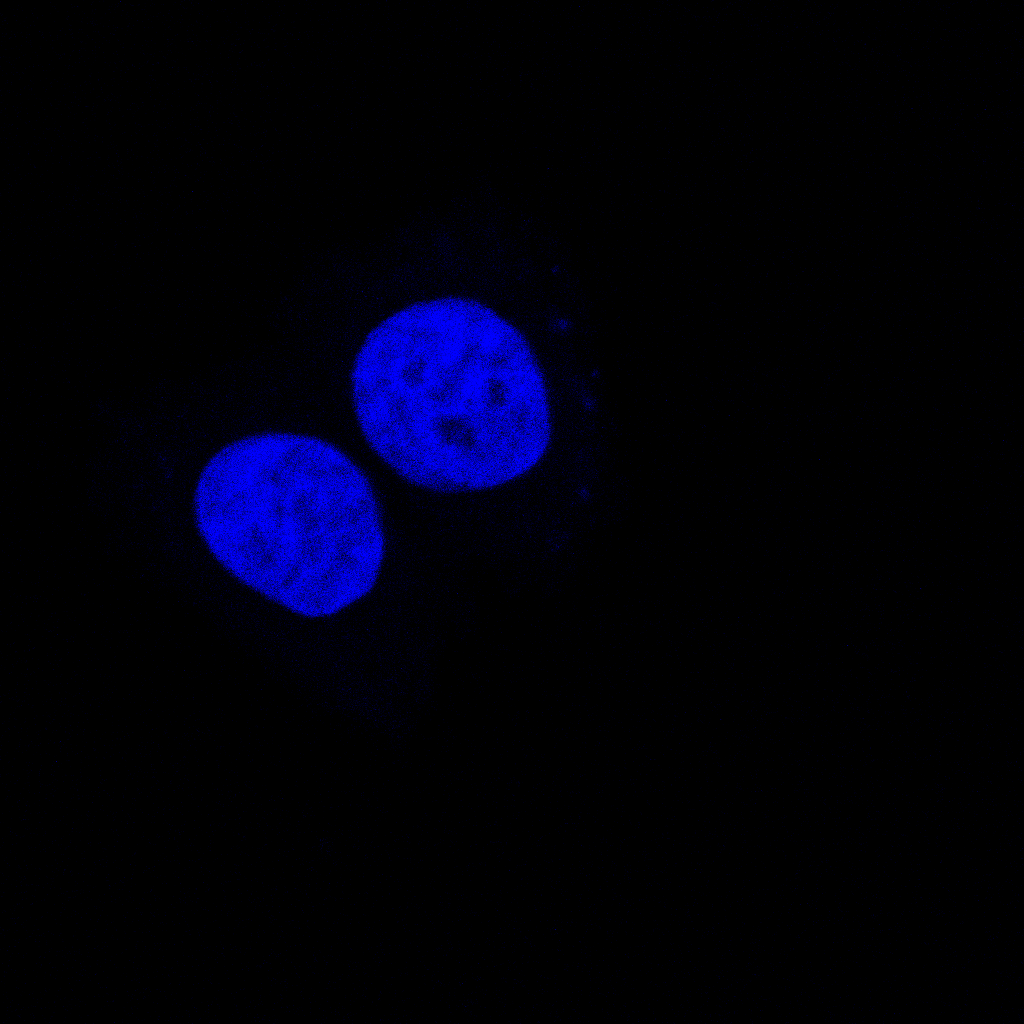

Supplement: Supplementary file 15 — Figure EV5 Source Data [file 44318_2024_353_MOESM15_ESM.zip › EVFigure 5/5F/RHEB-K169R/HP_RGB_DAPI.tif]

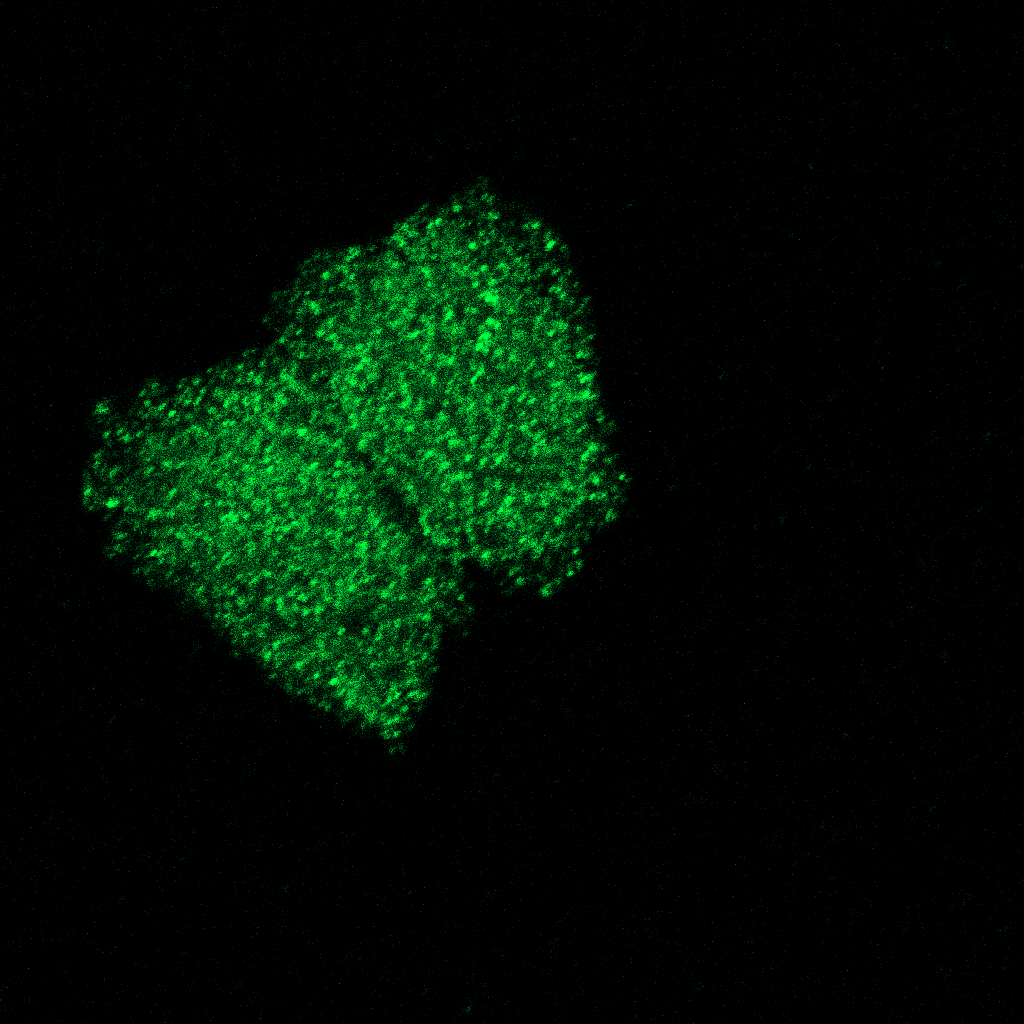

Supplement: Supplementary file 15 — Figure EV5 Source Data [file 44318_2024_353_MOESM15_ESM.zip › EVFigure 5/5F/RHEB-K169R/HP_RGB_FITC.tif]

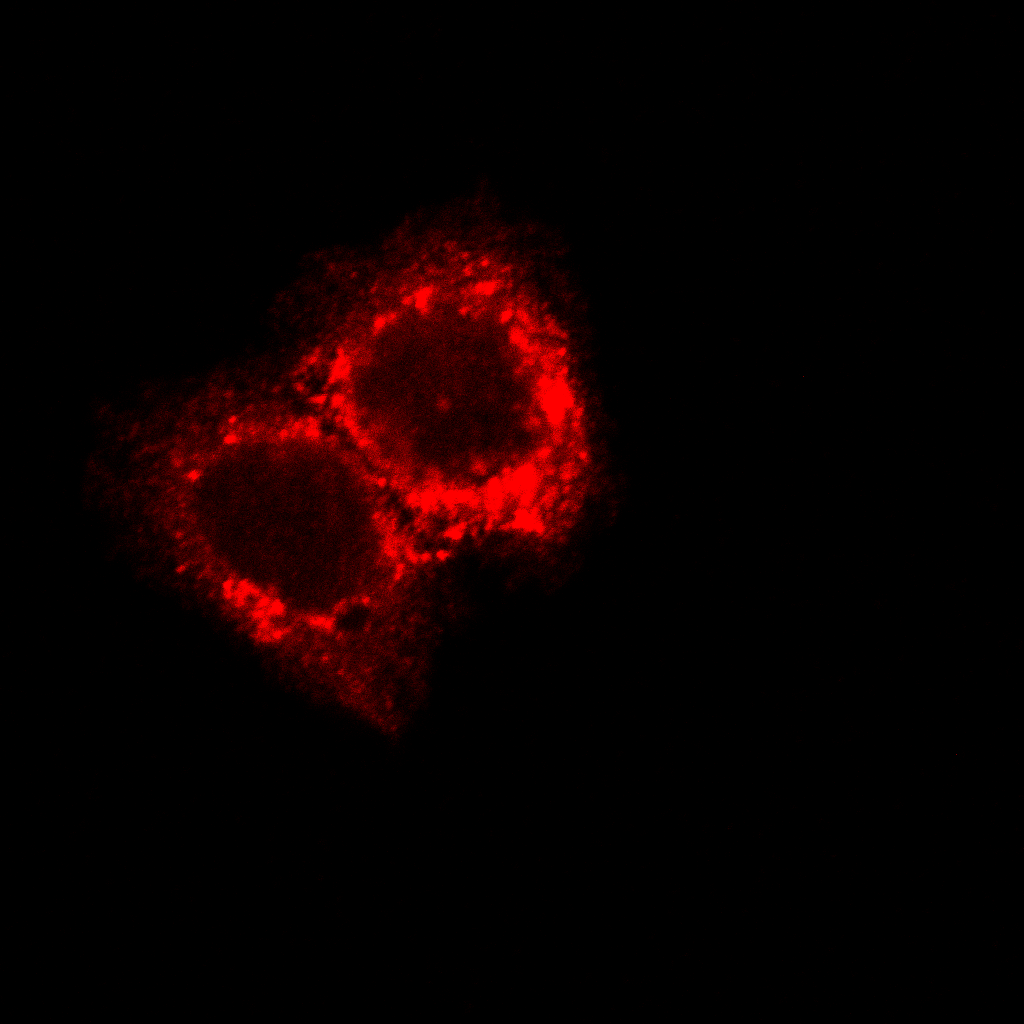

Supplement: Supplementary file 15 — Figure EV5 Source Data [file 44318_2024_353_MOESM15_ESM.zip › EVFigure 5/5F/RHEB-K169R/HP_RGB_TRITC.tif]

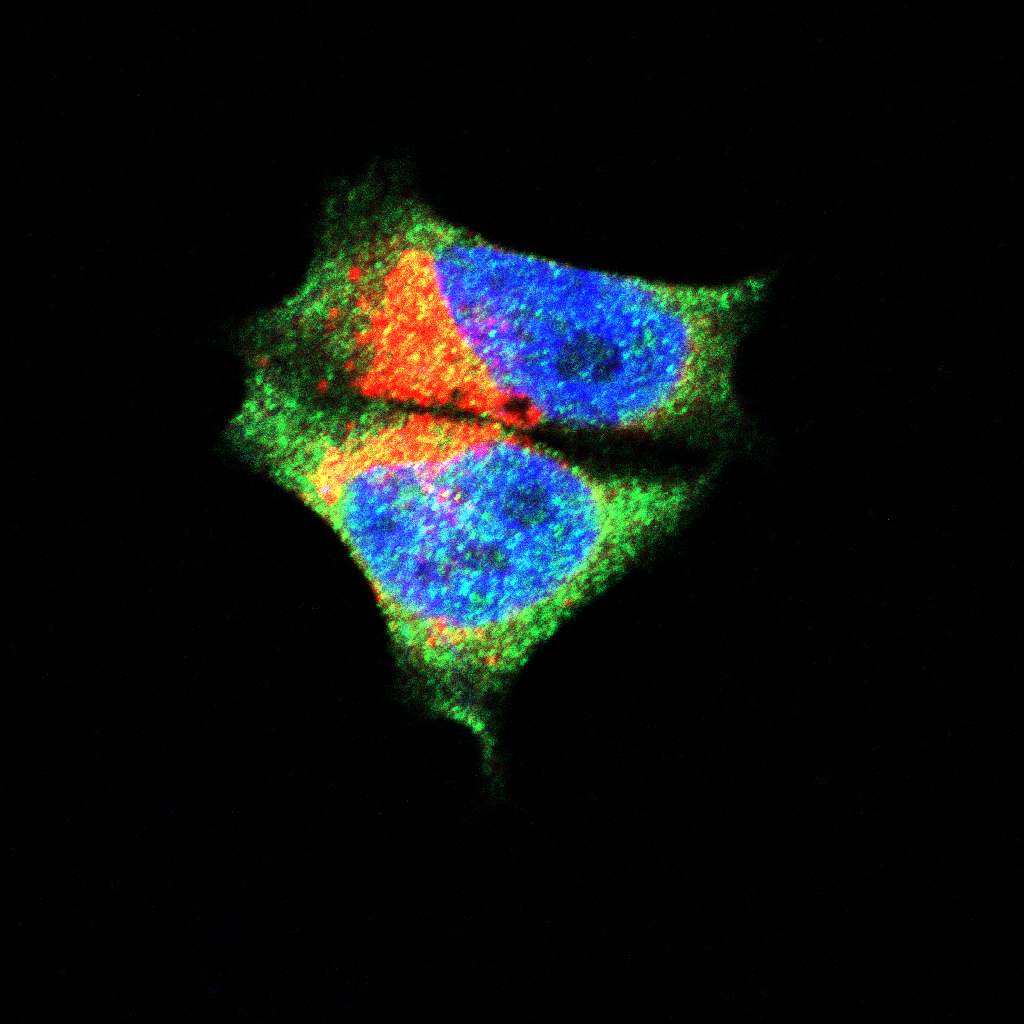

Supplement: Supplementary file 15 — Figure EV5 Source Data [file 44318_2024_353_MOESM15_ESM.zip › EVFigure 5/5F/WT-RHEB/HP_RGB.tif]

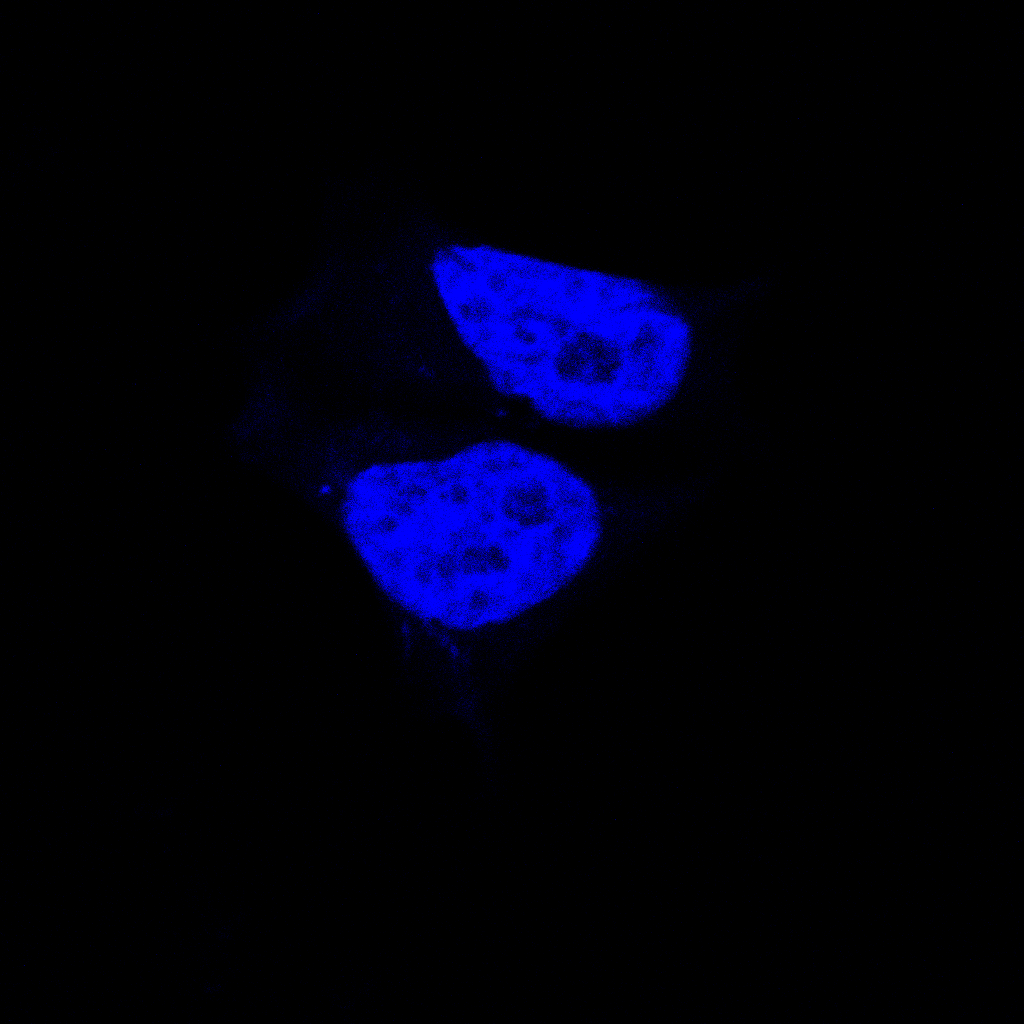

Supplement: Supplementary file 15 — Figure EV5 Source Data [file 44318_2024_353_MOESM15_ESM.zip › EVFigure 5/5F/WT-RHEB/HP_RGB_DAPI.tif]

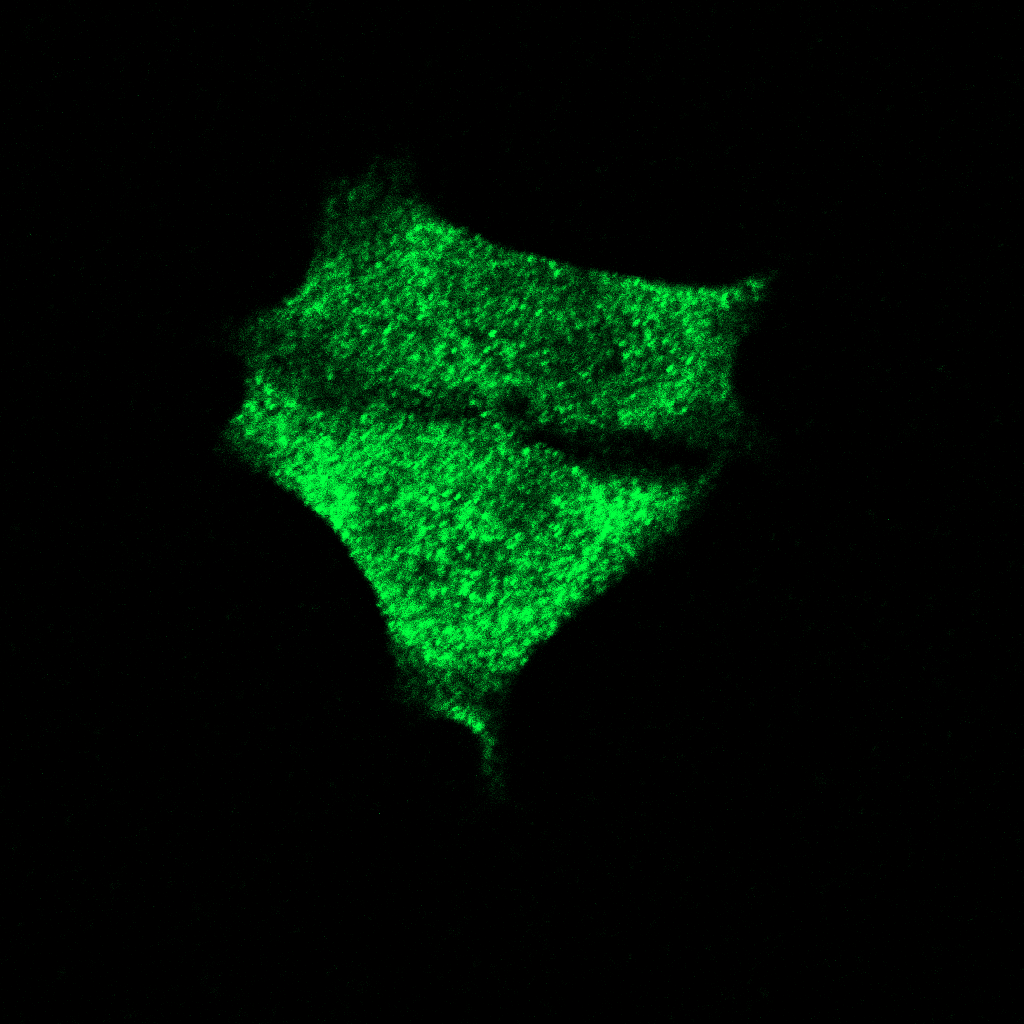

Supplement: Supplementary file 15 — Figure EV5 Source Data [file 44318_2024_353_MOESM15_ESM.zip › EVFigure 5/5F/WT-RHEB/HP_RGB_FITC.tif]

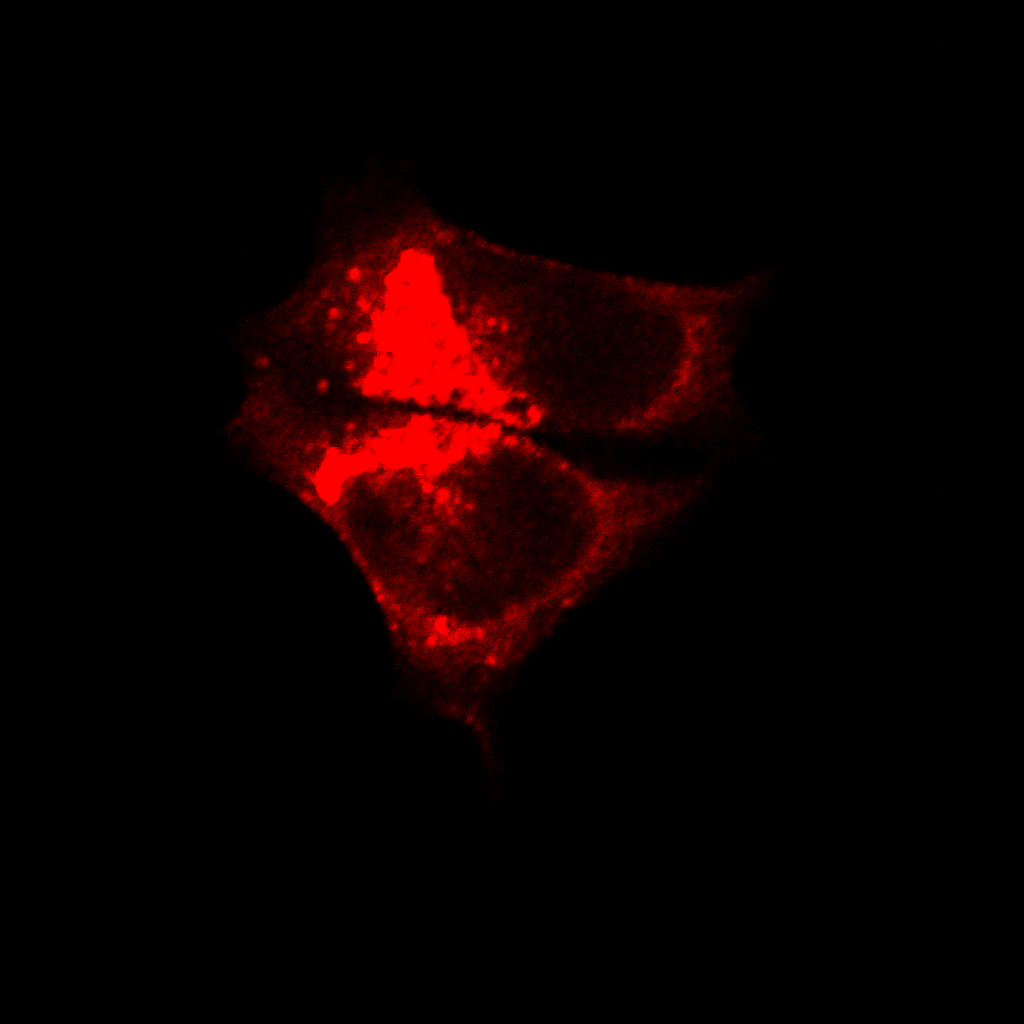

Supplement: Supplementary file 15 — Figure EV5 Source Data [file 44318_2024_353_MOESM15_ESM.zip › EVFigure 5/5F/WT-RHEB/HP_RGB_TRITC.tif]

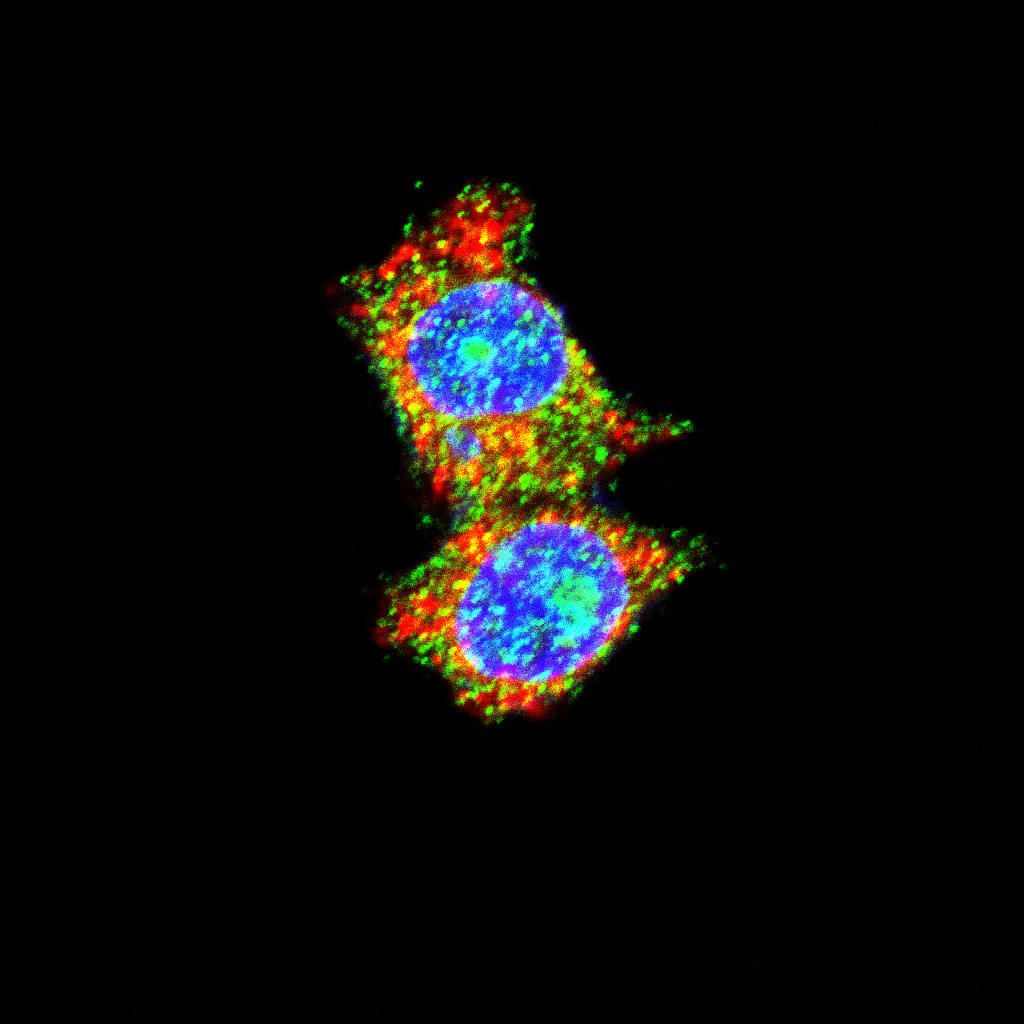

Supplement: Supplementary file 15 — Figure EV5 Source Data [file 44318_2024_353_MOESM15_ESM.zip › EVFigure 5/5H/PLCPRF5 RHEB-K169R+FLAG-N8/HP_RGB.tif]

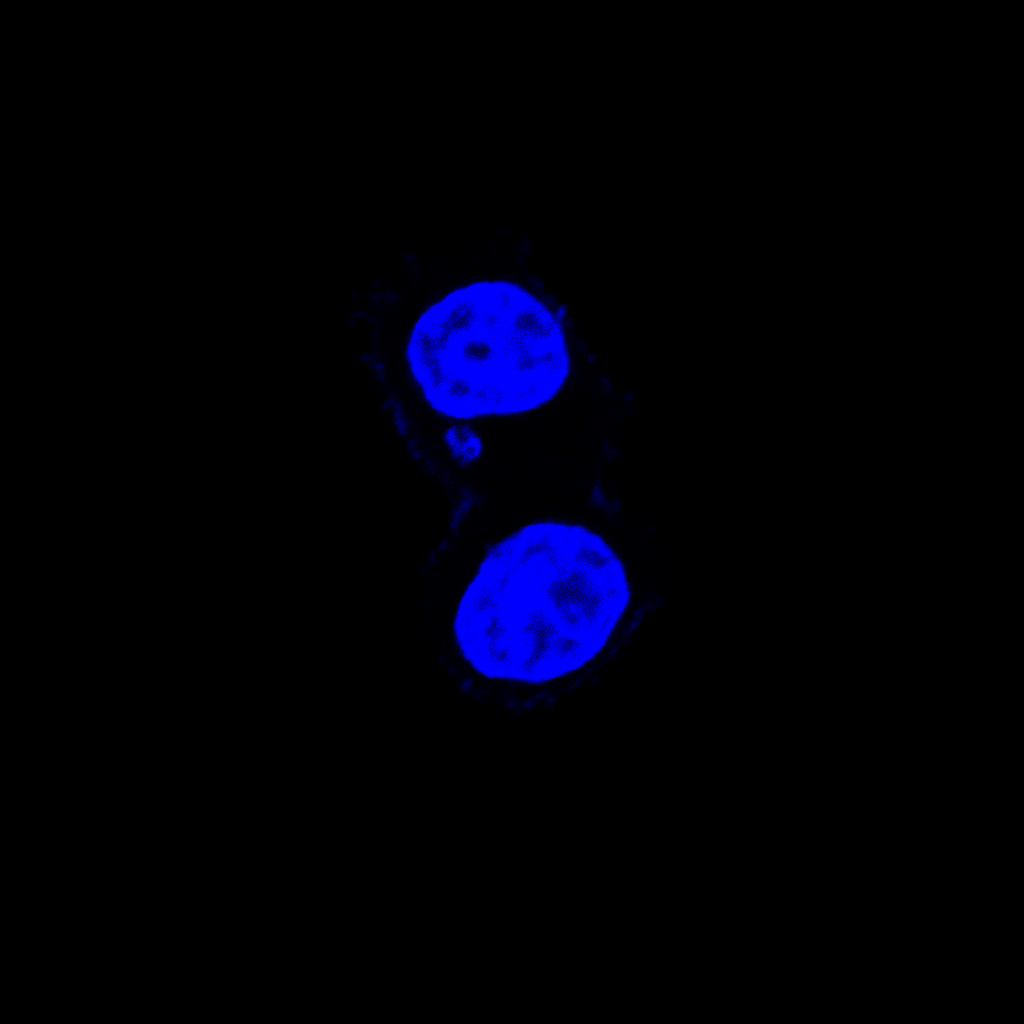

Supplement: Supplementary file 15 — Figure EV5 Source Data [file 44318_2024_353_MOESM15_ESM.zip › EVFigure 5/5H/PLCPRF5 RHEB-K169R+FLAG-N8/HP_RGB_DAPI.tif]

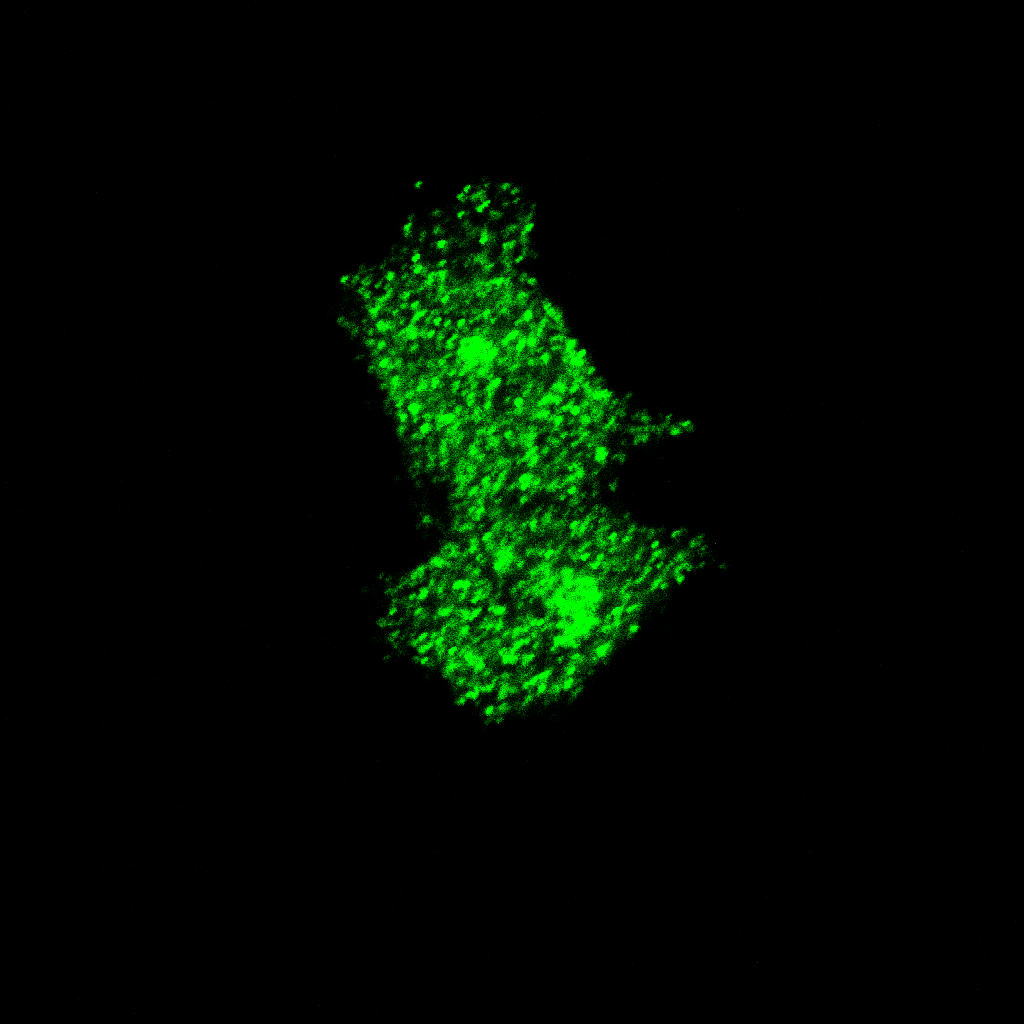

Supplement: Supplementary file 15 — Figure EV5 Source Data [file 44318_2024_353_MOESM15_ESM.zip › EVFigure 5/5H/PLCPRF5 RHEB-K169R+FLAG-N8/HP_RGB_FITC.tif]

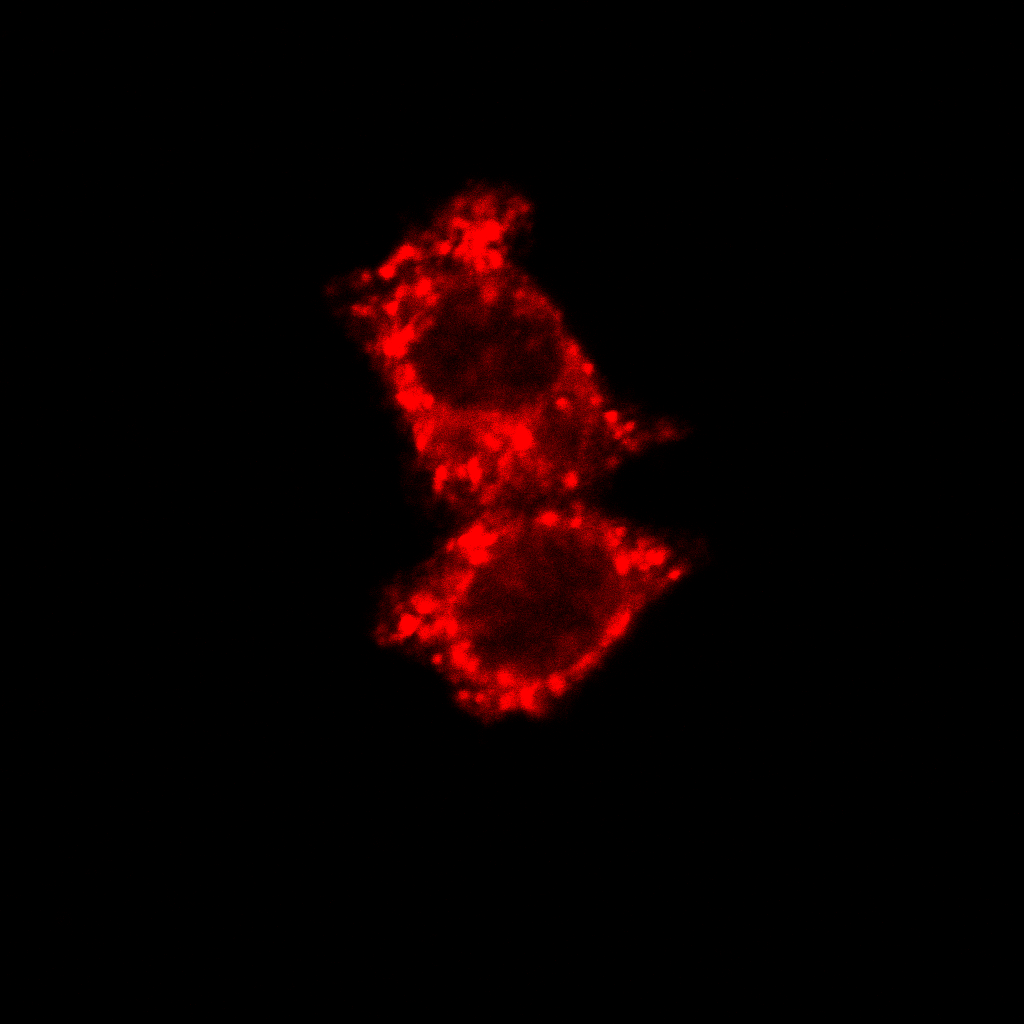

Supplement: Supplementary file 15 — Figure EV5 Source Data [file 44318_2024_353_MOESM15_ESM.zip › EVFigure 5/5H/PLCPRF5 RHEB-K169R+FLAG-N8/HP_RGB_TRITC.tif]

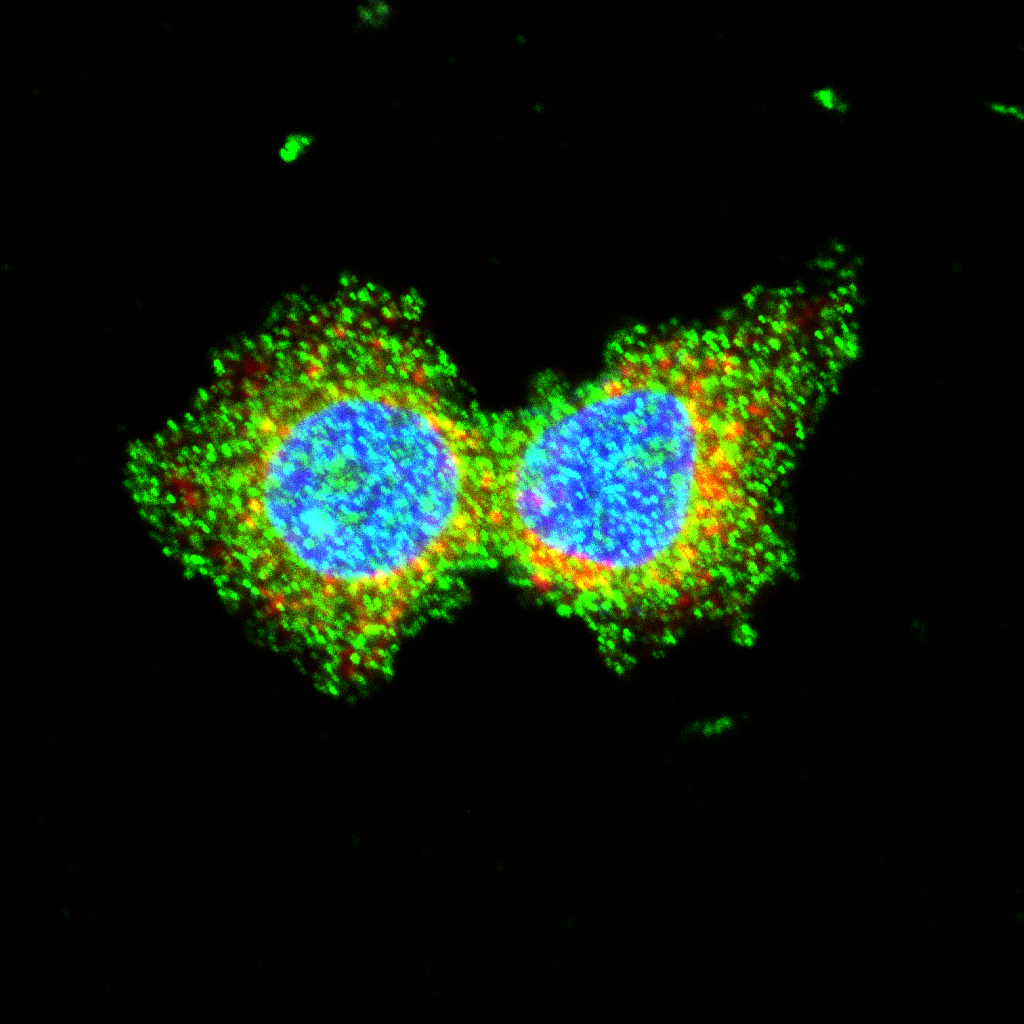

Supplement: Supplementary file 15 — Figure EV5 Source Data [file 44318_2024_353_MOESM15_ESM.zip › EVFigure 5/5H/PLCPRF5 RHEB-K169R+Vector/HP_RGB.tif]

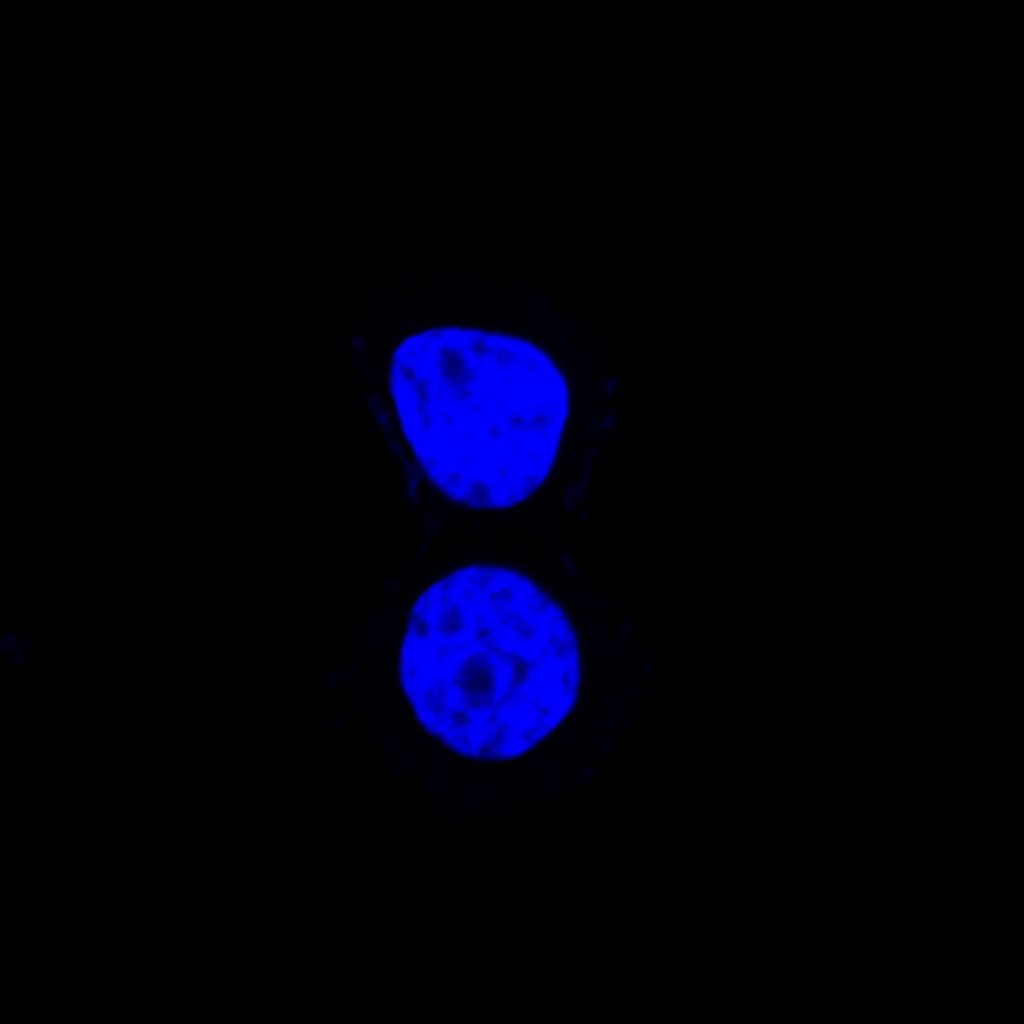

Supplement: Supplementary file 15 — Figure EV5 Source Data [file 44318_2024_353_MOESM15_ESM.zip › EVFigure 5/5H/PLCPRF5 RHEB-K169R+Vector/HP_RGB_DAPI.tif]

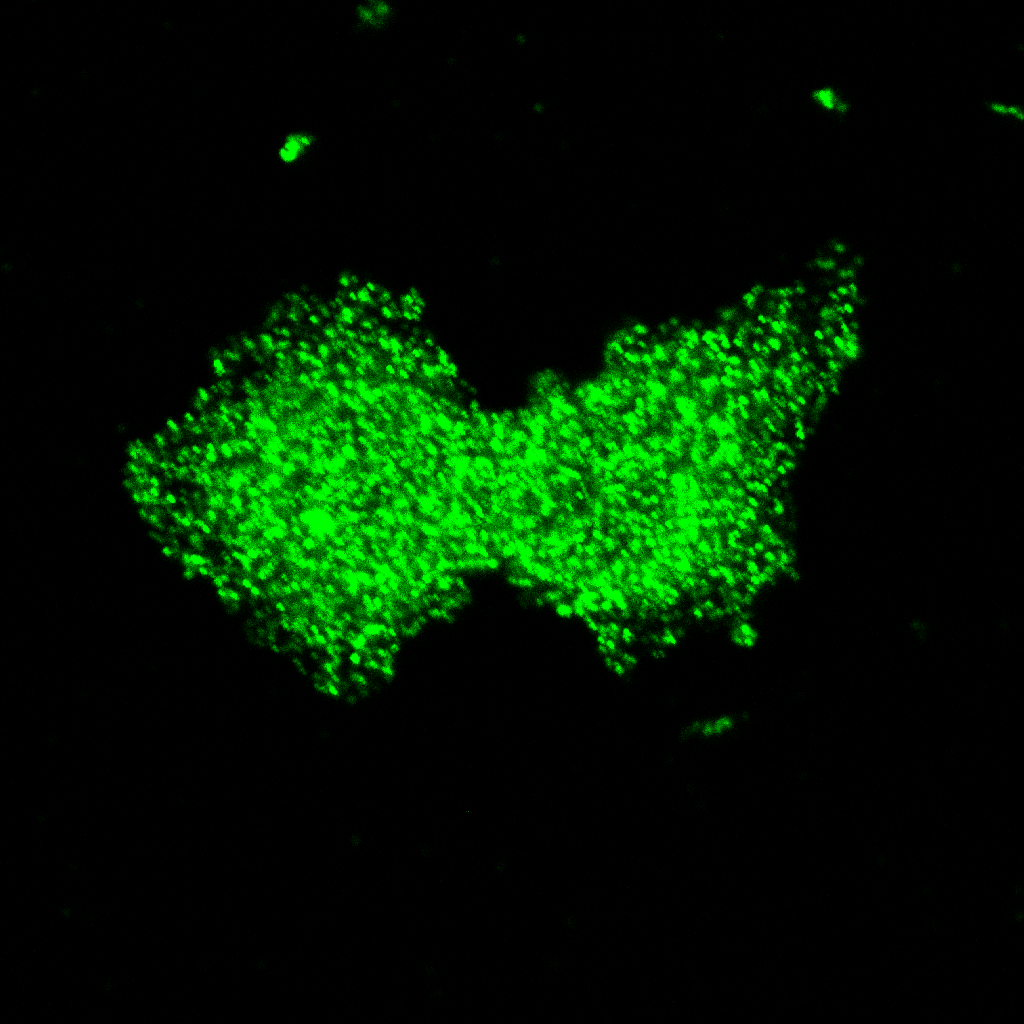

Supplement: Supplementary file 15 — Figure EV5 Source Data [file 44318_2024_353_MOESM15_ESM.zip › EVFigure 5/5H/PLCPRF5 RHEB-K169R+Vector/HP_RGB_FITC.tif]

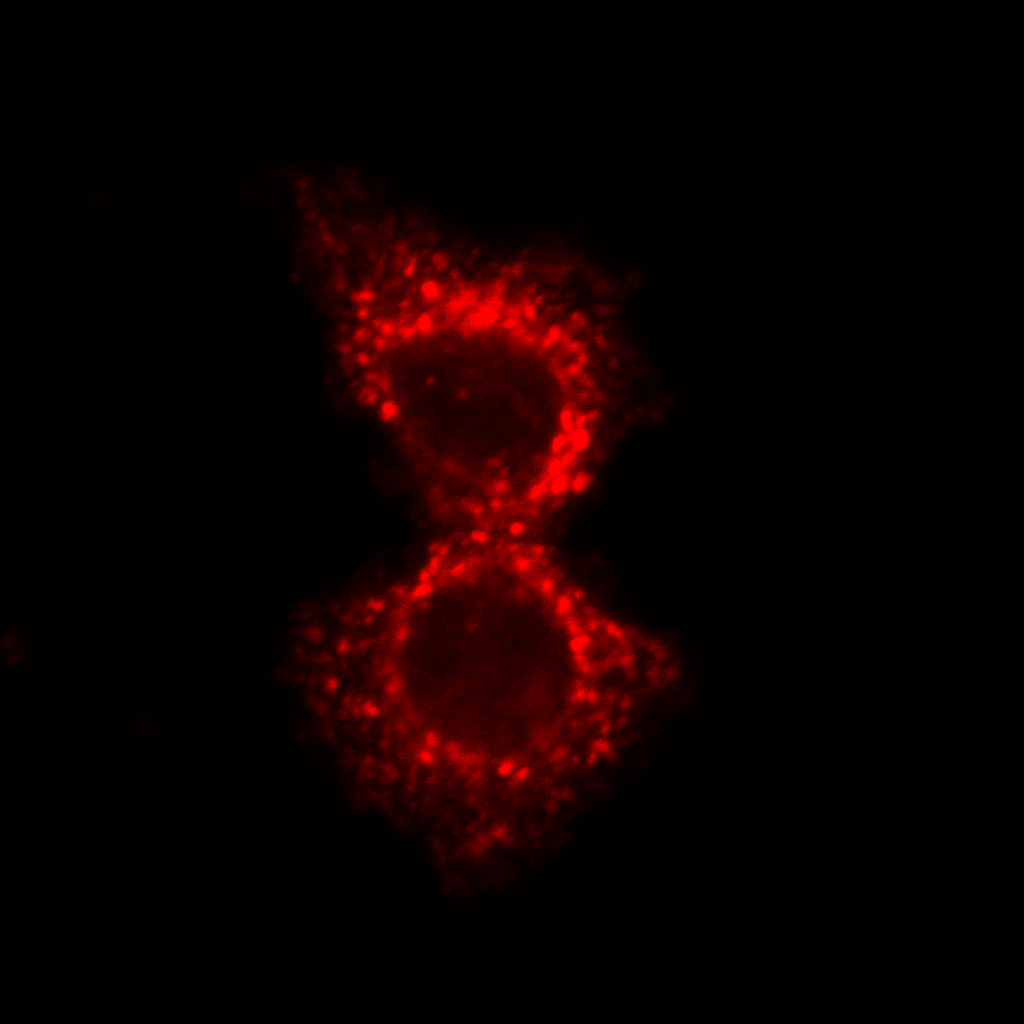

Supplement: Supplementary file 15 — Figure EV5 Source Data [file 44318_2024_353_MOESM15_ESM.zip › EVFigure 5/5H/PLCPRF5 RHEB-K169R+Vector/HP_RGB_TRITC.tif]

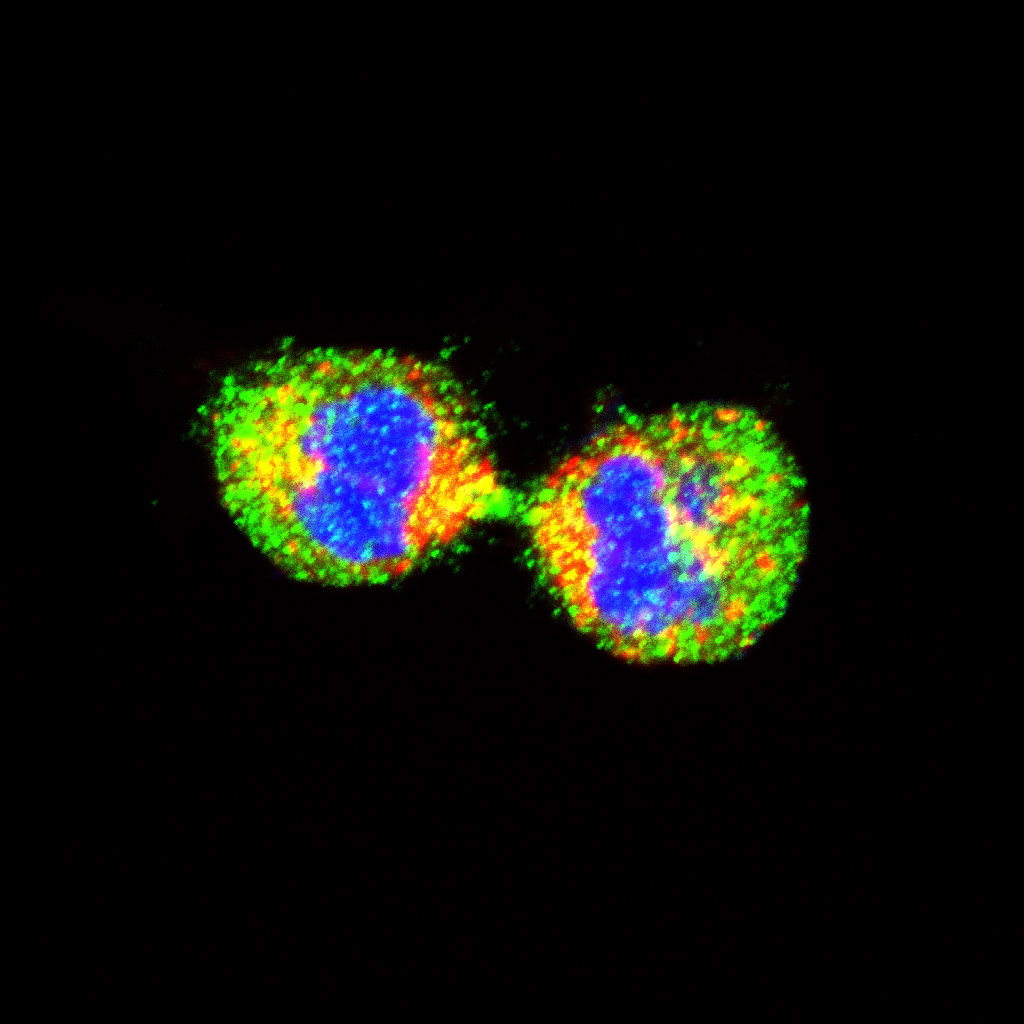

Supplement: Supplementary file 15 — Figure EV5 Source Data [file 44318_2024_353_MOESM15_ESM.zip › EVFigure 5/5H/PLCPRF5 WT-RHEB+FLAG-N8/HP_RGB.tif]

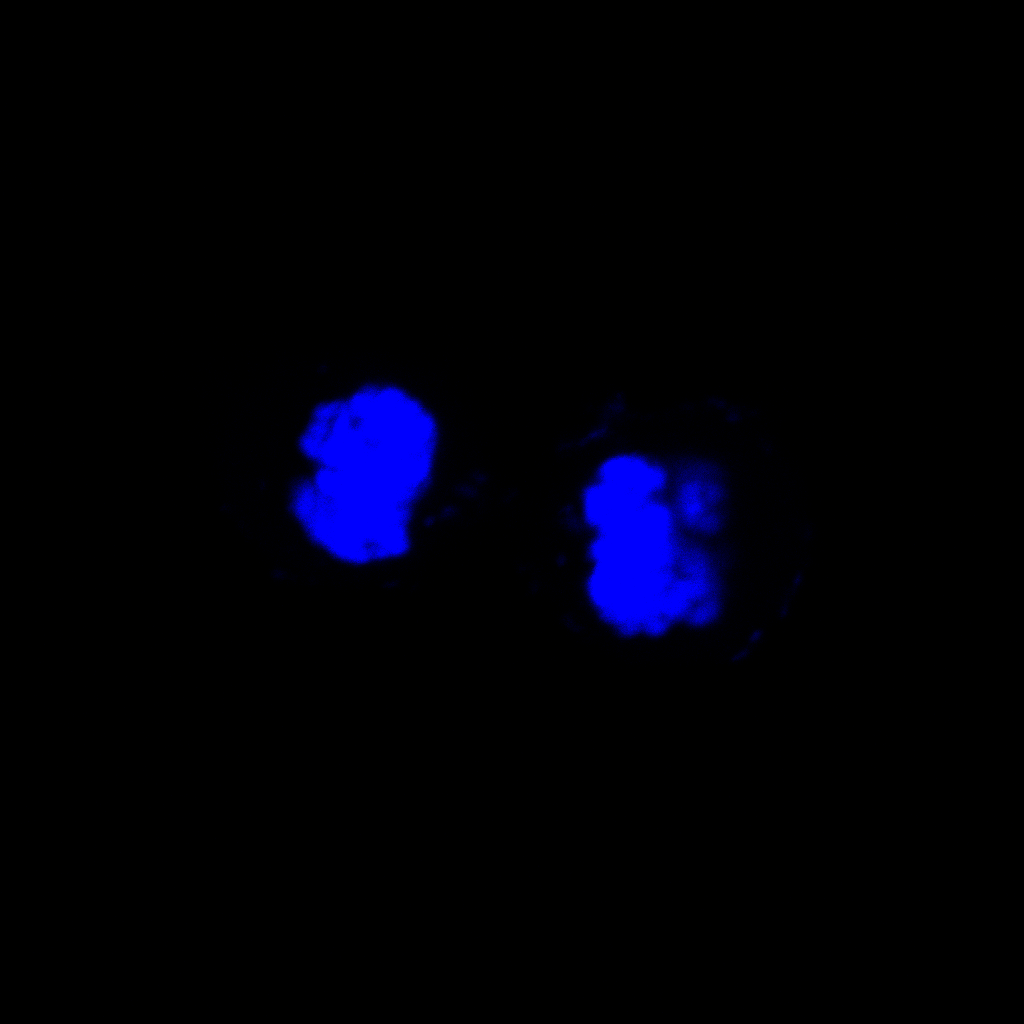

Supplement: Supplementary file 15 — Figure EV5 Source Data [file 44318_2024_353_MOESM15_ESM.zip › EVFigure 5/5H/PLCPRF5 WT-RHEB+FLAG-N8/HP_RGB_DAPI.tif]

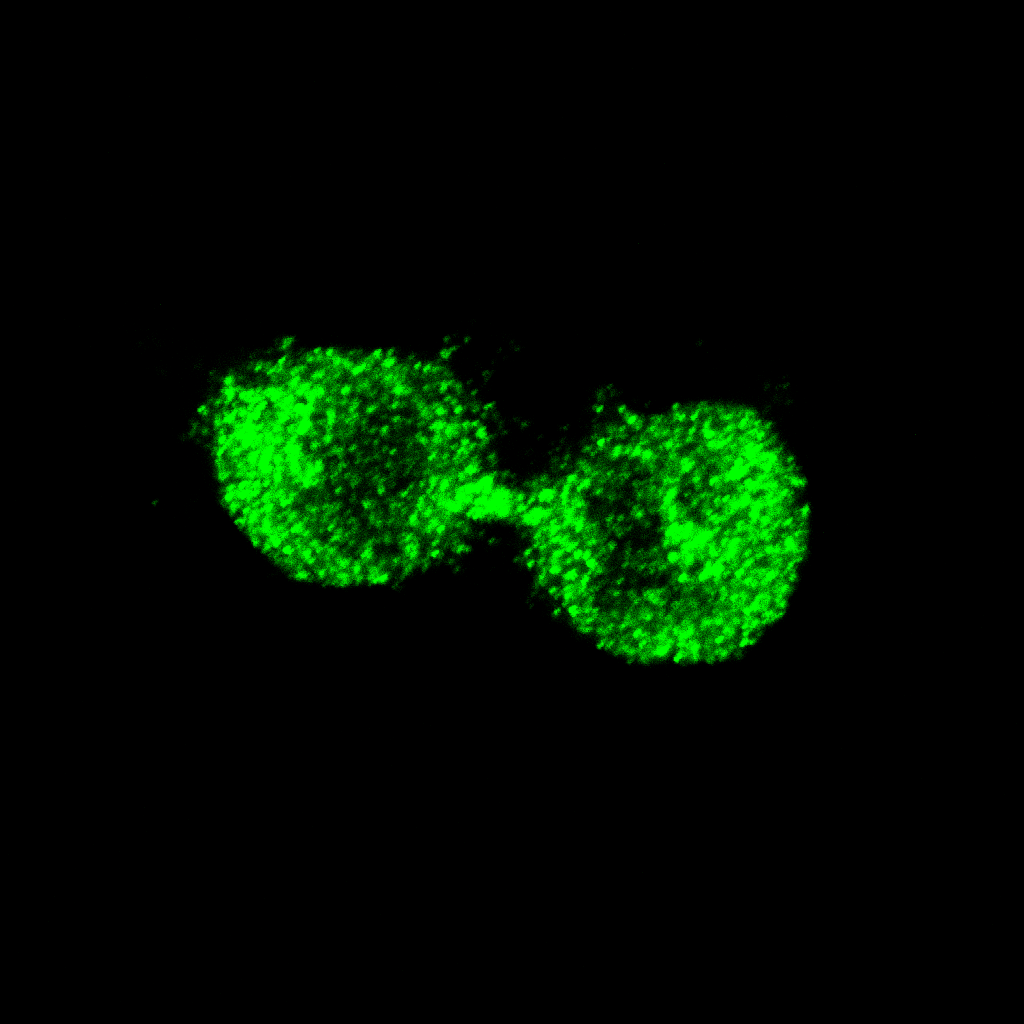

Supplement: Supplementary file 15 — Figure EV5 Source Data [file 44318_2024_353_MOESM15_ESM.zip › EVFigure 5/5H/PLCPRF5 WT-RHEB+FLAG-N8/HP_RGB_FITC.tif]

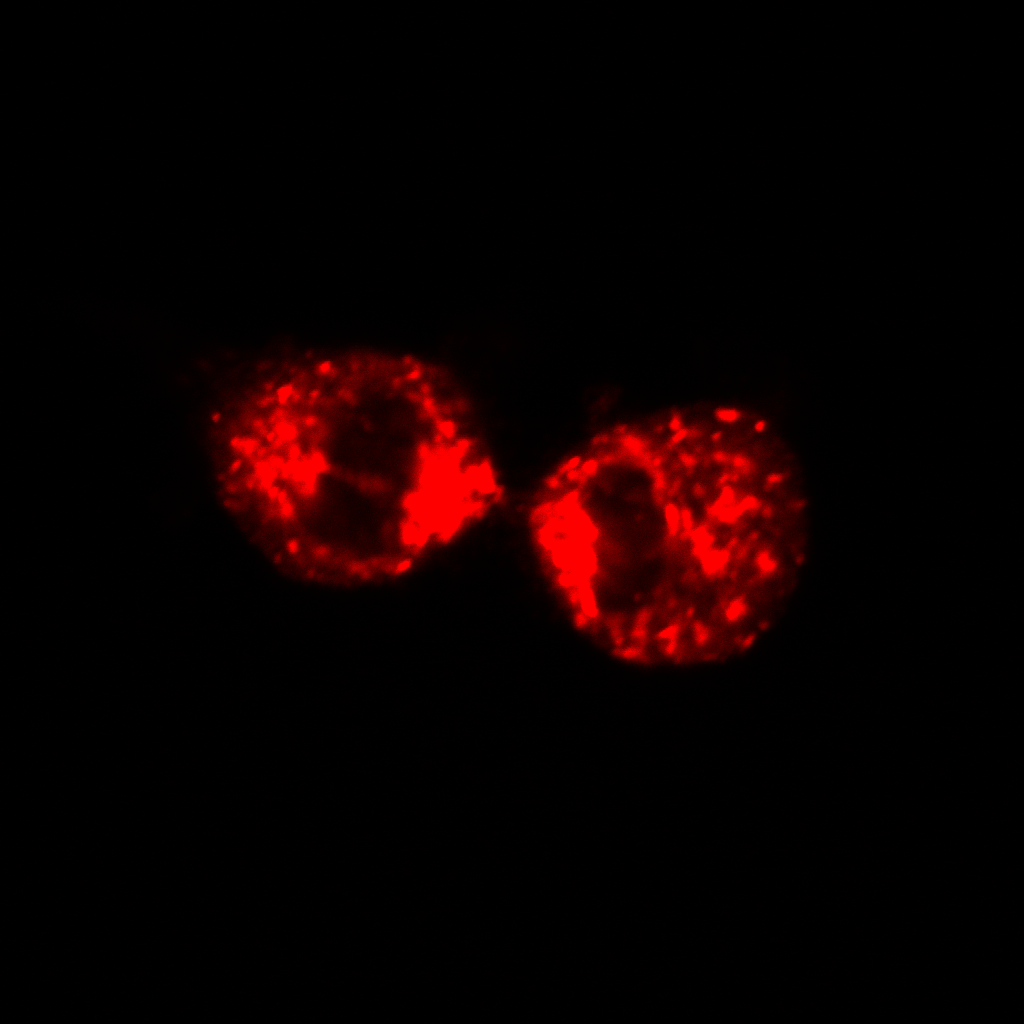

Supplement: Supplementary file 15 — Figure EV5 Source Data [file 44318_2024_353_MOESM15_ESM.zip › EVFigure 5/5H/PLCPRF5 WT-RHEB+FLAG-N8/HP_RGB_TRITC.tif]

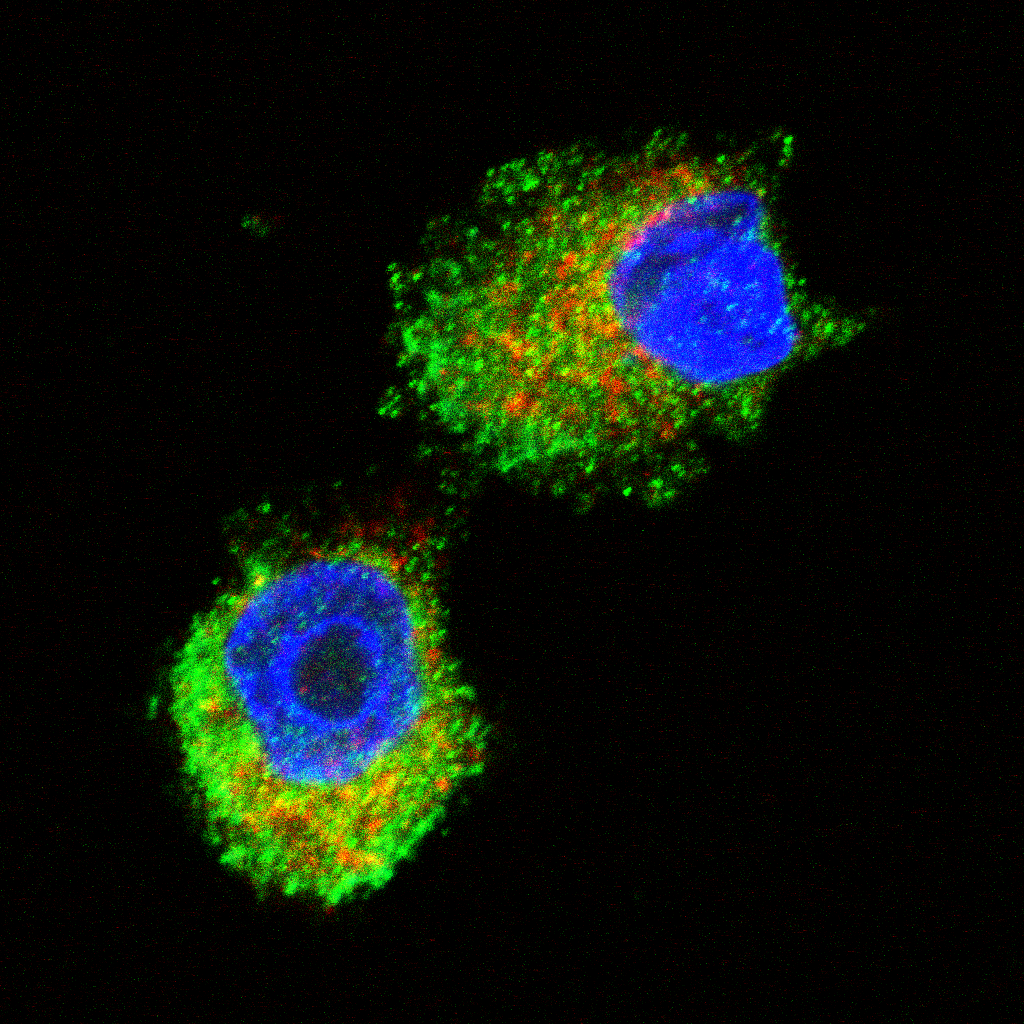

Supplement: Supplementary file 15 — Figure EV5 Source Data [file 44318_2024_353_MOESM15_ESM.zip › EVFigure 5/5H/PLCPRF5 WTRHEB+Vector/HP_PP5 WTRHEB+V HA-R+LAMP2-M 60X3.5-1-2_RGB.tif]

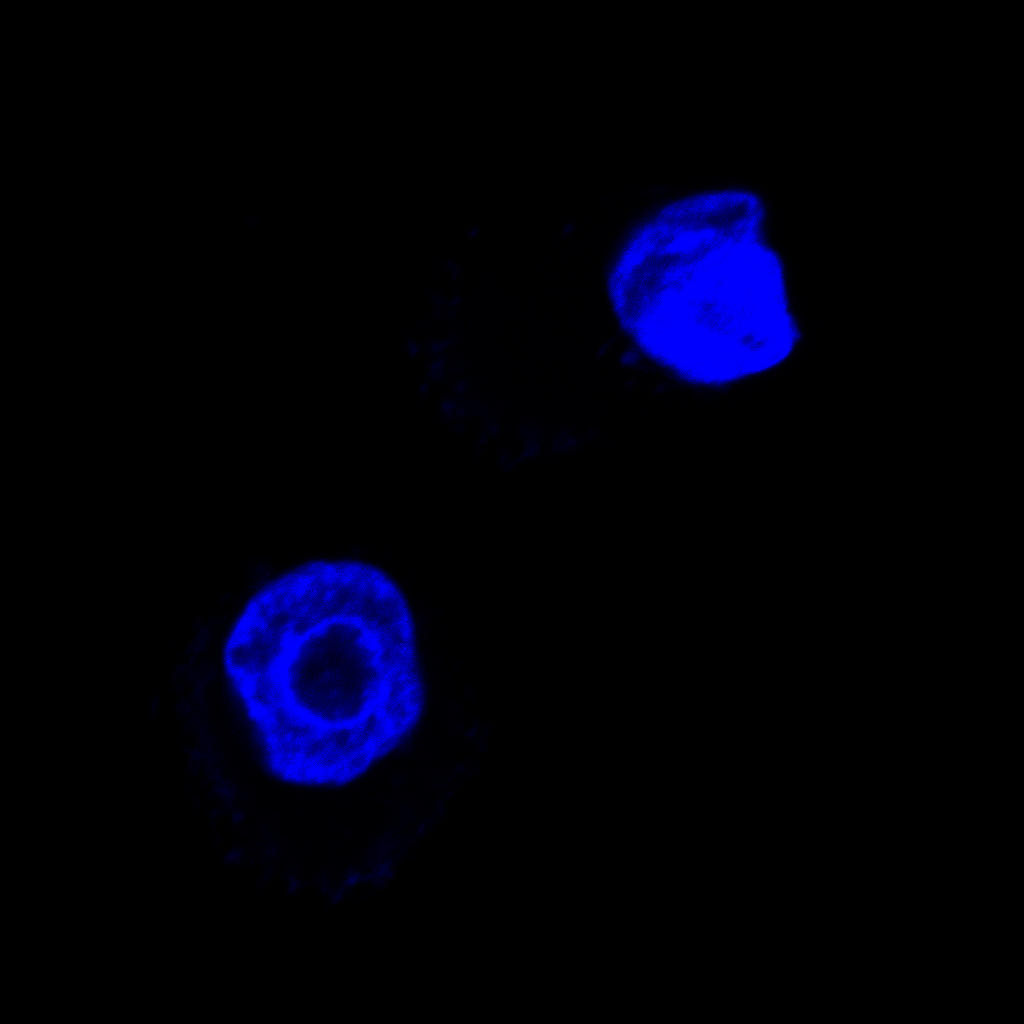

Supplement: Supplementary file 15 — Figure EV5 Source Data [file 44318_2024_353_MOESM15_ESM.zip › EVFigure 5/5H/PLCPRF5 WTRHEB+Vector/HP_PP5 WTRHEB+V HA-R+LAMP2-M 60X3.5-1-2_RGB_DAPI.tif]

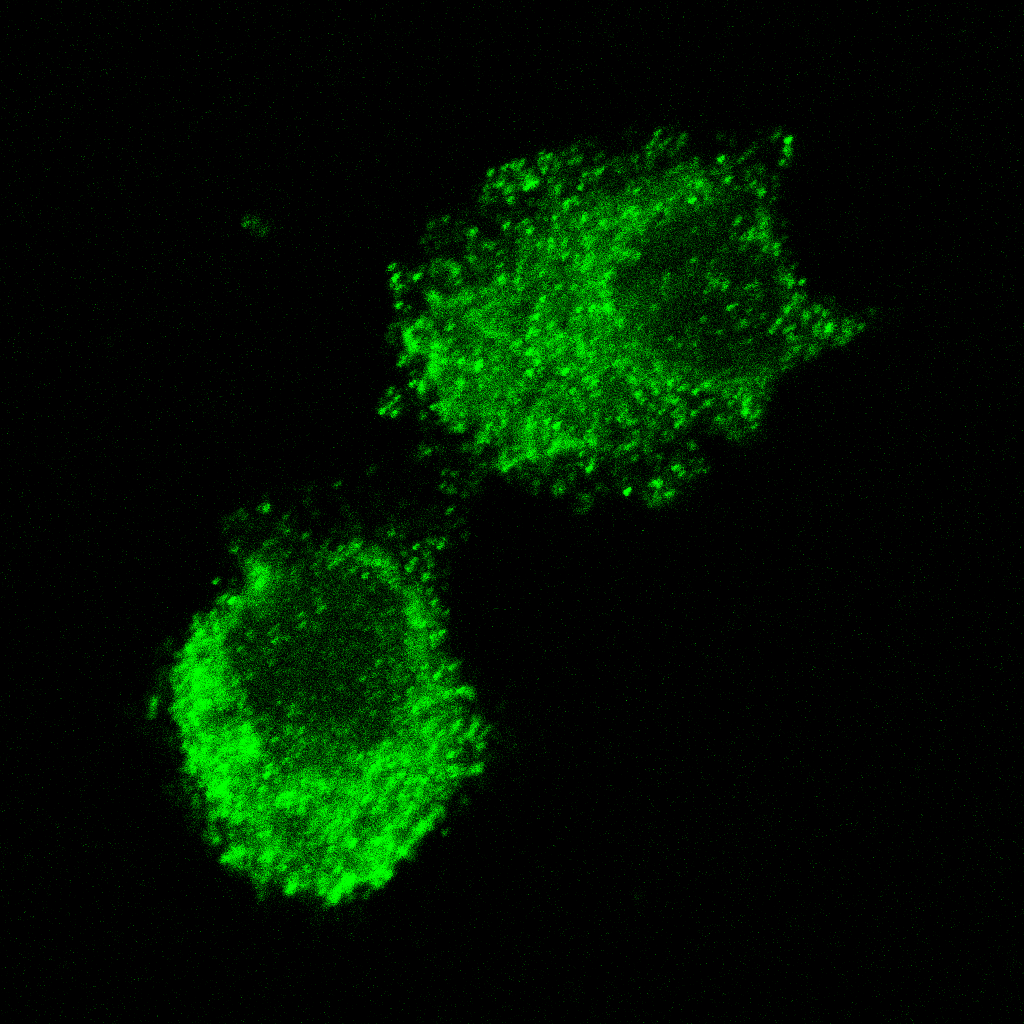

Supplement: Supplementary file 15 — Figure EV5 Source Data [file 44318_2024_353_MOESM15_ESM.zip › EVFigure 5/5H/PLCPRF5 WTRHEB+Vector/HP_PP5 WTRHEB+V HA-R+LAMP2-M 60X3.5-1-2_RGB_FITC.tif]

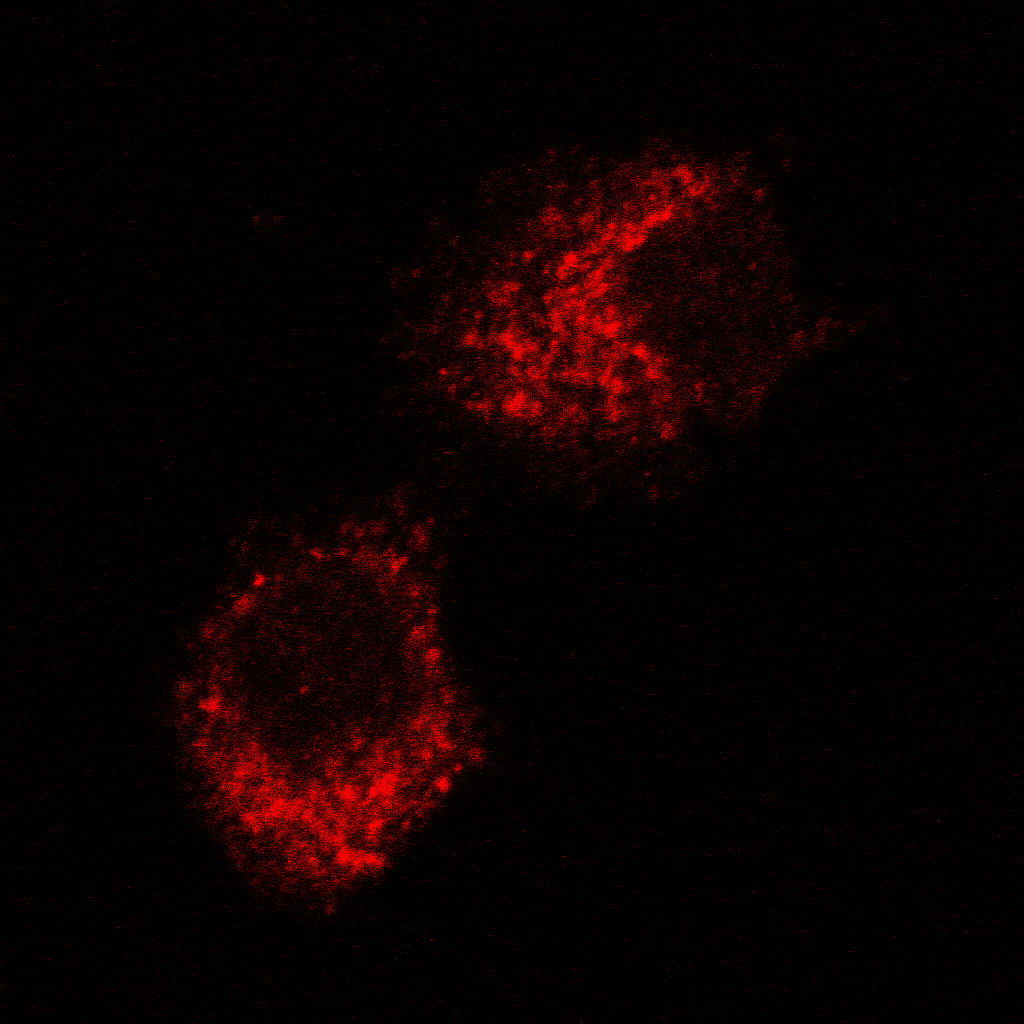

Supplement: Supplementary file 15 — Figure EV5 Source Data [file 44318_2024_353_MOESM15_ESM.zip › EVFigure 5/5H/PLCPRF5 WTRHEB+Vector/HP_PP5 WTRHEB+V HA-R+LAMP2-M 60X3.5-1-2_RGB_TRITC.tif]

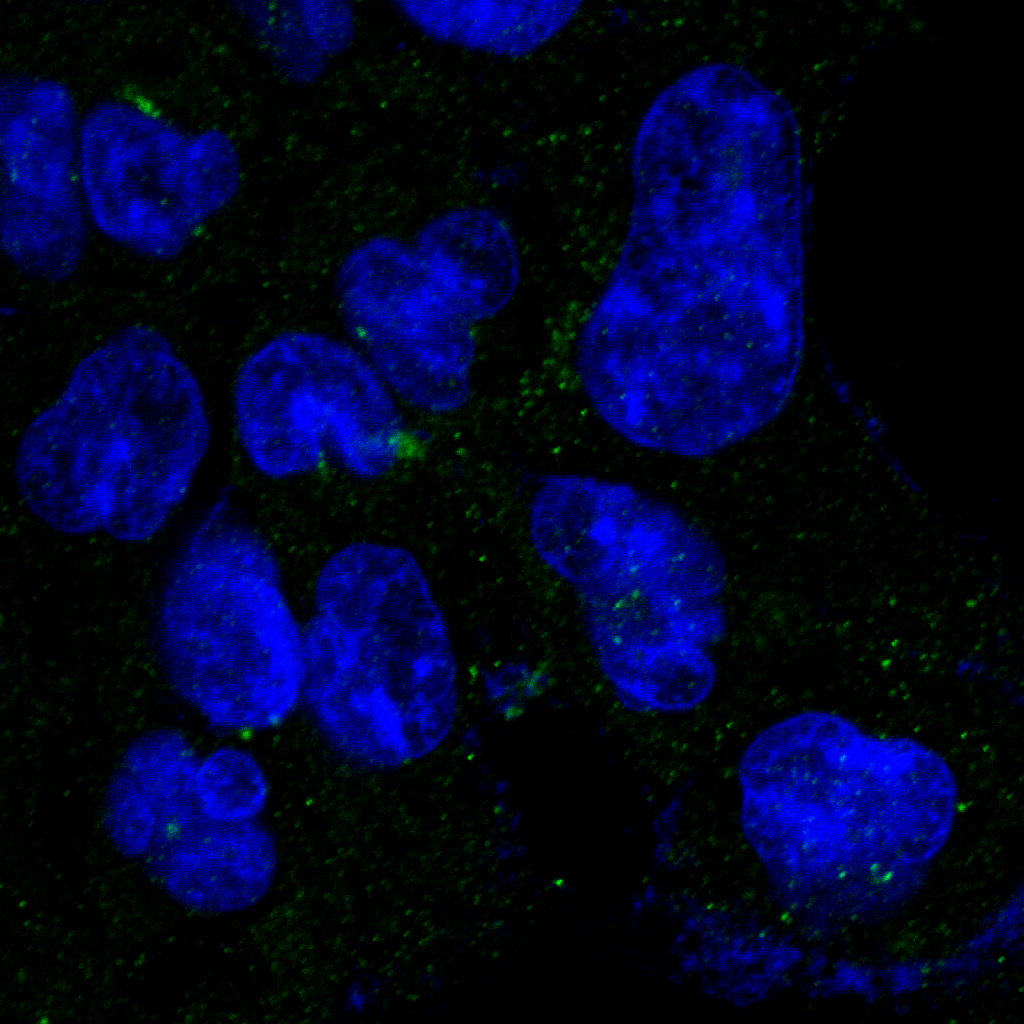

Supplement: Supplementary file 16 — Figure EV6 Source Data [file 44318_2024_353_MOESM16_ESM.zip › EVFigure 6/6E/Hep3B siCtrl+RHEB-K169R/HP_3B SINC+RHEBK169R 60X3-1_RGB.tif]

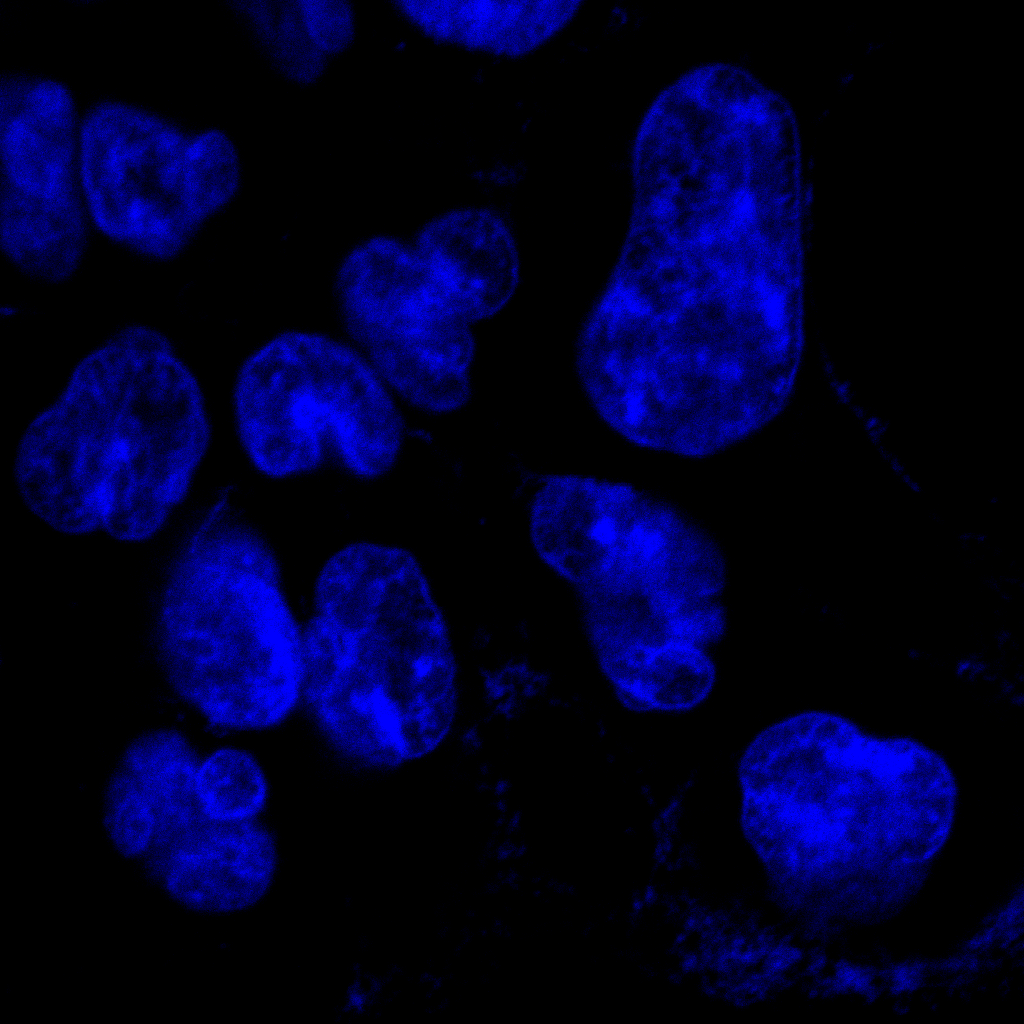

Supplement: Supplementary file 16 — Figure EV6 Source Data [file 44318_2024_353_MOESM16_ESM.zip › EVFigure 6/6E/Hep3B siCtrl+RHEB-K169R/HP_3B SINC+RHEBK169R 60X3-1_RGB_C1.tif]

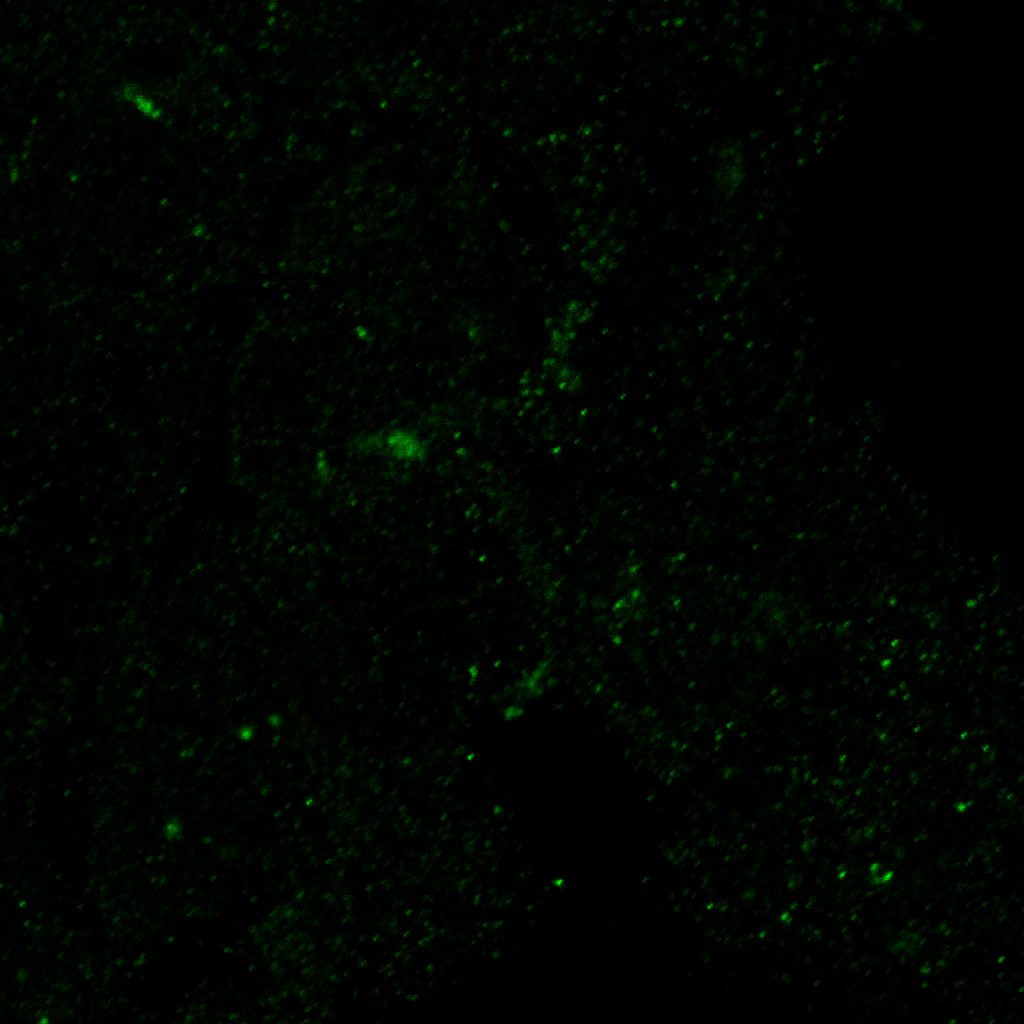

Supplement: Supplementary file 16 — Figure EV6 Source Data [file 44318_2024_353_MOESM16_ESM.zip › EVFigure 6/6E/Hep3B siCtrl+RHEB-K169R/HP_3B SINC+RHEBK169R 60X3-1_RGB_C2.tif]

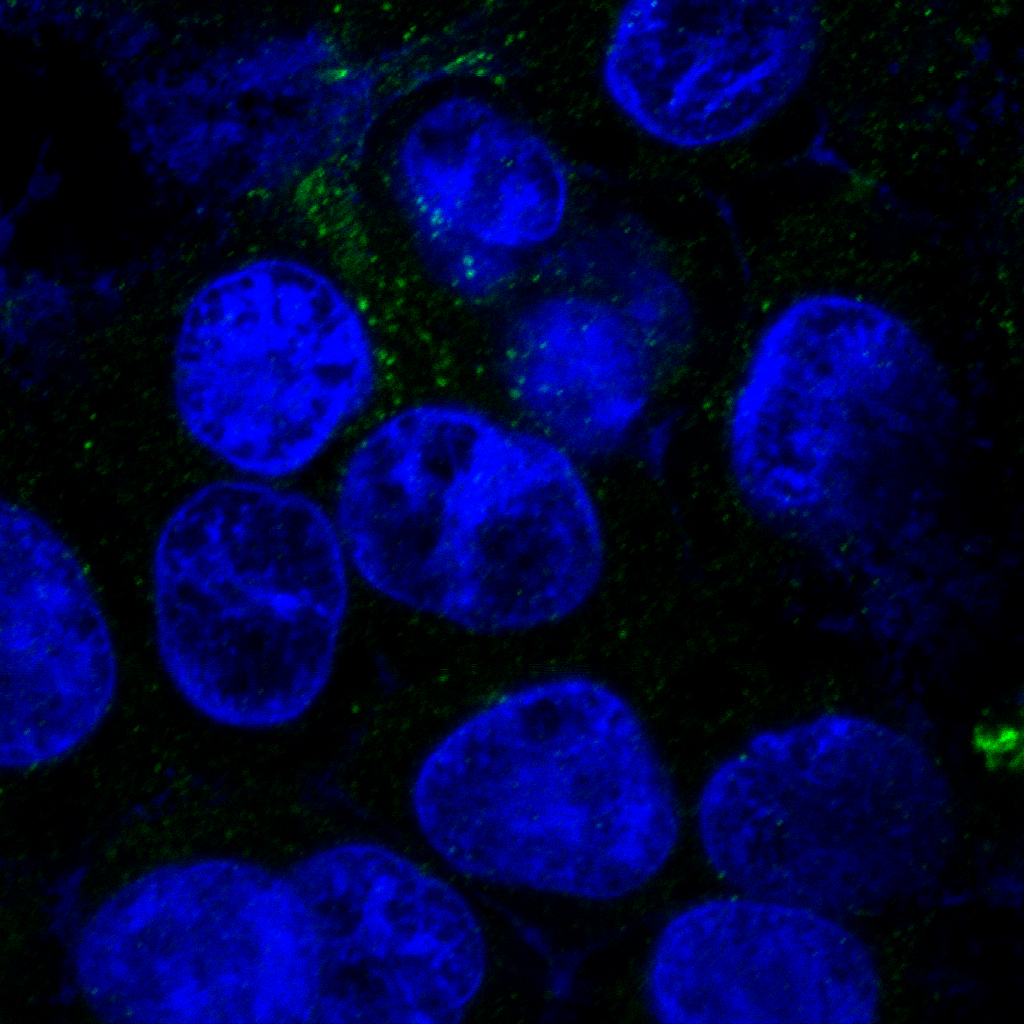

Supplement: Supplementary file 16 — Figure EV6 Source Data [file 44318_2024_353_MOESM16_ESM.zip › EVFigure 6/6E/Hep3B siCtrl+Vector/HP_3B SINC+V 60X3-1_RGB.tif]

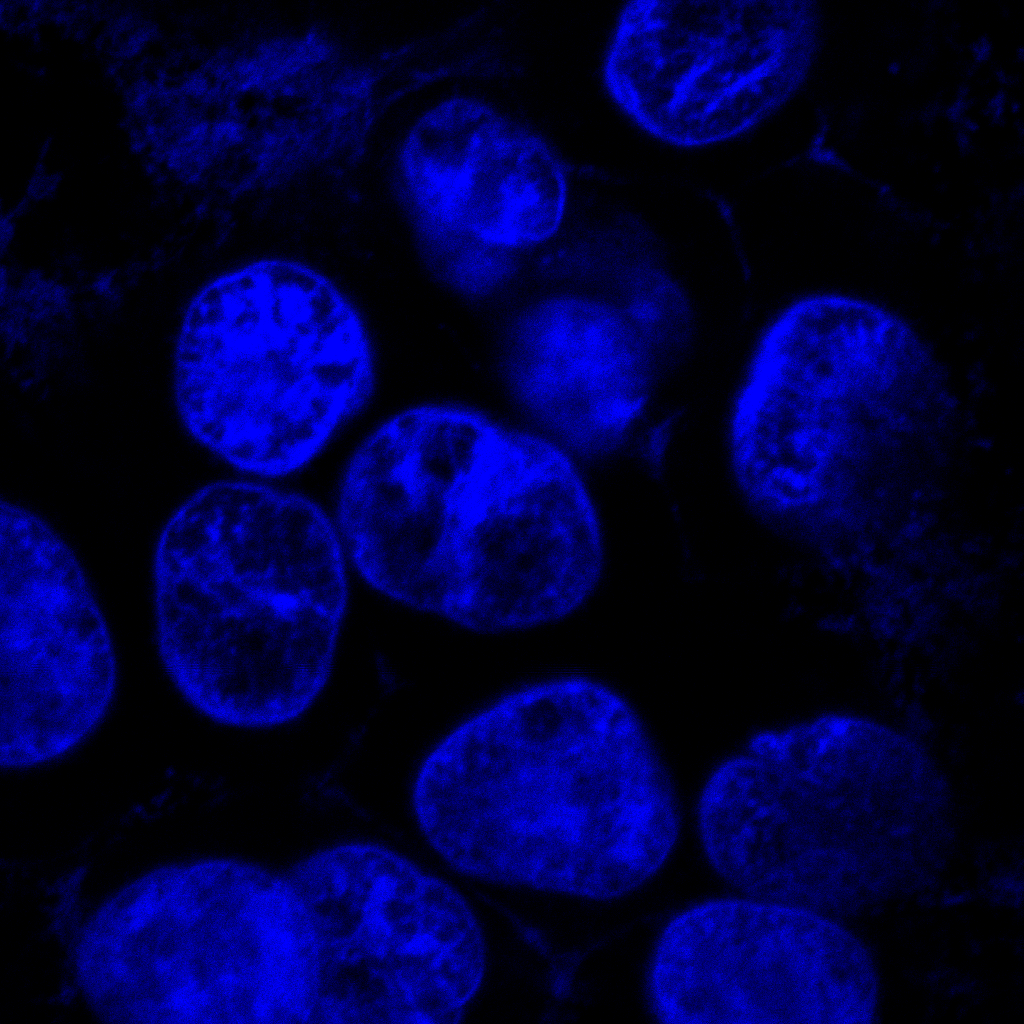

Supplement: Supplementary file 16 — Figure EV6 Source Data [file 44318_2024_353_MOESM16_ESM.zip › EVFigure 6/6E/Hep3B siCtrl+Vector/HP_3B SINC+V 60X3-1_RGB_C1.tif]

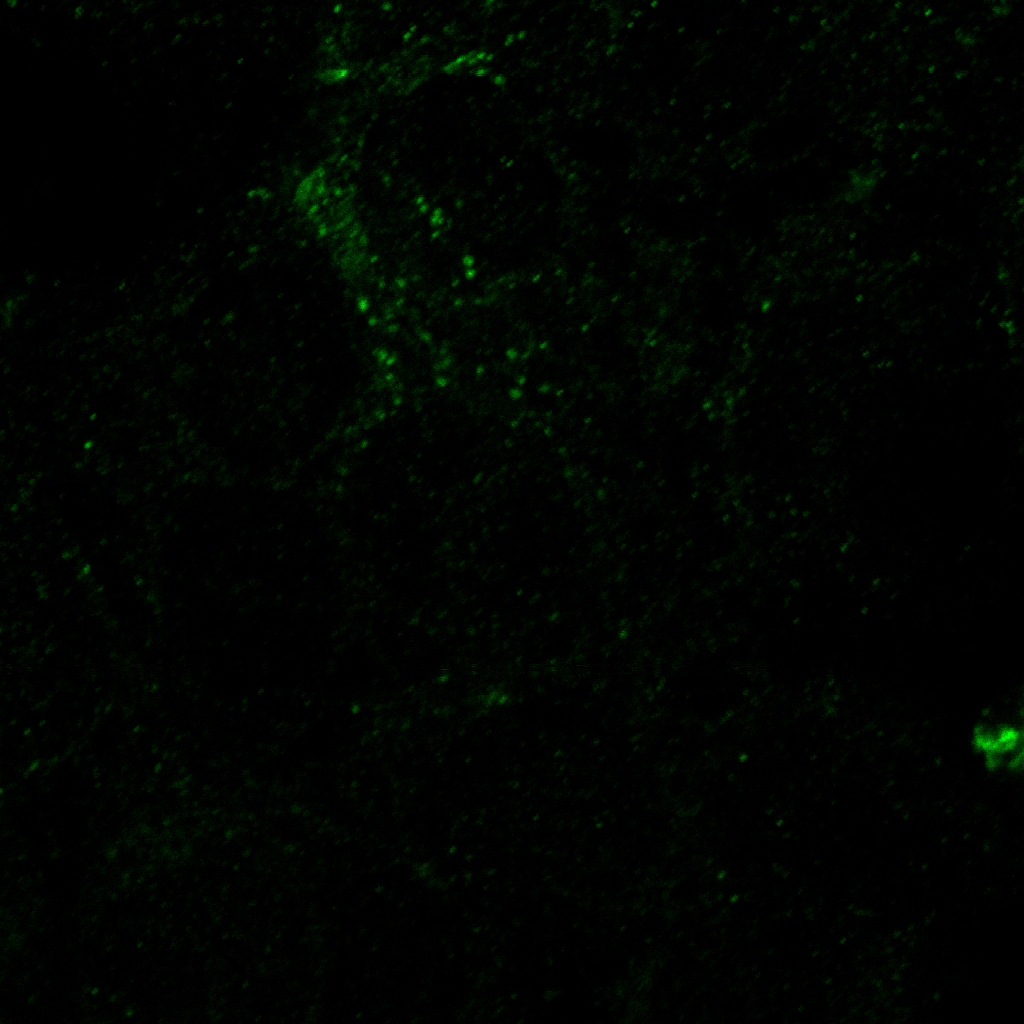

Supplement: Supplementary file 16 — Figure EV6 Source Data [file 44318_2024_353_MOESM16_ESM.zip › EVFigure 6/6E/Hep3B siCtrl+Vector/HP_3B SINC+V 60X3-1_RGB_C2.tif]

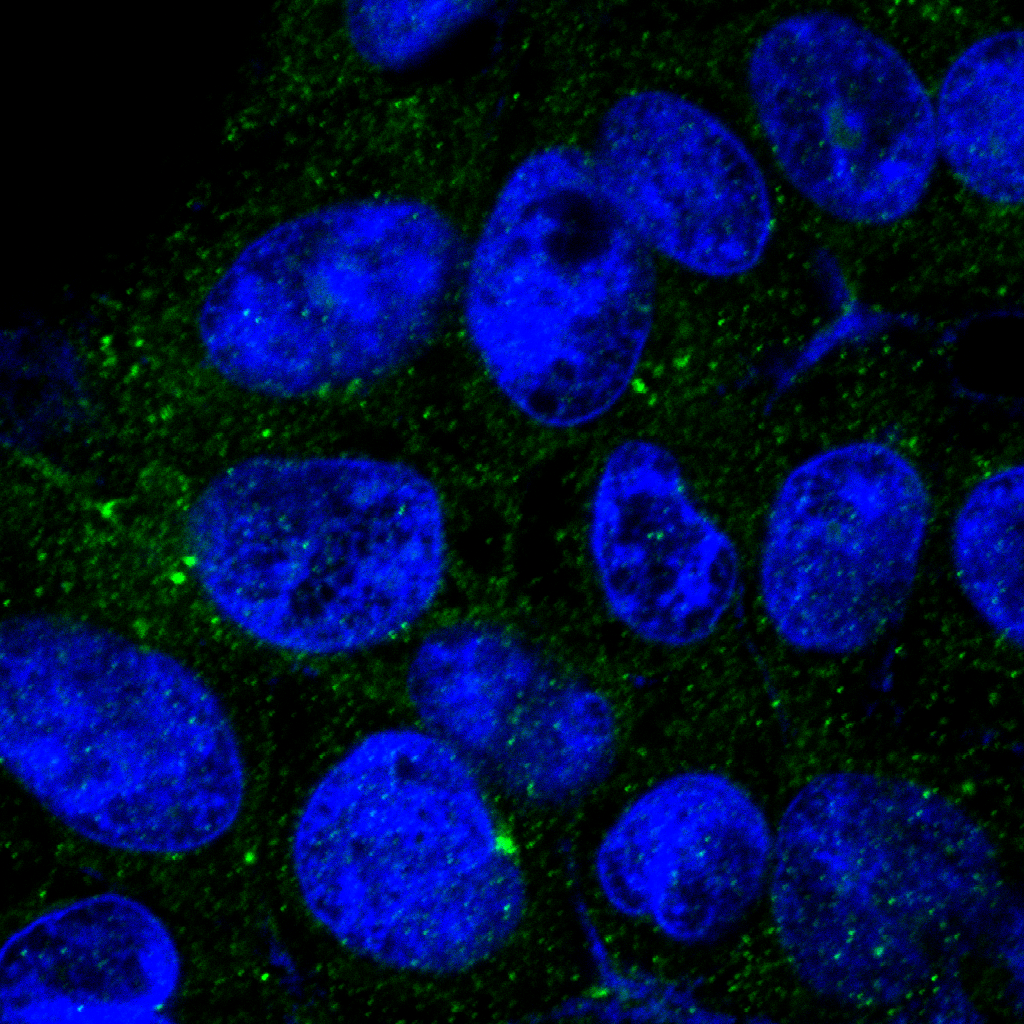

Supplement: Supplementary file 16 — Figure EV6 Source Data [file 44318_2024_353_MOESM16_ESM.zip › EVFigure 6/6E/Hep3B siCtrl+WTRHEB/HP_3B SINC+WTRHEB 60X3-3_RGB.tif]

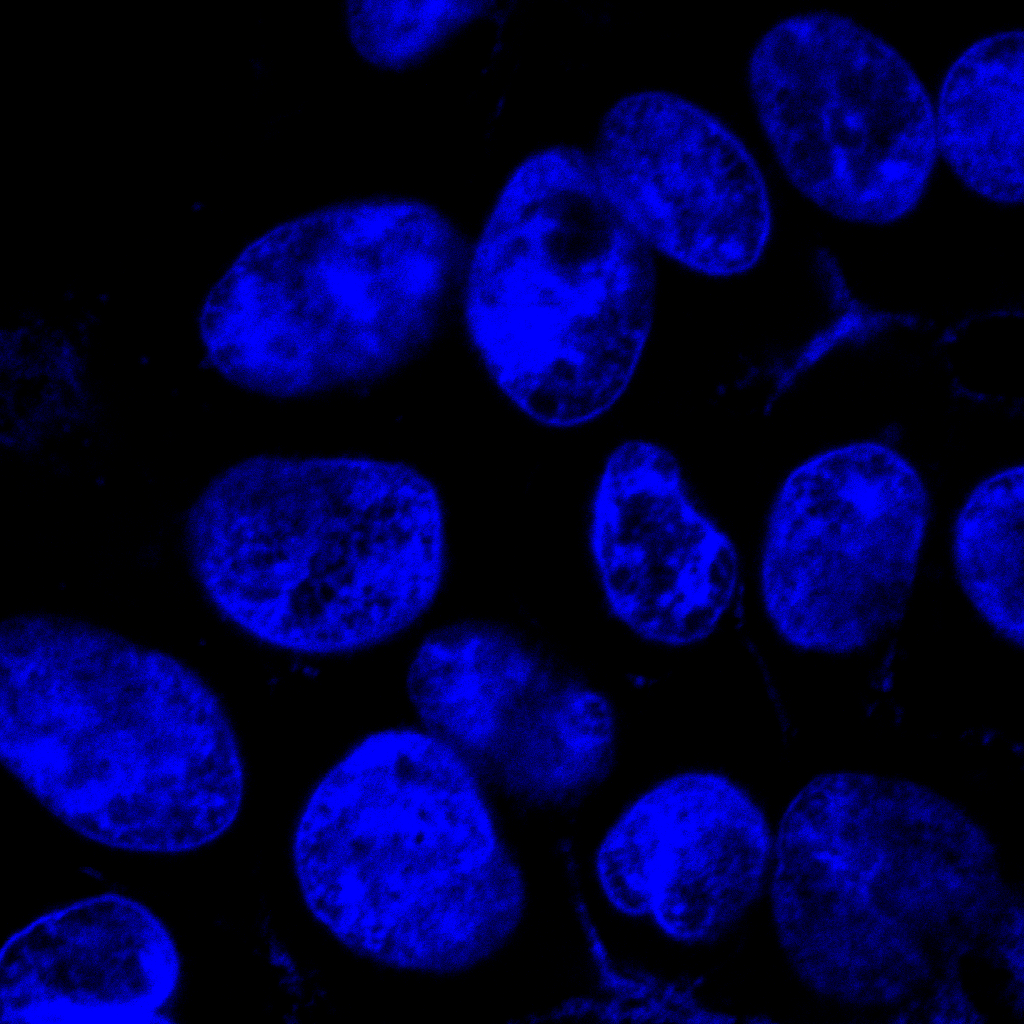

Supplement: Supplementary file 16 — Figure EV6 Source Data [file 44318_2024_353_MOESM16_ESM.zip › EVFigure 6/6E/Hep3B siCtrl+WTRHEB/HP_3B SINC+WTRHEB 60X3-3_RGB_C1.tif]

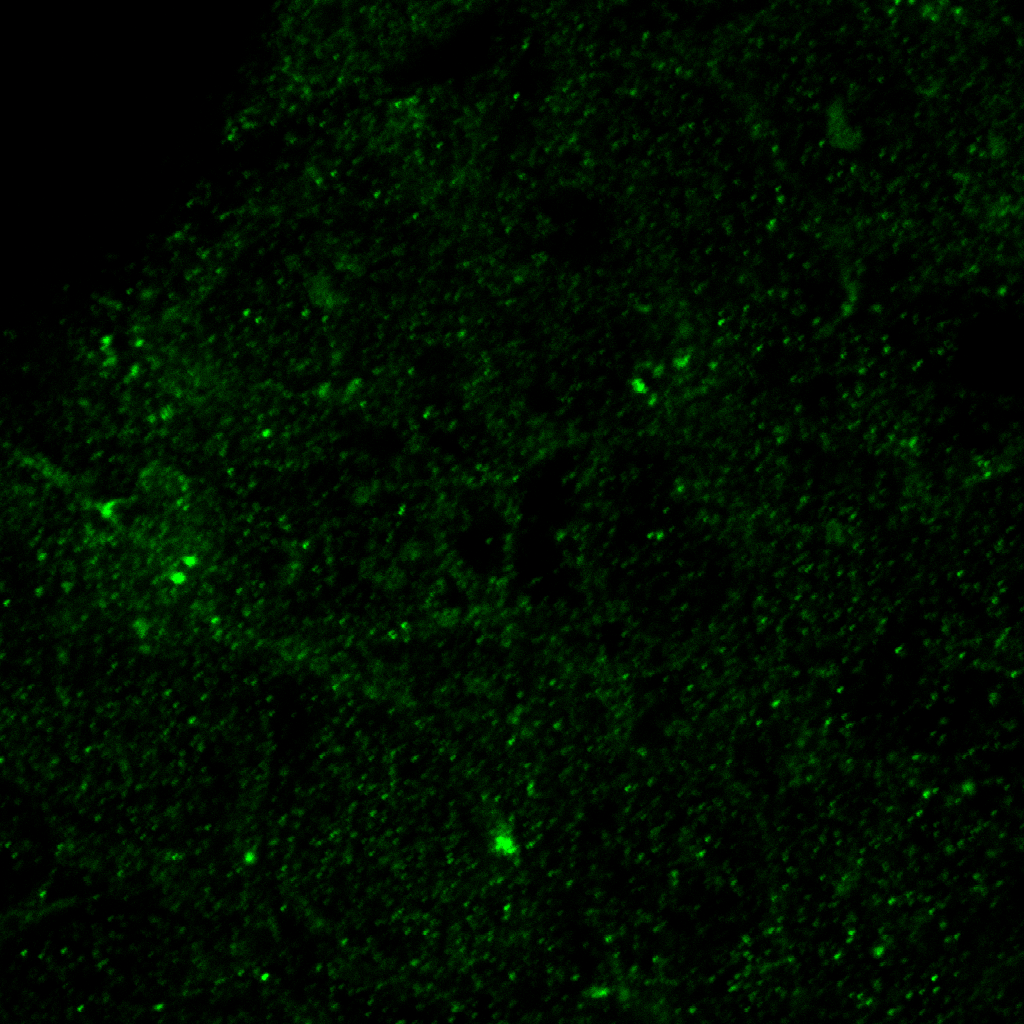

Supplement: Supplementary file 16 — Figure EV6 Source Data [file 44318_2024_353_MOESM16_ESM.zip › EVFigure 6/6E/Hep3B siCtrl+WTRHEB/HP_3B SINC+WTRHEB 60X3-3_RGB_C2.tif]

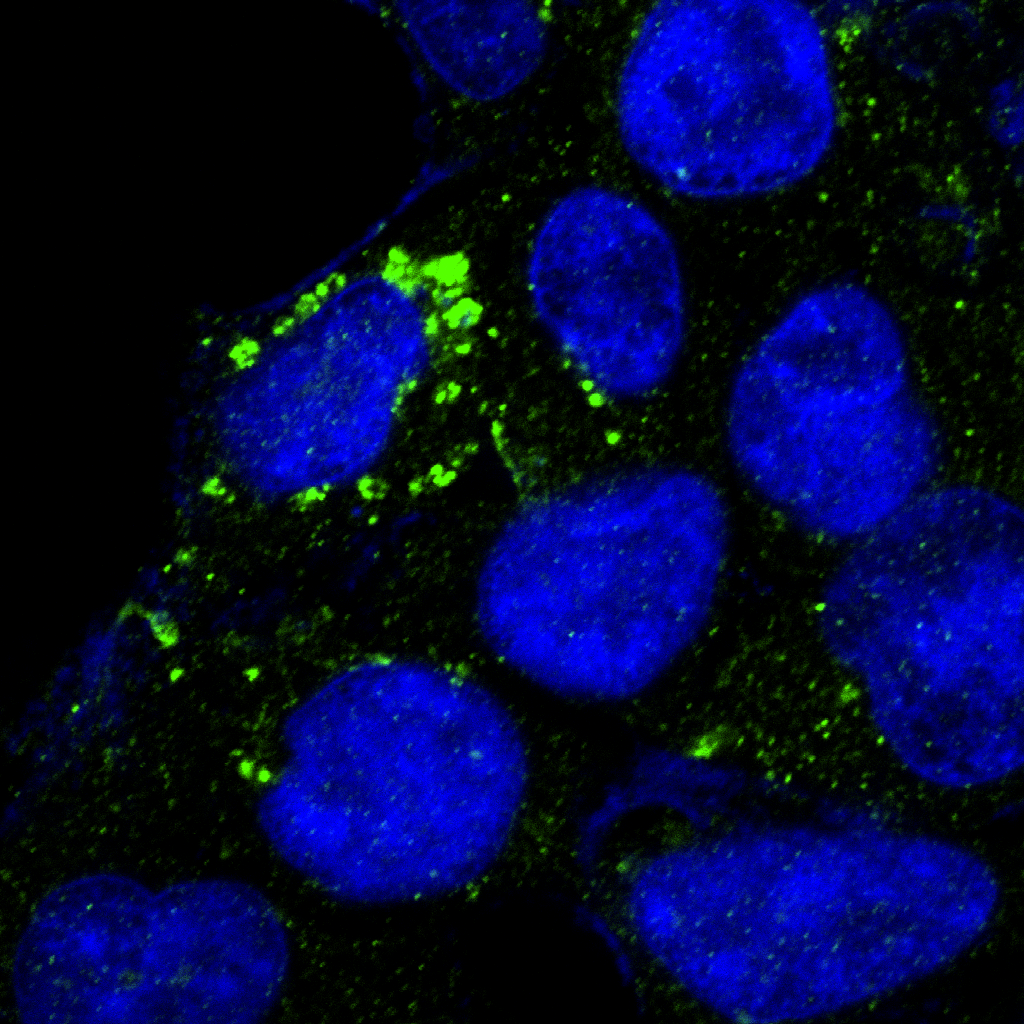

Supplement: Supplementary file 16 — Figure EV6 Source Data [file 44318_2024_353_MOESM16_ESM.zip › EVFigure 6/6E/Hep3B siSAG+RHEB-K169R/HP_3B SISAG+RHEBK169R 60X3-4_RGB.tif]

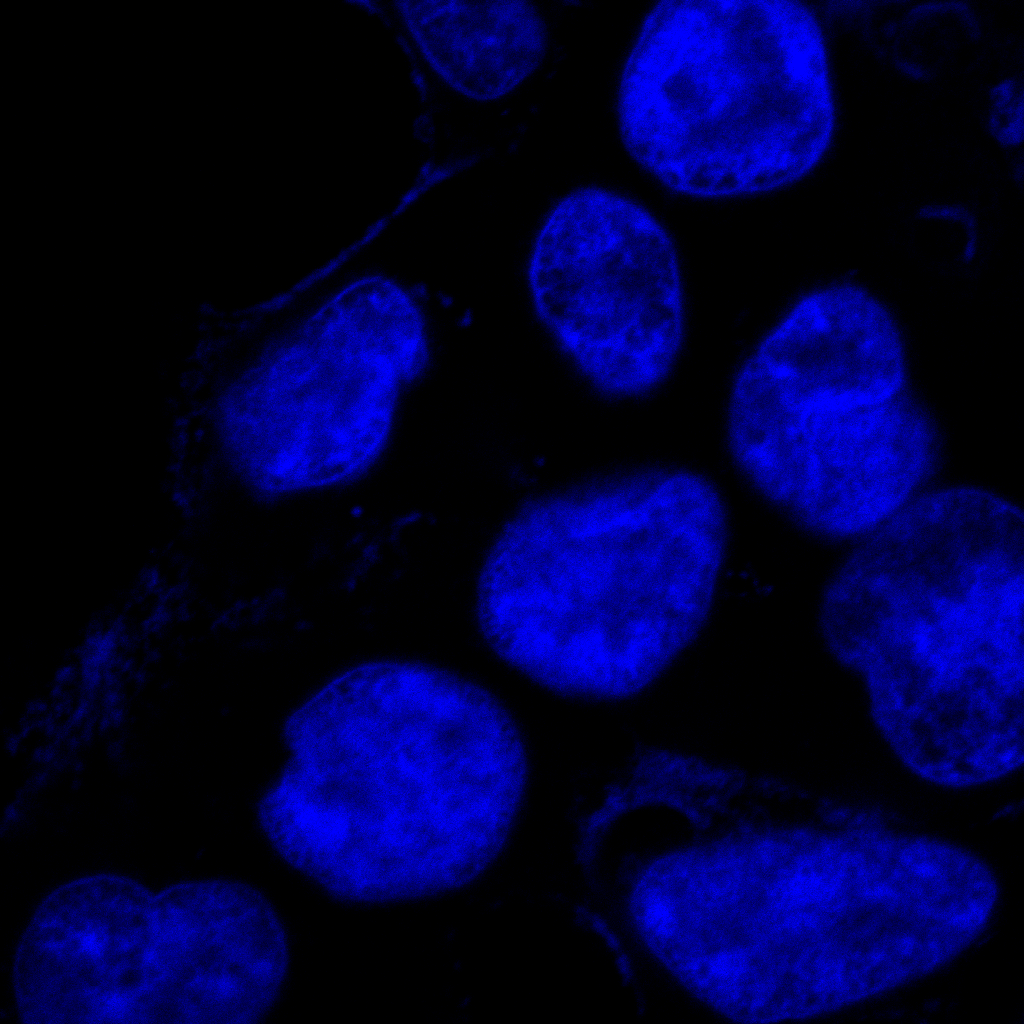

Supplement: Supplementary file 16 — Figure EV6 Source Data [file 44318_2024_353_MOESM16_ESM.zip › EVFigure 6/6E/Hep3B siSAG+RHEB-K169R/HP_3B SISAG+RHEBK169R 60X3-4_RGB_C1.tif]

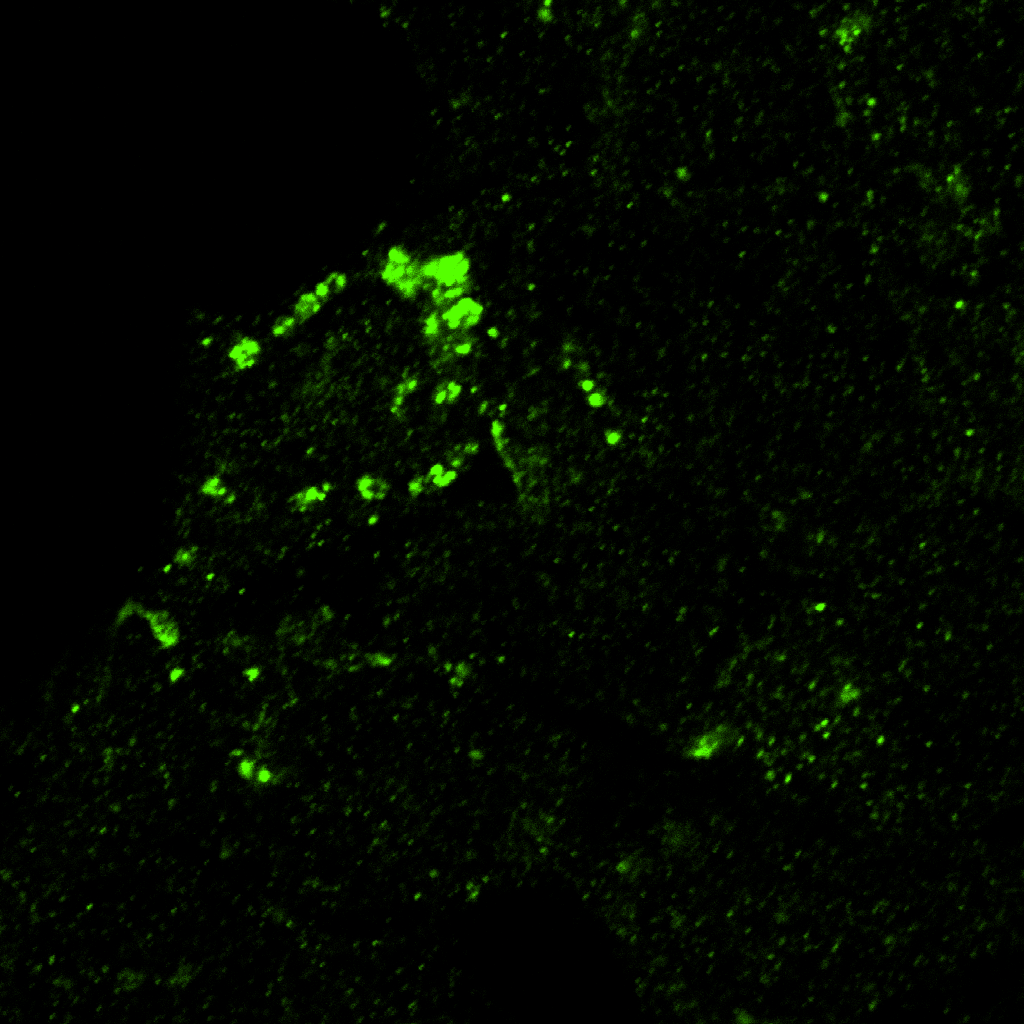

Supplement: Supplementary file 16 — Figure EV6 Source Data [file 44318_2024_353_MOESM16_ESM.zip › EVFigure 6/6E/Hep3B siSAG+RHEB-K169R/HP_3B SISAG+RHEBK169R 60X3-4_RGB_C2.tif]

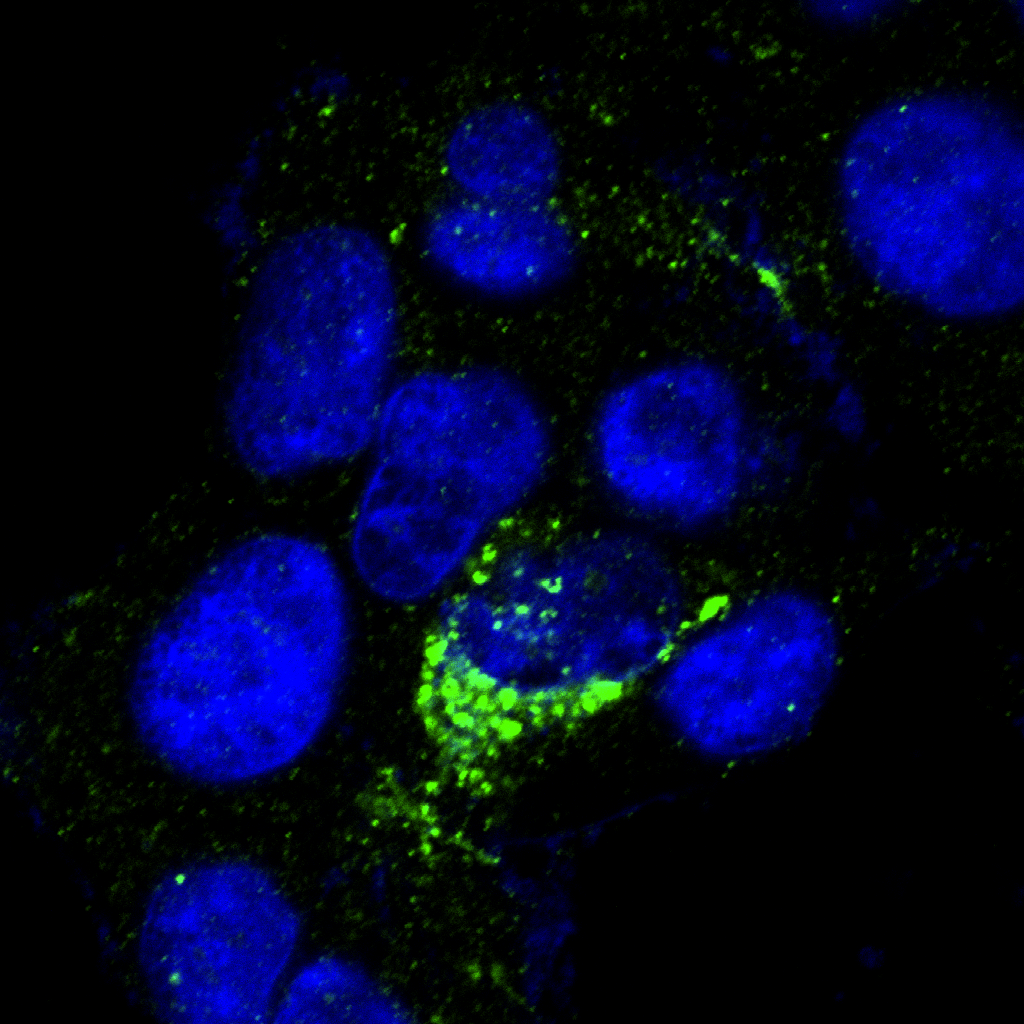

Supplement: Supplementary file 16 — Figure EV6 Source Data [file 44318_2024_353_MOESM16_ESM.zip › EVFigure 6/6E/Hep3B siSAG+Vector/HP_3B SISAG+V 60X3-2_RGB.tif]

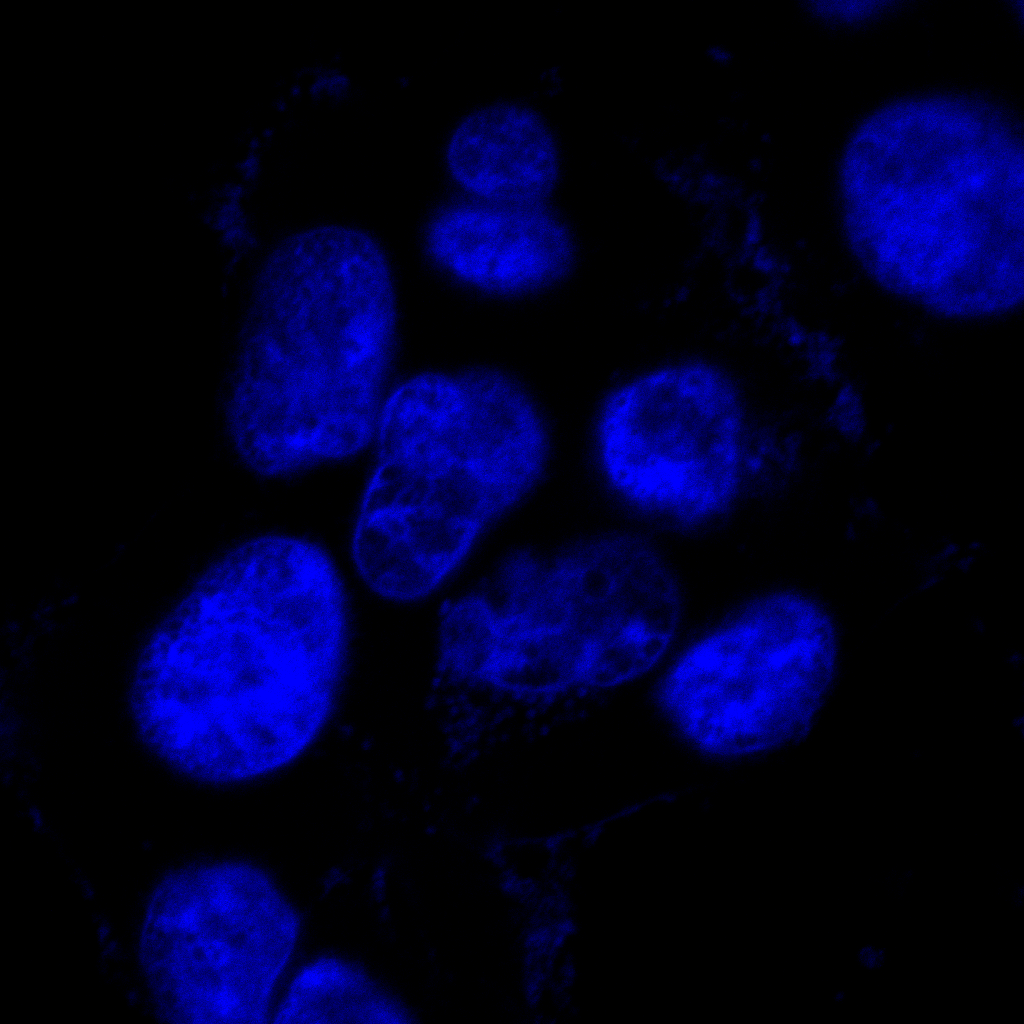

Supplement: Supplementary file 16 — Figure EV6 Source Data [file 44318_2024_353_MOESM16_ESM.zip › EVFigure 6/6E/Hep3B siSAG+Vector/HP_3B SISAG+V 60X3-2_RGB_C1.tif]

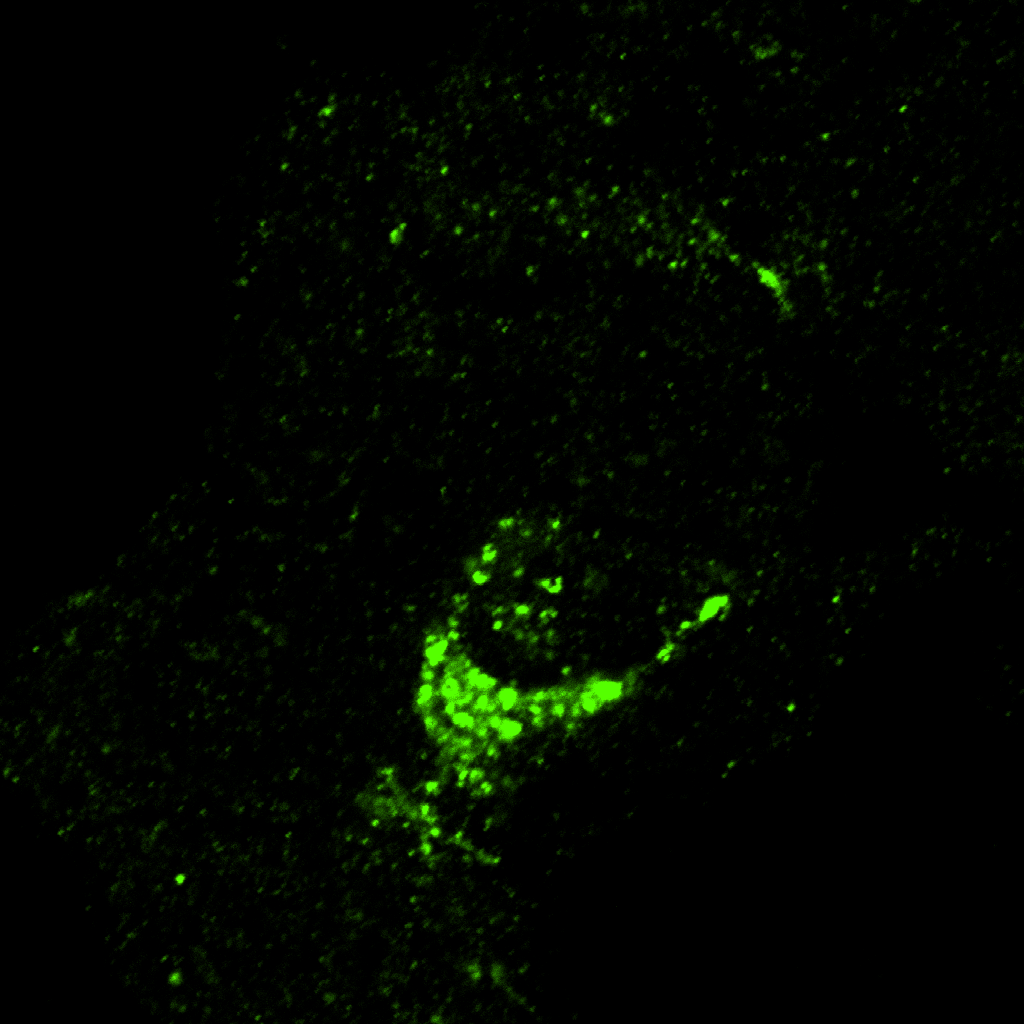

Supplement: Supplementary file 16 — Figure EV6 Source Data [file 44318_2024_353_MOESM16_ESM.zip › EVFigure 6/6E/Hep3B siSAG+Vector/HP_3B SISAG+V 60X3-2_RGB_C2.tif]

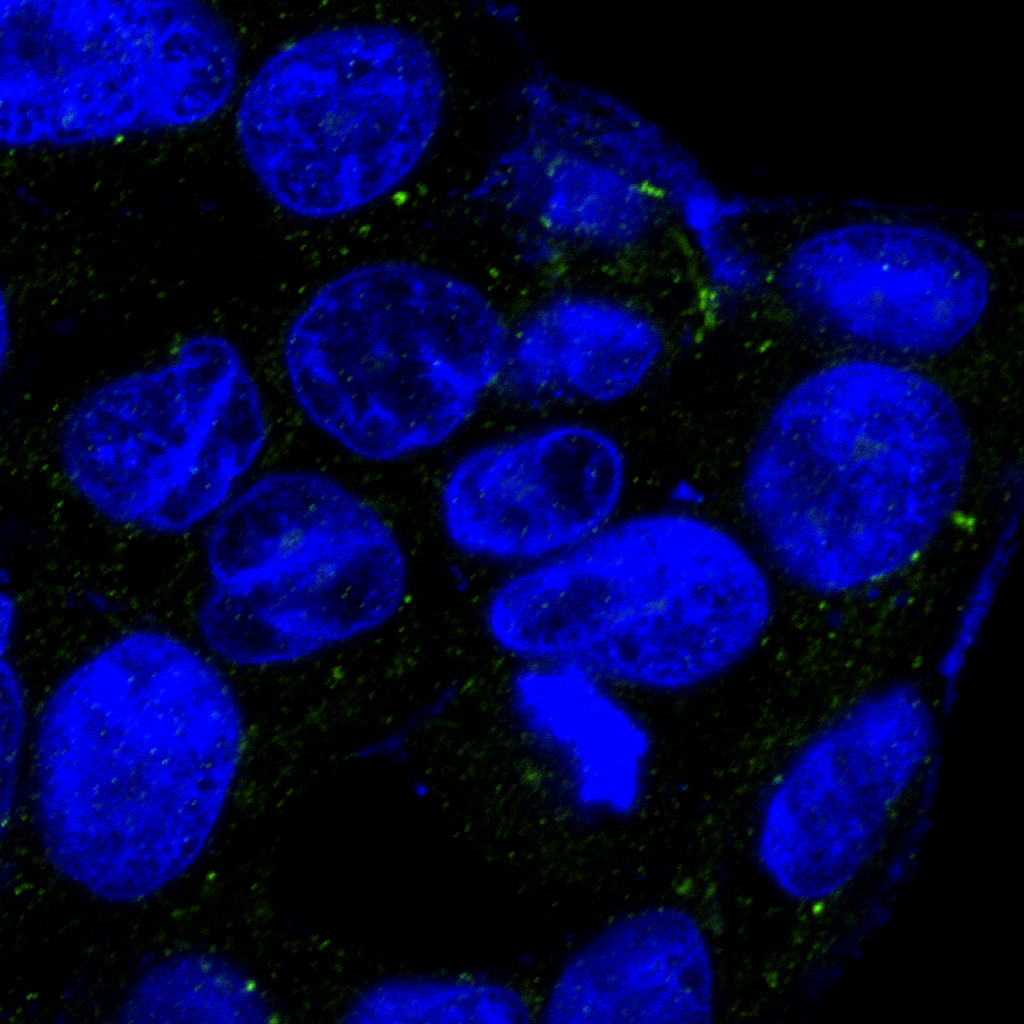

Supplement: Supplementary file 16 — Figure EV6 Source Data [file 44318_2024_353_MOESM16_ESM.zip › EVFigure 6/6E/Hep3B siSAG+WT-RHEB/HP_3B SISAG+WTRHEB 60X3-1_RGB.tif]

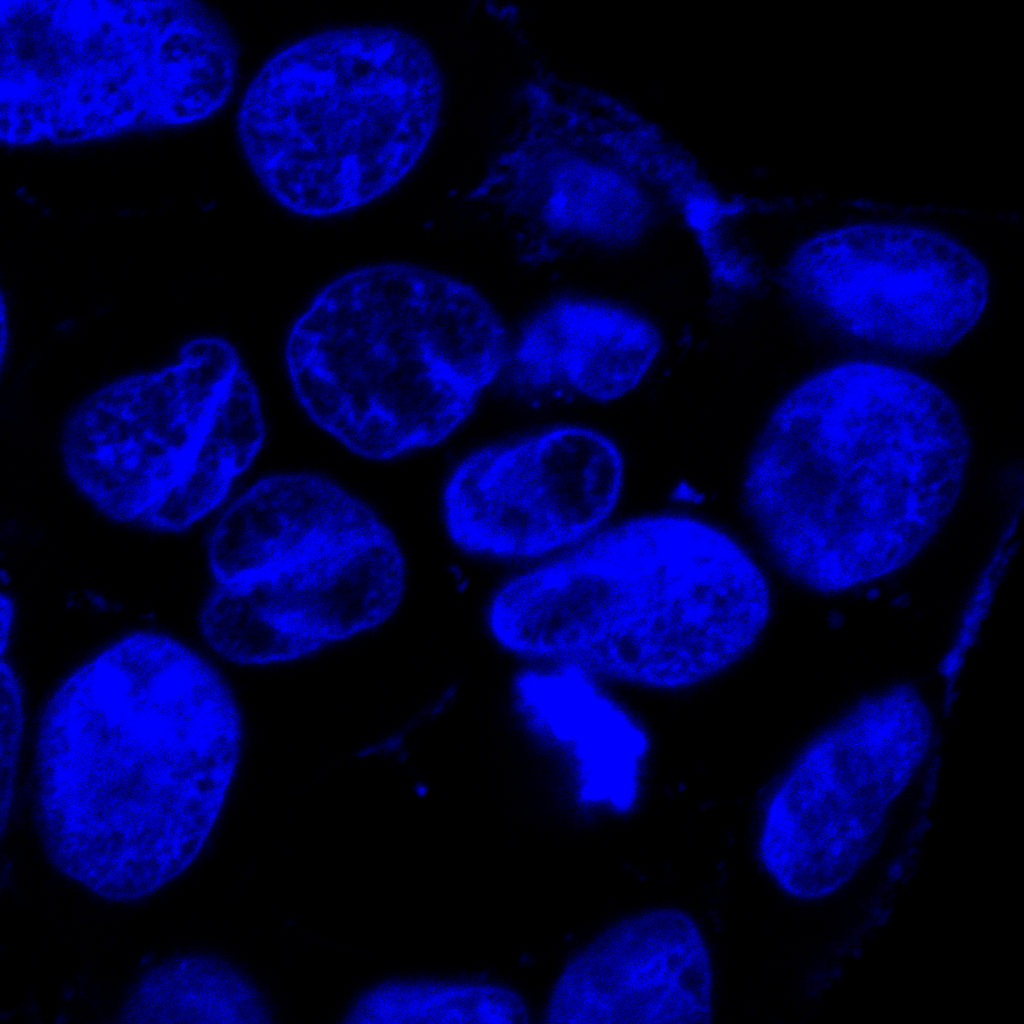

Supplement: Supplementary file 16 — Figure EV6 Source Data [file 44318_2024_353_MOESM16_ESM.zip › EVFigure 6/6E/Hep3B siSAG+WT-RHEB/HP_3B SISAG+WTRHEB 60X3-1_RGB_C1.tif]

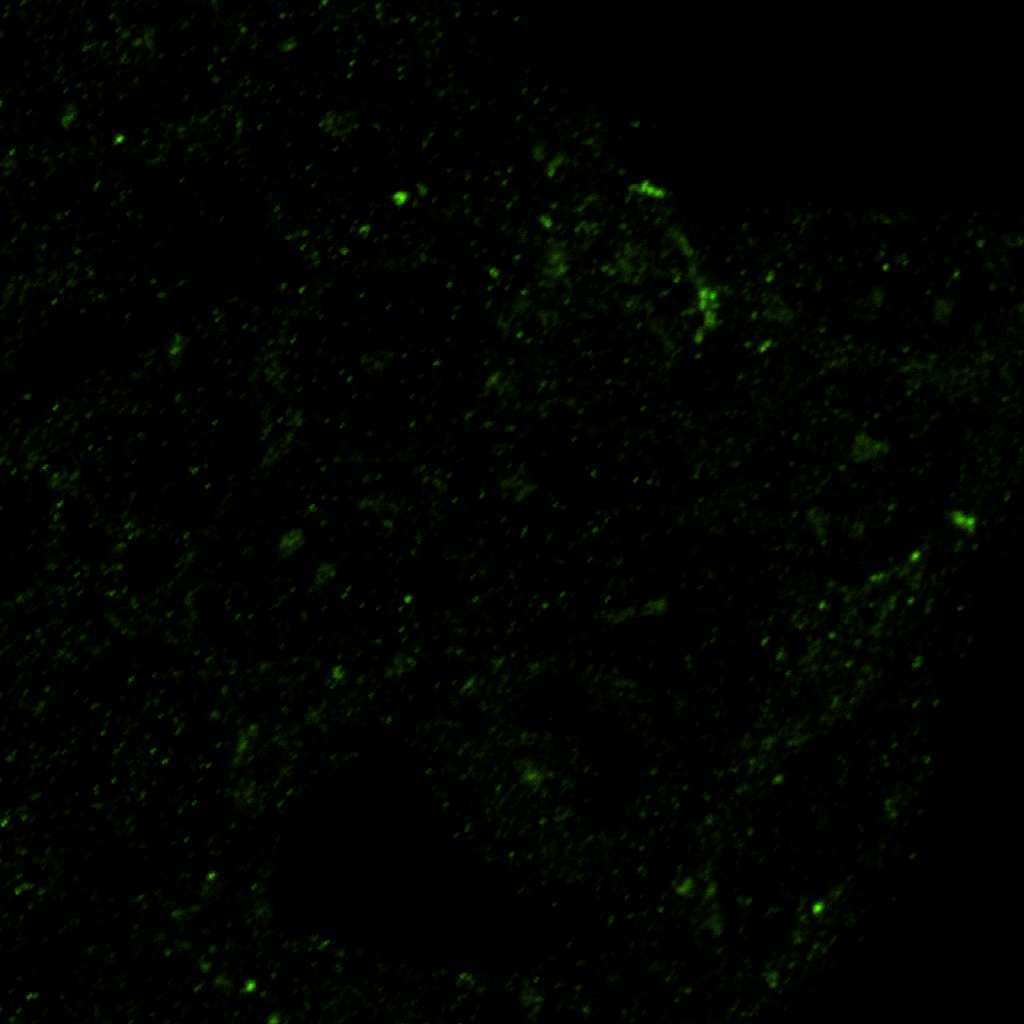

Supplement: Supplementary file 16 — Figure EV6 Source Data [file 44318_2024_353_MOESM16_ESM.zip › EVFigure 6/6E/Hep3B siSAG+WT-RHEB/HP_3B SISAG+WTRHEB 60X3-1_RGB_C2.tif]

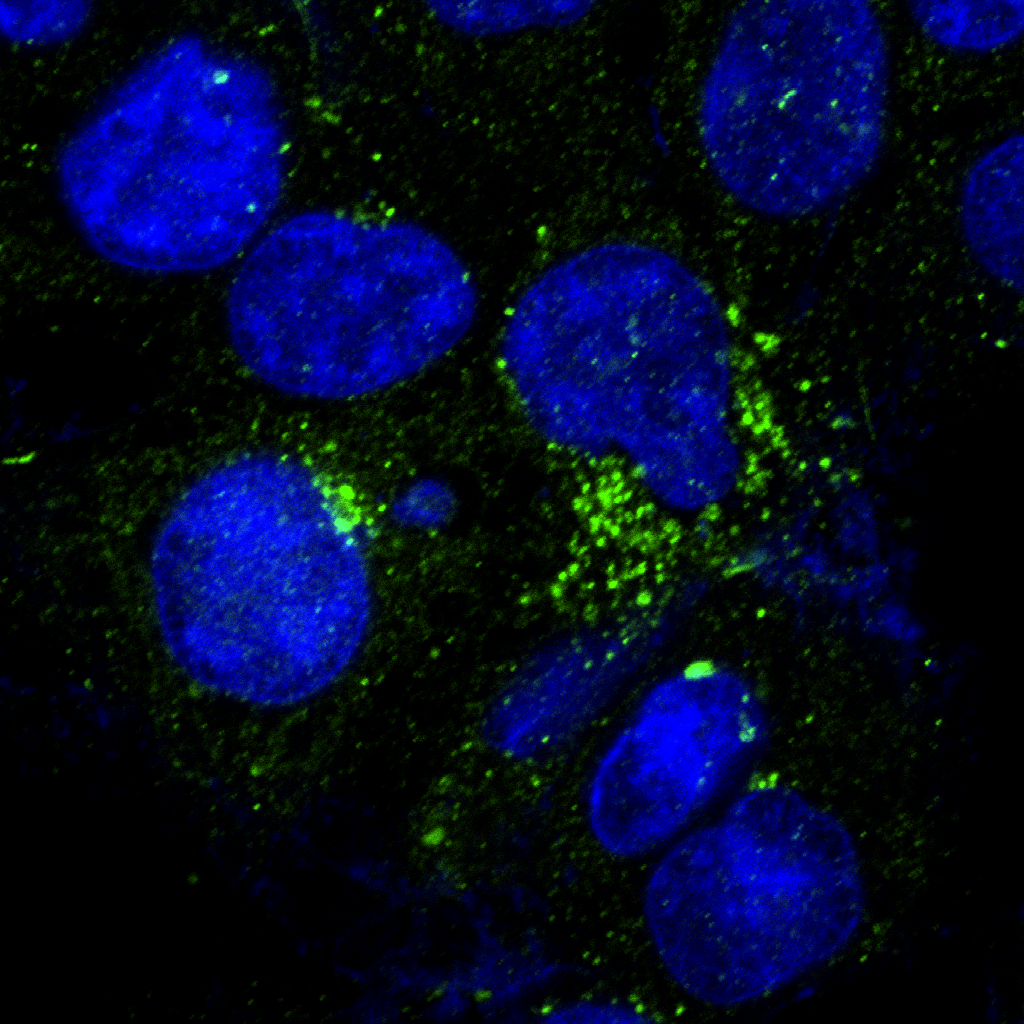

Supplement: Supplementary file 16 — Figure EV6 Source Data [file 44318_2024_353_MOESM16_ESM.zip › EVFigure 6/6E/Hep3B siUBE2F+RHEB-K169R/HP_3B SI2F+RHEBK169R 60X3-4_RGB.tif]

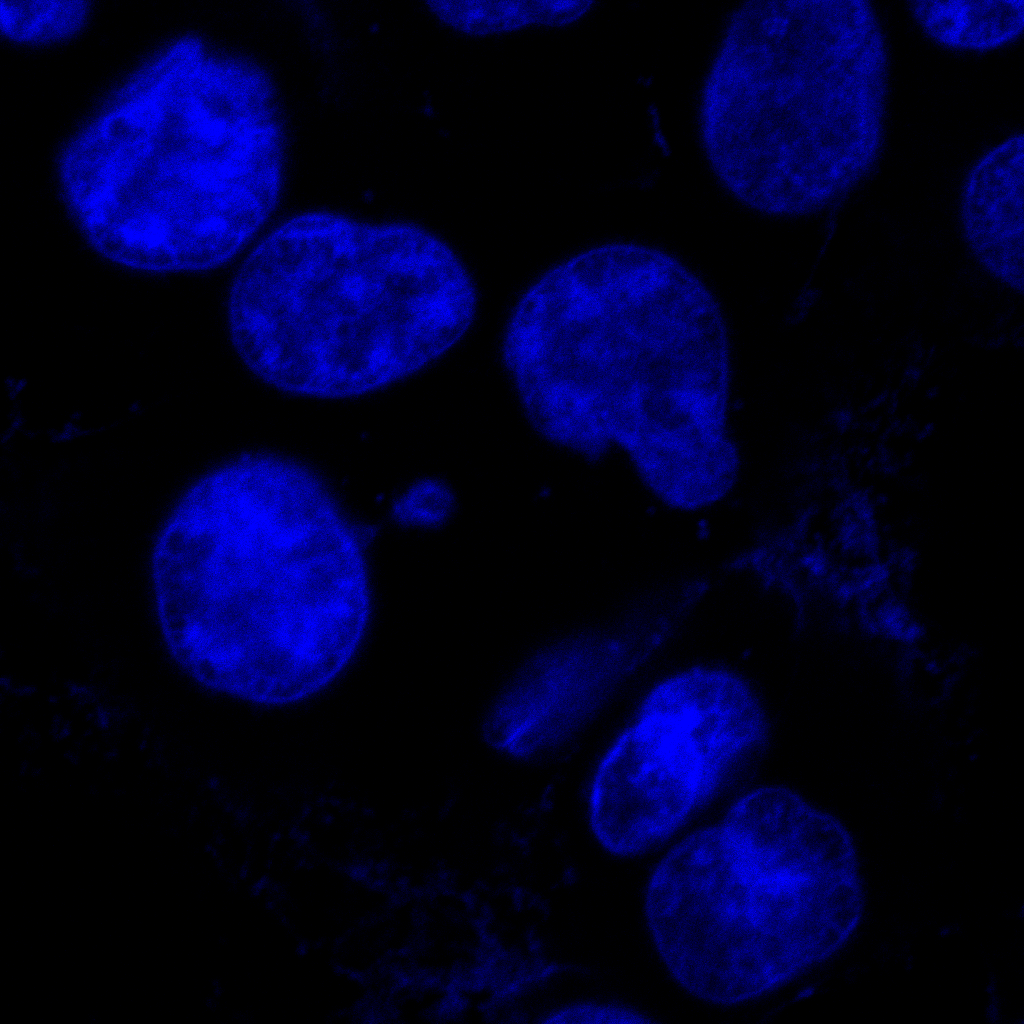

Supplement: Supplementary file 16 — Figure EV6 Source Data [file 44318_2024_353_MOESM16_ESM.zip › EVFigure 6/6E/Hep3B siUBE2F+RHEB-K169R/HP_3B SI2F+RHEBK169R 60X3-4_RGB_C1.tif]

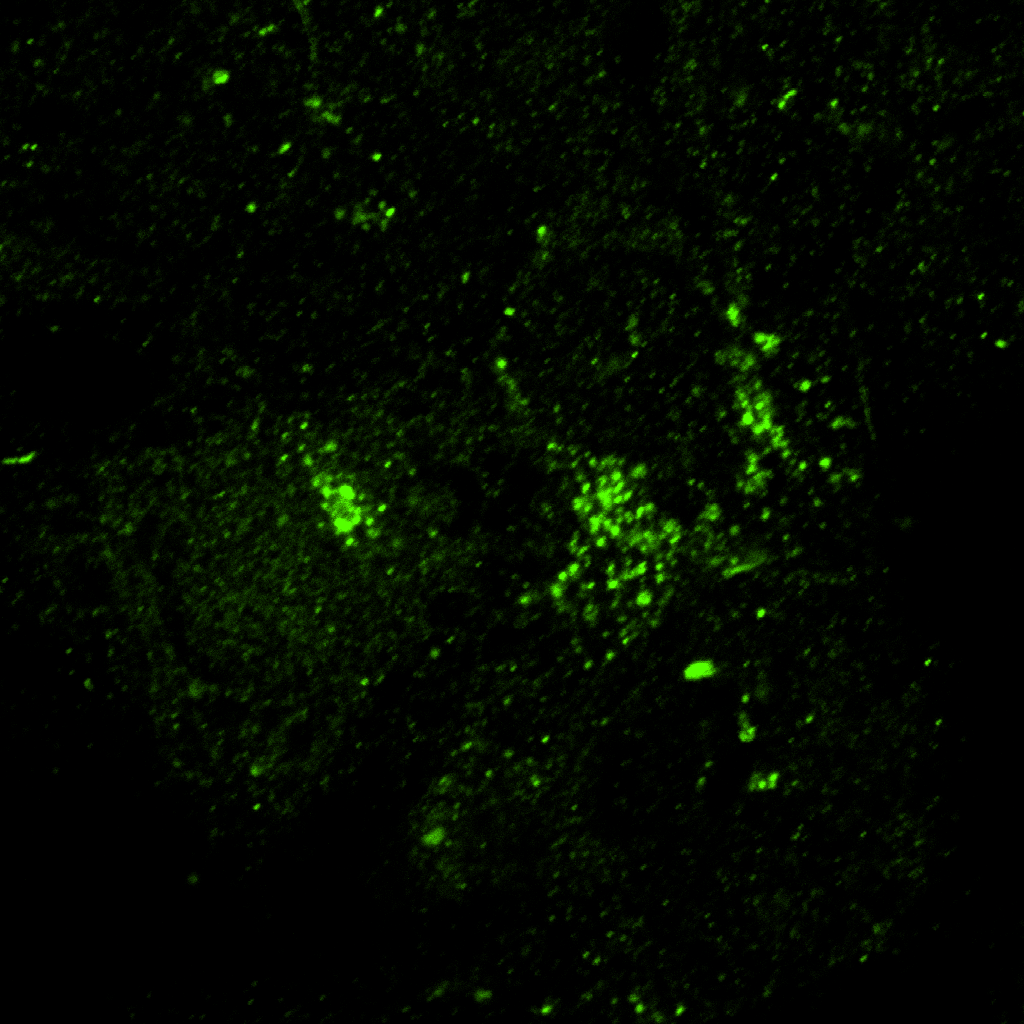

Supplement: Supplementary file 16 — Figure EV6 Source Data [file 44318_2024_353_MOESM16_ESM.zip › EVFigure 6/6E/Hep3B siUBE2F+RHEB-K169R/HP_3B SI2F+RHEBK169R 60X3-4_RGB_C2.tif]

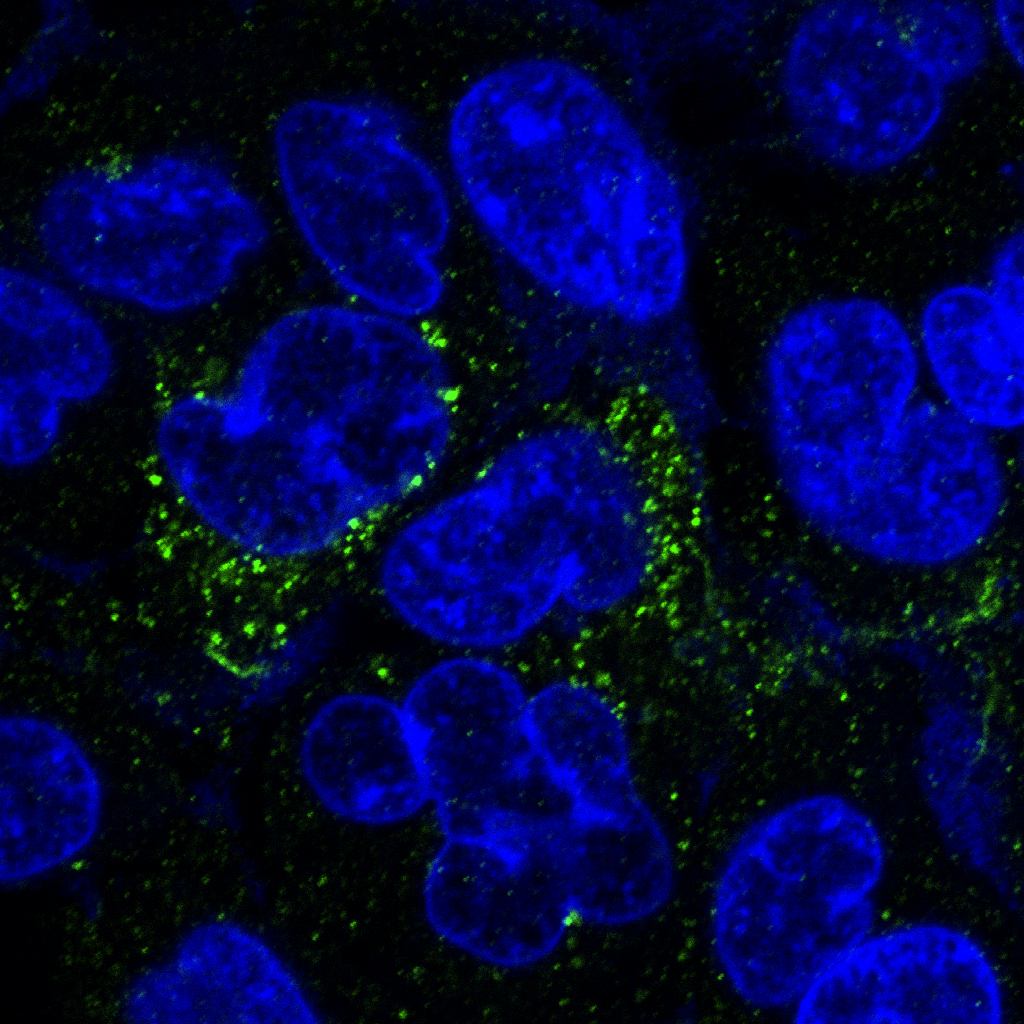

Supplement: Supplementary file 16 — Figure EV6 Source Data [file 44318_2024_353_MOESM16_ESM.zip › EVFigure 6/6E/Hep3B siUBE2F+Vector/HP_3B SI2F+V 60X3-5_RGB.tif]

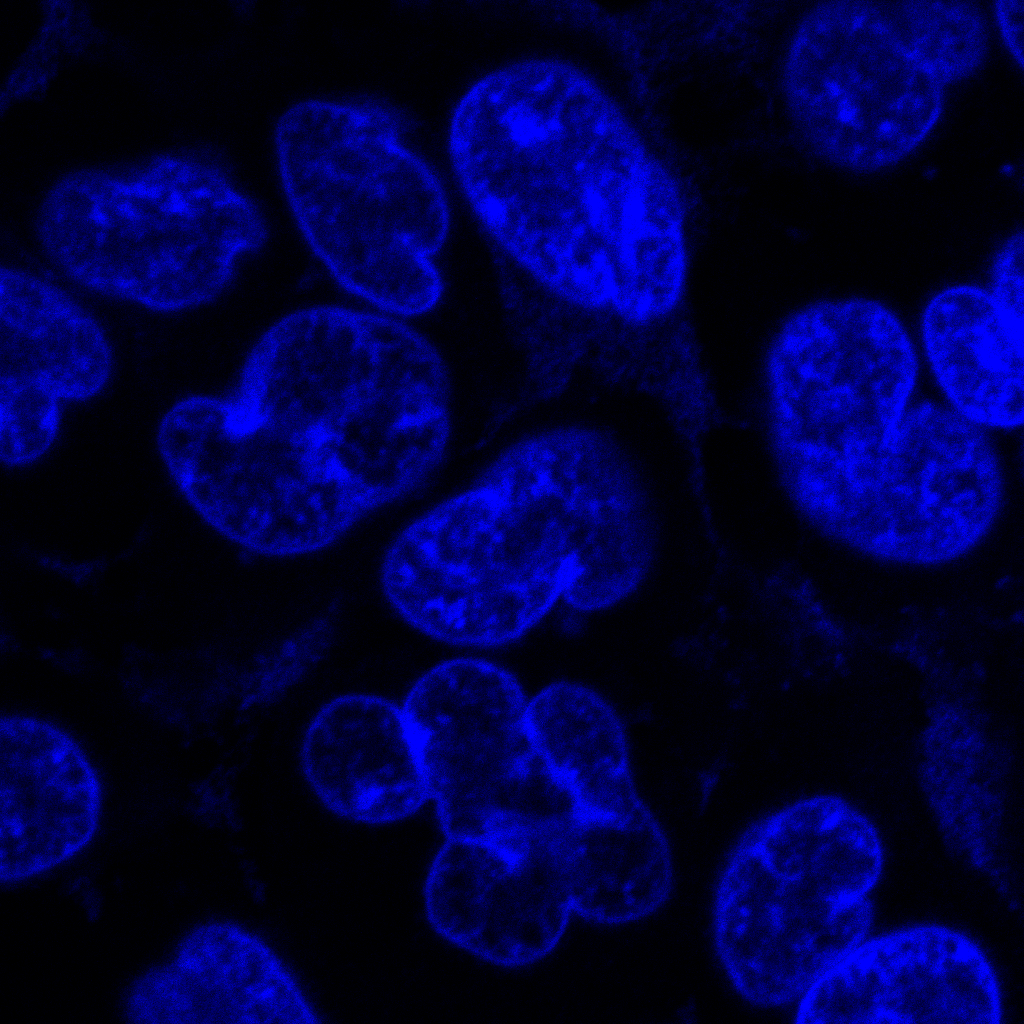

Supplement: Supplementary file 16 — Figure EV6 Source Data [file 44318_2024_353_MOESM16_ESM.zip › EVFigure 6/6E/Hep3B siUBE2F+Vector/HP_3B SI2F+V 60X3-5_RGB_C1.tif]

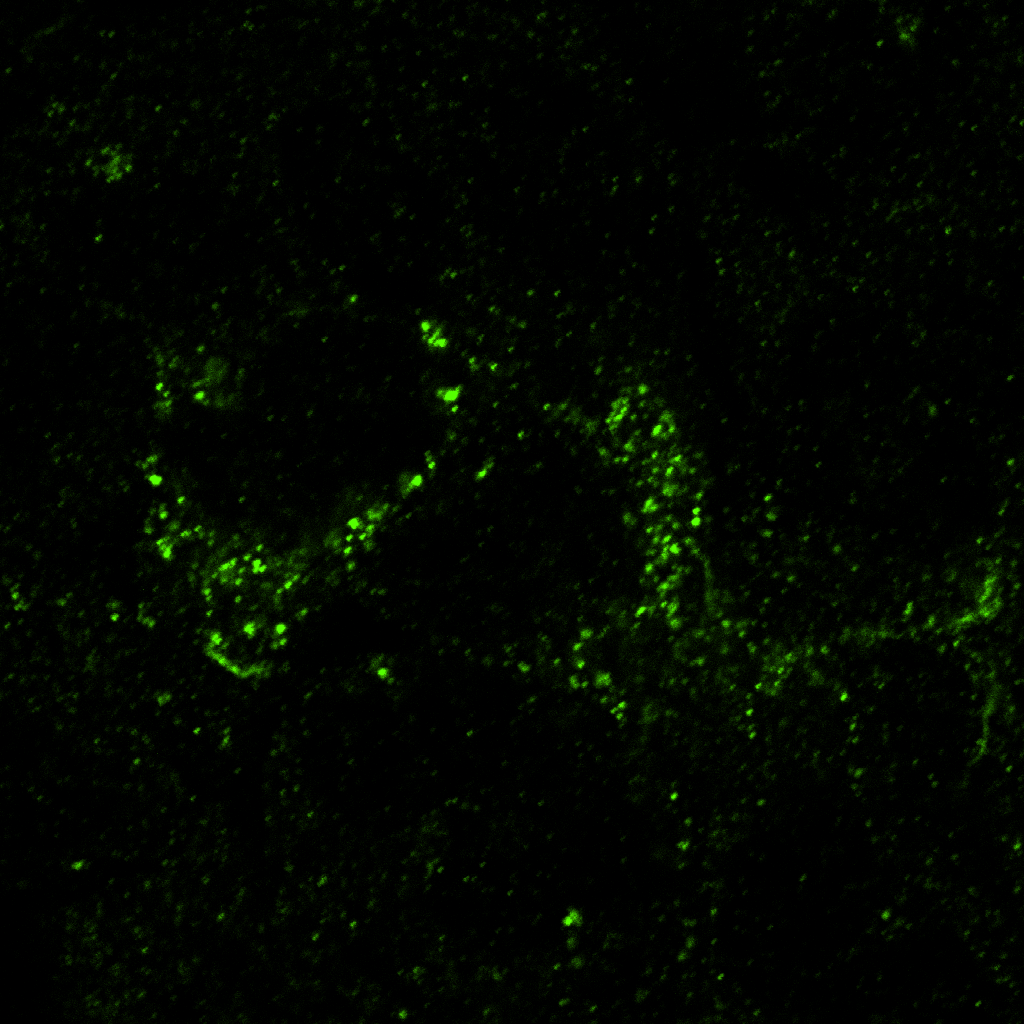

Supplement: Supplementary file 16 — Figure EV6 Source Data [file 44318_2024_353_MOESM16_ESM.zip › EVFigure 6/6E/Hep3B siUBE2F+Vector/HP_3B SI2F+V 60X3-5_RGB_C2.tif]

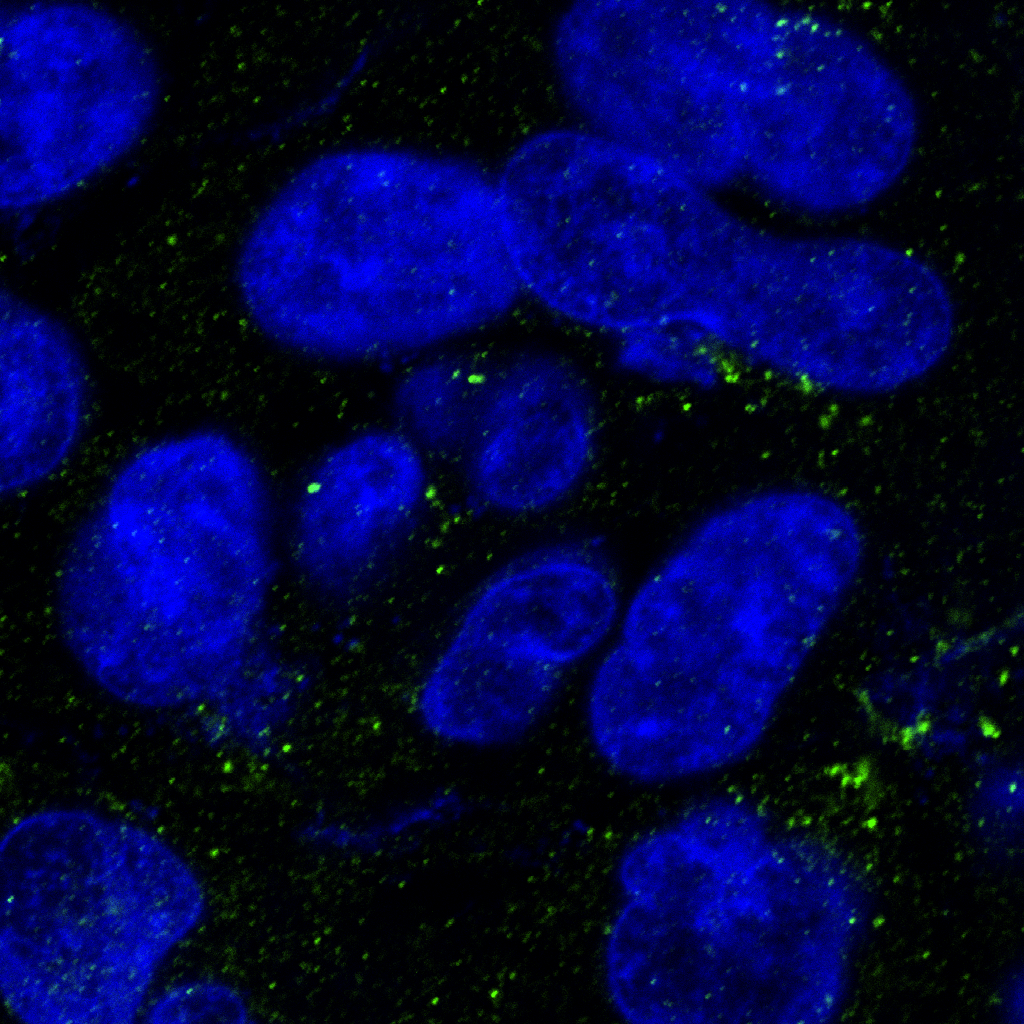

Supplement: Supplementary file 16 — Figure EV6 Source Data [file 44318_2024_353_MOESM16_ESM.zip › EVFigure 6/6E/Hep3B siUBE2F+WTRHEB/HP_3B SI2F+WTRHEB 60X3-4_RGB.tif]

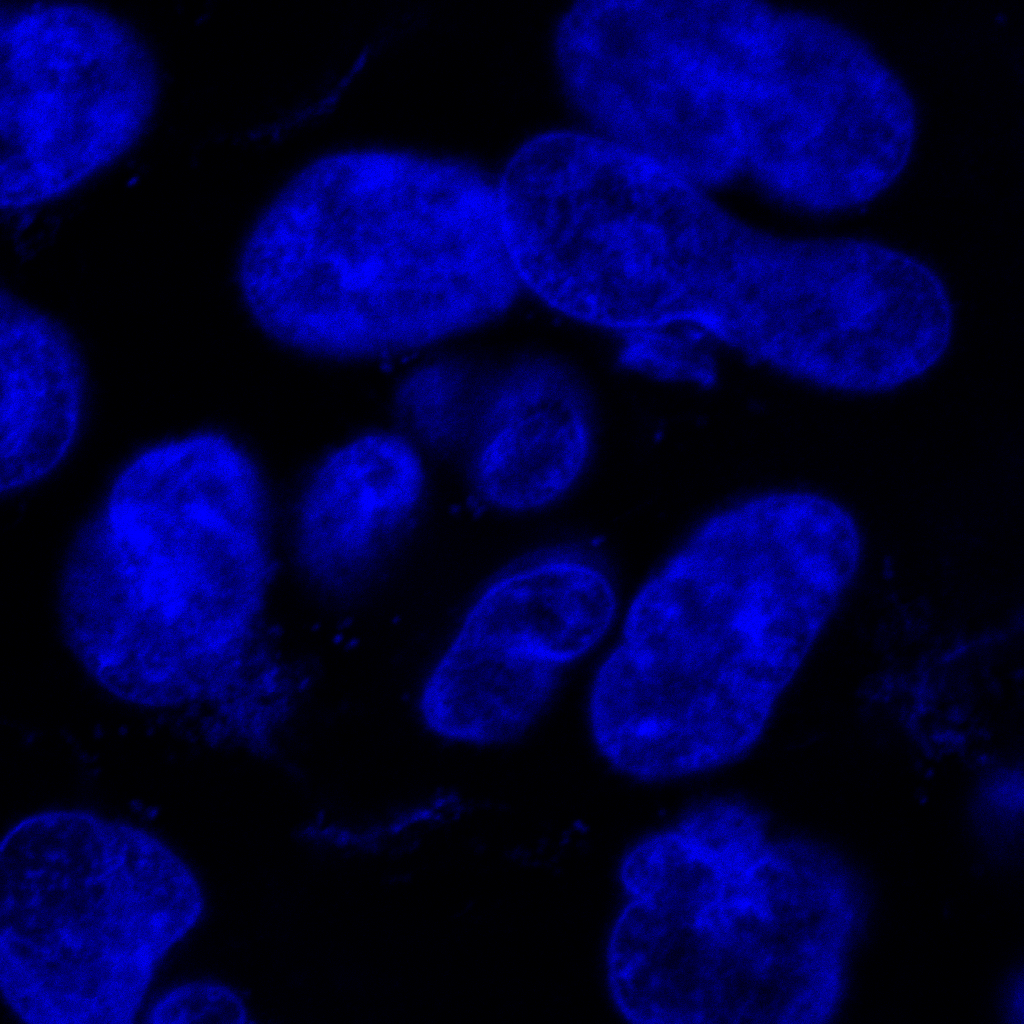

Supplement: Supplementary file 16 — Figure EV6 Source Data [file 44318_2024_353_MOESM16_ESM.zip › EVFigure 6/6E/Hep3B siUBE2F+WTRHEB/HP_3B SI2F+WTRHEB 60X3-4_RGB_C1.tif]

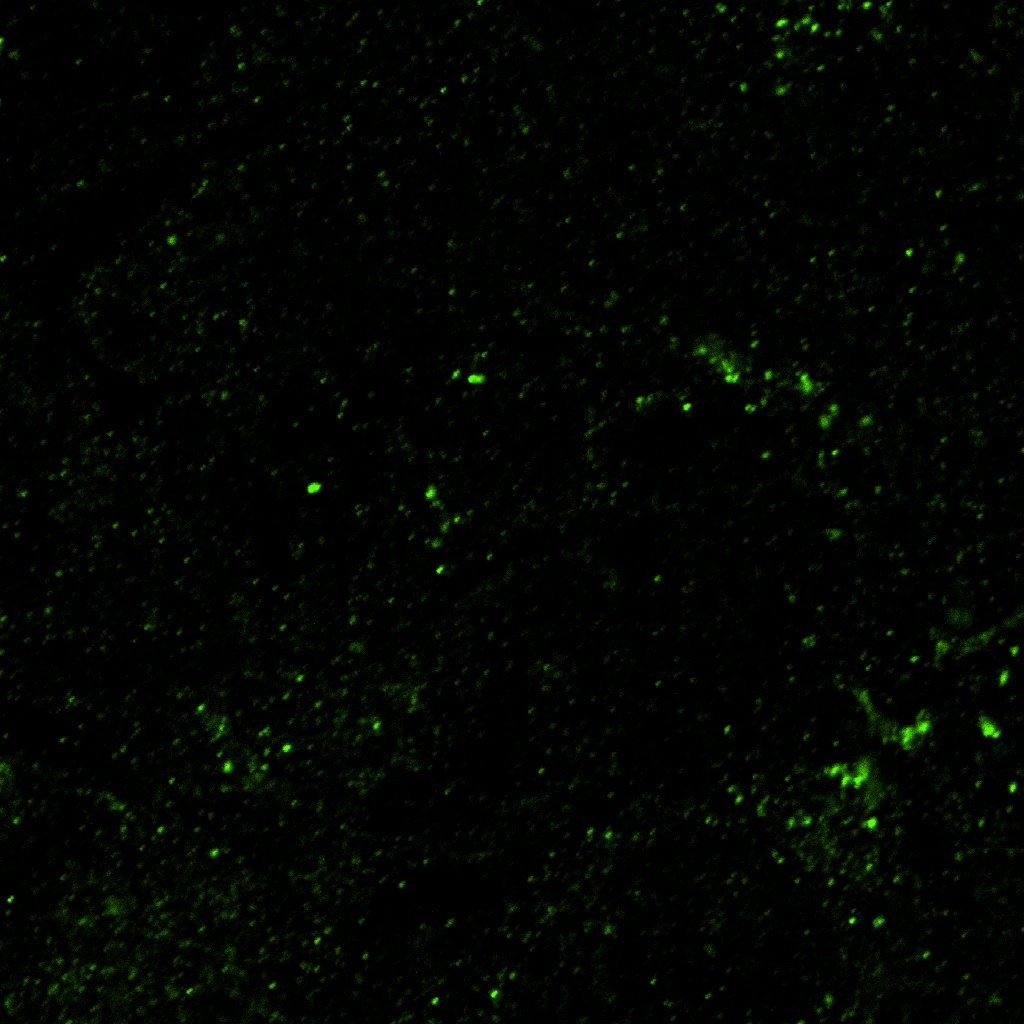

Supplement: Supplementary file 16 — Figure EV6 Source Data [file 44318_2024_353_MOESM16_ESM.zip › EVFigure 6/6E/Hep3B siUBE2F+WTRHEB/HP_3B SI2F+WTRHEB 60X3-4_RGB_C2.tif]

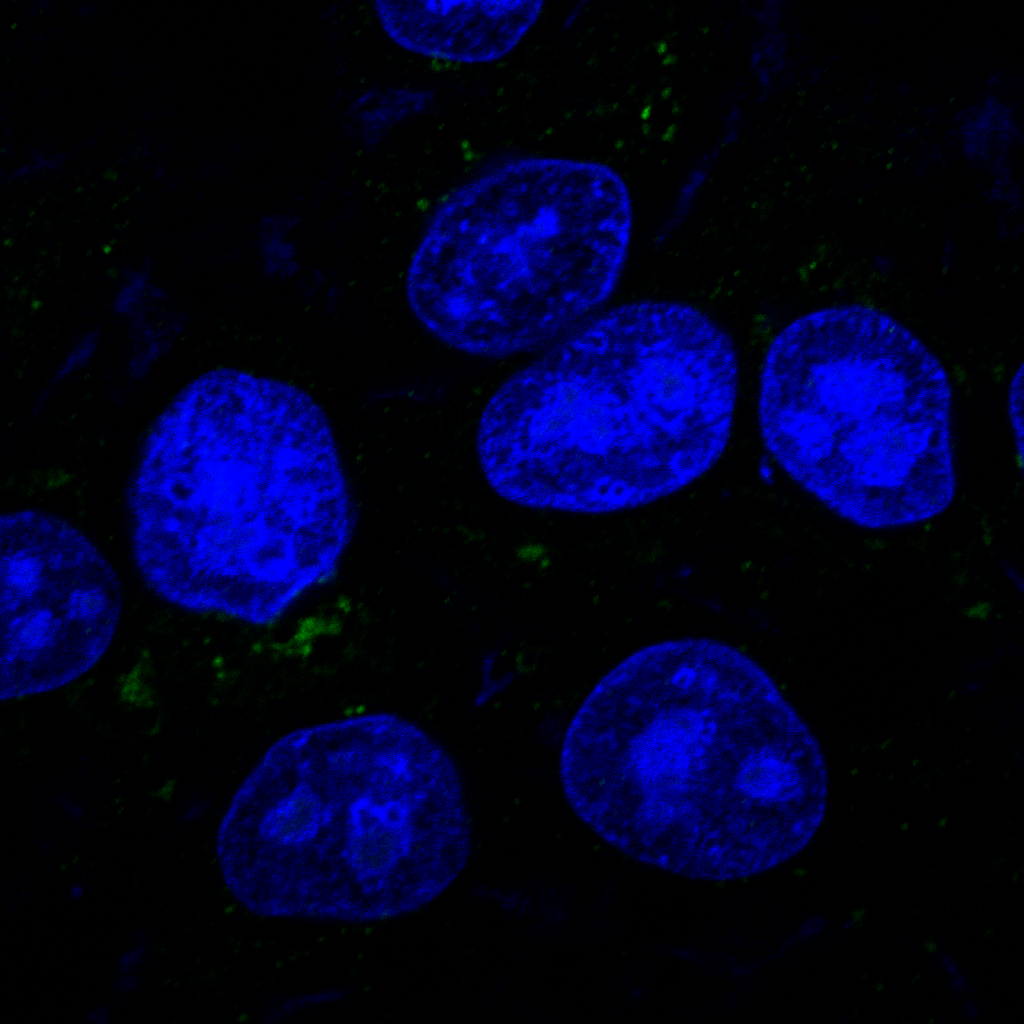

Supplement: Supplementary file 16 — Figure EV6 Source Data [file 44318_2024_353_MOESM16_ESM.zip › EVFigure 6/6G/PLCPRF5 siCtrl+RHEB-K169R/HP_PLC SINC+K169R 60X3-1_RGB.tif]

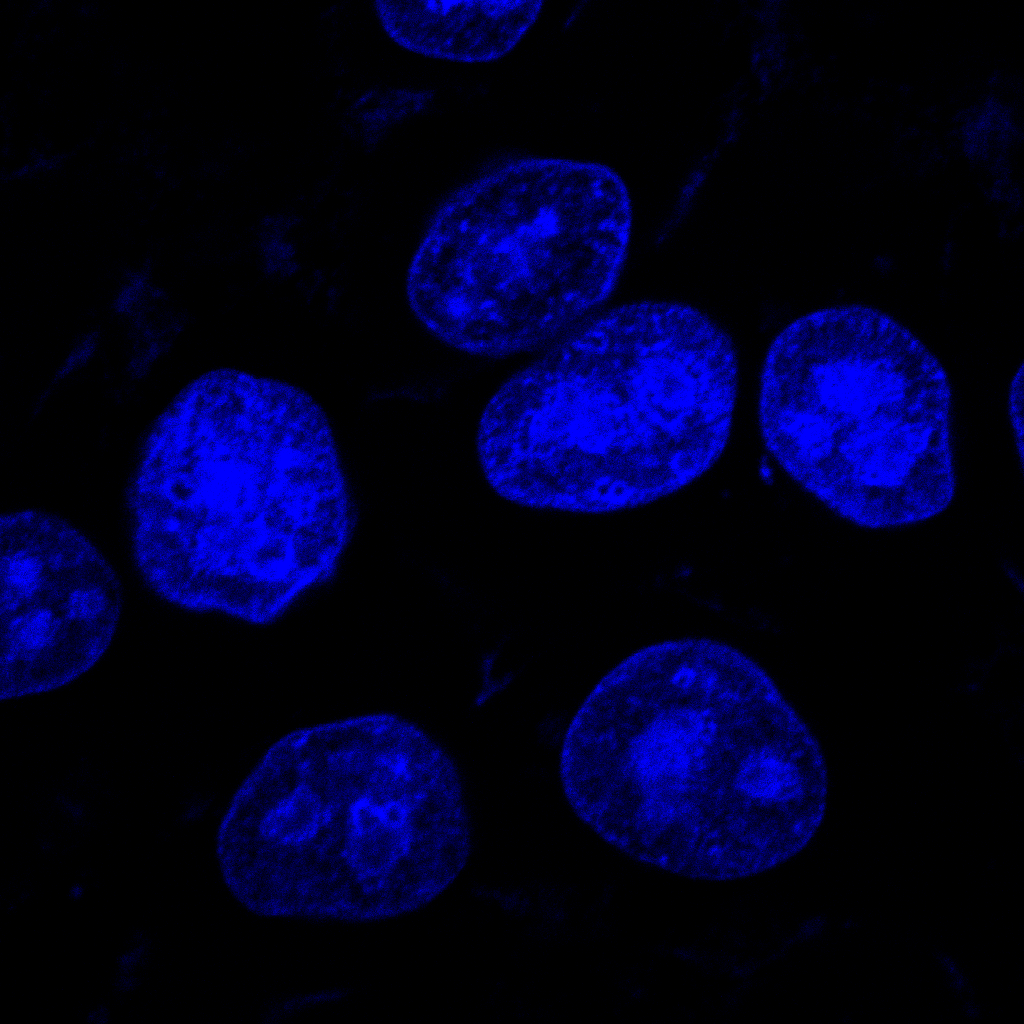

Supplement: Supplementary file 16 — Figure EV6 Source Data [file 44318_2024_353_MOESM16_ESM.zip › EVFigure 6/6G/PLCPRF5 siCtrl+RHEB-K169R/HP_PLC SINC+K169R 60X3-1_RGB_C1.tif]

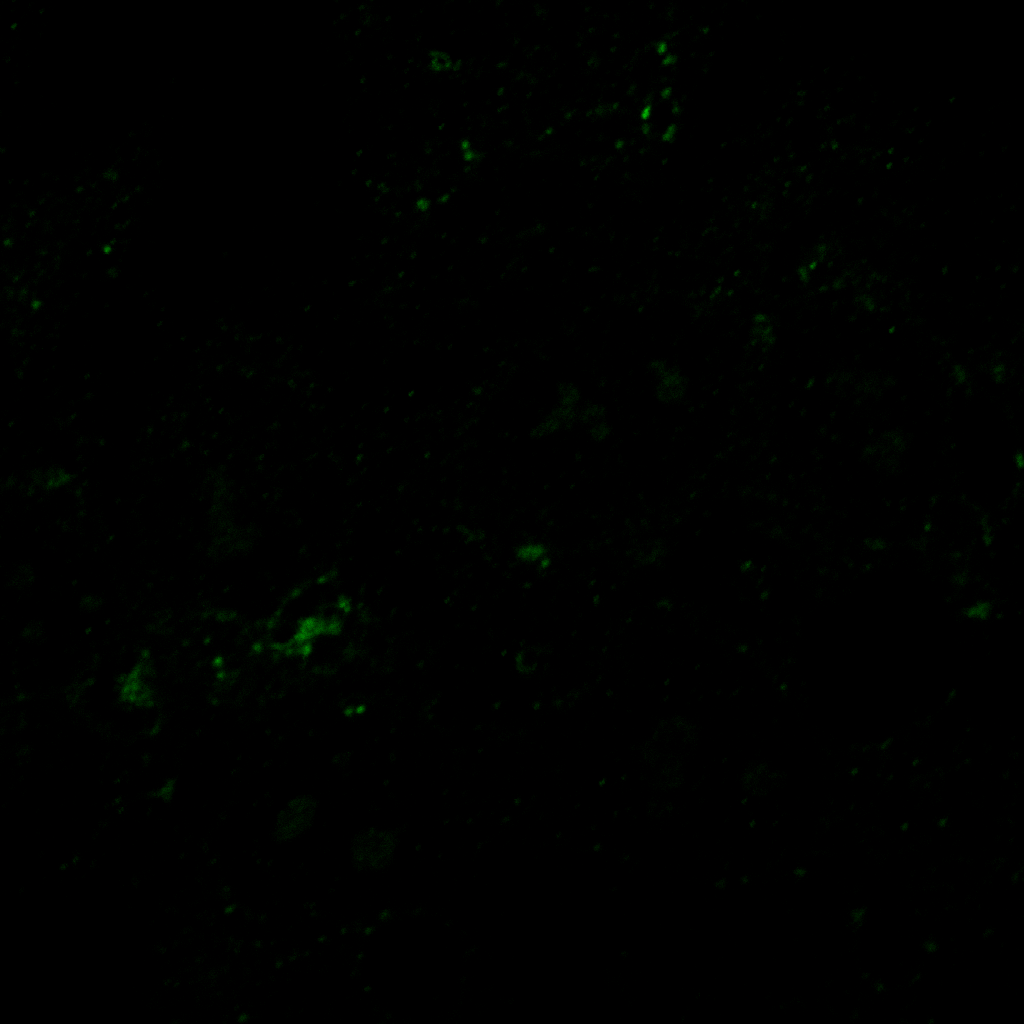

Supplement: Supplementary file 16 — Figure EV6 Source Data [file 44318_2024_353_MOESM16_ESM.zip › EVFigure 6/6G/PLCPRF5 siCtrl+RHEB-K169R/HP_PLC SINC+K169R 60X3-1_RGB_C2.tif]

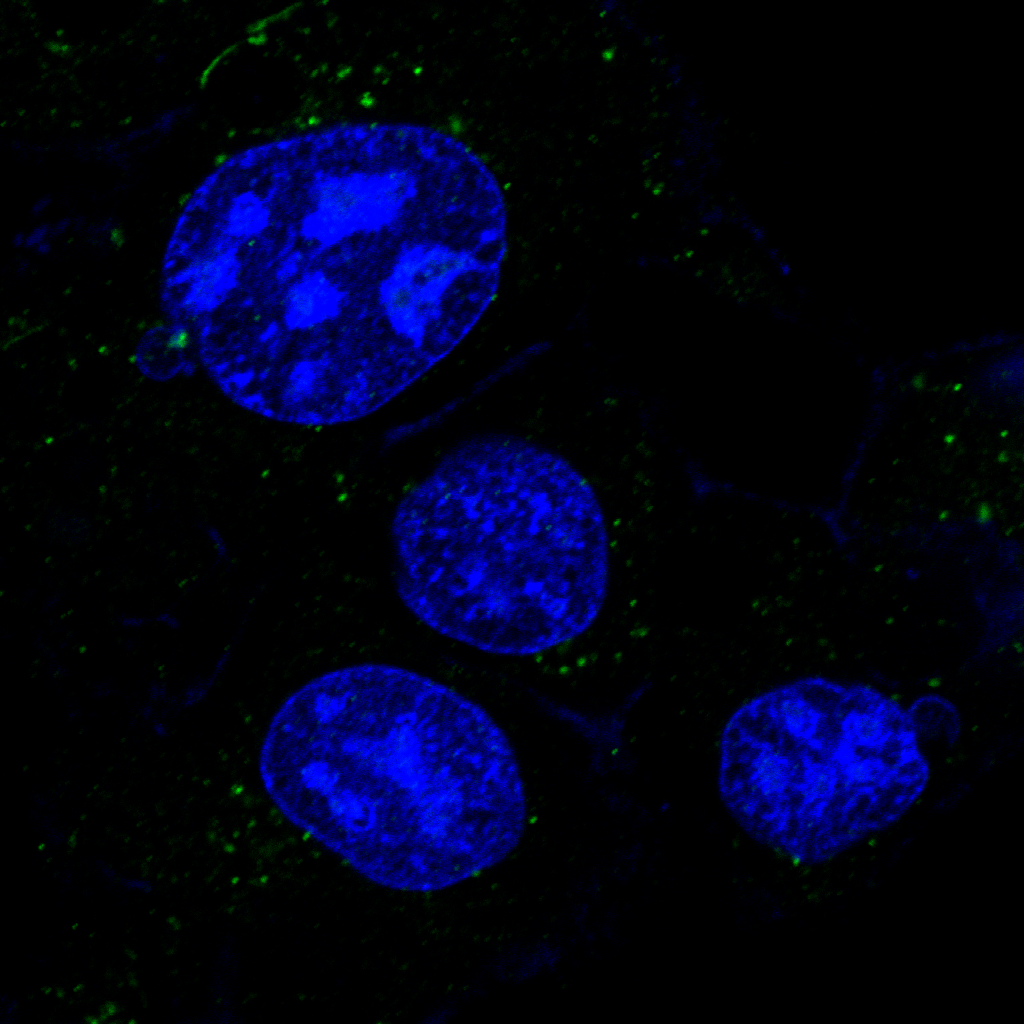

Supplement: Supplementary file 16 — Figure EV6 Source Data [file 44318_2024_353_MOESM16_ESM.zip › EVFigure 6/6G/PLCPRF5 siCtrl+Vector/HP_PLC SINC+V 60X3-2_RGB.tif]

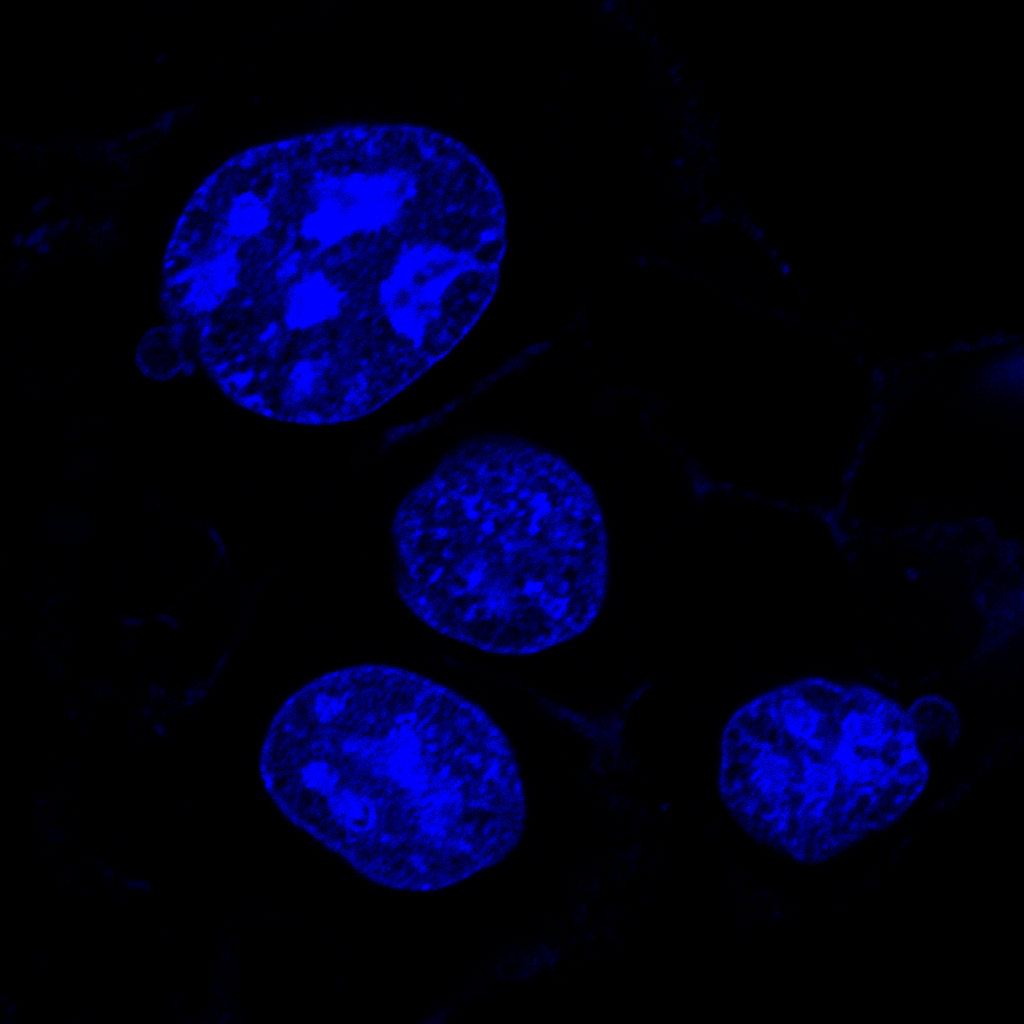

Supplement: Supplementary file 16 — Figure EV6 Source Data [file 44318_2024_353_MOESM16_ESM.zip › EVFigure 6/6G/PLCPRF5 siCtrl+Vector/HP_PLC SINC+V 60X3-2_RGB_C1.tif]

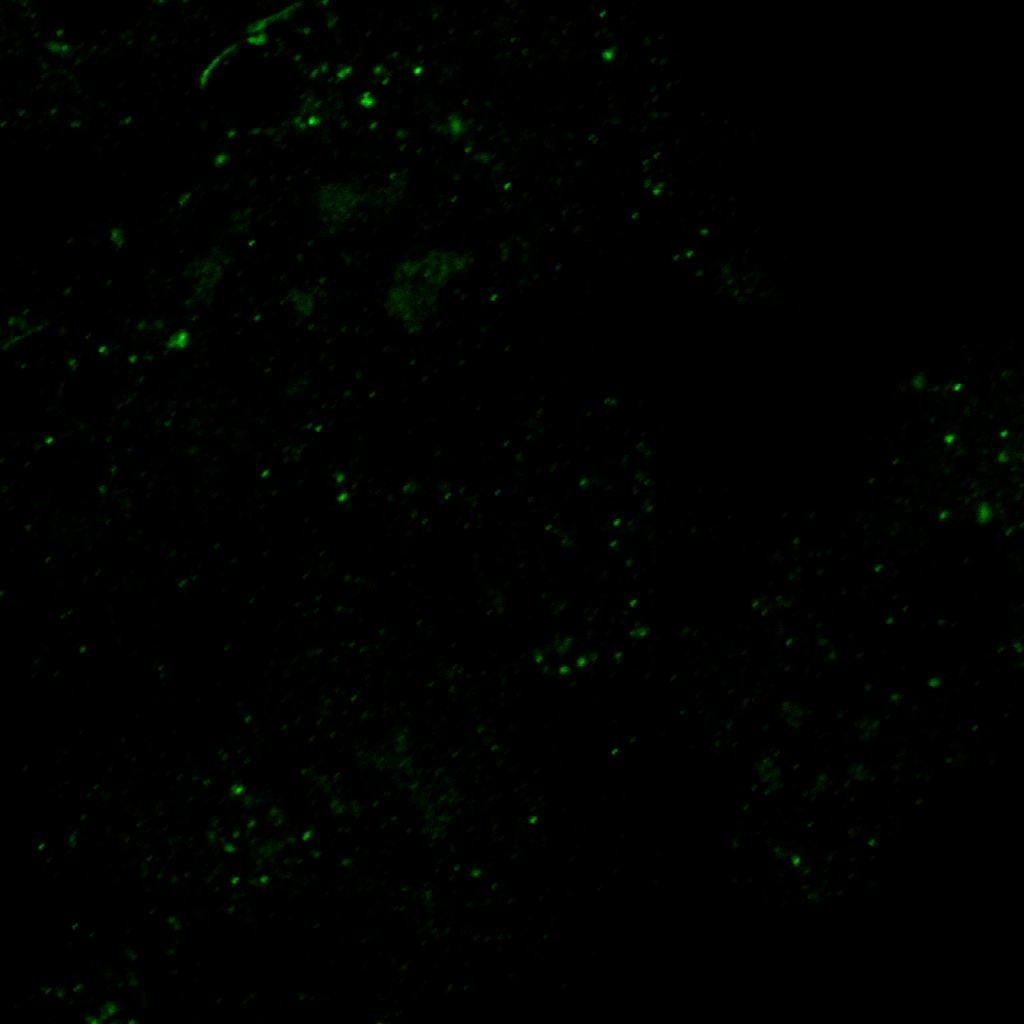

Supplement: Supplementary file 16 — Figure EV6 Source Data [file 44318_2024_353_MOESM16_ESM.zip › EVFigure 6/6G/PLCPRF5 siCtrl+Vector/HP_PLC SINC+V 60X3-2_RGB_C2.tif]

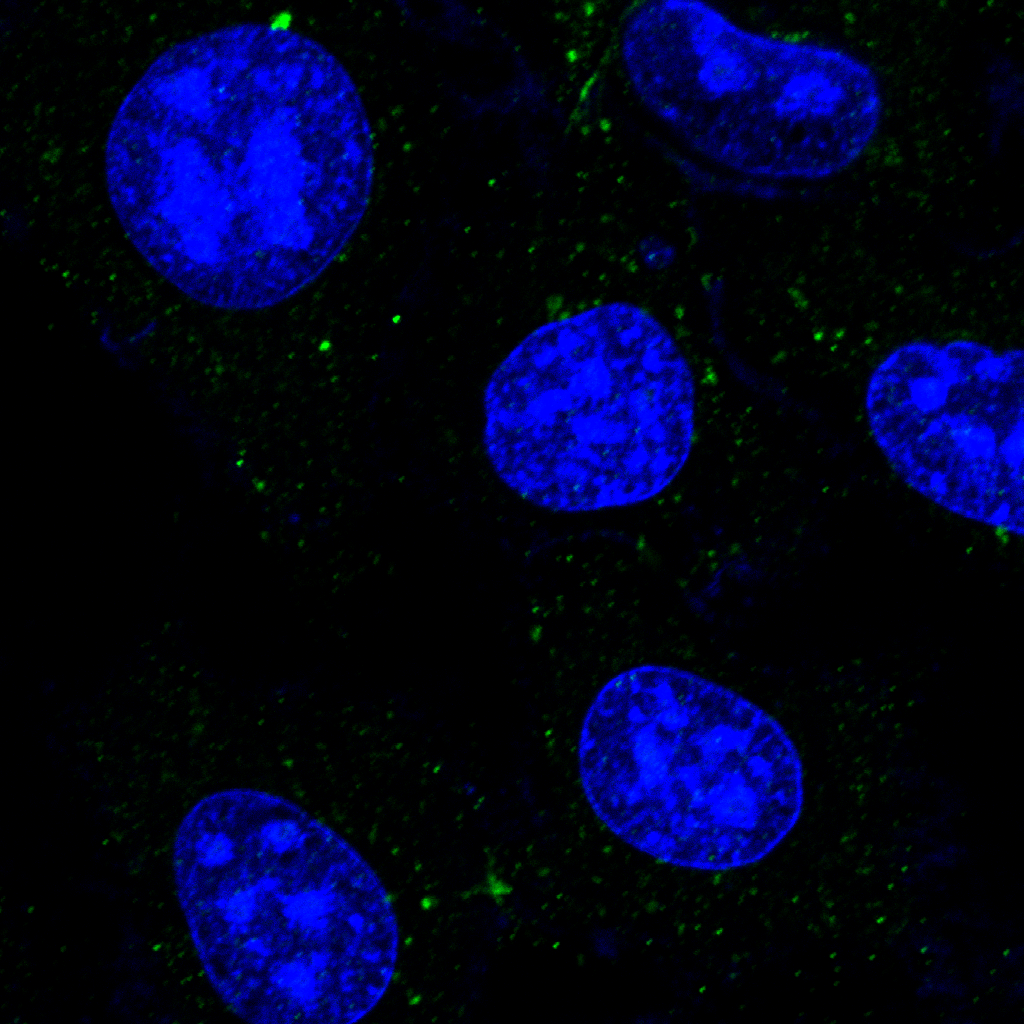

Supplement: Supplementary file 16 — Figure EV6 Source Data [file 44318_2024_353_MOESM16_ESM.zip › EVFigure 6/6G/PLCPRF5 siCtrl+WT-RHEB/HP_PLC SINC+WT 60X3-2_RGB.tif]

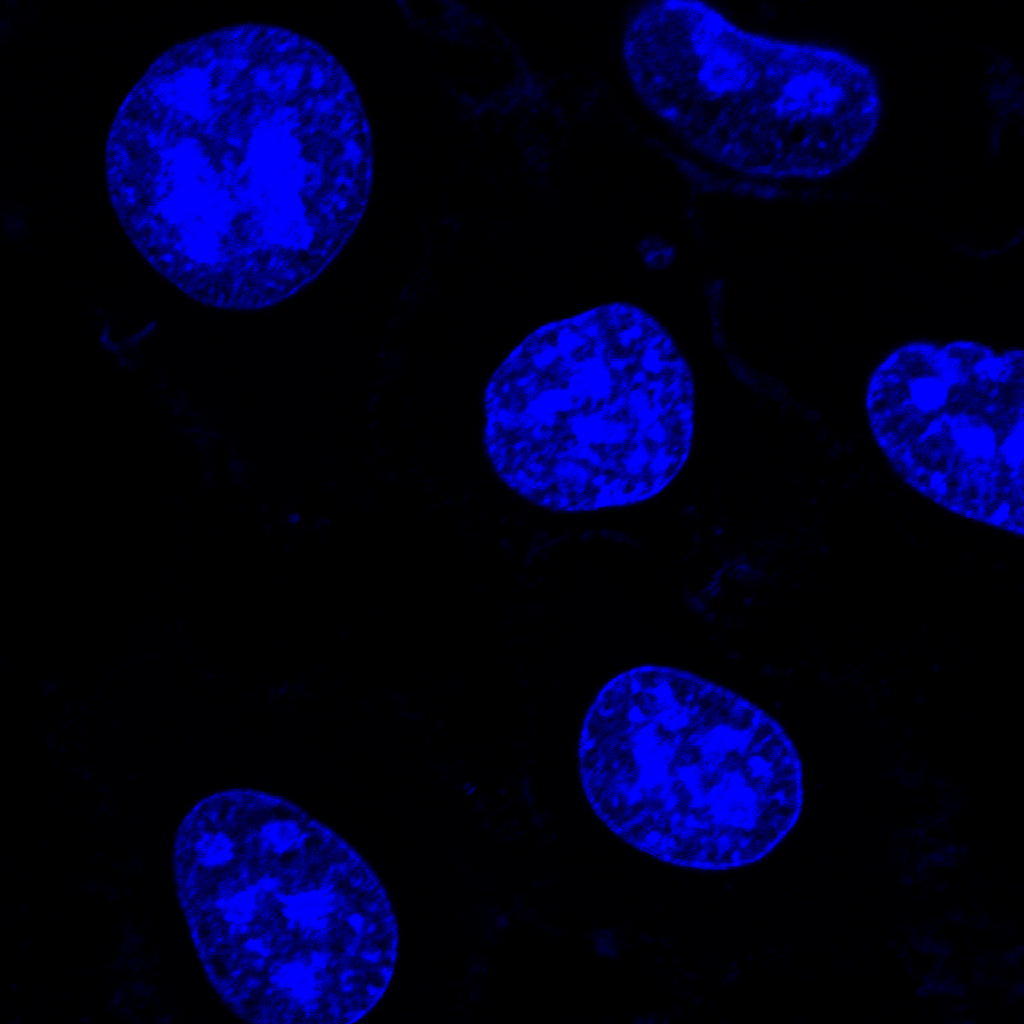

Supplement: Supplementary file 16 — Figure EV6 Source Data [file 44318_2024_353_MOESM16_ESM.zip › EVFigure 6/6G/PLCPRF5 siCtrl+WT-RHEB/HP_PLC SINC+WT 60X3-2_RGB_C1.tif]

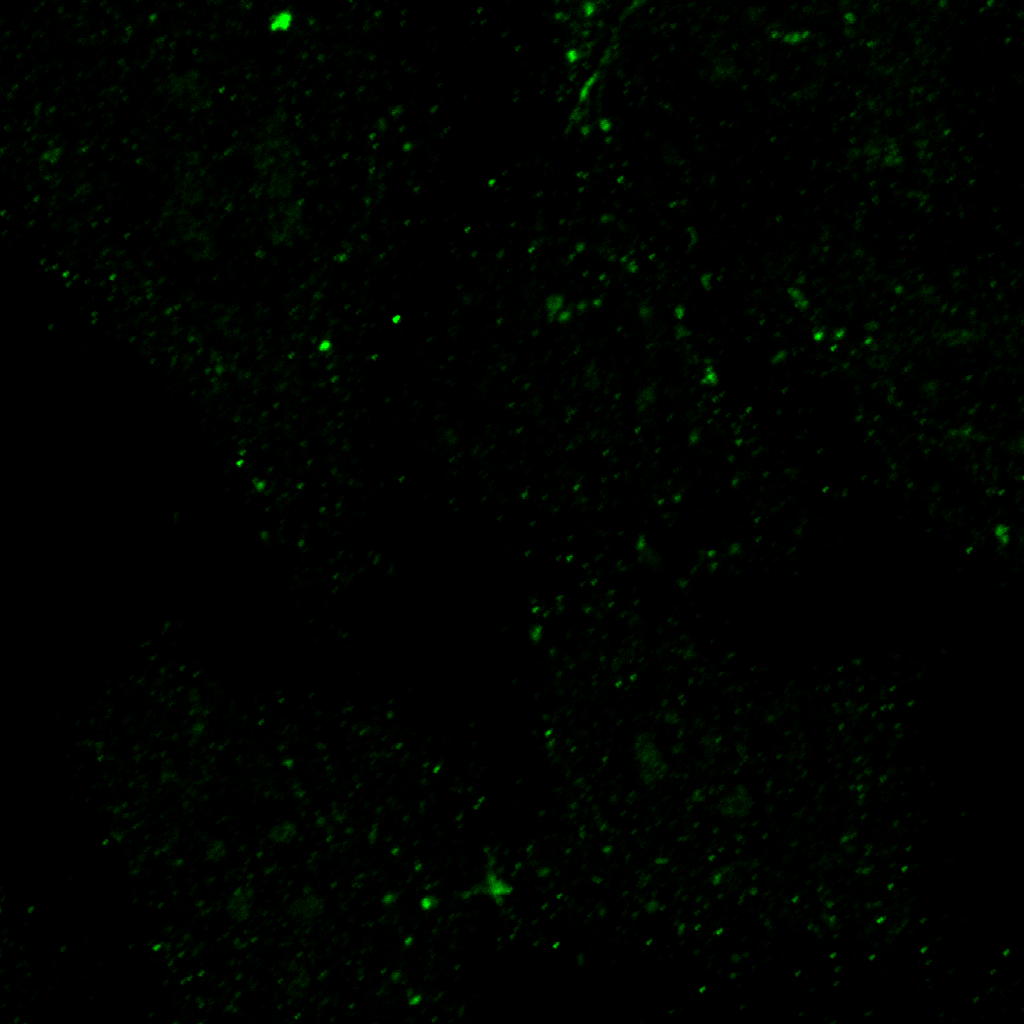

Supplement: Supplementary file 16 — Figure EV6 Source Data [file 44318_2024_353_MOESM16_ESM.zip › EVFigure 6/6G/PLCPRF5 siCtrl+WT-RHEB/HP_PLC SINC+WT 60X3-2_RGB_C2.tif]

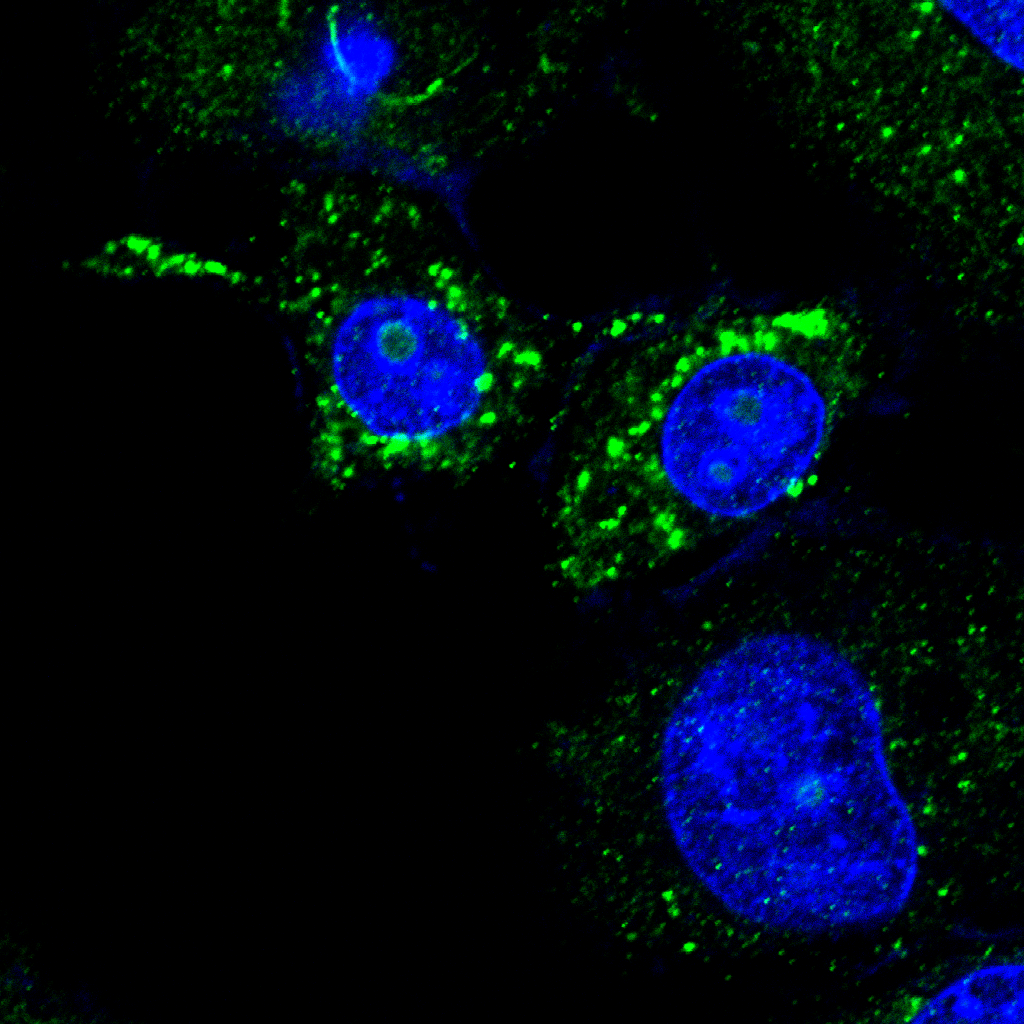

Supplement: Supplementary file 16 — Figure EV6 Source Data [file 44318_2024_353_MOESM16_ESM.zip › EVFigure 6/6G/PLCPRF5 siSAG+RHEBK169R/HP_PLC SISAG+RHEBK169R 60X3-2_RGB.tif]

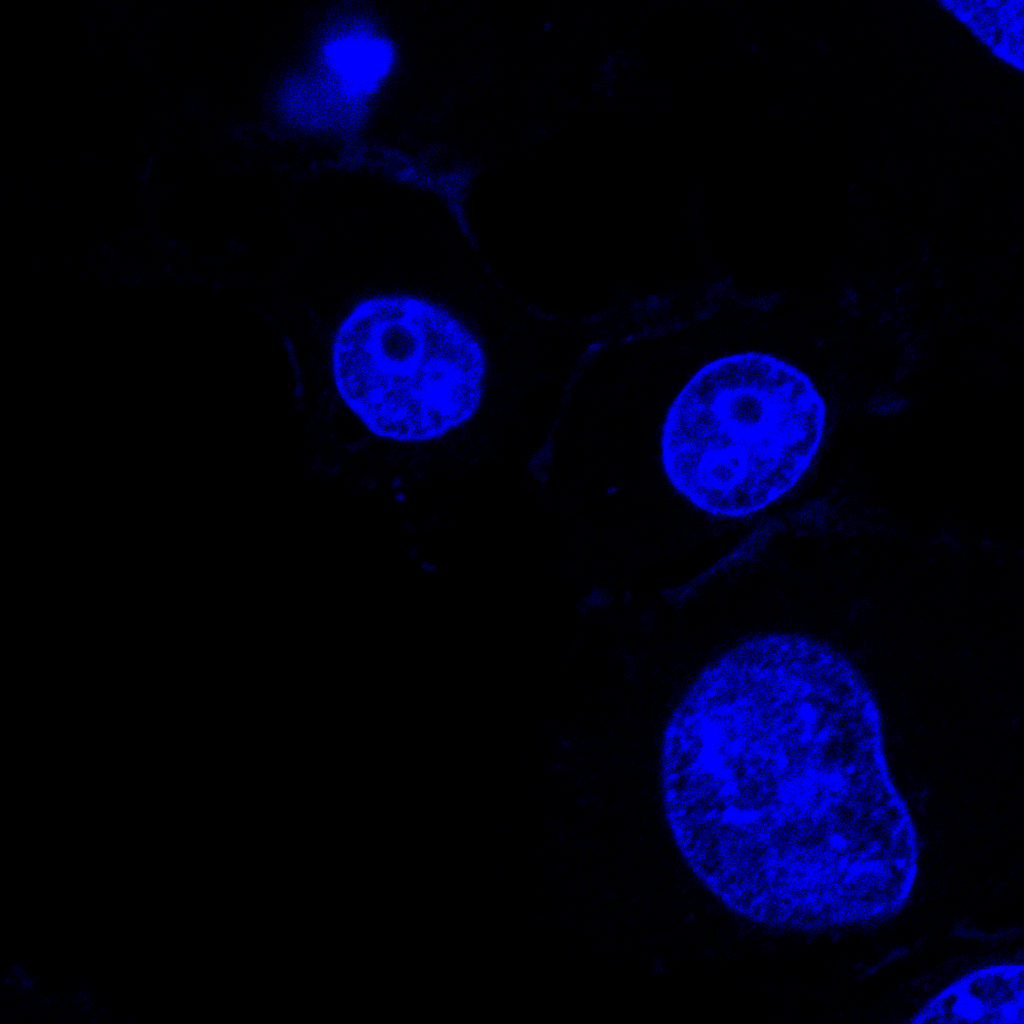

Supplement: Supplementary file 16 — Figure EV6 Source Data [file 44318_2024_353_MOESM16_ESM.zip › EVFigure 6/6G/PLCPRF5 siSAG+RHEBK169R/HP_PLC SISAG+RHEBK169R 60X3-2_RGB_C1.tif]

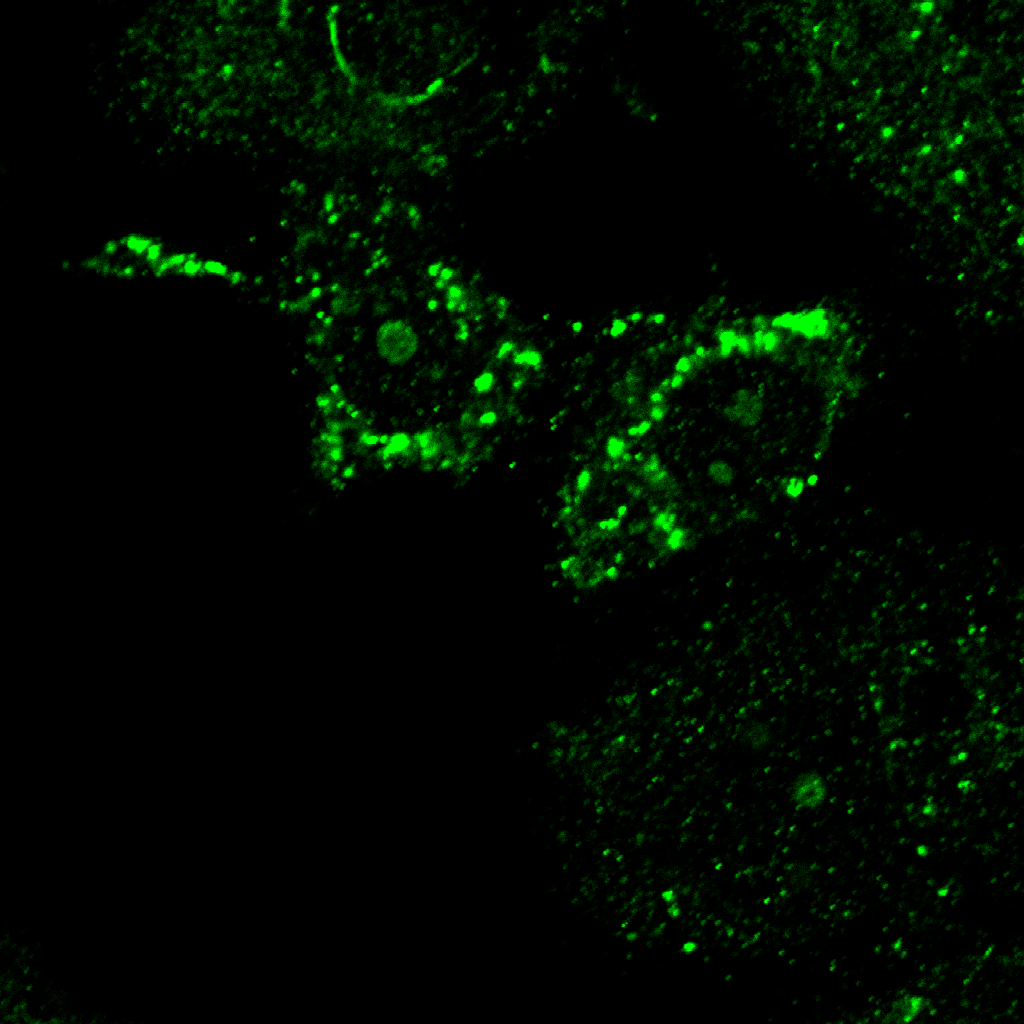

Supplement: Supplementary file 16 — Figure EV6 Source Data [file 44318_2024_353_MOESM16_ESM.zip › EVFigure 6/6G/PLCPRF5 siSAG+RHEBK169R/HP_PLC SISAG+RHEBK169R 60X3-2_RGB_C2.tif]

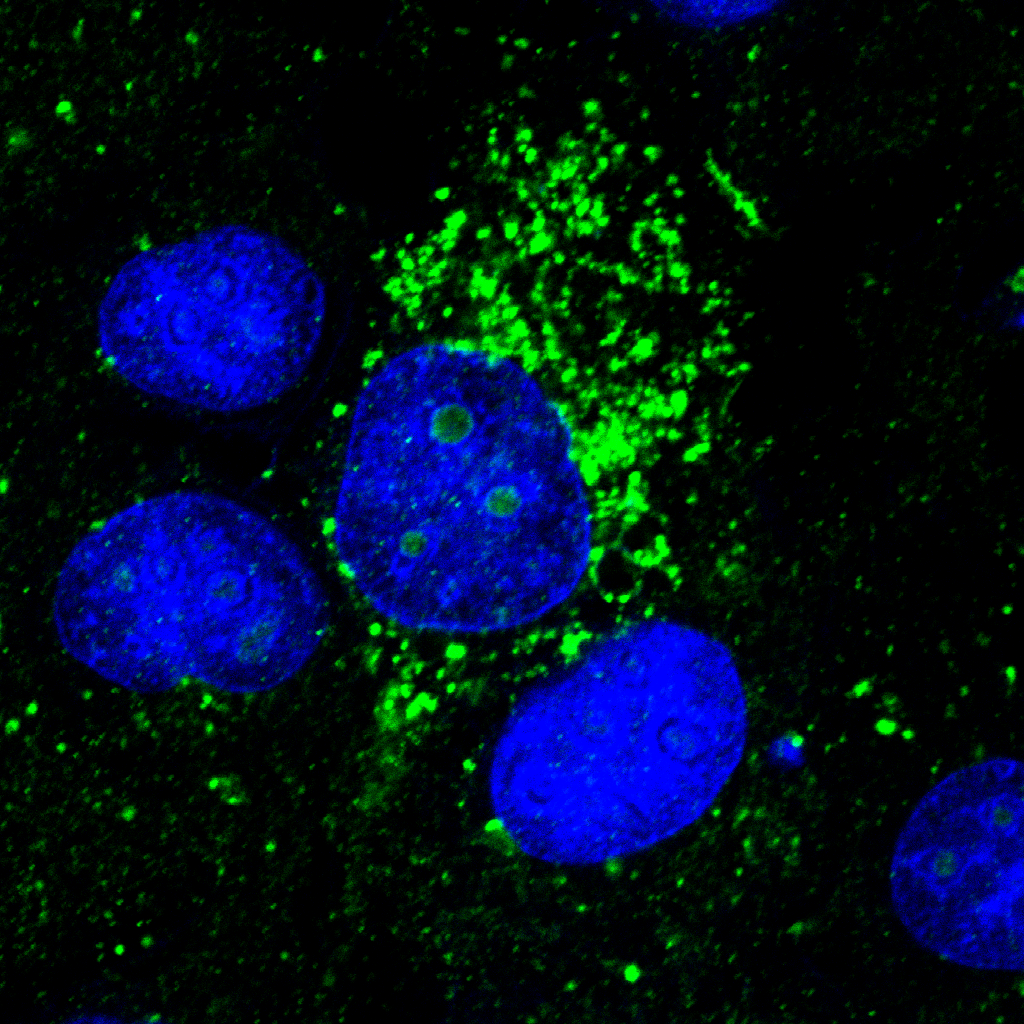

Supplement: Supplementary file 16 — Figure EV6 Source Data [file 44318_2024_353_MOESM16_ESM.zip › EVFigure 6/6G/PLCPRF5 siSAG+Vector/HP_PLC SISAG+V 60X3-5_RGB.tif]

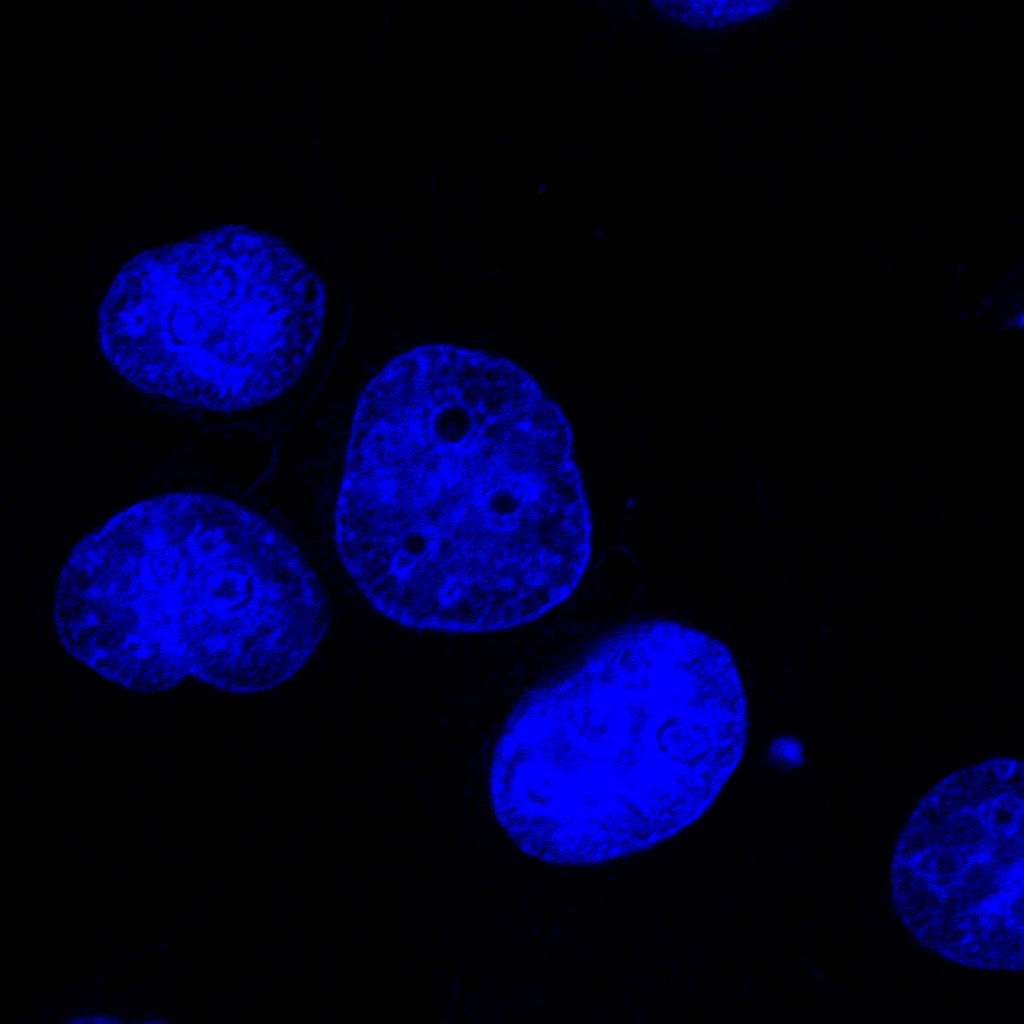

Supplement: Supplementary file 16 — Figure EV6 Source Data [file 44318_2024_353_MOESM16_ESM.zip › EVFigure 6/6G/PLCPRF5 siSAG+Vector/HP_PLC SISAG+V 60X3-5_RGB_C1.tif]

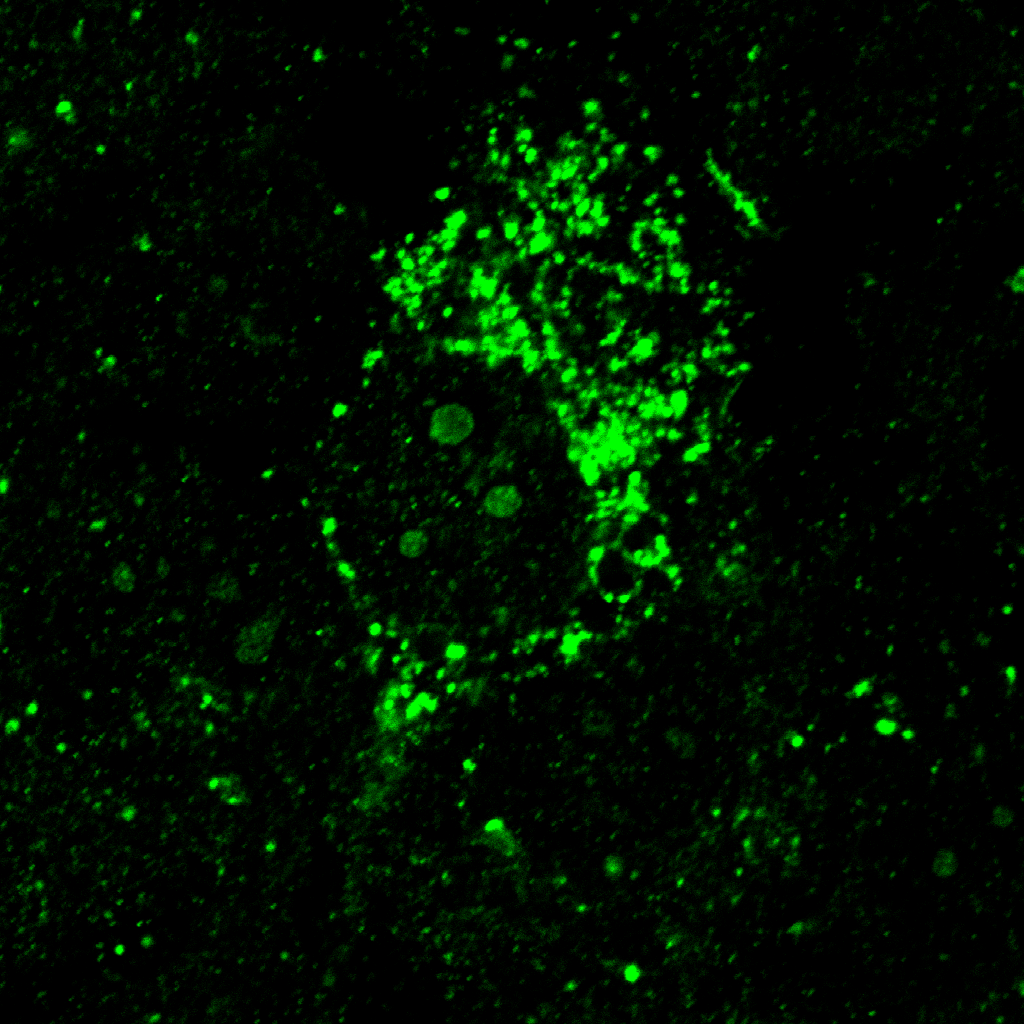

Supplement: Supplementary file 16 — Figure EV6 Source Data [file 44318_2024_353_MOESM16_ESM.zip › EVFigure 6/6G/PLCPRF5 siSAG+Vector/HP_PLC SISAG+V 60X3-5_RGB_C2.tif]

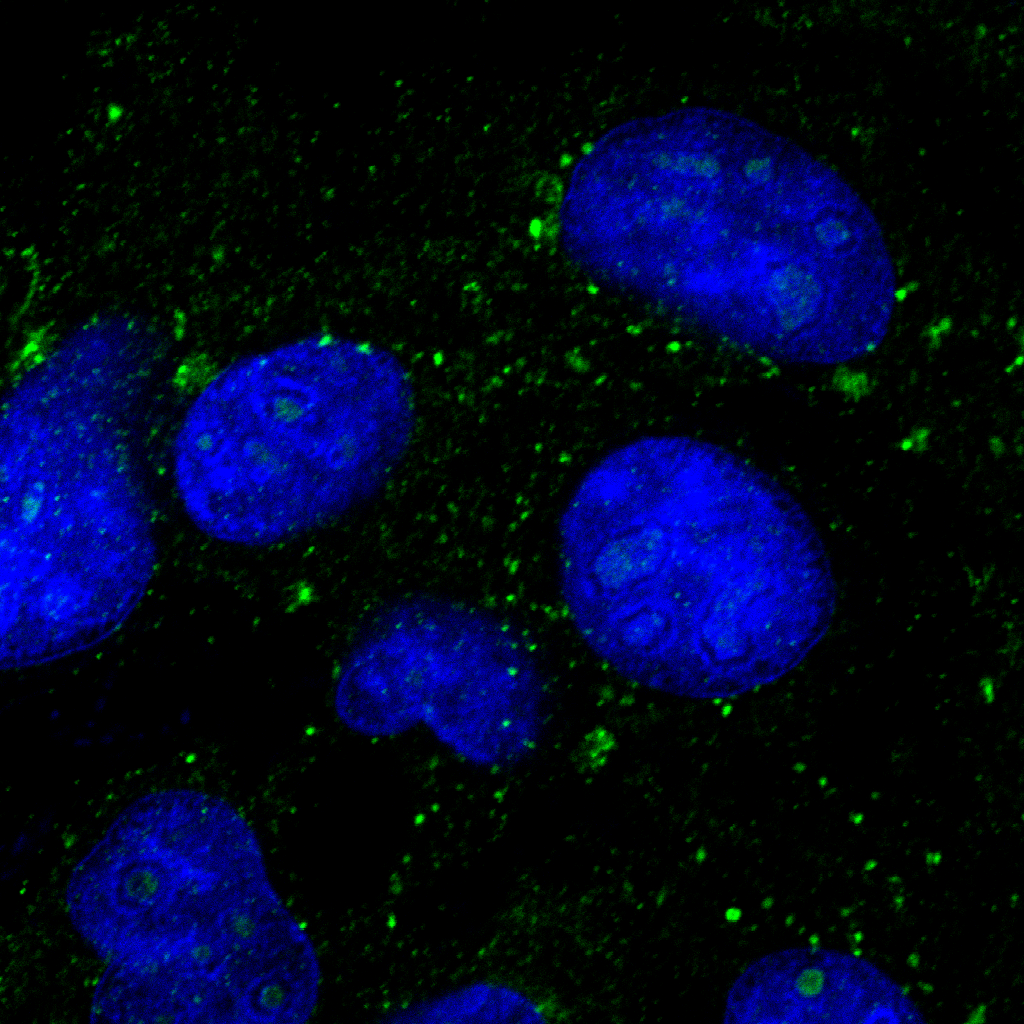

Supplement: Supplementary file 16 — Figure EV6 Source Data [file 44318_2024_353_MOESM16_ESM.zip › EVFigure 6/6G/PLCPRF5 siSAG+WTRHEB/HP_PLC SISAG+WTRHEB 60X3-1_RGB.tif]

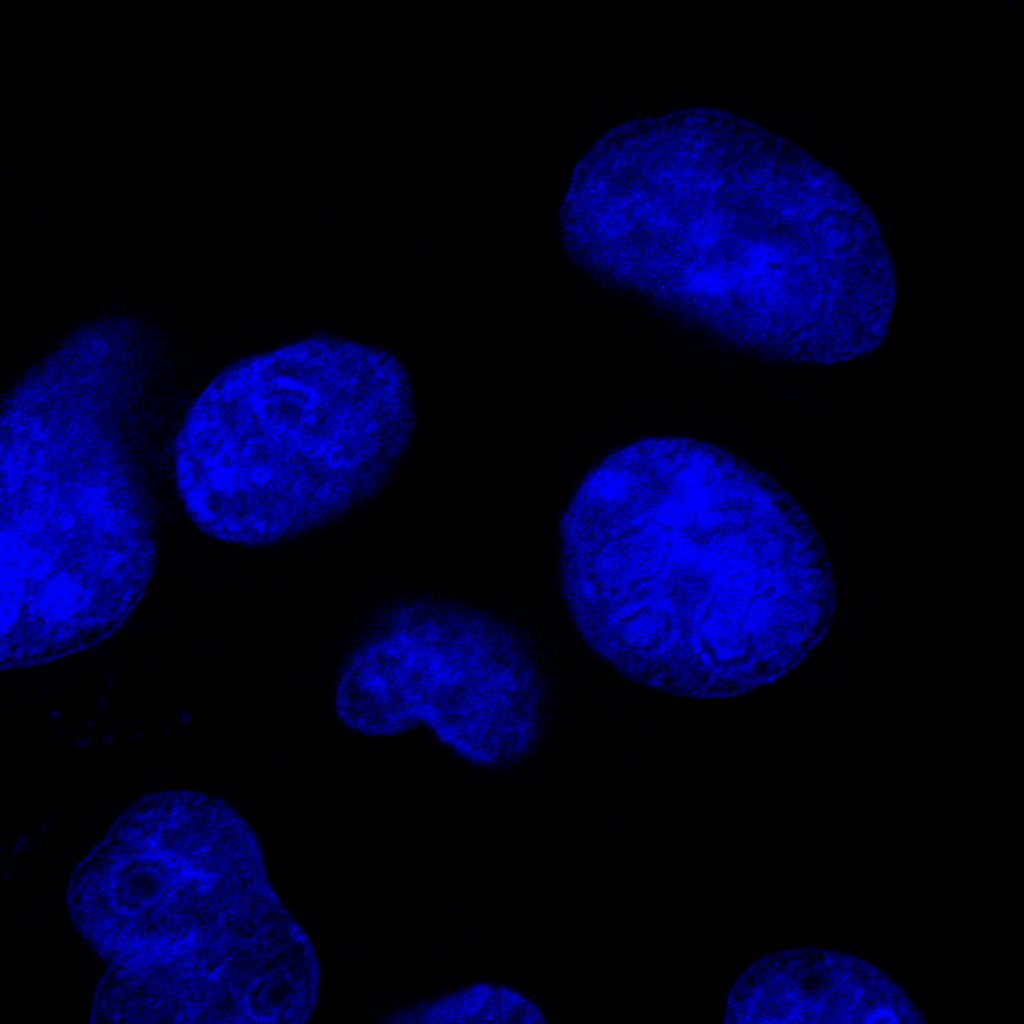

Supplement: Supplementary file 16 — Figure EV6 Source Data [file 44318_2024_353_MOESM16_ESM.zip › EVFigure 6/6G/PLCPRF5 siSAG+WTRHEB/HP_PLC SISAG+WTRHEB 60X3-1_RGB_C1.tif]

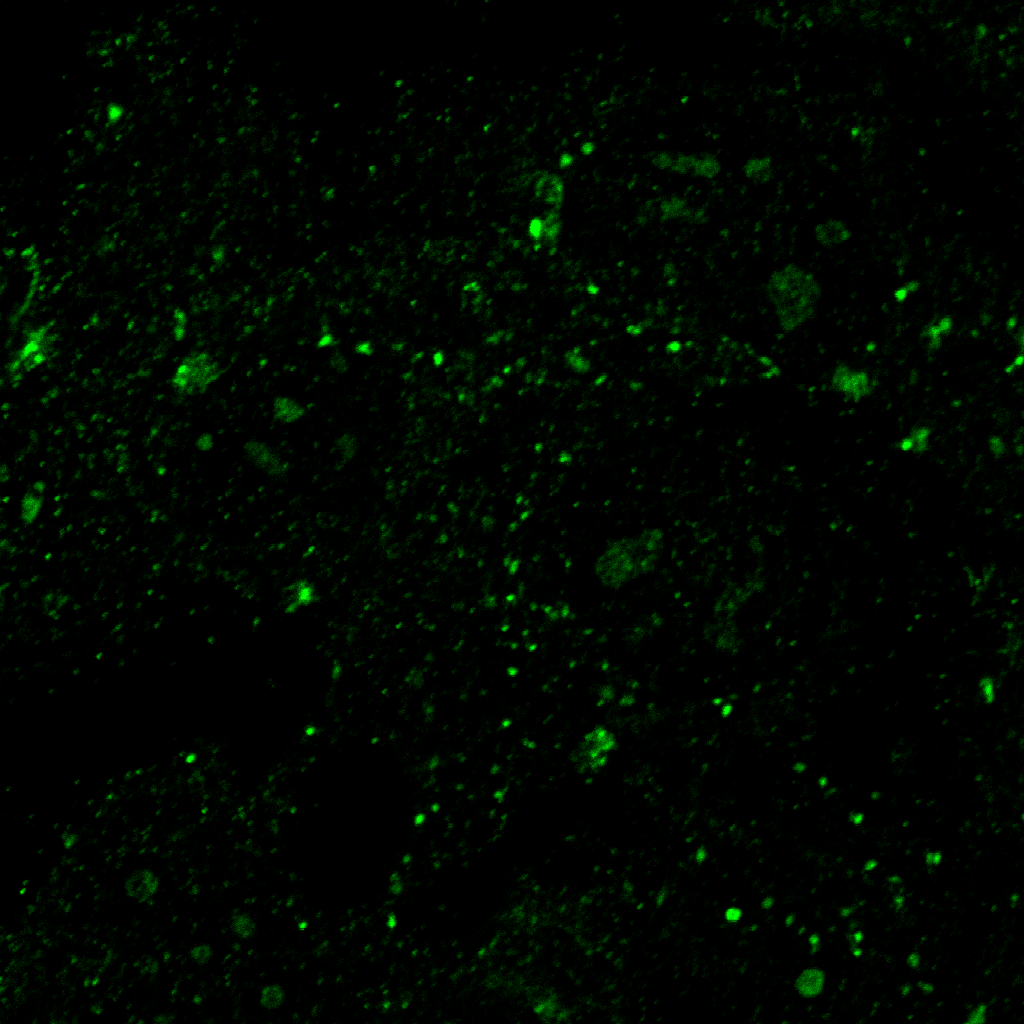

Supplement: Supplementary file 16 — Figure EV6 Source Data [file 44318_2024_353_MOESM16_ESM.zip › EVFigure 6/6G/PLCPRF5 siSAG+WTRHEB/HP_PLC SISAG+WTRHEB 60X3-1_RGB_C2.tif]
