# Supplementary figures and images for: RHEB neddylation by the UBE2F-SAG axis enhances mTORC1 activity and aggravates liver tumorigenesis (part 3 of 3)
Source: EMBO J. 2025 Jan 6;44(4):1185–219. doi: 10.1038/s44318-024-00353-5 (PMC11832924; doi:10.1038/s44318-024-00353-5)

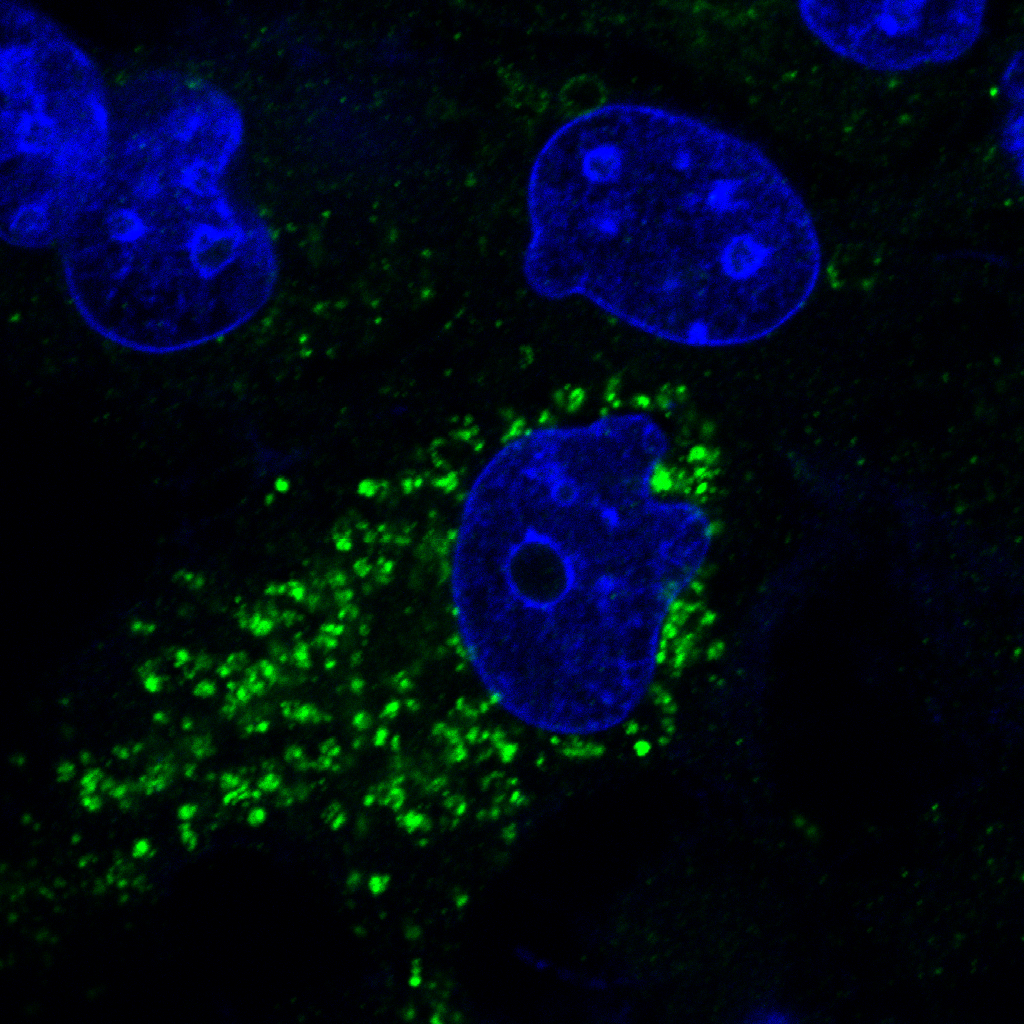

Supplement: Supplementary file 16 — Figure EV6 Source Data [file 44318_2024_353_MOESM16_ESM.zip › EVFigure 6/6G/PLCPRF5 siUBE2F+RHEB-K169R/HP_PLC SI2F+RHEBK169R 60X-3-1_RGB.tif]

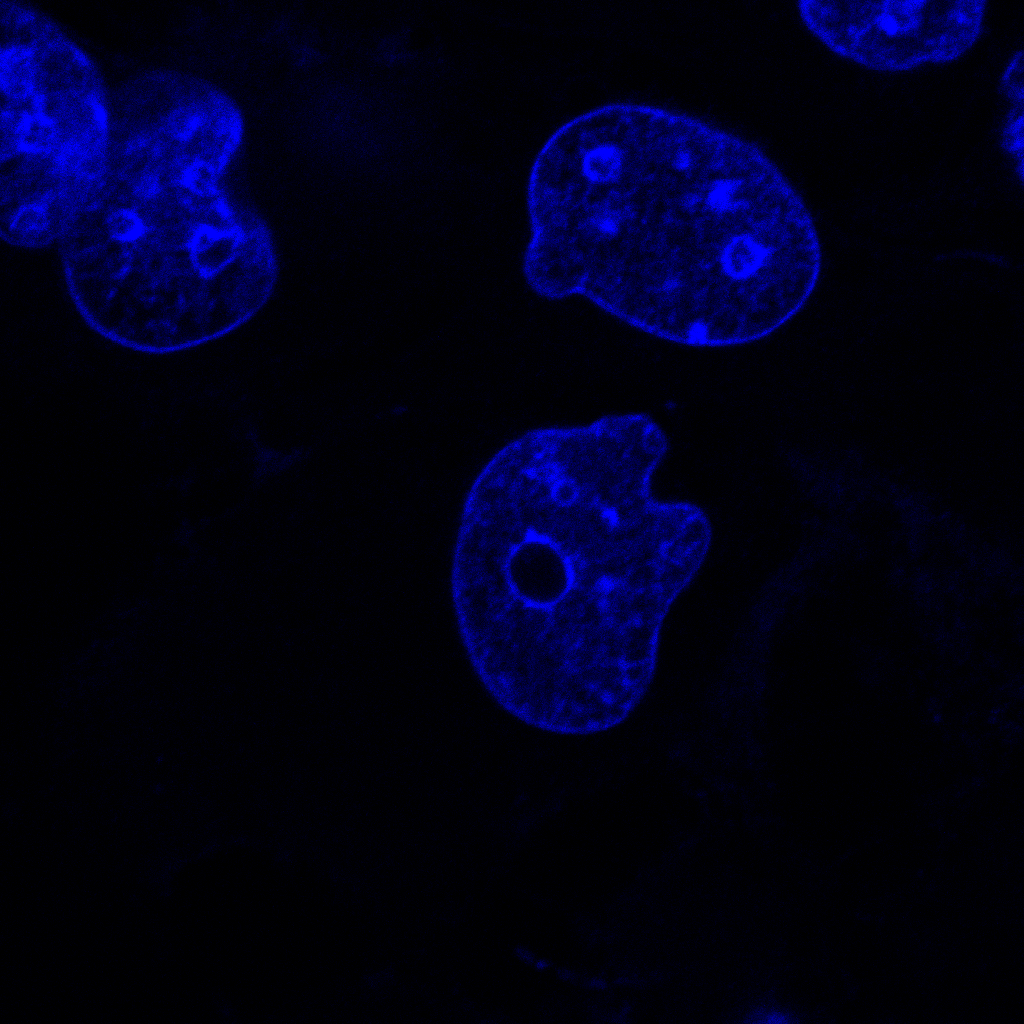

Supplement: Supplementary file 16 — Figure EV6 Source Data [file 44318_2024_353_MOESM16_ESM.zip › EVFigure 6/6G/PLCPRF5 siUBE2F+RHEB-K169R/HP_PLC SI2F+RHEBK169R 60X-3-1_RGB_C1.tif]

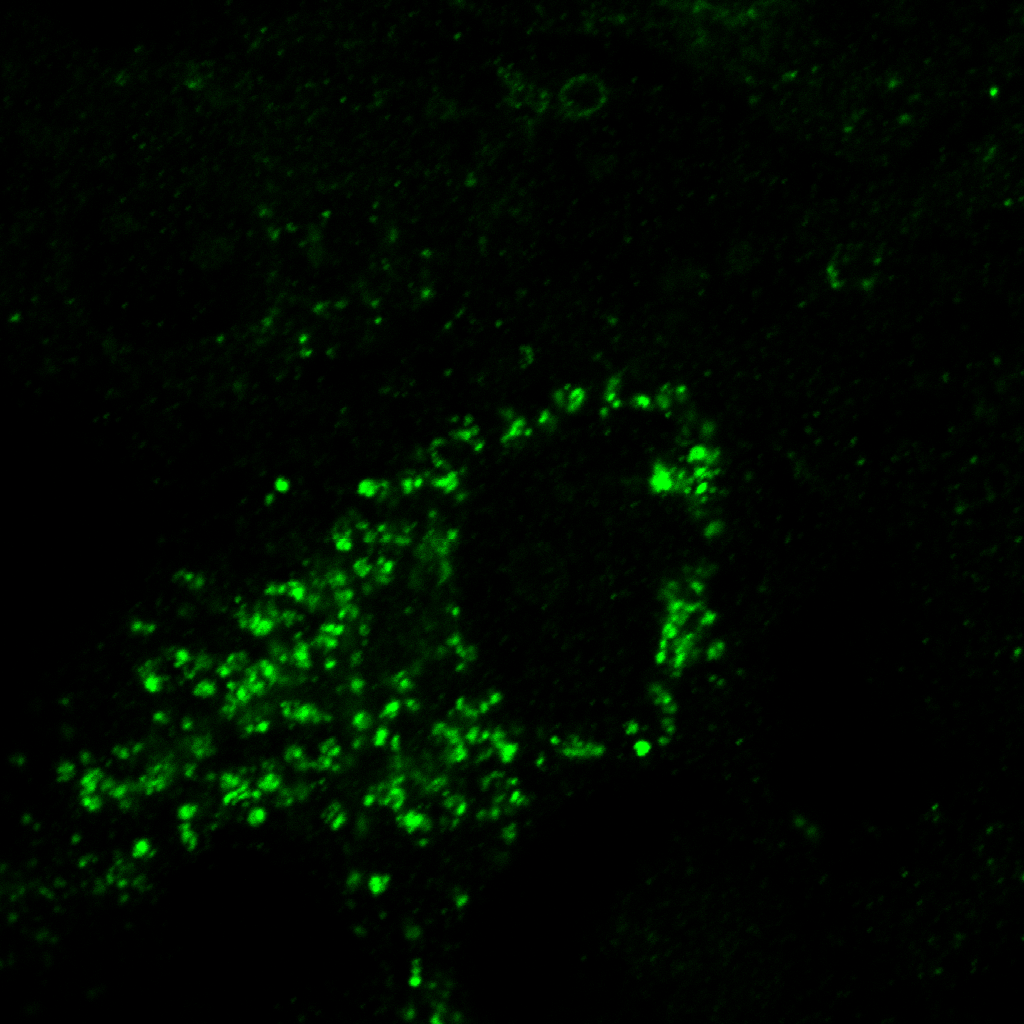

Supplement: Supplementary file 16 — Figure EV6 Source Data [file 44318_2024_353_MOESM16_ESM.zip › EVFigure 6/6G/PLCPRF5 siUBE2F+RHEB-K169R/HP_PLC SI2F+RHEBK169R 60X-3-1_RGB_C2.tif]

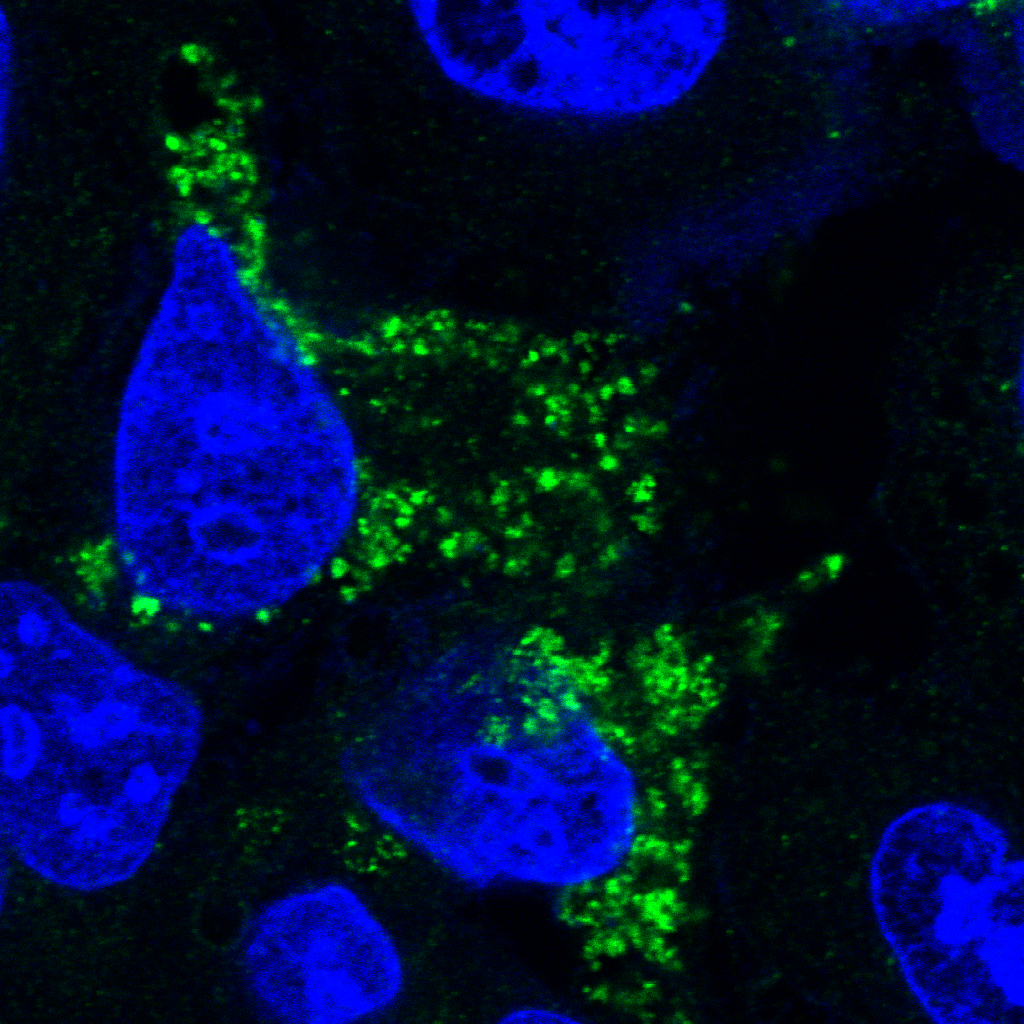

Supplement: Supplementary file 16 — Figure EV6 Source Data [file 44318_2024_353_MOESM16_ESM.zip › EVFigure 6/6G/PLCPRF5 siUBE2F+Vector/HP_PLC SI2F+V 60X3-3_RGB.tif]

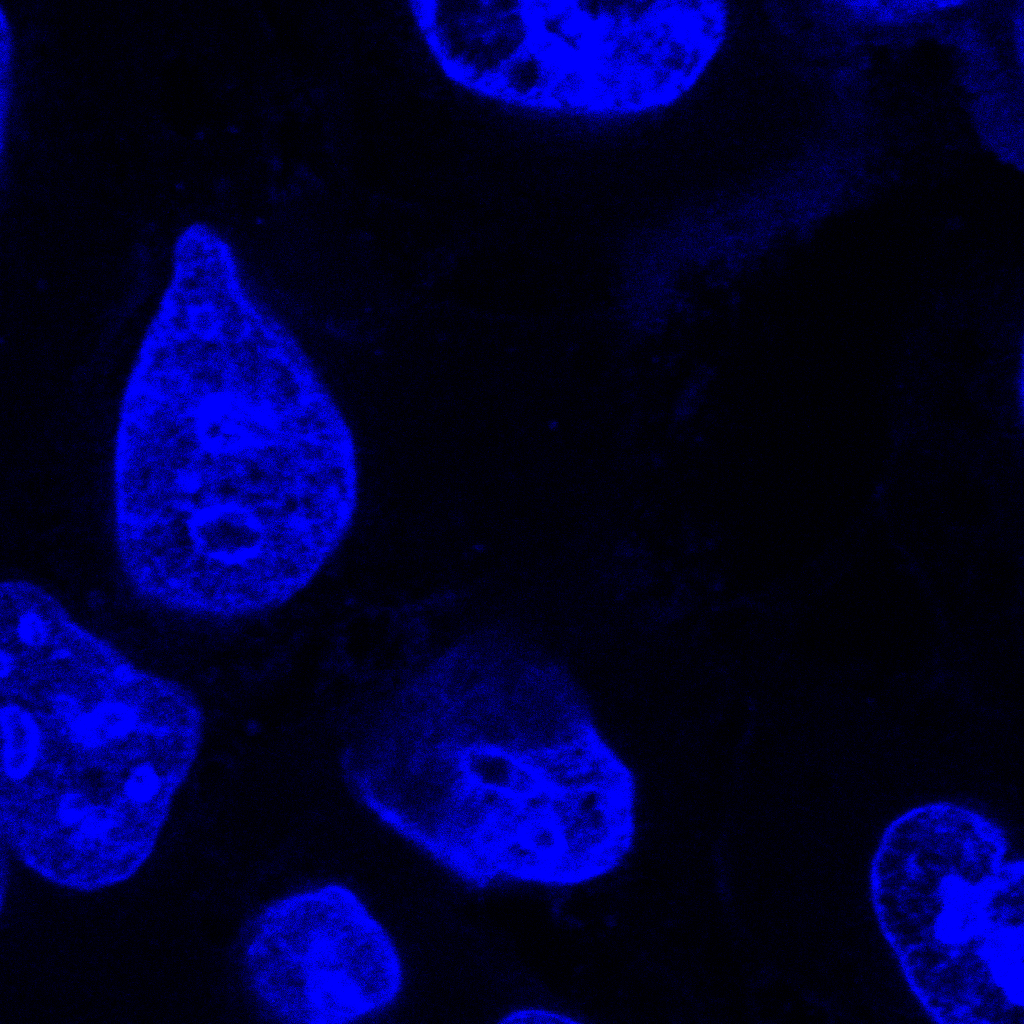

Supplement: Supplementary file 16 — Figure EV6 Source Data [file 44318_2024_353_MOESM16_ESM.zip › EVFigure 6/6G/PLCPRF5 siUBE2F+Vector/HP_PLC SI2F+V 60X3-3_RGB_C1.tif]

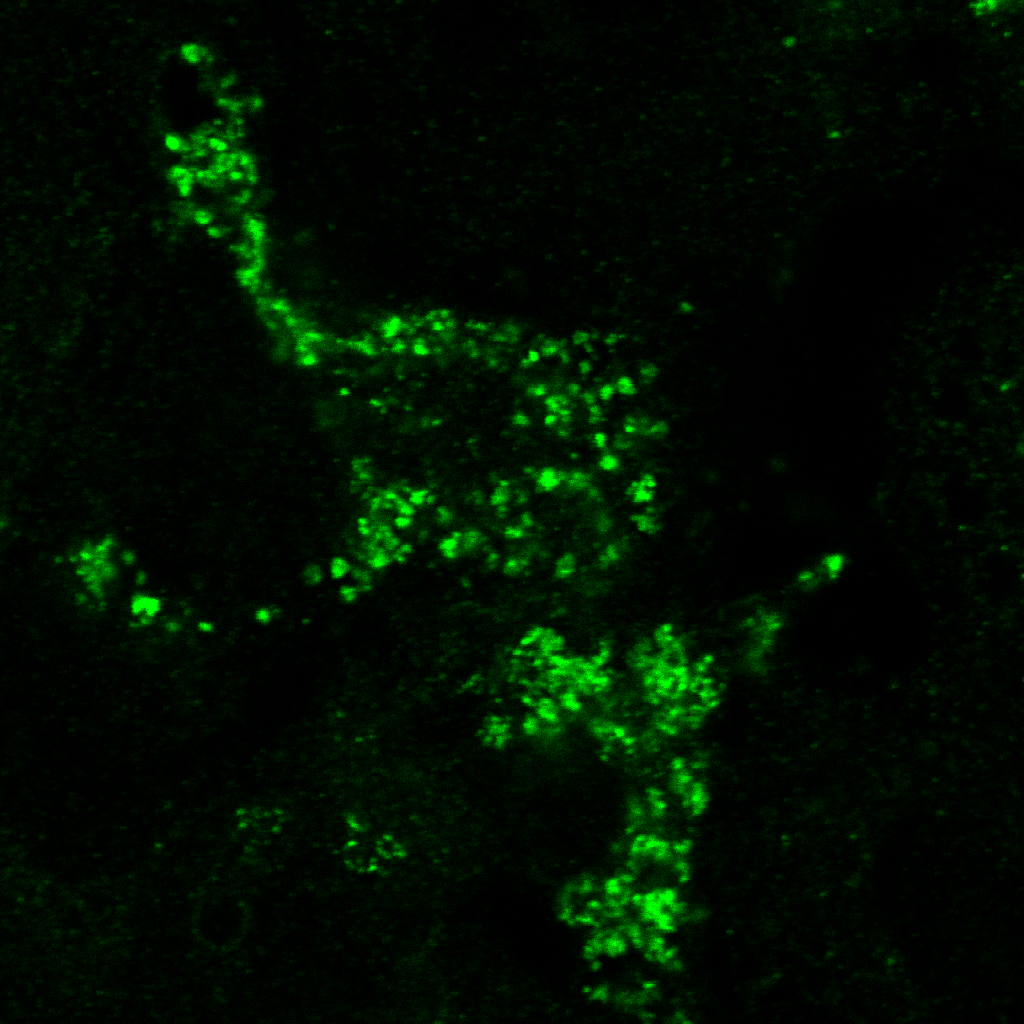

Supplement: Supplementary file 16 — Figure EV6 Source Data [file 44318_2024_353_MOESM16_ESM.zip › EVFigure 6/6G/PLCPRF5 siUBE2F+Vector/HP_PLC SI2F+V 60X3-3_RGB_C2.tif]

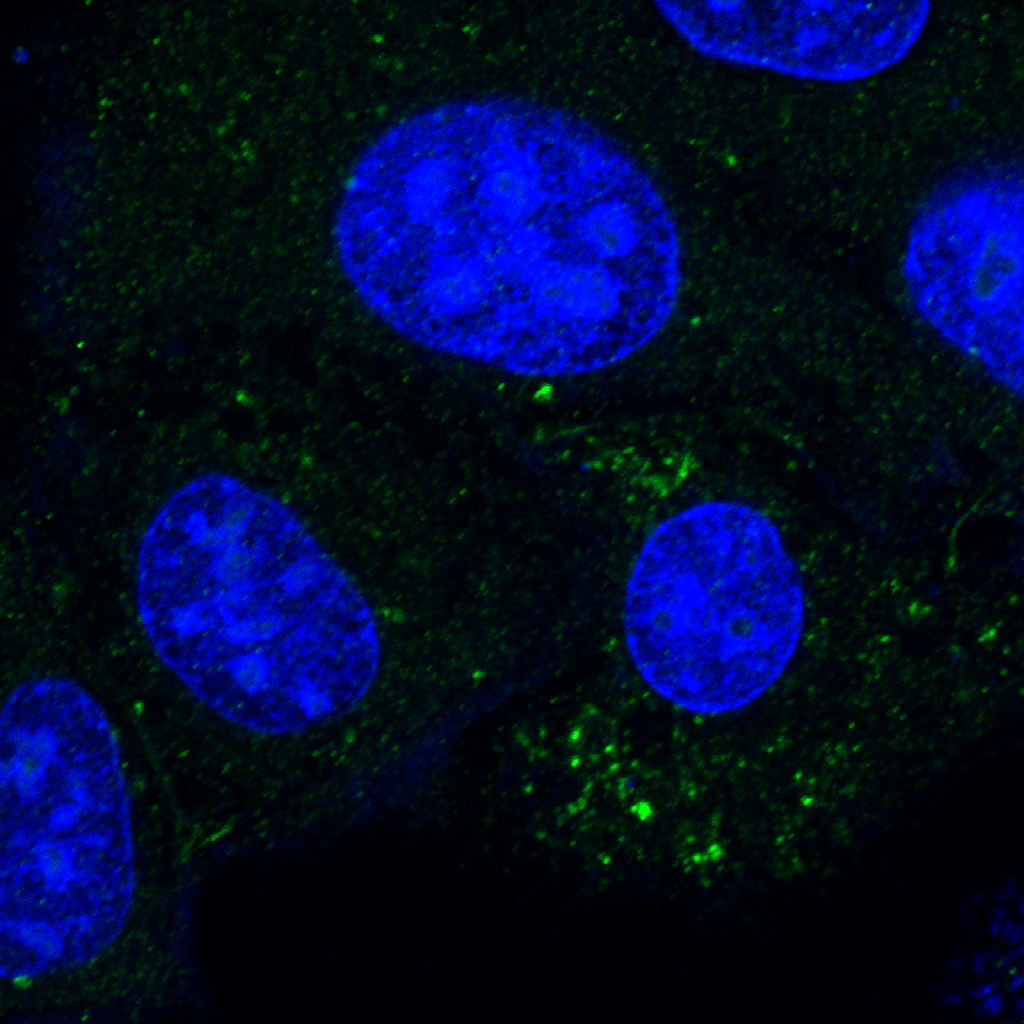

Supplement: Supplementary file 16 — Figure EV6 Source Data [file 44318_2024_353_MOESM16_ESM.zip › EVFigure 6/6G/PLCPRF5 siUBE2F+WTRHEB/HP_PLC SI2F+WTRHEB 60X-3-1_RGB.tif]

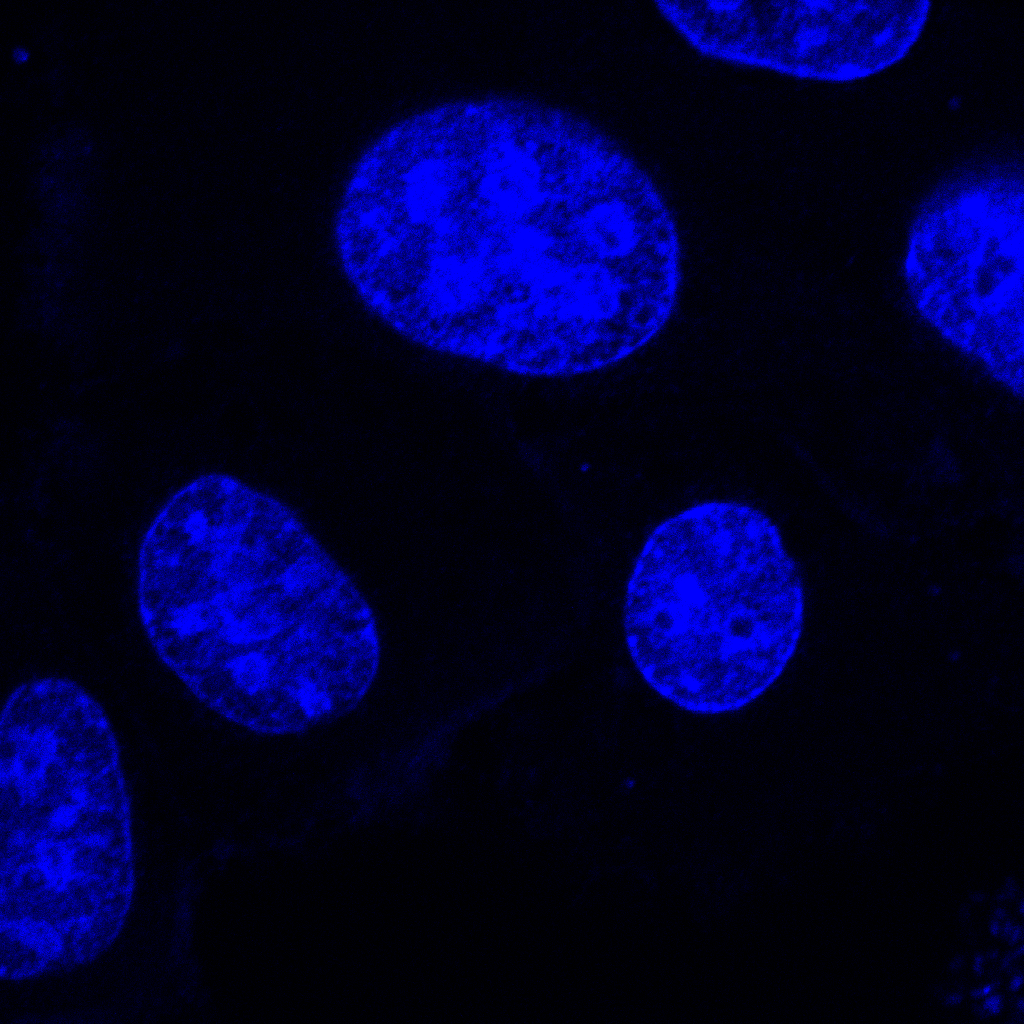

Supplement: Supplementary file 16 — Figure EV6 Source Data [file 44318_2024_353_MOESM16_ESM.zip › EVFigure 6/6G/PLCPRF5 siUBE2F+WTRHEB/HP_PLC SI2F+WTRHEB 60X-3-1_RGB_C1.tif]

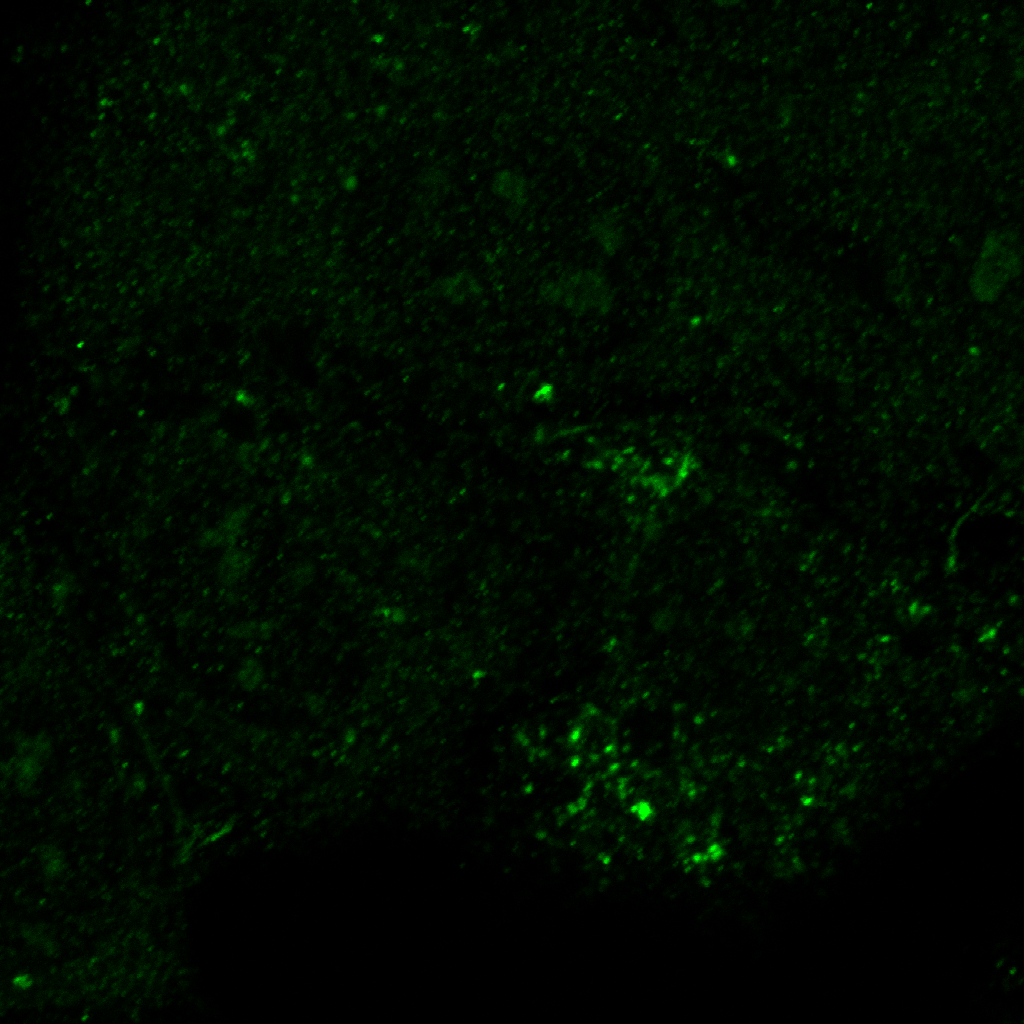

Supplement: Supplementary file 16 — Figure EV6 Source Data [file 44318_2024_353_MOESM16_ESM.zip › EVFigure 6/6G/PLCPRF5 siUBE2F+WTRHEB/HP_PLC SI2F+WTRHEB 60X-3-1_RGB_C2.tif]

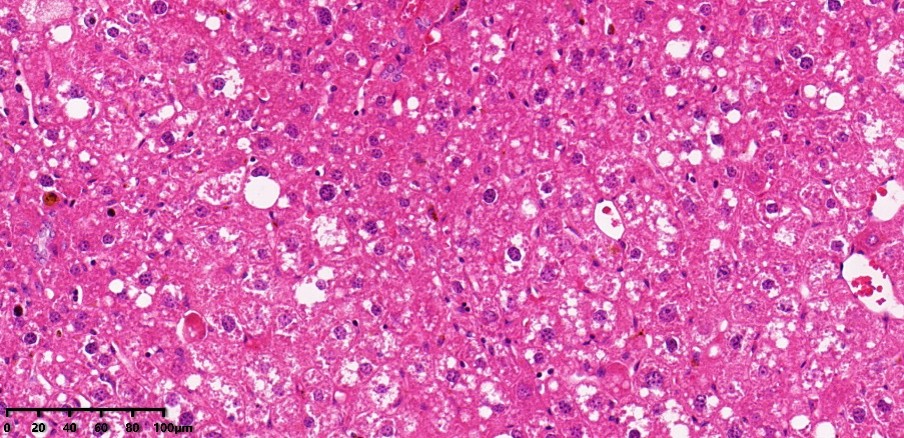

Supplement: Supplementary file 17 — Figure EV7 Source Data [file 44318_2024_353_MOESM17_ESM.zip › EVFigure 7/7F/KO HE insert.jpg]

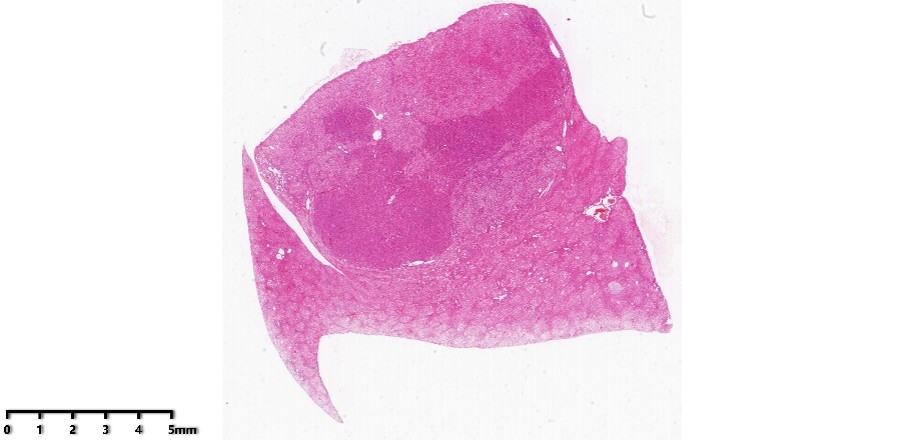

Supplement: Supplementary file 17 — Figure EV7 Source Data [file 44318_2024_353_MOESM17_ESM.zip › EVFigure 7/7F/KO HE.jpg]

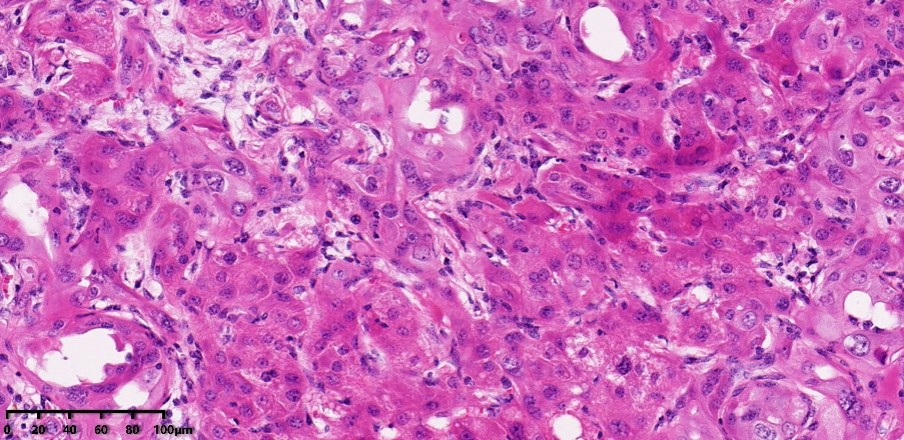

Supplement: Supplementary file 17 — Figure EV7 Source Data [file 44318_2024_353_MOESM17_ESM.zip › EVFigure 7/7F/WT HE insert.jpg]

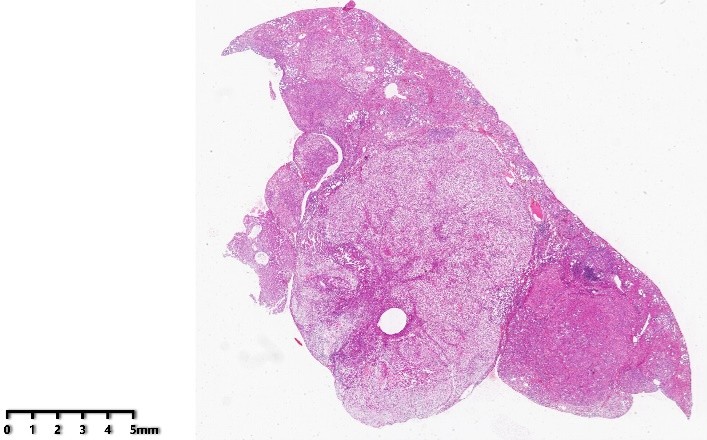

Supplement: Supplementary file 17 — Figure EV7 Source Data [file 44318_2024_353_MOESM17_ESM.zip › EVFigure 7/7F/WT HE.jpg]

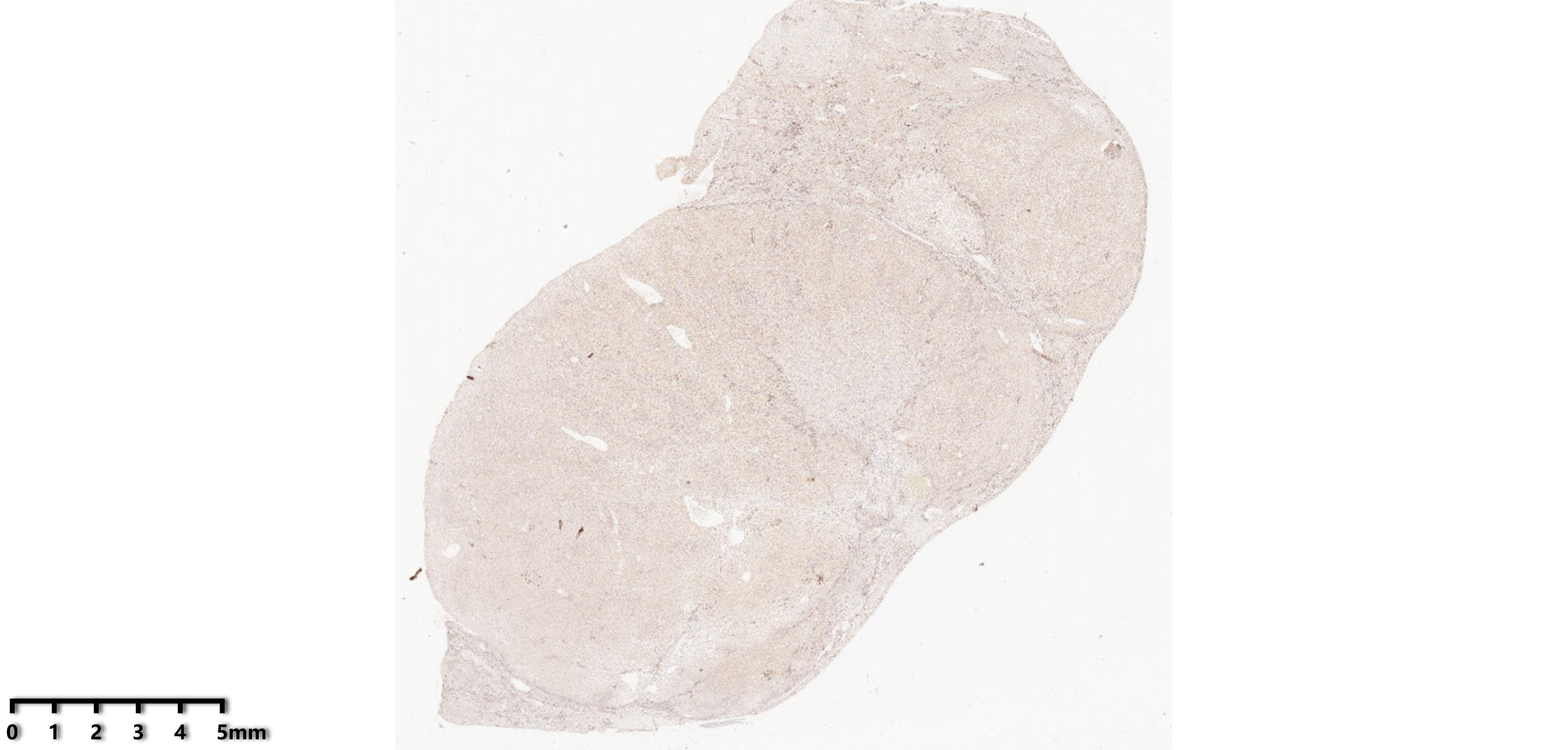

Supplement: Supplementary file 17 — Figure EV7 Source Data [file 44318_2024_353_MOESM17_ESM.zip › EVFigure 7/7I/18712CK192023-04-09_19_25_05_0.31x_1.jpg]

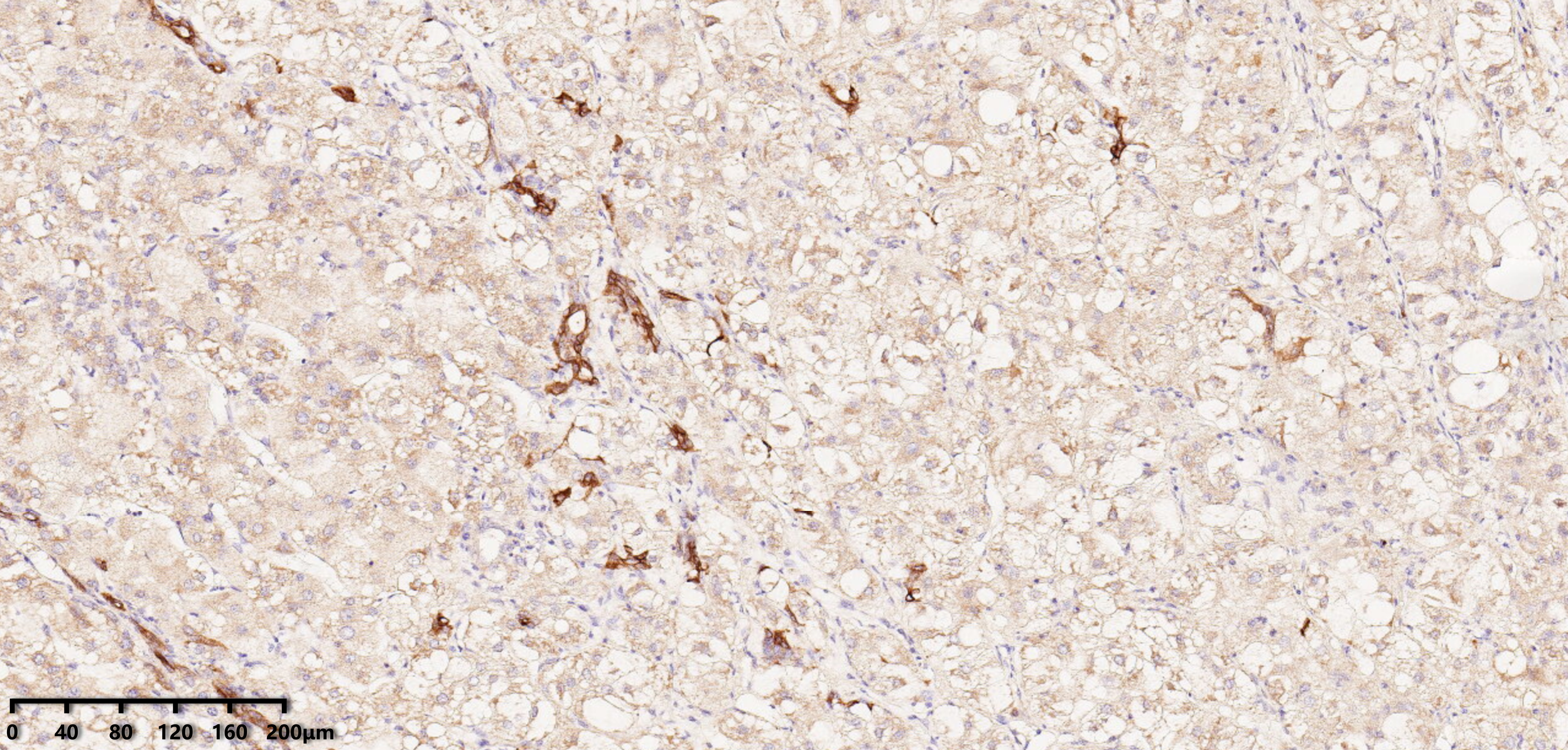

Supplement: Supplementary file 17 — Figure EV7 Source Data [file 44318_2024_353_MOESM17_ESM.zip › EVFigure 7/7I/18712CK192023-04-09_19_25_05_10x_2.jpg]

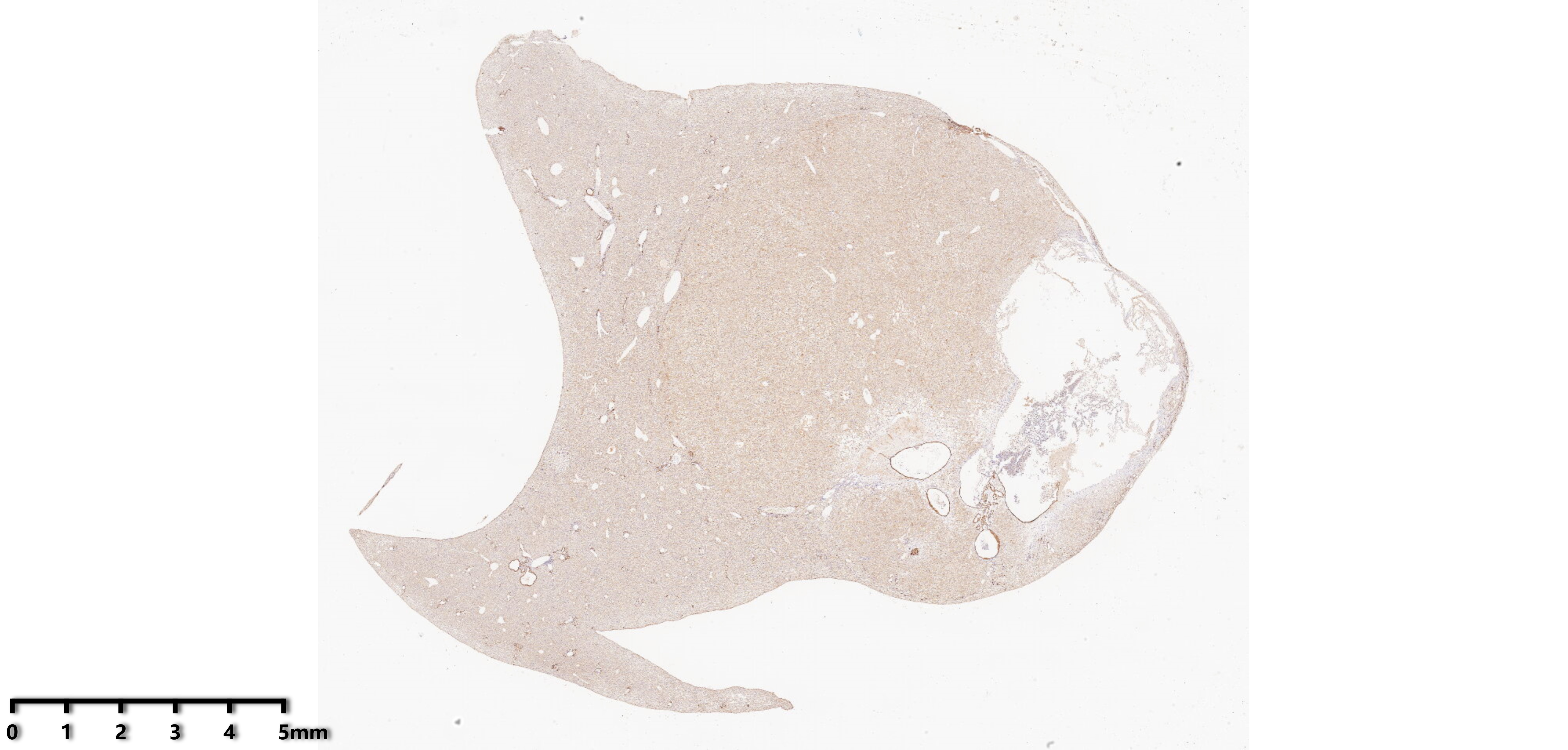

Supplement: Supplementary file 17 — Figure EV7 Source Data [file 44318_2024_353_MOESM17_ESM.zip › EVFigure 7/7I/18716CK192023-04-09_19_31_27_0.4x_1.jpg]

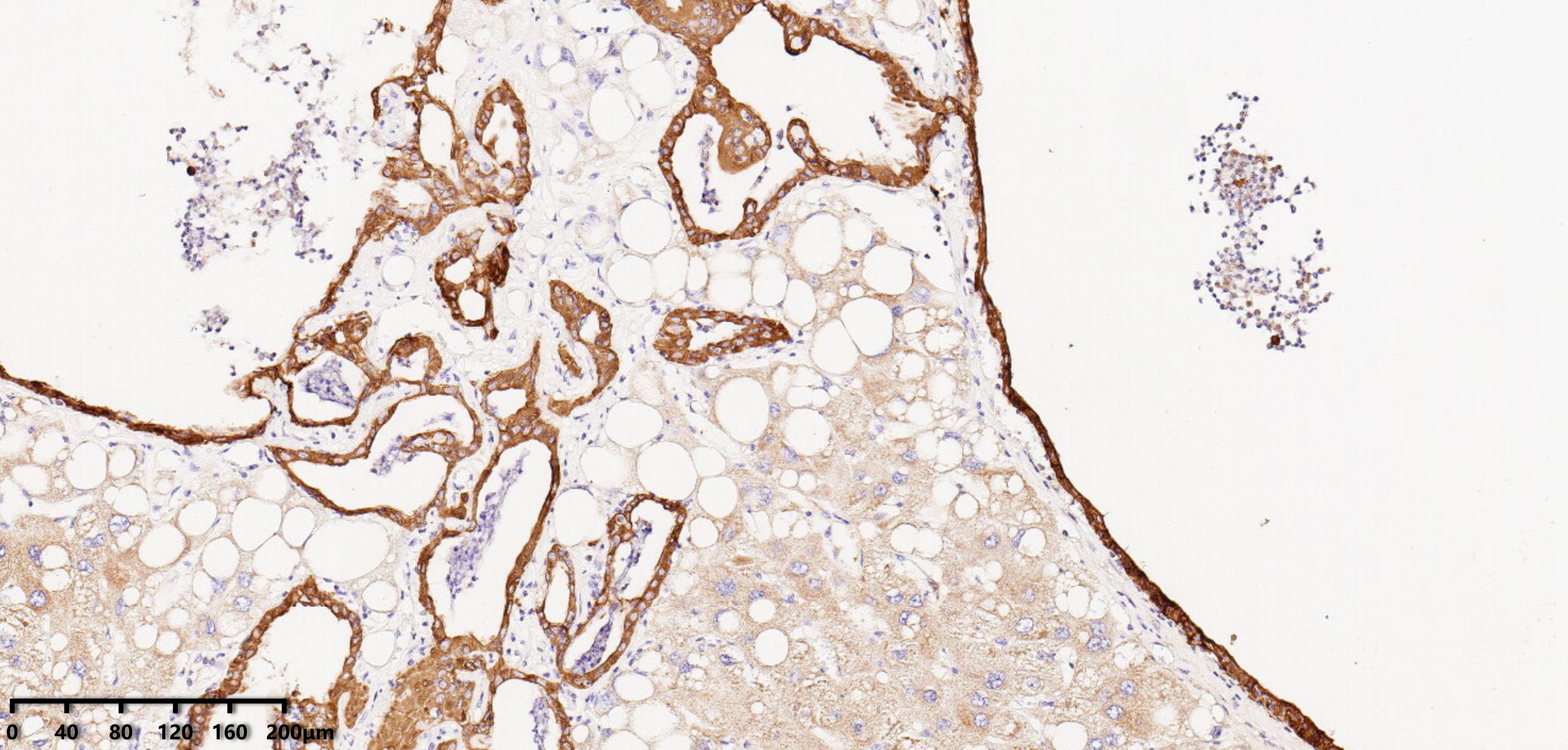

Supplement: Supplementary file 17 — Figure EV7 Source Data [file 44318_2024_353_MOESM17_ESM.zip › EVFigure 7/7I/18716CK192023-04-09_19_31_27_10x_2.jpg]
