# Supplementary material for: RHEB neddylation by the UBE2F-SAG axis enhances mTORC1 activity and aggravates liver tumorigenesis
Source: EMBO J. 2025 Jan 6;44(4):1185–219. doi: 10.1038/s44318-024-00353-5 (PMC11832924; doi:10.1038/s44318-024-00353-5)
Supplement: Supplementary file 18 — Expanded View Figures [file 44318_2024_353_MOESM18_ESM.pdf]

Expanded View Figures

Figure EV1. High UBE2F expression correlates with poor survival of liver cancer patients and UBE2F knockdown suppresses growth and survival by inducing autophagy.

(**A**, **B**) Cells stably expressing shRNAs targeting UBE2F were subjected to cell growth assay (**A**) and clonogenic survival assay (**B**). Representative images of the clonogenic assay are shown (top, **B**), and colony numbers are plotted (bottom, **B**). Data were presented as mean ± SEM from three independent experiments and analyzed by two-way ANOVA (**A**) or one-way ANOVA (**B**), respectively. The P values for the comparisons were as follows: (**A**) shGFP vs. shUBE2F-1 ($P = 5E\text{-}14$) and shGFP vs. shUBE2F-2 ($P = 5.1E\text{-}14$). (**B**) shGFP vs. shUBE2F-1 ($P = 2.41E\text{-}5$) and shGFP vs. shUBE2F-2 ($P = 1.76E\text{-}4$). ***$P < 0.001$. (**C–F**) Cells transfected with the indicated siRNA were synchronized in the G1/S phase using 2 mM thymidine to block, followed by releasing with the indicated time periods. Cells were then subjected to FACS analysis (**C**) or IB with the indicated Abs (**F**), and the percentages of cells at the G0/G1 (**D**) and G2/M phases (**E**) are shown. Data are presented as mean ± SEM from three independent experiments and analyzed by two-way ANOVA. The P values for the comparisons were as follows: (**D**) siCtrl vs. siUBE2F-1 ($P = 3.69E\text{-}4$ for 2 h; 6.19E-5 for 4 h; 0.0235 for 6 h; 0.0033 for 10 h; 0.0012 for 12 h) and siCtrl vs. siUBE2F-2 ($P = 0.007$ for 2 h; 6.03E-5 for 4 h; 0.0027 for 6 h; 0.0039 for 10 h; 6.85E-4 for 12 h). (**E**) siCtrl vs. siUBE2F-1 ($P = 3.92E\text{-}10$ for 4 h, 5.58E-5 for 6 h; 1.25E-4 for 10 h; 0.0025 for 12 h) and siCtrl vs. siUBE2F-2 ($P = 1.47E\text{-}8$ for 4 h; 1.12E-4 for 6 h; 1.92E-4 for 10 h; 5.82E-4 for 12 h). **$P < 0.01$; ***$P < 0.001$. (**G**) Cells were transfected with indicated siRNAs for 72 h, followed by IB analysis with indicated Abs. (**H**) Cells were transfected with indicated siRNAs for 72 h, autophagosomes were detected by transmission electron microscopy (TEM). The arrows indicated autophagosomes, scale bar, 1 µm. (**I**) *Ube2f*$^{fl/fl}$ MEF cells were infected with Ad-GFP or Ad-Cre adenovirus for 72 h, followed by IB analysis with indicated Abs. Source data are available online for this figure.

© The Author(s)

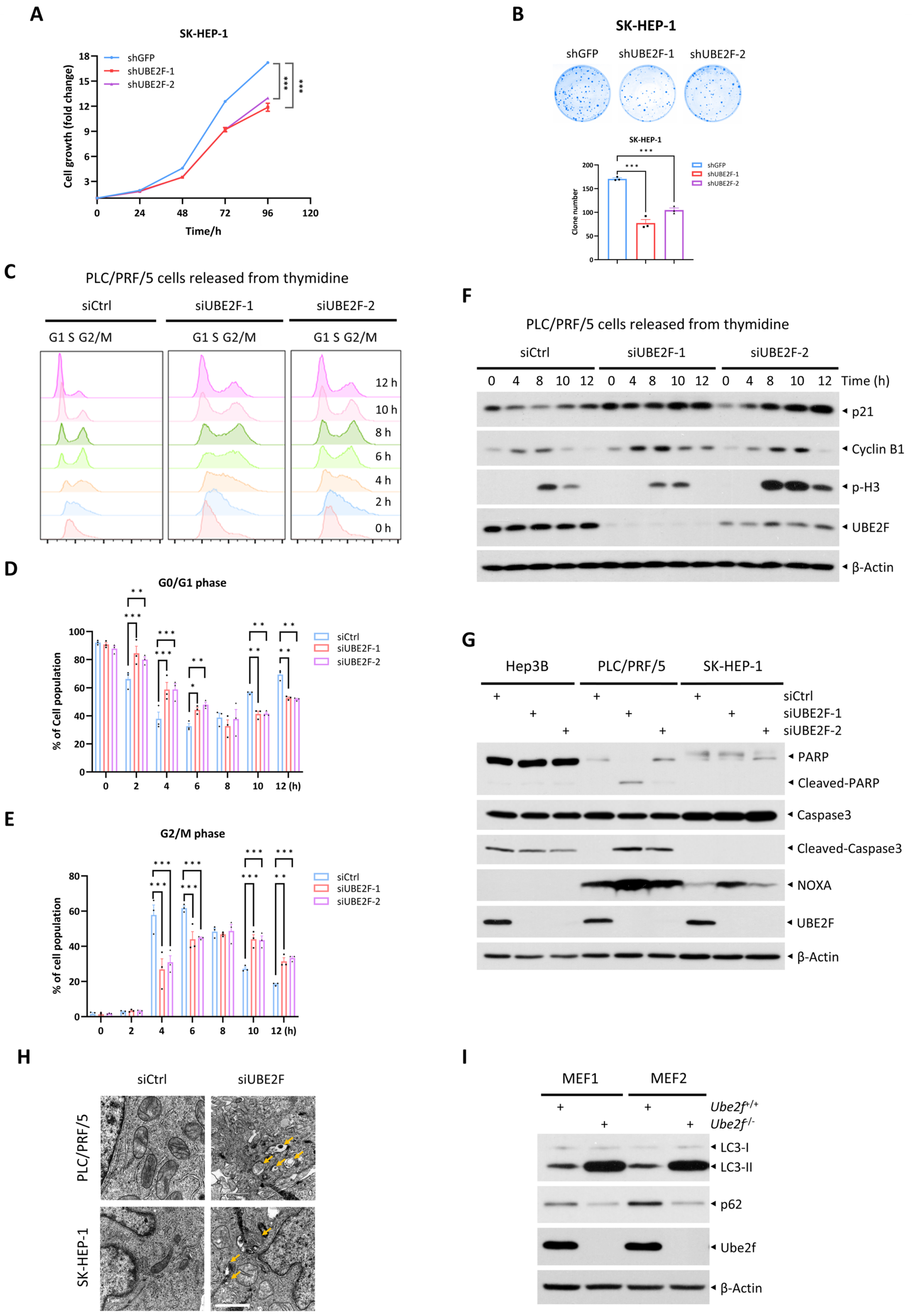
A
SK-HEP-1
shGFP
shUBE2F-1
shUBE2F-2
Cell growth (fold change)
Time/h
***
***
B
SK-HEP-1
shGFP
shUBE2F-1
shUBE2F-2
Clone number
C
PLC/PRF/5 cells released from thymidine
siCtrl
siUBE2F-1
siUBE2F-2
G1 S G2/M
12 h
10 h
8 h
6 h
4 h
2 h
0 h
D
G0/G1 phase
% of Cell population
siCtrl
siUBE2F-1
siUBE2F-2
E
G2/M phase
% of cell population
F
PLC/PRF/5 cells released from thymidine
siCtrl
siUBE2F-1
siUBE2F-2
Time (h)
p21
Cyclin B1
p-H3
UBE2F
β-Actin
G
Hep3B
PLC/PRF/5
SK-HEP-1
siCtrl
siUBE2F-1
siUBE2F-2
PARP
Cleaved-PARP
Caspase3
Cleaved-Caspase3
NOXA
UBE2F
β-Actin
H
siCtrl
siUBE2F
PLC/PRF/5
SK-HEP-1
I
MEF1
MEF2
Ube2f+/+
Ube2f-/-
LC3-I
LC3-II
p62
Ube2f
β-Actin

 © The Author(s)

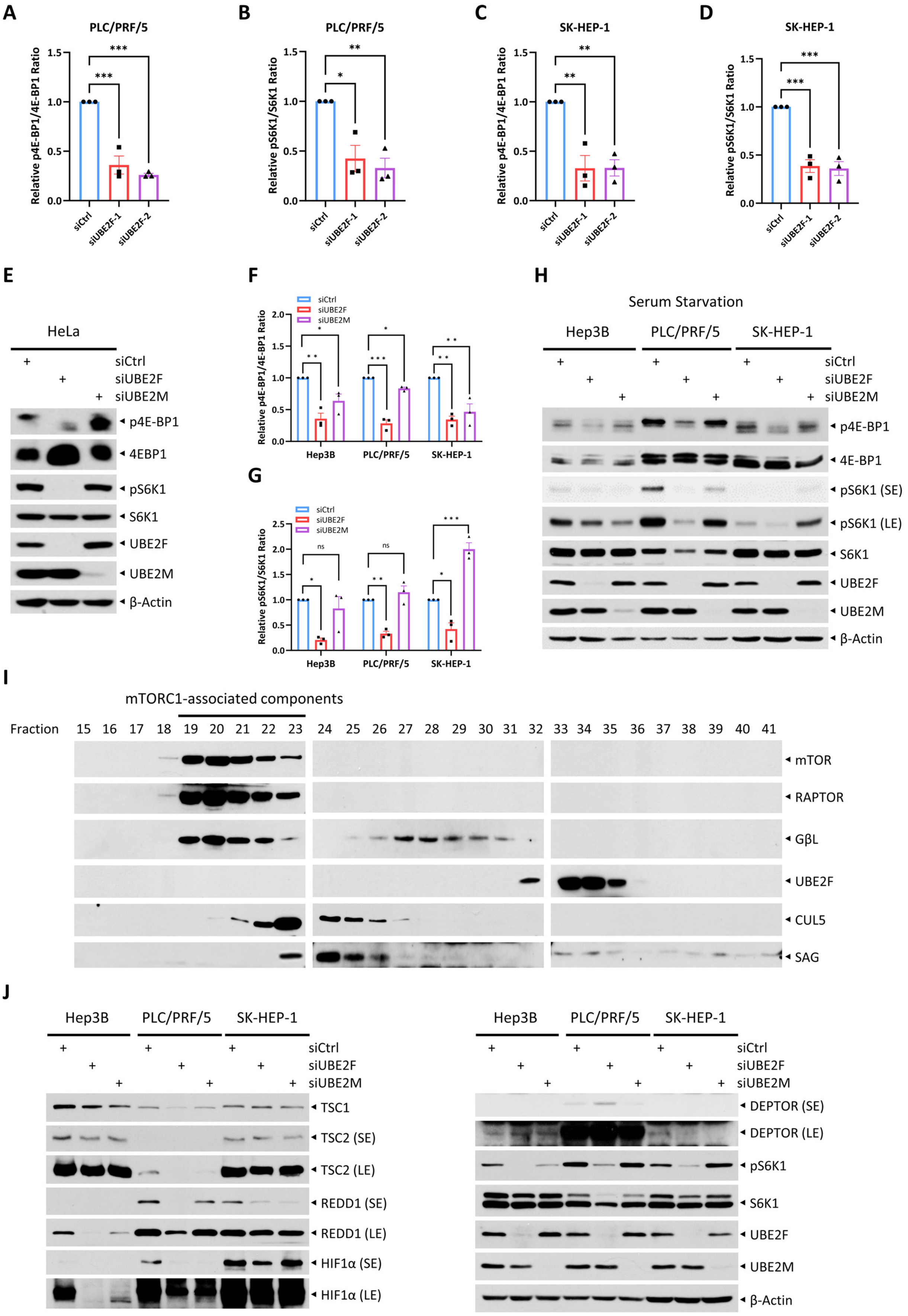
A
PLC/PRF/5
Relative p4E-BP1/4E-BP1 Ratio
siCtrl
siUBE2F-1
siUBE2F-2
B
PLC/PRF/5
Relative pS6K1/S6K1 Ratio
C
SK-HEP-1
D
SK-HEP-1
E
HeLa
siCtrl
siUBE2F
siUBE2M
p4E-BP1
4EBP1
pS6K1
S6K1
UBE2F
UBE2M
β-Actin
F
siCtrl
siUBE2F
siUBE2M
Hep3B
PLC/PRF/5
SK-HEP-1
G
H
Serum Starvation
pS6K1 (SE)
pS6K1 (LE)
I
mTORC1-associated components
Fraction
mTOR
RAPTOR
GβL
CUL5
SAG
J
TSC1
TSC2 (SE)
TSC2 (LE)
REDD1 (SE)
REDD1 (LE)
HIF1α (SE)
HIF1α (LE)
DEPTOR (SE)
DEPTOR (LE)

Figure EV2. UBE2F knockdown inactivates mTORC1 activity.

(**A–D**) Statistical analysis of the p4E-BP1/4E-BP1 and p-S6K1/S6K1 ratios of Fig. 2A. Data were presented as mean ± SEM from three independent experiments and analyzed by one-way ANOVA. The *P* values were as follows: (**A**) siCtrl vs. siUBE2F-1 ($P = 4E-4$) and siCtrl vs. siUBE2F-2 ($P = 2E-4$). (**B**) siCtrl vs. siUBE2F-1 ($P = 0.0158$) and siCtrl vs. siUBE2F-2 ($P = 0.0075$). (**C**) siCtrl vs. siUBE2F-1 ($P = 0.0041$) and siCtrl vs. siUBE2F-2 ($P = 0.0042$). (**D**) siCtrl vs. siUBE2F-1 ($P = 8E-4$) and siCtrl vs. siUBE2F-2 ($P = 6E-4$). *$P < 0.05$; **$P < 0.01$; ***$P < 0.001$. (**E**) HeLa cells were transfected with indicated siRNAs for 72 h, followed by IB with indicated Abs. (**F**, **G**) Statistical analysis of the p4E-BP1/4E-BP1 and p-S6K1/S6K1 ratios of Fig. 2B. Data were presented as mean ± SEM from three independent experiments and analyzed by one-way ANOVA. The *P* values were as follows: (**F**) Hep3B siCtrl vs. siUBE2F ($P = 0.0022$) and siCtrl vs. siUBE2M ($P = 0.0317$); PLC/PRF/5 siCtrl vs. siUBE2F ($P = 1.14E-5$) and siCtrl vs. siUBE2M ($P = 0.025$); SK-HEP-1 siCtrl vs. siUBE2F ($P = 0.0019$) and siCtrl vs. siUBE2M ($P = 0.0054$). (**G**) Hep3B siCtrl vs. siUBE2F ($P = 0.0108$) and siCtrl vs. siUBE2M ($P = 0.6087$); PLC/PRF/5 siCtrl vs. siUBE2F ($P = 0.0016$) and siCtrl vs. siUBE2M ($P = 0.3603$); SK-HEP-1 siCtrl vs. siUBE2F ($P = 0.0127$) and siCtrl vs. siUBE2M ($P = 8E-4$). *$P < 0.05$; **$P < 0.01$; ***$P < 0.001$; ns: no significant. (**H**) Cells were transfected with indicated siRNAs for 48 h, and then serum starved for 24 h before being harvested for IB analysis with indicated Abs. (**I**) SK-HEP-1 cells were lysed in CHAPS buffer. After being adjusted to 10 mg/mL of protein concentration, the lysate was passed through a 0.45 μm filter. 500 μL of the lysate was injected into the Superdex 200 10/300 GL column, and equal amounts were then collected for IB analysis. (**J**) Cells were transfected with indicated siRNAs for 72 h and then subjected to IB analysis with indicated Abs. SE short exposure, LE long exposure. Source data are available online for this figure.

 © The Author(s)

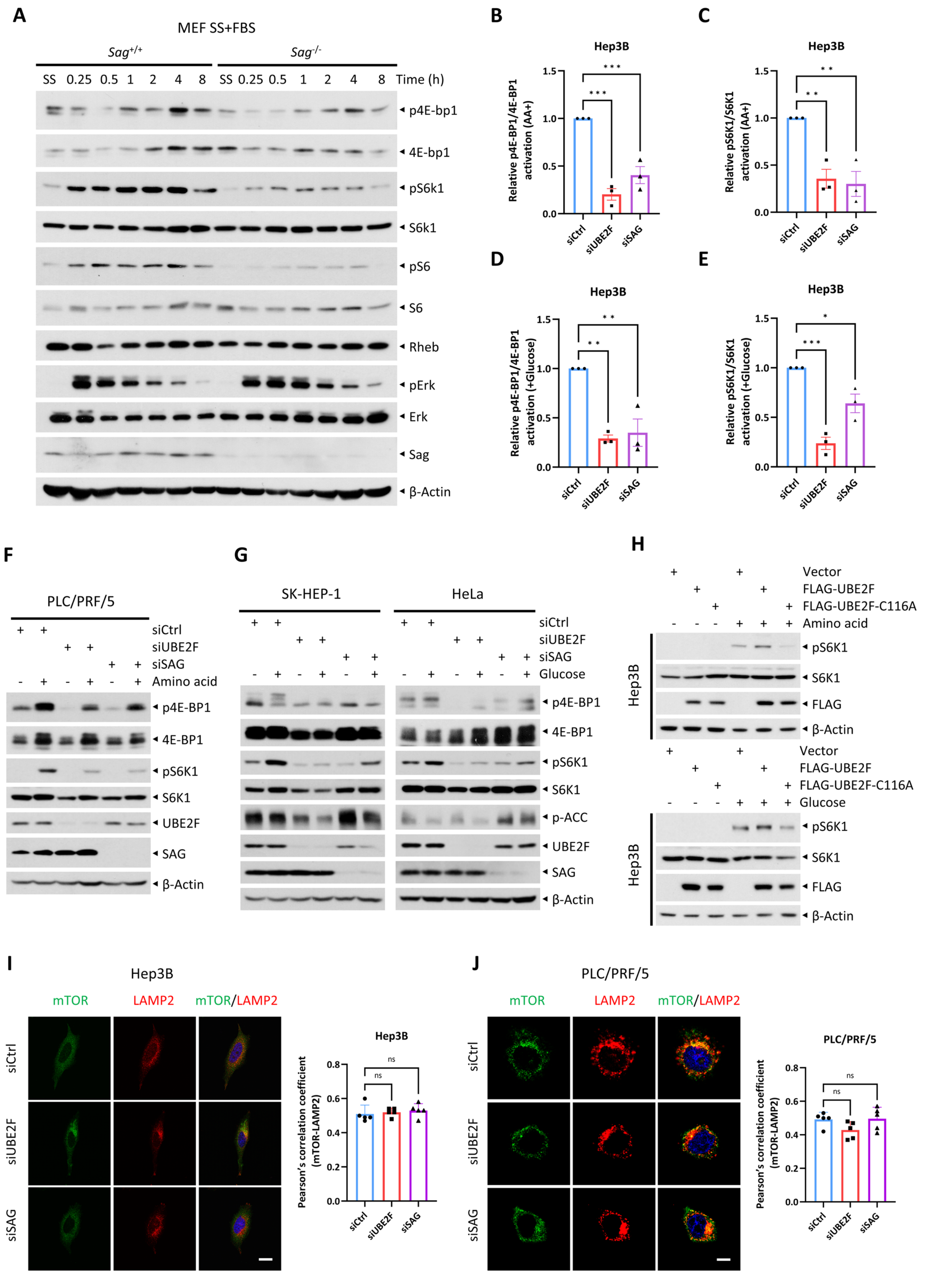
A
MEF SS+FBS
Sag+/+
Sag-/-
SS 0.25 0.5 1 2 4 8 SS 0.25 0.5 1 2 4 8 Time (h)
p4E-bp1
4E-bp1
pS6k1
S6k1
pS6
S6
Rheb
pErk
Erk
Sag
β-Actin
B
Hep3B
Relative p4E-BP1/4E-BP1 activation (AA+)
siCtrl siUBE2F siSAG
C
Hep3B
Relative pS6K1/S6K1 activation (AA+)
D
Hep3B
Relative p4E-BP1/4E-BP1 activation (+Glucose)
E
Hep3B
Relative pS6K1/S6K1 activation (+Glucose)
F
PLC/PRF/5
siCtrl
siUBE2F
siSAG
Amino acid
p4E-BP1
4E-BP1
pS6K1
S6K1
UBE2F
SAG
β-Actin
G
SK-HEP-1
HeLa
siCtrl
siUBE2F
siSAG
Glucose
p4E-BP1
4E-BP1
pS6K1
S6K1
p-ACC
UBE2F
SAG
β-Actin
H
Vector
FLAG-UBE2F
FLAG-UBE2F-C116A
Amino acid
Glucose
Hep3B
pS6K1
S6K1
FLAG
β-Actin
I
Hep3B
mTOR LAMP2 mTOR/LAMP2
siCtrl
siUBE2F
siSAG
Pearson's correlation coefficient (mTOR-LAMP2)
ns
J
PLC/PRF/5
mTOR LAMP2 mTOR/LAMP2
siCtrl
siUBE2F
siSAG
Pearson's correlation coefficient (mTOR-LAMP2)
ns

Figure EV3. The UBE2F-SAG axis regulates mTORC1 activity.

(**A**) $Sag^{+/+}$ and $Sag^{fl/fl}$ MEF cells were infected with Ad-Cre adenovirus for 72 h, and then serum starved for 24 h, followed by serum re-suppy for indicated time periods before being harvested for IB analysis with indicated Abs. (**B–E**) Statistical analysis of the p4E-BP1/4E-BP1 and p-S6K1/S6K1 ratios from Fig. 3B,C. Data were presented as mean ± SEM from three independent experiments and analyzed by one-way ANOVA. The *P* values were as follows: (**B**) siCtrl vs. siUBE2F ($P = 2E\text{-}4$) and siCtrl vs. siSAG ($P = 9E\text{-}4$). (**C**) siCtrl vs. siUBE2F ($P = 0.0063$) and siCtrl vs. siSAG ($P = 0.0042$). (**D**) siCtrl vs. siUBE2F ($P = 0.0016$) and siCtrl vs. siSAG ($P = 0.0026$). (**E**) siCtrl vs. siUBE2F ($P = 3E\text{-}4$) and siCtrl vs. siSAG ($P = 0.0138$). $^{*}P < 0.05$; $^{**}P < 0.01$; $^{***}P < 0.001$. (**F**) PLC/PRF/5 cells were transfected with indicated siRNAs for 72 h, and then deprived of amino acid for 50 min, followed by re-stimulation with amino acid for 15 min before being harvested for IB analysis with indicated Abs. (**G**) Cells were transfected with indicated siRNAs for 72 h, and then starved of glucose for 6 h, followed by restimulation with glucose for 20 min before being harvested for IB analysis with indicated Abs. (**H**) Hep3B cells transfected with 3 µg of WT-RHEB or the enzymatic-dead mutant UBE2F-C116A were subjected to amino acid or glucose deprivation, followed by refeeding before being harvested for IB analysis with the indicated Abs. (**I**, **J**) Hep3B and PLC/PRF/5 cells were transfected with indicated siRNAs for 48 h, and then immunostained for mTOR (green) and LAMP2 (red). The statistical analysis of the co-localization of mTOR and LAMP2 was performed by calculating Pearson's correlation coefficient. Data were presented as mean ± SD from five random fields and analyzed by one-way ANOVA. ns: no significant. Scale bars, 10 µm. Source data are available online for this figure.

 © The Author(s)

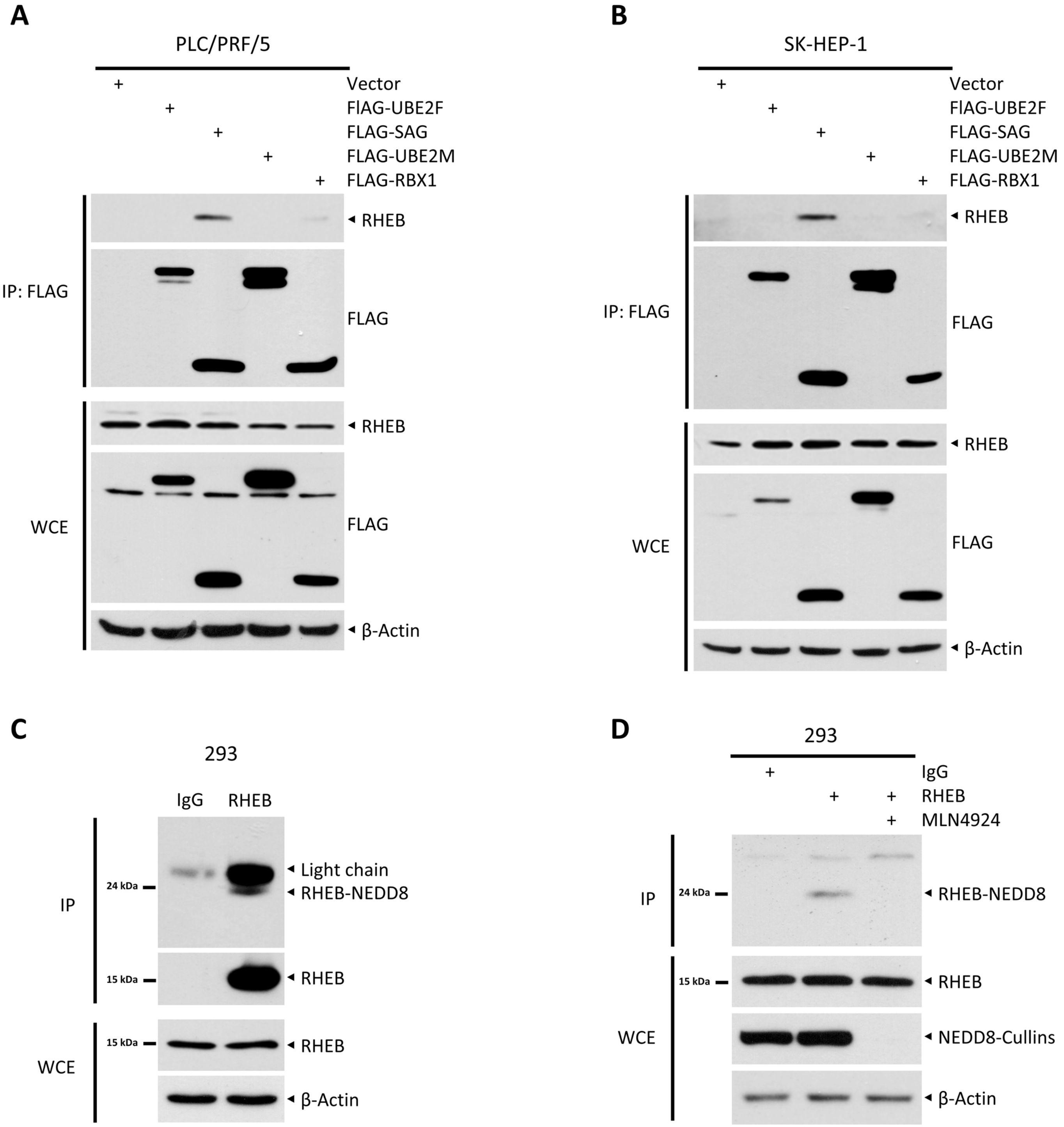

Figure EV4. The UBE2F-SAG axis promotes RHEB neddylation.

(**A**, **B**) PLC/PRF/5 (**A**) and SK-HEP-1 (**B**) cells were transfected with indicated plasmids for 48 h, and then subjected to IP with FLAG beads, followed by IB analysis with indicated Abs. (**C**) HEK293 cell lysates were incubated with anti-RHEB Ab or normal IgG as a control, followed by IB analysis with anti-NEDD8 Ab to detect endogenous RHEB neddylation. (**D**) Cells were left untreated or treated with 1 μM MLN4924 for 24 h and then subjected to IP with anti-RHEB Ab, along with normal IgG, followed by IB analysis with anti-NEDD8 Ab to detect endogenous RHEB neddylation. WCE whole cell extract. Source data are available online for this figure.

© The Author(s)

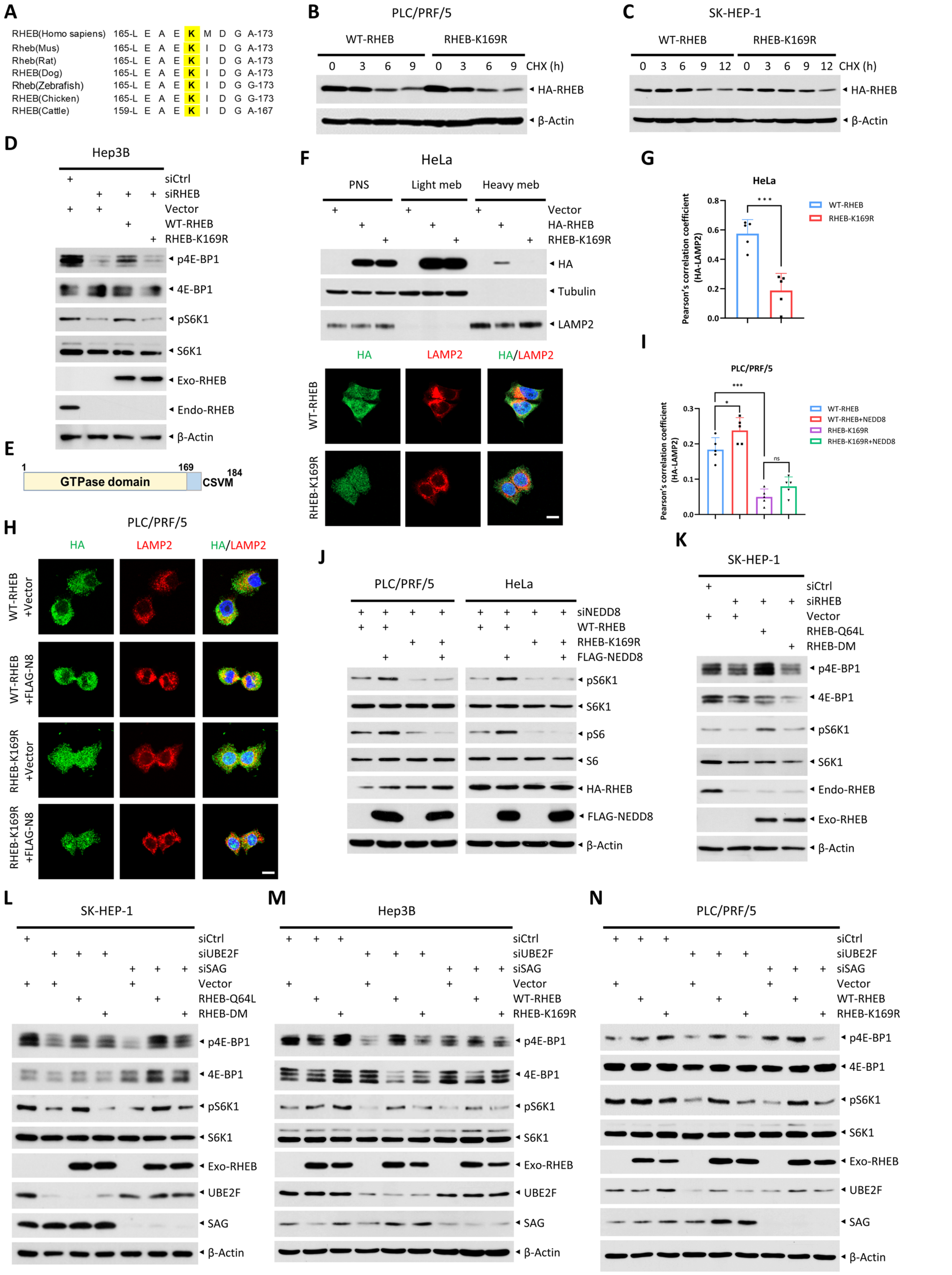
A
RHEB(Homo sapiens) 165-L E A E K M D G A-173
Rheb(Mus) 165-L E A E K I D G A-173
Rheb(Rat) 165-L E A E K I D G A-173
RHEB(Dog) 165-L E A E K I D G A-173
Rheb(Zebrafish) 165-L E A E K I D G G-173
RHEB(Chicken) 165-L E A E K I D G G-173
RHEB(Cattle) 159-L E A E K I D G A-167
B
PLC/PRF/5
WT-RHEB
RHEB-K169R
CHX (h)
HA-RHEB
β-Actin
C
SK-HEP-1
D
Hep3B
siCtrl
siRHEB
Vector
WT-RHEB
RHEB-K169R
p4E-BP1
4E-BP1
pS6K1
S6K1
Exo-RHEB
Endo-RHEB
E
GTPase domain
CSVM
F
HeLa
PNS
Light meb
Heavy meb
HA-RHEB
HA
Tubulin
LAMP2
HA/LAMP2
G
Pearson's correlation coefficient (HA-LAMP2)
H
WT-RHEB +Vector
WT-RHEB +FLAG-N8
RHEB-K169R +Vector
RHEB-K169R +FLAG-N8
I
WT-RHEB+NEDD8
RHEB-K169R+NEDD8
J
siNEDD8
FLAG-NEDD8
pS6
S6
K
RHEB-Q64L
RHEB-DM
L
siUBE2F
siSAG
UBE2F
SAG
M
N

 © The Author(s)

Figure EV5. Neddylation K169R mutant loses RHEB activity with altered lysosome localization.

(**A**) RHEB K169 residues were evolutionarily conserved and highlighted. (**B**, **C**) Cells were transfected with 3 µg HA-tagged wild type RHEB (WT-RHEB) or RHEB-K169R mutant for 48 h, and then treated with 100 µg/mL CHX for indicated time periods, followed by IB analysis with indicated Abs. (**D**) Hep3B cells were simultaneously transfected with siRNA targeting RHEB 3'UTR region along with 3 µg exogenous RHEB plasmids for 72 h, followed by IB analysis with indicated Abs. (**E**) RHEB domain structure. (**F**, **G**) HeLa cells were transfected with 15 µg HA-WT-RHEB or HA-RHEB-K169R mutant per 15 cm dish for 48 h, and then subjected to IB analysis with indicated Abs after isolation of heavy membrane and light membrane fractions (top, **F**), or immunofluorescent labeling of HA (green) and LAMP2 (red) (bottom, **F**). The statistical analysis of the co-localization of HA and LAMP2 was performed by calculating Pearson's correlation coefficient (**G**). Data were presented as mean ± SD from five random fields and analyzed by Student's *t* test, $P = 4E\text{-}4$. ***$P < 0.001$. PNS: post-nuclear supernatants. Scale bar, 10 µm. (**H**–**J**) PLC/PRF/5 and HeLa cells were co-transfected with 1.5 µg of RHEB and 2 µg of NEDD8 plasmids for 48 h. Cells were subjected to immunofluorescent labeling of HA (green) and LAMP2 (red) (**H**) or IB analysis (**J**). The statistical analysis of the co-localization of HA and LAMP2 was performed by calculating Pearson's correlation coefficient (**I**). Data were presented as mean ± SD from five random fields and analyzed by Student's *t* test. The *P* values for the comparisons were as follows: WT-RHEB vs. WT-RHEB + NEDD8 ($P = 0.0406$); WT-RHEB vs. RHEB-K169R ($P = 7.46E\text{-}5$); RHEB-K169R vs. RHEB-K169R + NEDD8 ($P = 0.0888$). Scale bar, 10 µm, *$P < 0.05$; ***$P < 0.001$; ns: no significant. (**K**) SK-HEP-1 cells were simultaneously transfected with siRNA targeting the 3'UTR region of RHEB, along with 3 µg of exogenous RHEB plasmids for 72 h, followed by IB analysis with the indicated Abs. (**L**–**N**) Cells were transfected with indicated siRNA and plamids for 72 h, followed by IB analysis with indicated Abs. RHEB-DM: RHEB-Q64L-K169R (**K**, **L**). Source data are available online for this figure.

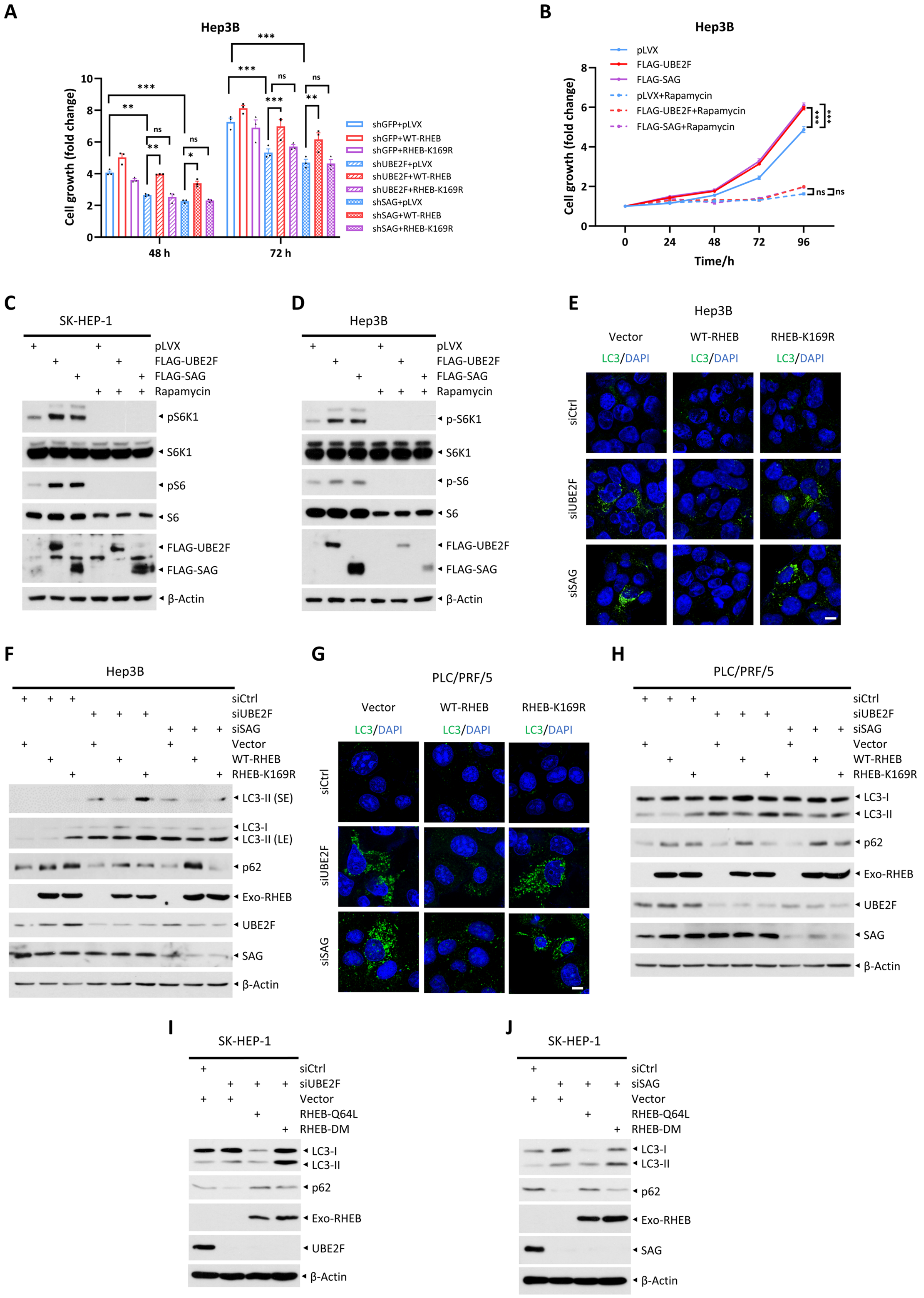
A
Hep3B
Cell growth (fold change)
48 h
72 h
shGFP+pLVX
shGFP+WT-RHEB
shGFP+RHEB-K169R
shUBE2F+pLVX
shUBE2F+WT-RHEB
shUBE2F+RHEB-K169R
shSAG+pLVX
shSAG+WT-RHEB
shSAG+RHEB-K169R
B
Hep3B
pLVX
FLAG-UBE2F
FLAG-SAG
pLVX+Rapamycin
FLAG-UBE2F+Rapamycin
FLAG-SAG+Rapamycin
Cell growth (fold change)
Time/h
C
SK-HEP-1
pLVX
FLAG-UBE2F
FLAG-SAG
Rapamycin
pS6K1
S6K1
pS6
S6
FLAG-UBE2F
FLAG-SAG
β-Actin
D
Hep3B
pLVX
FLAG-UBE2F
FLAG-SAG
Rapamycin
p-S6K1
S6K1
p-S6
S6
FLAG-UBE2F
FLAG-SAG
β-Actin
E
Hep3B
Vector
WT-RHEB
RHEB-K169R
LC3/DAPI
siCtrl
siUBE2F
siSAG
F
Hep3B
siCtrl
siUBE2F
siSAG
Vector
WT-RHEB
RHEB-K169R
LC3-II (SE)
LC3-I
LC3-II (LE)
p62
Exo-RHEB
UBE2F
SAG
β-Actin
G
PLC/PRF/5
Vector
WT-RHEB
RHEB-K169R
LC3/DAPI
siCtrl
siUBE2F
siSAG
H
PLC/PRF/5
siCtrl
siUBE2F
siSAG
Vector
WT-RHEB
RHEB-K169R
LC3-I
LC3-II
p62
Exo-RHEB
UBE2F
SAG
β-Actin
I
SK-HEP-1
siCtrl
siUBE2F
Vector
RHEB-Q64L
RHEB-DM
LC3-I
LC3-II
p62
Exo-RHEB
UBE2F
β-Actin
J
SK-HEP-1
siCtrl
siSAG
Vector
RHEB-Q64L
RHEB-DM
LC3-I
LC3-II
p62
Exo-RHEB
SAG
β-Actin

 © The Author(s)

Figure EV6. RHEB-K169R mutant fails to rescue altered growth phenotypes induced by knockdown of UBE2F or SAG.

(**A**) Hep3B cells stably expressing shRNA and plasmids were subjected to cell growth assay. Data were presented as mean ± SEM from three independent experiments and analyzed by two-way ANOVA. The *P* values for the various comparisons were as follows: shGFP+pLVX vs. shUBE2F+pLVX ($P = 0.0036$ for 48 h and 3.57E-5 for 72 h); shGFP+pLVX vs. shSAG+pLVX ($P = 1.11E\text{-}4$ for 48 h and 1.16E-7 for 72 h); shUBE2F+pLVX vs. shUBE2F+WT-RHEB ($P = 0.0084$ for 48 h and 4.48E-4 for 72 h); shUBE2F +pLVX vs. shUBE2F+RHEB-K169R ($P = 0.9999$ for 48 h and 0.9618 for 72 h); shSAG+pLVX vs. shSAG+WT-RHEB ($P = 0.035$ for 48 h and 0.0024 for 72 h); shSAG +pLVX vs. shSAG+RHEB-K169R ($P = 0.9999$ for both 48 h and 72 h). *$P < 0.05$; **$P < 0.01$; ***$P < 0.001$; ns: no significant. (**B**) Hep3B cells were infected with lentivirus-based FLAG-UBE2F or FLAG-SAG plasmids, cultured with 2.5% FBS, and then treated with or without 1 μM rapamycin, followed by cell growth assay. Data were presented as mean ± SEM from three independent experiments and analyzed by two-way ANOVA. The *P* values for the comparisons were as follows: pLVX vs. FLAG-UBE2F ($p = 1.9924E\text{-}11$); pLVX vs. FLAG-SAG ($P = 1.9904E\text{-}11$); pLVX+Rapamycin vs. FLAG-UBE2F+Rapamycin ($P = 0.0867$); pLVX+Rapamycin vs. FLAG-SAG+Rapamycin ($P = 0.0858$). ***$P < 0.001$; ns: no significant. (**C**, **D**) Hep3B and SK-HEP-1 cells were infected with the indicated expressing lentivirus and then treated with 100 nM rapamycin for 24 h in 2.5% FBS before being harvested for IB analysis. (**E–H**) Hep3B and PLC/PRF/5 cells were transfected with the indicated siRNA and plasmids for 48 h, followed by co-staining of LC3 (green) and DAPI (blue) (**E**, **G**) or IB analysis with the indicated Abs (**F**, **H**). Scale bar, 10 μm. (**I**, **J**) Cells were transfected with indicated siRNA and plasmids for 72 h, followed by IB analysis with indicated Abs. Source data are available online for this figure.

© The Author(s)

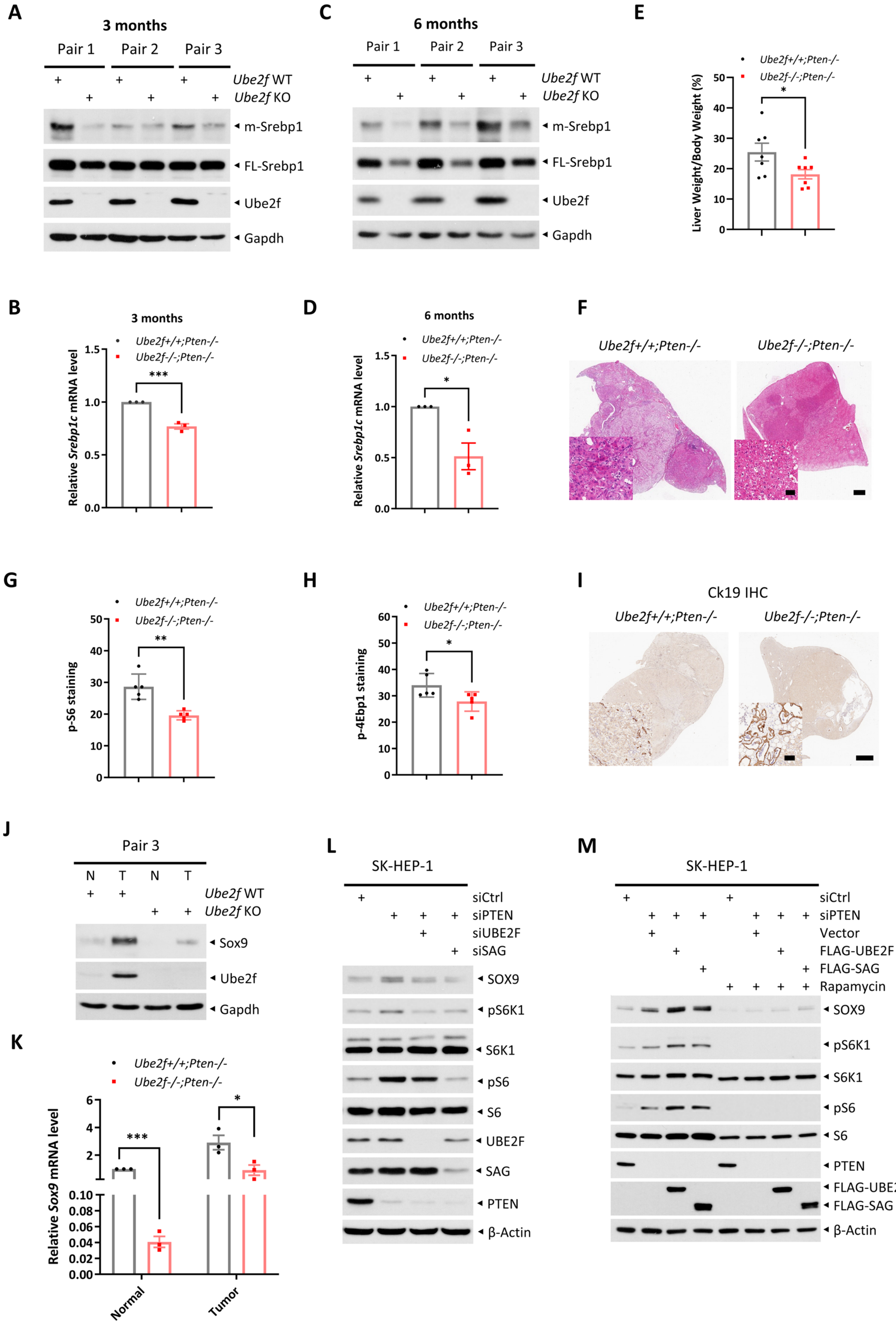
A
3 months
Pair 1 Pair 2 Pair 3
Ube2f WT
Ube2f KO
m-Srebp1
FL-Srebp1
Ube2f
Gapdh
C
6 months
Pair 1 Pair 2 Pair 3
Ube2f WT
Ube2f KO
m-Srebp1
FL-Srebp1
Ube2f
Gapdh
E
Ube2f+/+;Pten-/-
Ube2f-/-;Pten-/-
Liver Weight/Body Weight (%)
B
3 months
Ube2f+/+;Pten-/-
Ube2f-/-;Pten-/-
Relative Srebp1c mRNA level
D
6 months
Ube2f+/+;Pten-/-
Ube2f-/-;Pten-/-
Relative Srebp1c mRNA level
F
Ube2f+/+;Pten-/-
Ube2f-/-;Pten-/-
G
Ube2f+/+;Pten-/-
Ube2f-/-;Pten-/-
p-S6 staining
H
Ube2f+/+;Pten-/-
Ube2f-/-;Pten-/-
p-4Ebp1 staining
I
Ck19 IHC
Ube2f+/+;Pten-/-
Ube2f-/-;Pten-/-
J
Pair 3
N T N T
Ube2f WT
Ube2f KO
Sox9
Ube2f
Gapdh
K
Ube2f+/+;Pten-/-
Ube2f-/-;Pten-/-
Relative Sox9 mRNA level
Normal
Tumor
L
SK-HEP-1
siCtrl
siPTEN
siUBE2F
siSAG
SOX9
pS6K1
S6K1
pS6
S6
UBE2F
SAG
PTEN
β-Actin
M
SK-HEP-1
siCtrl
siPTEN
Vector
FLAG-UBE2F
FLAG-SAG
Rapamycin
SOX9
pS6K1
S6K1
pS6
S6
PTEN
FLAG-UBE2F
FLAG-SAG
β-Actin

 © The Author(s)

Figure EV7. ***Ube2f*** **knockout attenuates liver steatosis and tumorigenesis in vivo.**

(**A–D**) Liver tissues were isolated from paired *Ube2f+/+;Pten-/-* (Ube2f WT) and *Ube2f-/-;Pten-/-* (*Ube2f* KO) mice at the ages of 3 and 6 months and then subjected to IB analysis with indicated Abs (A&C) or qRT-PCR analysis (B&D). Data were shown as mean ± SEM and analyzed by Student's *t* test. $n = 3$ for each genotype. The *P* values were 7E-4 for (**B**) and 0.0199 for (**D**). *$P < 0.05$; ***$P < 0.001$. (**E**) The ratios of liver/body weight of Ube2f WT and KO mice at 12-month old were shown as mean ± SEM and analyzed by Student's *t* test. $P = 0.0471$, $n = 7$ for each genotype; *$P < 0.05$. (**F**) H&E staining of liver tissues from 12-month-old Ube2f WT and KO mice. Scale bars, 1 mm and 40 μm (inset), respectively. (**G**, **H**) Quantification of p-S6 (**G**) and p-4Ebp1 (**H**) staining from Fig. 7E. Data were shown as mean ± SD from five random fields and analyzed by Student's *t* test. The *P* values were 0.0014 for (**G**) and 0.0449 for (**H**). *$P < 0.05$; **$P < 0.01$. (**I**) Ck-19 staining of liver sections from 12-month-old Ube2f WT and KO mice. Scale bars, 2 mm and 80 μm (inset), respectively. (**J**, **K**) Liver tissues from 12-month-old Ube2f WT and KO mice were harvested for IB (**J**) or qRT-PCR analysis (**K**). Data were shown as mean ± SEM and analyzed by Student's *t* test. $n = 3$ for each genotype. The *P* values were as follows: normal tissue (Ube2f WT vs. KO, $P = 1.7$E-8) and tumor tissue (Ube2f WT vs. KO, $P = 0.0352$). *$P < 0.05$; ***$P < 0.001$. (**L**, **M**) SK-HEP-1 cells were transfected with the indicated siRNAs (**L**) or with 3 μg of the indicated plasmids (**M**) for 72 h. The cells were then treated with or without 100 nM rapamycin for 48 h before being harvested for IB with indicated Abs. Source data are available online for this figure.

© The Author(s)
